# Target mimics: an embedded layer of microRNA-involved gene regulatory networks in plants

**DOI:** 10.1186/1471-2164-13-197

**Published:** 2012-05-21

**Authors:** Yijun Meng, Chaogang Shao, Huizhong Wang, Yongfeng Jin

**Affiliations:** 1 College of Life and Environmental Sciences, Hangzhou Normal University, Xuelin Street 16#, Xiasha, Hangzhou, 310036, P. R. China; 2 College of Life Sciences, Huzhou Teachers College, Huzhou, 313000, P. R. China; 3 Institute of Biochemistry, College of Life Sciences, Zhejiang University, Zijingang Campus, Yu Hang Tang Road 866#, Hangzhou, 310058, P. R. China

**Keywords:** Target mimic, Network, Subnetwork, MicroRNA, Degradome, Arabidopsis (*Arabidopsis thaliana*), Rice (*Oryza sativa*)

## Abstract

**Background:**

MicroRNAs (miRNAs) play an essential role in gene regulation in plants. At the same time, the expression of miRNA genes is also tightly controlled. Recently, a novel mechanism called “target mimicry” was discovered, providing another layer for modulating miRNA activities. However, except for the artificial target mimics manipulated for functional studies on certain miRNA genes, only one example, IPS1 (Induced by Phosphate Starvation 1)—miR399 was experimentally confirmed in planta. To date, few analyses for comprehensive identification of natural target mimics have been performed in plants. Thus, limited evidences are available to provide detailed information for interrogating the questionable issue whether target mimicry was widespread in planta, and implicated in certain biological processes.

**Results:**

In this study, genome-wide computational prediction of endogenous miRNA mimics was performed in Arabidopsis and rice, and dozens of target mimics were identified. In contrast to a recent report, the densities of target mimic sites were found to be much higher within the untranslated regions (UTRs) when compared to those within the coding sequences (CDSs) in both plants. Some novel sequence characteristics were observed for the miRNAs that were potentially regulated by the target mimics. GO (Gene Ontology) term enrichment analysis revealed some functional insights into the predicted mimics. After degradome sequencing data-based identification of miRNA targets, the regulatory networks constituted by target mimics, miRNAs and their downstream targets were constructed, and some intriguing subnetworks were further exploited.

**Conclusions:**

These results together suggest that target mimicry may be widely implicated in regulating miRNA activities in planta, and we hope this study could expand the current understanding of miRNA-involved regulatory networks.

## Background

MicroRNAs, the most sophisticatedly characterized small RNA (sRNA) species, were shown to play essential regulatory roles in gene expression in plants [1,2]. Based on the high complementarity of the recognition sites on their targets, the plant miRNAs exert repressive roles mostly through target RNA cleavages at post-transcriptional level [2]. Similar to the protein-coding genes, a dominant portion of miRNA genes are transcribed by RNA polymerase II [3-5]. At the same time, the biogenesis and the activities of these critical small molecules themselves were under tight surveillance transcriptionally or post-transcriptionally [6].

One novel mechanism involved in modulating miRNA activities in plants was unraveled by Franco-Zorrilla et al. (2007) [7]. A 23-nt-long motif was observed to be highly conserved among the phosphate starvation-induced, non-coding RNAs transcribed from the TPSI family genes including IPS1 and At4. In Arabidopsis, this motif could be recognized by miR399, but could not serve as an effective target cleavage site due to a 3-nt bulge on the “target” RNA sequence opposite the position 10^th^ to 11^th^ nt of miR399 which is the canonical slicing site. Intriguingly, the non-cleavable transcript acts as a target mimic to sequester the corresponding miRNA, thus reducing the active level of miR399. Based on this result, the term “target mimicry” was coined to describe the target mimic—miRNA regulatory relationships. By generating artificial mimics, the authors demonstrated that “target mimicry” might be not only implicated in phosphate signaling, but also in other biological processes, and the mechanisms might be widespread in plants [7]. By using the IPS1 transcript as a scaffold, the subsequent research efforts generated a collection of target mimics in Arabidopsis [8,9], which were valuable for functional studies on certain miRNA genes.

To date, however, only IPS1—miR399 has been experimentally identified as an example of target mimicry that exists in planta naturally. Although dozens of manipulated target mimics have shown great potential for modulating the activities of specific miRNA genes, the widespread existence of the related mechanism in plants remains to be a pressing question. Only one study by Ivashuta et al. (2011) was performed to partially uncover the natural target mimics of the miRNAs in Arabidopsis [8]. However, no in-depth analysis was performed except for some basic statistical results. Besides, the old version of the miRNA registries (miRBase release 15 previously used vs. miRBase 17 currently available) [10] and the gene model annotations [TAIR (The Arabidopsis Information Resource) 9 vs. TAIR 10] [11] utilized in that study, and the exclusion of the currently available non-coding gene information may lead to insufficient exploration on this topic [8].

Here, by using the latest versions of the gene annotations from TAIR (release 10) [11] and TIGR rice (The Institute for Genome Research, release 6.1; currently named the J. Craig Venter institute) [12], genome-wide in silico prediction of potential target mimics was performed for all the registered miRNAs of Arabidopsis and rice in miRBase (release 17) [10]. The miRNAs predicted to be sequestered by certain transcripts were further included for degradome sequencing data-based identification of the downstream targets. Combining these two results, numerous target mimic—miRNA—target regulatory relationships were extracted for comprehensive network construction. Certain subnetworks were further analyzed, and some interesting findings were provided.

## Results and discussion

### Transcriptome-wide prediction of natural target mimics of plant miRNAs

The latest versions of gene model annotations of Arabidopsis and rice were retrieved from TAIR [11] and TIGR rice [12], respectively, serving as the transcript database for the following prediction. All the miRBase-registered miRNAs of both plants (release 17) were included to search for their complementary sites on the gene transcripts by using the tool Ssearch belonging to the FASTA3 package [13,14]. Then, the search results were filtered to identify the potential target mimics of certain miRNAs according to the rules established based on the previous experimental experiences [7-9] (see Methods for detailed rule-based filtering). As a result, 300 and 260 mimic—miRNA interactions were identified, involving 137 and 155 different mature miRNAs in Arabidopsis and rice, respectively (Additional file 1: Table S1 and Additional file 2: Table S2). In Ivashuta et al.’s study (2011), only a limited set of non-coding transcripts from TAIR were included for target mimic prediction [8]. Thus, the question whether the non-coding RNAs tend to be more or less likely to function as target mimics needs to be addressed. To interrogate this issue, most currently available non-coding RNA sequences were obtained from Genomic tRNA Database [15] and NONCODE [16,17]. A same Ssearch- and rule-based identification of target mimics was carried out. Surprisingly, only one mimic of ath-miR418, tRNA238-LysTTT on chromosome 1, was predicted to be a potential candidate in Arabidopsis (Additional file 1: Table S1).

### Sequence characteristics of the target mimic sites and the sequestered miRNAs

**Figure 1:**
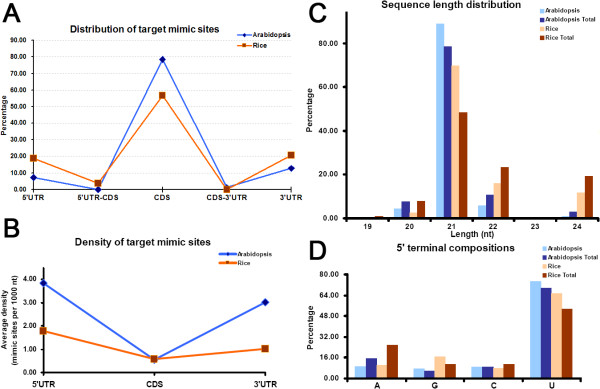
**Sequence characteristics of the sequestered microRNAs in Arabidopsis and rice.** (**A**) Statistical results of the distribution of the predicted target mimic sites along the transcripts. Each percentage was calculated by dividing the number of the target mimic sites located within a specific region by the total number of the mimic sites in the plant. Five regions on the target mimic transcripts were defined according to the sequence information provided by TAIR (The Arabidopsis Information Resource, release 10) [11] and TIGR rice (The Institute for Genome Research, release 6.1; currently named the J. Craig Venter institute) [12]: 5’ UTR (untranslated region), 3’ UTR, CDS (coding sequence), the boundary between 5’ UTR and the 5’ end of the CDS (5’ UTR—CDS), and the boundary between 3’ UTR and the 3’ end of the CDS (CDS—3’ UTR). (**B**) Distribution densities of the predicted target mimic sites. Each normalized density (number of mimic sites/1000 nt) was calculated by dividing the total number of the mimic sites within each region by the summed length of the corresponding region sequences. Three regions on the target mimic transcripts, i.e. 5’ UTR, 3’ UTR and CDS, were defined as described in (**A**). (**C**) Length distribution of the sequestered microRNAs. (**D**) 5’ terminal compositions of the sequestered microRNAs. For (**C**) and (**D**), all the microRNAs registered in the miRBase (release 17) were included as controls (see “Arabidopsis Total” and “Rice Total”).

Then, the distribution patterns of the predicted target mimic sites on the corresponding transcripts were analyzed (see detail in Methods). Quite consistent in both plants, a dominant portion of the mimic sites reside within the CDS (coding sequence) regions (78.53% of the mimic sites in Arabidopsis, and 56.60% in rice), although large portion locate within the UTRs (untranslated regions) (20.25% in Arabidopsis, and 39.62% in rice) (Figure 1A). Notably, more target mimic sites tend to distribute within the 3’ UTRs (12.88% in Arabidopsis, and 20.75% in rice) compared to the 5’ UTRs (7.36% in Arabidopsis, and 18.87% in rice) in both plants. To calculate the distribution density of the mimic sites, the total number of the mimic sites belonging to each region category (i.e. 5’ UTR, 3’ UTR or CDS) was divided by the summed length of the corresponding region sequences. Thus, a normalized distribution density (number of mimic sites/1000 nt) was generated for each region category. Surprisingly, the result presented a quite different view when compared to the result in Figure 1A. The densities of the target mimic sites are much higher in UTRs (3.83 sites/1000 nt within 5’ UTRs and 3.03 sites/1000 nt within 3’ UTRs in Arabidopsis, and 1.77 sites/1000 nt within 5’ UTRs and 1.02 sites/1000 nt within 3’ UTRs in rice) when compared to those in CDS regions (0.54 sites/1000 nt in Arabidopsis, and 0.56 sites/1000 nt in rice) in both plants (Figure 1B). It is also quite different from the previous observation that no significant difference was observed among the three categorized regions in Arabidopsis (~0.6 sites/1000 nt for 5’ UTRs, ~0.5 sites/1000 nt for CDSs, and ~0.4 sites/1000 nt for 3’ UTRs) [8]. We attributed this discrepancy to the different rules employed for target mimic site identification, since our search criteria were more stringent than the ones used in the previous study. Our statistical result raised the presumption that the non-coding regions of the gene transcripts, i.e. the UTRs, might be preferentially selected to serve as target mimic site-containing regions in plants. However, this possibility still needs verification.

**Figure 2:**
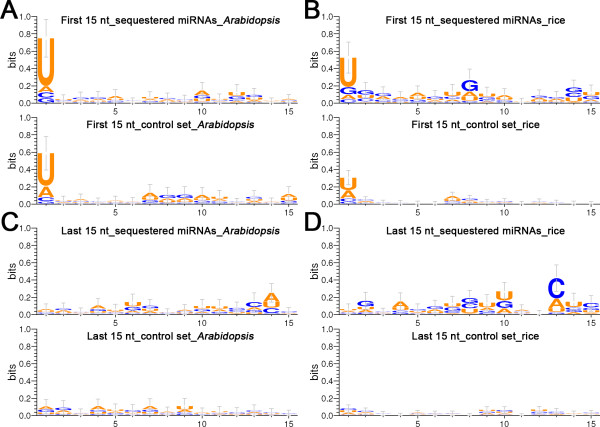
**Sequence conservation analysis of the sequestered microRNAs.** (**A**) Analysis of the first 15-nt sequences at the 5’ ends of all the sequestered microRNAs in Arabidopsis. (**B**) Analysis of the first 15-nt sequences at the 5’ ends of all the sequestered microRNAs in rice. (**C**) Analysis of the last 15-nt sequences at the 3’ ends of all the sequestered microRNAs in Arabidopsis. (**D**) Analysis of the last 15-nt sequences at the 3’ ends of all the sequestered microRNAs in rice. For (**A**) to (**D**), all the miRBase-registered microRNAs (release 17) excluding the sequestered microRNAs of Arabidopsis and rice were treated as the control sets, and the results are shown in the lower panels of (**A**) to (**D**). All the sequences were analyzed from the 5’ ends to the 3’ ends. The analysis was performed by using WebLogo 3 (http://weblogo.threeplusone.com/) [18,19].

Next, the sequence features of the sequestered miRNAs were characterized. Compared to all the miRBase-annotated miRNAs of Arabidopsis and rice, the sequence length of the sequestered miRNAs tends to be more enriched in 21 nt, and less enriched in 20, 22, and 24 nt (Figure 1C). Moreover, the 5’ terminal compositions of the sequestered miRNAs tend to be more enriched in U (uridine), and less in A (adenine) (Figure 1D). The first 15-nt sequences at both 5’ and 3’ ends of the sequestered miRNAs were collected for sequence conservation analysis. When compared to the control sets (all the miRBase-registered mature miRNAs of Arabidopsis and rice, excluding the sequestered miRNAs, were treated as the control sets), in both plants, the sequestered miRNAs preferentially start with 5’ U (Figure 2A and 2B), which was consistent with the above statistical results. More interestingly, the third nucleotides at the 3’ ends of the sequestered miRNAs were found to be dominantly occupied by C (cytosine) in rice, although it was less prominent in Arabidopsis (Figure 2C and 2D). Further investigation of the canonical example, IPS1—miR399, revealed that all the miR399 family members of both Arabidopsis and rice were 21 nt in length, and started with 5’ U, and the third nucleotides at their 3’ ends were C, only one exception was observed that the third nucleotide at the 3’ end of ath-miR399e was U. In this regard, whether these features, such as the third nucleotide at the 3’ end, could determine the probability that a specific miRNA is regulated by certain target mimic(s) needs to be further interrogated. Together, all the evidences point to the fact that the miRNAs tending to be sequestered by specific target mimics are more likely to possess the classic sequence features of plant miRNAs [6].

### Functional analysis of the target mimics

**Figure 3:**
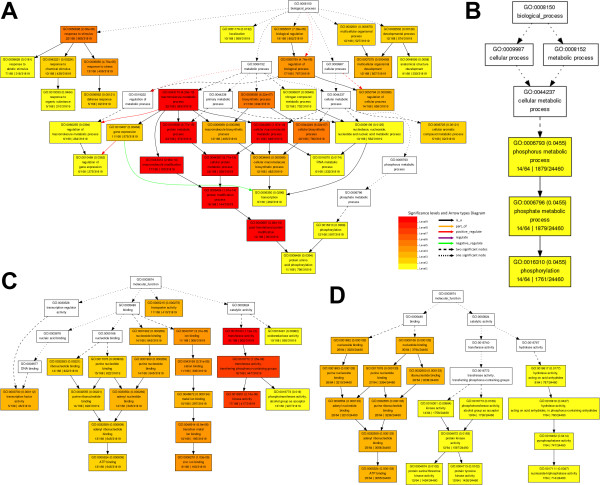
**GO (Gene Ontology) term enrichment analysis of the target mimic genes.** (**A**) Analysis of Arabidopsis target mimics within the “Biological Process” category. (**B**) Analysis of rice target mimics within the “Biological Process” category. (**C**) Analysis of Arabidopsis target mimics within the “Molecular Function” category. (**D**) Analysis of rice target mimics within the “Molecular Function” category. This analysis was performed by using the online tool agriGO [20], selecting the “Arabidopsis genome locus (TAIR)” or the “Rice TIGR locus” as a control set. For (**A**) to (**D**), the figure keys are shown at the centre of the figure.

All the target mimics were included for GO term enrichment analysis by using agriGO [20]. Intriguingly, many identified enriched GO terms were highly conserved between the two plants analyzed. For example, within the “Biological Process” category, the phosphorus metabolism-related processes such as “phosphorylation” were indicated to be enriched processes that the mimics were involved in (Figure 3A and 3B). Supporting this observation, within the “Molecular Function” category, “ATP binding” and “kinase activity” which were implicated in phosphorus metabolism, were highly enriched functions possessed by the sets of target mimics in both plants (Figure 3C and 3D). Besides, the GO terms “transcription factor activity” and “zinc ion binding” were also highly enriched in the target mimic gene set of Arabidopsis. Based on the gene annotations provided by TAIR and TIGR, we found that a portion of the target mimics were encoded by transposable element (TE) genes (4.59% in Arabidopsis and 12.59% in rice), indicating a novel functional activity of the TEs for regulating the miRNAs in plants. Based on the TAIR annotations, five mimic genes, AT1G55860 regulating ath-miR159a, AT2G29070 regulating ath-miR172, AT1G77870 modulating ath-miR775, and AT4G02950 and AT4G03360 modulating ath-miR862-3p, were implicated in post-translational protein modification through a ubiquitin-related pathway (Figure 3A and Additional file 3: Table S3).

### Uncovering the functions of the sequestered miRNAs through target identification

**Figure 4:**
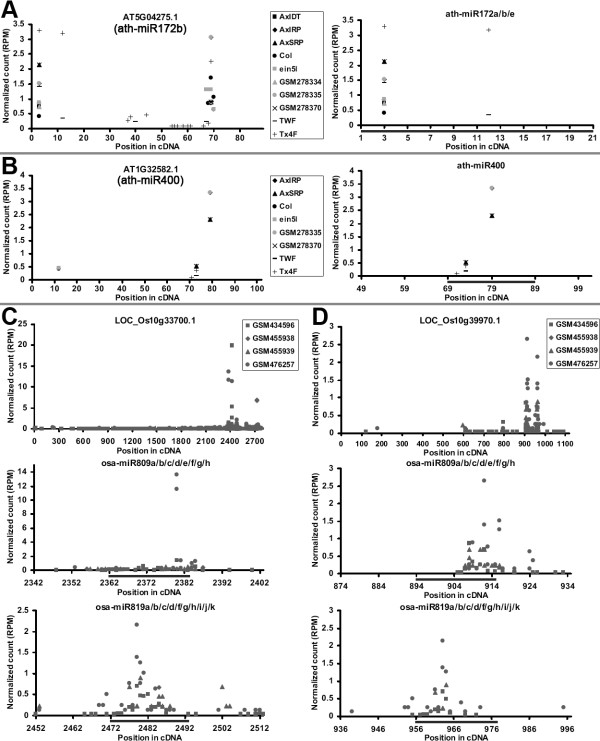
**Degradome sequencing data-based identification of the targets regulated by the sequestered microRNAs revealed the novel self-regulation and co-regulation mechanisms.** (**A**) Self-regulation of ath-miR172b. (**B**) Self-regulation of ath-miR400. (**C**) Co-regulation of the transcript LOC_Os10g33700.1 by osa-miR809 and osa-miR819. (**D**) Co-regulation of the transcript LOC_Os10g39970.1 by osa-miR809 and osa-miR819. For all the sub-figures [(**A**) to (**D**)], the first panels depict the degradome signals all along the target transcripts, and the other panels provide detailed views of the cleavage signals within the regions surrounding the target recognition sites (denoted by gray horizontal lines). The transcript IDs are shown in the first panels, and the miRNA IDs are listed in the other panels. The x axes measure the positions of the signals along the transcripts, and the y axes measure the signal intensities based on normalized counts (in RPM, reads per million), allowing cross-library comparison. See “Data sets used in this study” in the METHODS section for the degradome data sets used in this analysis.

Most plant miRNAs exert their regulatory roles in various biological processes through direct target cleavages [6]. To gain deeper functional insights of the miRNAs regulated by the target mimics, large-scale target prediction was performed by using miRU algorithm [21,22]. The predicted results were filtered by using degradome sequencing data-based approach in order to gain highly reliable miRNA—target regulatory relationships (Additional file 4: Figure S1 and Additional file 5: Figure S2). Among the identified miRNA—target pairs, some interesting regulations were observed. In Arabidopsis, the precursors of ath-miR172b and ath-miR400 could be recognized by their own mature miRNAs at the miRNA*-coding regions, and significant cleavage signals were observed in the middle of the target regions (Figure 4A and 4B). This observation further supports the “self-regulation” notion proposed previously [23,24]. In rice, both LOC_Os10g33700.1 and LOC_Os10g39970.1 were indicated to be co-regulated by osa-miR809 and osa-miR819 family members (Figure 4C and 4D). Additionally, alternative splicing employed by numerous genes may enable certain transcript variants to escape from miRNA-mediated regulation. For instance, the splicing variant AT5G63260.2 was found to be cleaved by ath-miR415, while AT5G63260.1 could not be targeted by this miRNA due to the lack of the target recognition site.

A large portion of miRNA targets have been demonstrated to be transcription factors in plants, suggesting their important role in gene regulatory cascades [2]. To gain a global functional view of these identified targets of the sequestered miRNAs, GO term enrichment analysis was performed again. As expected, “transcription factor activity” is a highly enriched function possessed by the target sets in both plants (Additional file 6: Figure S3A and S3B). Interestingly, the GO term “hydrolase activity, acting on acid anhydrides, in phosphorus-containing anhydrides” belonging to the “Molecular Function” category was found to be enriched in the rice target set (Additional file 6: Figure S3B). Considering the functional enrichment of the target mimic genes in phosphorus metabolism-related processes in both Arabidopsis and rice, the embedded implication is worth investigating. Moreover, according to the GO annotations, a large portion of the miRNA targets in Arabidopsis were suggested to be involved in the biological processes “RNA interference”, “cell differentiation”, “vegetative (leaf) and reproductive (flower, fruit, and seed) organ development”, and “meristem initiation” (Additional file 6: Figure S3C).

Also based on target prediction and degradome data-based validation, certain target mimics were identified to be regulated by specific miRNAs. For instance, the mimic transcripts AT1G69440.1 and AT5G03545.1 were indicated to be regulated by ath-miR5021 and ath-miR414 respectively (Additional file 7: Figure S4), and LOC_Os02g36880.3 was cleaved by osa-miR164a-f in rice (Additional file 8: Figure S5).

### Construction of the networks constituted by “miRNA—mimic—miRNA—target” regulatory cascades and subnetwork characterization

Through target mimic prediction and degradome data-based miRNA target identification, the basic data for establishing the “miRNA—mimic—miRNA—target” regulatory relationships were obtained. Thus, we set out to construct comprehensive networks involving target mimic—miRNA regulations in both Arabidopsis and rice by using Cytoscape [25]. At first glance, 465 nodes (including miRNAs, miRNA targets, and target mimics) were found to be connected by 559 edges in Arabidopsis, and 441 nodes connected by 1048 edges in rice (Additional file 9: Figure S6 and Additional file 10: Figure S7). To demonstrate the biological meanings of the constructed networks, certain subnetworks were further investigated.

**Figure 5:**
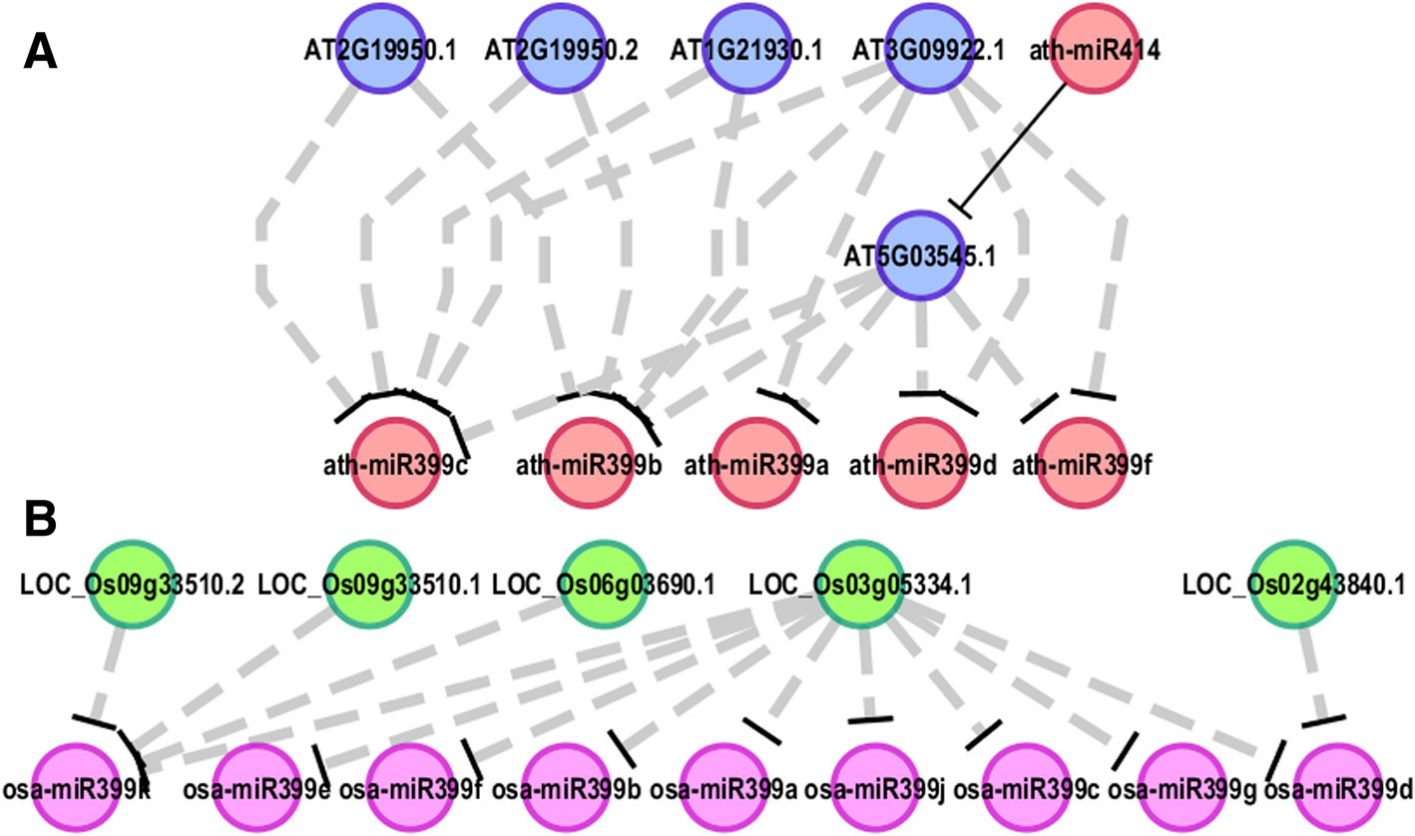
**miR399-involved subnetworks in Arabidopsis and rice.** (**A**) ath-miR399- and ath-miR414-involved phosphorous signaling-related subnetwork. (**B**) osa-miR399-involved phosphorous signaling-related subnetwork. The predicted regulatory relationships between target mimics and certain microRNAs were depicted by gray dashed lines, and the microRNA—target regulatory relationship was denoted by black line. All the networks were drawn by Cytoscape [25].

In Arabidopsis, the genes IPS1 (AT3G09922) and AT4 (AT5G03545) belonging to TPSI/Mt4 family were identified as the target mimics of ath-miR399 (Figure 5A and Additional file 3: Table S3), which was consistent with the previous experimental report [7]. It also indicates the high reliability of our criteria set for the prediction of target mimics. Based on the same prediction criteria, the IPS1 homologous gene LOC_Os03g05334 was identified in rice (Figure 5B and Additional file 11: Table S4). Besides, several novel genes (AT1G21930, AT2G19950, LOC_Os02g43840, LOC_Os06g03690, and LOC_Os09g33510) not belonging to the TPSI/Mt4 family were also indicated to have great potential to modulate the activities of miR399 in both plants. Whether these potential mimics are involved in phosphate signaling needs to be further addressed. One example is that miR399 was predicted to be under surveillance of the mimic gene LOC_Os02g43840 encoding an ethylene-responsive element-binding protein, suggesting a potential interplay between phosphorus and ethylene signaling pathways in rice. This scenario is supported by the recent finding that ethylene response factors are potentially implicated in the regulatory responses to phosphate deprivation in Arabidopsis [26]. More interestingly, within the ath-miR399-involved subnetwork, one mimic transcript encoded by AT5G03545 (annotated as AT4 belonging to the TPSI/Mt4 family) was found to be regulated by ath-miR414 through cleavage (Additional file 6: Figure S3). To our best knowledge, ath-miR414 has never been uncovered to participate in the phosphorus-related signaling pathways. Thus, the regulatory cascade ath-miR414—AT4—ath-miR399—PHO2 [the reported target PHO2 of miR399 [27] was not identified based on our degradome-based search, which might be attributed to the stringent search criteria that we used or the limited degradome sequencing data sets that available for this analysis] is worth further investigating.

**Figure 6:**
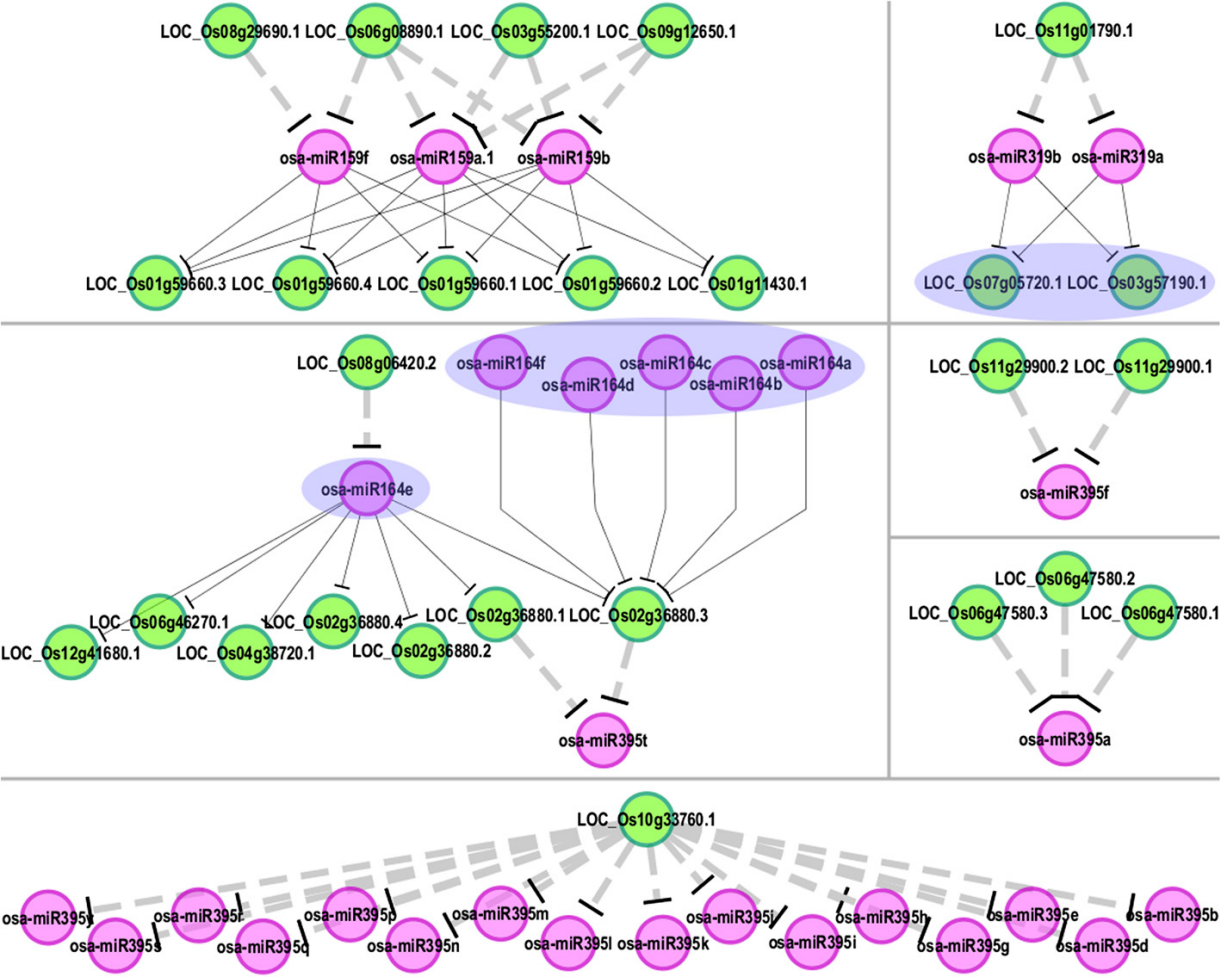
**osa-miR159-, osa-miR319-, osa-miR164-, and osa-miR395-involved subnetworks.** The predicted regulatory relationships between target mimics and certain microRNAs were depicted by gray dashed lines, and the microRNA—target regulatory relationships were denoted by black lines. The transcripts LOC_Os03g57190.1 and LOC_Os07g05720.1 belonging to TCP gene family, and the osa-miR164 family members were highlighted by translucent blue ovals, indicating that the expression of osa-miR164 was positively regulated by the TCP family genes. The network was drawn by Cytoscape [25].

Another novel finding was exploited from the miR159/319-, miR164- and miR395-involved subnetwork in rice (Figure 6). osa-miR164 was demonstrated to cleave the transcript LOC_Os02g36880.3 [belonging to NAC family, a well-characterized target gene family regulated by miR164 in plants [2]], which in turn regulated osa-miR395t through target mimicry. Since miR164 and miR395 were reported to be involved in auxin signaling [28] and sulfate metabolism [29,30] respectively, the possibility that the auxin—sulfur signal interplay could be mediated by the cascade miR164—NAC—miR395 needs in-depth investigations. However, we observed that only osa-miR395t was predicted to be regulated by the mimic transcript LOC_Os02g36880.3, and no downstream target was identified for this miRNA based on degradome sequencing data. Thus, whether the regulatory relationship, LOC_Os02g36880.3—osa-miR395t specifically exists in rice needs to be interrogated carefully. But, we suggest that the “target mimicry” relationship between NAC family gene transcript and miR395 identified in this subnetwork may not be a false positive. Another NAC—miR395 relationship was identified between LOC_Os10g33760.1 and most of the miR395 family members in rice (Figure 6 and Additional file 11: Table S4). More complicatedly, the target genes of osa-miR319, LOC_Os03g57190 and LOC_Os07g05720, encode transcription factors belonging to TCP family, which was demonstrated to positively regulate the expression of miR164 at the transcriptional level in Arabidopsis [31]. Thus, the regulatory cascade TCP—miR164—NAC—miR395 may be at the nexus of the auxin and the sulfur signaling pathways. In Arabidopsis, a largely conserved subnetwork involving miR159/319, miR164 and miR395 was also extracted from the comprehensive network (Additional file 12: Figure S8). However, no NAC-related gene was discovered as a potential target mimic of miR395. Thus, whether the NAC—miR395-mediated auxin—sulfur signal interaction is specifically existed in rice needs further interpretation. Another interesting finding is that different from rice, in which miR159 and miR319 regulate distinct sets of target genes separately (MYB and TCP genes, respectively), miR159 and miR319 in Arabidopsis share an overlapping set of targets. Previous study indicates that although the sequences of miR159 and miR319 show high identity with each other, these two miRNA species possess specialized functions by regulating distinct sets of targets, i.e. MYB and TCP genes, separately [32]. Our observation in Arabidopsis indicated the partial functional redundancy between the two homologous miRNA families. Additionally, the mimic gene of ath-miR164, AT4G03280, is suggested to play a role in photosynthetic electron transfer based on TAIR annotation. On the other hand, the involvement of miR164 in plant photosynthesis is supported by several pieces of recent evidences [33-35]. Notably, based on the previous study, the expression of AT1G60710, a potential mimic of ath-miR395a/day/e, showed specific response to sulfur depletion treatment [36]. Thus, the target mimicry relationship between AT1G60710 and ath-miR395 could add a potential layer of miR395-mediated sulfur signaling pathways.

Several other interesting subnetworks were also extracted for characterization. For instances, only one member of miR156 families in both plants, i.e. ath-miR156h and osa-miR156k, was identified within the established comprehensive networks (Additional file 13: Figure S9). Both mimics and downstream targets were discovered for the two miR156 genes. Thus, whether these two miR156 family members play a dominant role in specific regulatory pathway in both plants requires further investigations. Based on our computational approach, the target genes of miR172 belonging to the AP2 family were identified based on the significant cleavage signals within the target sites in Arabidopsis (Additional file 4: Figure S1 and Additional file 13: Figure S9). Thus, in addition to the previously reported translational repressive effect of miR172 on the AP2 genes [37,38], target cleavages of the AP2 transcripts may also play an indispensable role in floral organ development. Within the ath-miR169-mediated subnetwork, the miRNA star species, ath-miR169g*, was found to be potentially regulated by three mimic transcripts, AT1G52060.1, AT4G16070.1 and AT4G16070.2 (Additional file 13: Figure S9). Considering the widespread regulatory activities of miRNA*s unraveled in recent years [39-42], it is reasonable that the active levels of certain miRNA*s should be under strict surveillance through target mimicry. In rice, a similar miR169-invovel subnetwork was identified, although no such mimic—miR169* regulatory relationship existed (Additional file 13: Figure S9). However, based on the TIGR rice annotations, two mimic genes of osa-miR169, LOC_Os05g24010 and LOC_Os09g37800, were suggested to be responsive to stress. Considering the reported involvement of miR169 in drought [43] and nitrogen starvation [44] response in rice, the characterized subnetwork may play an essential role in multi-stress-induced response. Moreover, the largest subnetwork involving miR446, miR809 and miR819 was identified in rice (Additional file 13: Figure S9). These three miRNA families seem to be rice-specific according to the current miRBase registries (release 17), and their biological functions have not been characterized. Unfortunately, we could not gain any informative hints from the current annotations of the mimic and target genes within this subnetwork. Hence, it will be interesting to gain functional insights from the established subnetwork through experimental approaches.

## Conclusions

Taken together, comprehensive networks constituted by numerous target mimic—miRNA—target regulatory cascades have been constructed in Arabidopsis and rice through in silico target mimic site prediction and degradome data-based target identification. By in-depth characterization of certain interesting subnetworks, the established networks were demonstrated to be relatively reliable and biologically meaningful. Several subnetworks were observed to be conserved between Arabidopsis and rice to some extent (Figure 5, Figure 6, Additional file 12: Figure S8 and Additional file 13: Figure S9). And some might be species-specific. We hope this study could expand the current view of miRNA-mediated regulatory networks in plants, and will inspire more research efforts on the novel regulatory mechanisms for modulating miRNA activities, such as the target mimicry characterized in our analysis.

## Methods

### Data sets used in this study

The degradome sequencing data sets were retrieved from GEO (Gene Expression Omnibus; http://www.ncbi.nlm.nih.gov/geo/) [45] and NGSDBs (Next-Gen Sequence Databases; http://mpss.udel.edu/) [46]. The accession numbers of these data sets are: (1) Arabidopsis degradome data: GSM278333, GSM278334, GSM278335, and GSM278370 from GEO; and AxIDT, AxIRP, AxSRP, Col, ein5l, TWF, and Tx4F from NGSDBs. (2) rice degradome data: GSM434596, GSM455938, GSM455939, and GSM476257 from GEO. The gene annotation and sequence information of Arabidopsis and rice were retrieved from the FTP sites of TAIR (release 10; ftp://ftp.arabidopsis.org/home/tair/Sequences/blast_datasets/) [11] and TIGR rice (release 6.1; ftp://ftp.plantbiology.msu.edu/pub/data/Eukaryotic_Projects/o_sativa/annotation_dbs/pseudomolecules/) [12], respectively. The sequences of the miRNA precursors and the mature miRNAs were downloaded from miRBase (release 17; http://www.mirbase.org/) [10]. The other non-protein-coding RNA sequences were retrieved from Genomic tRNA Database (http://gtrnadb.ucsc.edu/download.html) [15] and NONCODE (http://www.noncode.org/NONCODERv3/download.htm) [16,17].

### Prediction of endogenous target mimics in Arabidopsis and rice

First, Ssearch from the FASTA3 program package [downloaded from the FTP site of EBI (European Bioinformatics Institute), ftp://ftp.ebi.ac.uk/pub/software/unix/fasta/[13,14] was used to search for the sites in cDNA sequences that were reverse complementary to the miRNAs. Each miRNA of Arabidopsis and rice was included to search against the cDNA sequence library of the corresponding plant species. The cDNA sequences were retrieved from TAIR and TIGR rice, and the miRNAs from miRBase as mentioned above. To retain the predicted site with low complementary to the miRNAs (to allow the identification of target mimics with big bulges within the complementary sites), the first 5,000 Ssearch results for each miRNA were obtained for further identification. To discover the miRNA mimics, we applied the following set of rules referring to the previous experimental results [7-9]: (1) The 3- to 5-nt bulges must exist within the complementary sites of the cDNAs, and the bulges should located in the middle of the corresponding miRNAs (definition of the middle positions: 9^th^ to 11^th^ nt of the 19-nt-long miRNAs; 10^th^ to 11^th^ nt of the 20-nt miRNAs; 10^th^ to 12^th^ nt of the 21-nt ones; 11^th^ to 12^th^ nt of the 22-nt ones; 11^th^ to 13^th^ nt of the 23-nt ones; 12^th^ to 13^th^ nt of the 24-nt ones). (2) For the total mismatches within the non-middle region of each miRNA, no more than 4 were allowed, and the consecutive mismatches should not exceed 2 nt. (3) No bulge was permitted within the non-middle regions of the miRNAs. A Perl script was developed to perform this rule-based screening. The cDNAs satisfied the above criteria were considered to be target mimic candidates.

### Distribution pattern analysis of the target mimic sites on the gene transcripts

Based on the TAIR and TIGR rice annotations, only the target mimic sites located on the gene transcripts with 5’ UTR—CDS—3’ UTR structure were included in this analysis. In some cases, one mimic site might be recognized by two or more different miRNAs (especially for the members of the same miRNA families). These sites were considered only once.

### Prediction and degradome-based validation of miRNA targets

Target prediction was performed by using miRU algorithm [21,22] with default parameters. The degradome sequencing data were utilized to validate the predicted miRNA—target pairs. First, the read counts of all the degradome reads from each library were normalized in order to allow cross-library comparison. The normalized read count (in RPM, reads per million) of a short read from a specific library was calculated by dividing the raw count of this read by the total counts of the library, and then multiplied by 10^6^. Second, all the degradome short reads were mapped to the predicted target transcripts by using BLAST algorithm [47], and only the perfectly matched reads were retained. Then, two-step filtering was performed to extract the most likely miRNA—target pairs. During the first step, the predicted targets were retained for further validation only if there were three or more degradome reads with identical 5’ ends located within the predicted target binding sites. For this filtering step, all the degradome data sets were utilized at the same time to do a comprehensive screening. It was based on the scenario that a miRNA—target pair was considered to be the candidate once the cleavage signal(s) existed in any data set(s). After the first filtering, the degradome signals along each retained transcript were obtained from the BLAST results to provide a global view of the signal noise when compared to the signal intensity within a specific target binding site. Referring to our previous study [48], both the global and the local t-plots (target plots) [49,50] were drawn. Finally, exhaustive manual filtering was performed, and only the transcripts with cleavage signals easy to be recognized were extracted as the miRNA—target pairs.

## Abbreviations

miRNA, MicroRNA; IPS1, Induced by Phosphate Starvation 1; UTR, Untranslated region; CDS, Coding sequence; GO, Gene Ontology; sRNA, Small RNA; TAIR, The Arabidopsis Information Resource; TIGR, The Institute for Genome Research; U, Uridine; A, Adenine; C, Cytosine; TE, Transposable element; GEO, Gene Expression Omnibus; NGSDBs, Next-Gen Sequence Databases; RPM, Reads per million; t-plots, Target plots.

## Competing interests

The authors declare that they have no competing interest.

## Authors’ contributions

Conceived and designed the experiments: YM, HW, YJ. Performed the experiments: CS. Analyzed the data: YM CS. Contributed reagents/materials/analysis tools: YM CS. Wrote the paper: YM, HW, YJ. All authors read and approved the final manuscript.

## Supplementary Material

Additional file 1 **Table S1.** Prediction results of the target mimics of the Arabidopsis microRNAs. Click here for file

Additional file 2 **Table S2.** Prediction results of the target mimics of the rice microRNAs. Click here for file

Additional file 3 **Table S3.** Annotation information of the target mimics in Arabidopsis. Click here for file

Additional file 4 **Figure S1.** Degradome sequencing data-based identification of the targets of sequestered microRNAs in Arabidopsis. Click here for file

Additional file 5 **Figure S2.** Degradome sequencing data-based identification of the targets of sequestered microRNAs in rice. Click here for file

Additional file 6 **Figure S3**. GO (Gene Ontology) term enrichment analysis of the targets of sequestered microRNAs in Arabidopsis and rice. Click here for file

Additional file 7 **Figure S4.** Degradome sequencing data-based identification of the microRNAs regulating the target mimics in Arabidopsis. Click here for file

Additional file 8 **Figure S5.** Degradome sequencing data-based identification of the microRNAs regulating the target mimics in rice. Click here for file

Additional file 9 **Figure S6.** The whole network constructed in Arabidopsis. Click here for file

Additional file 10 **Figure S7.** The whole network constructed in rice. Click here for file

Additional file 11 **Table S4.** Annotation information of the target mimics in rice. Click here for file

Additional file 12 **Figure S8.** Ath-miR159/319-, ath-miR164-, and ath-miR395-involved subnetworks. Click here for file

Additional file 13 **Figure S9.** Certain subnetworks in Arabidopsis and rice. Click here for file
