# Supplementary figures and images for: Target mimics: an embedded layer of microRNA-involved gene regulatory networks in plants
Source: BMC Genomics. 2012 May 21;13:197. doi: 10.1186/1471-2164-13-197 (PMC3441763; doi:10.1186/1471-2164-13-197)

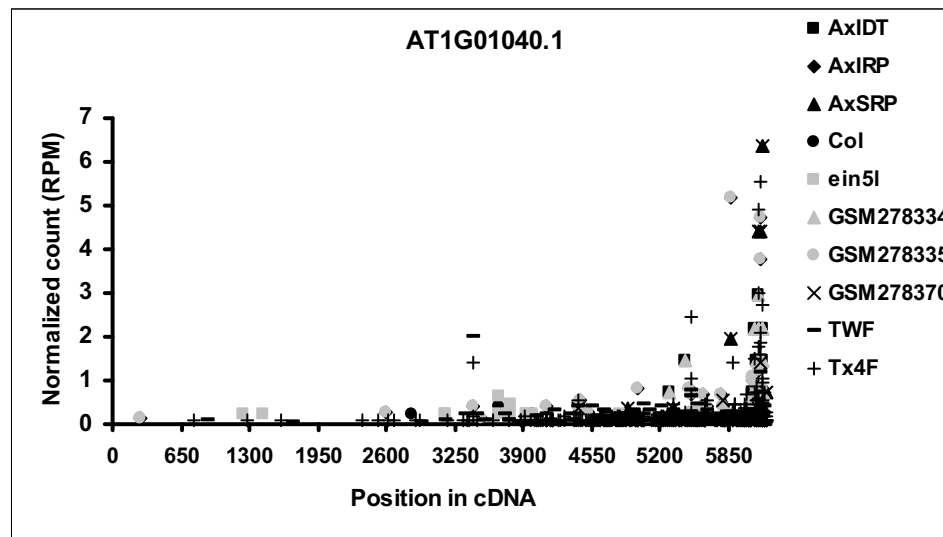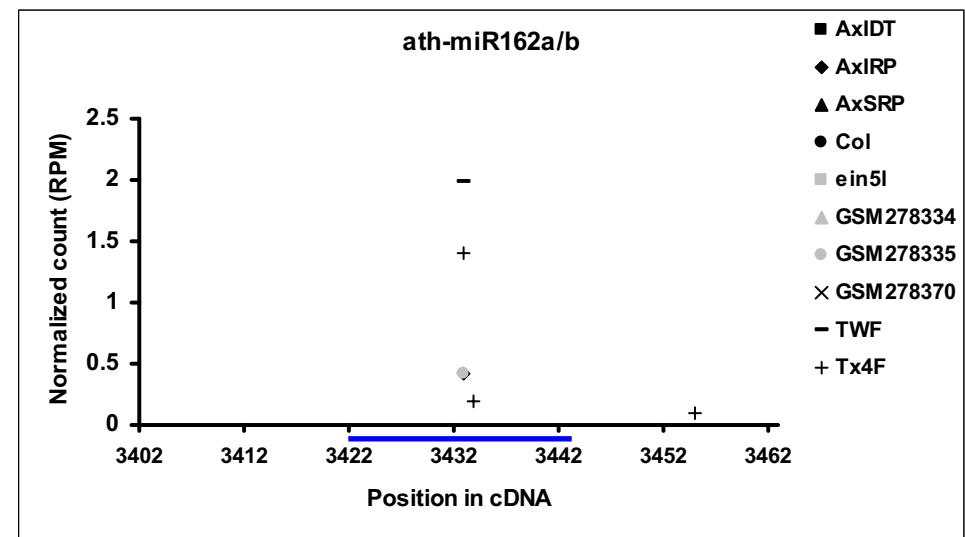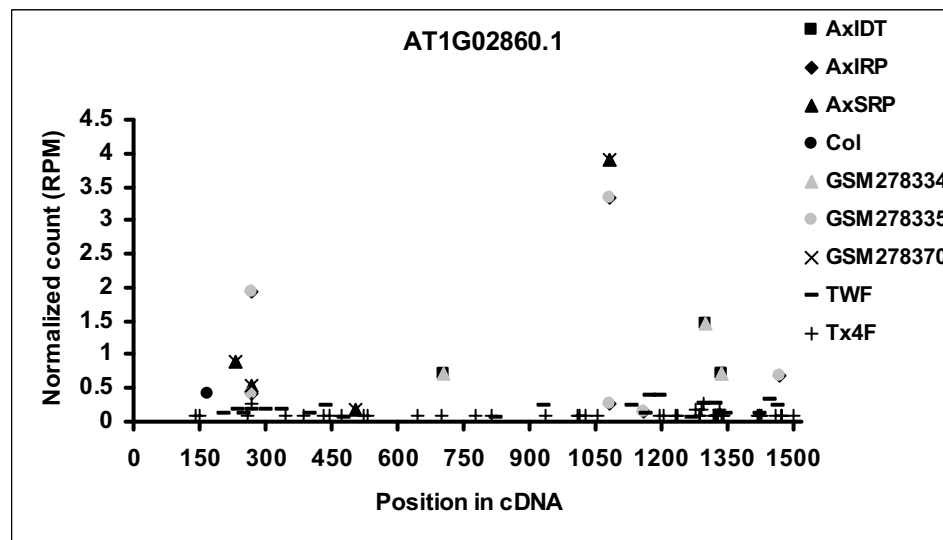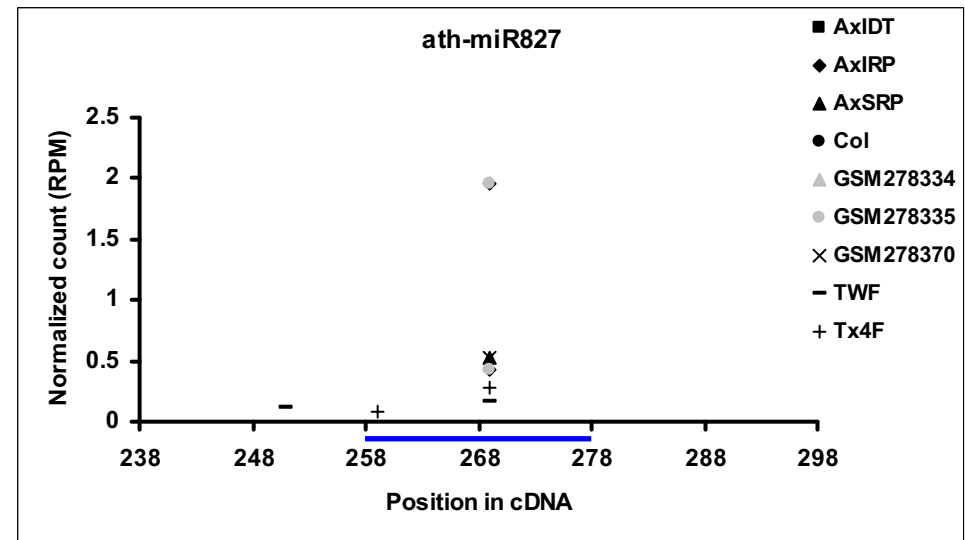

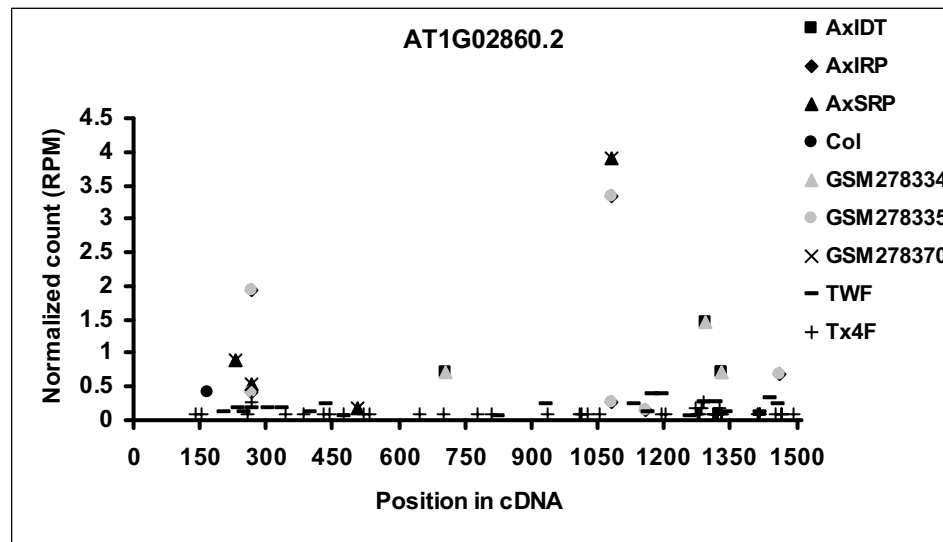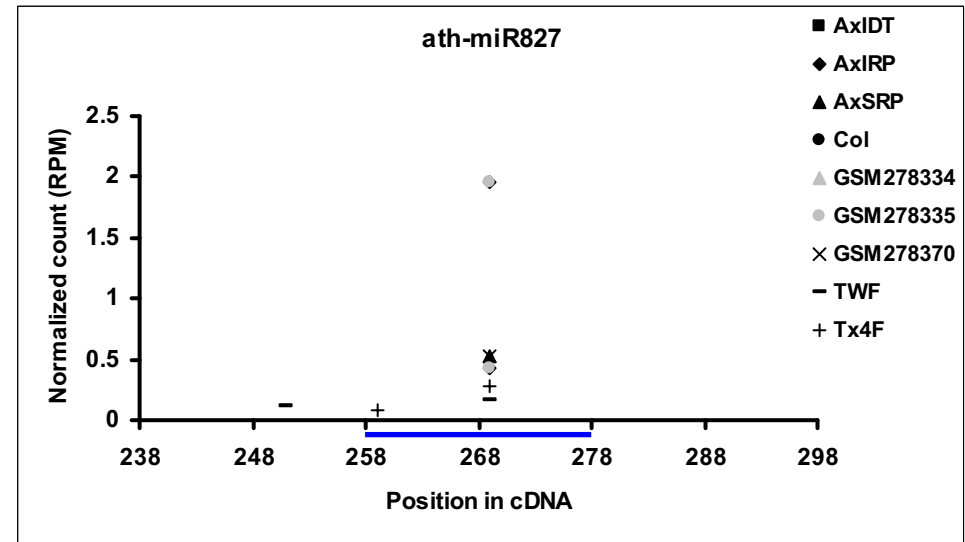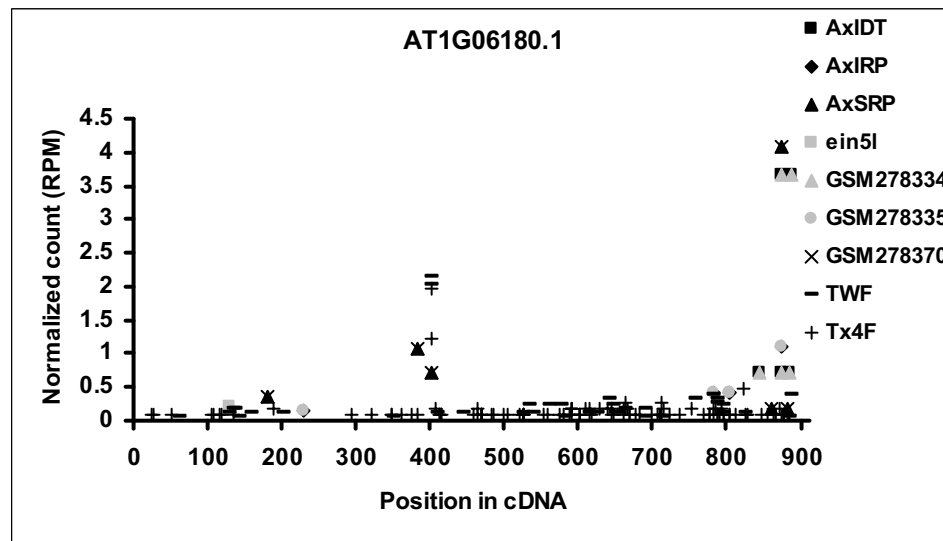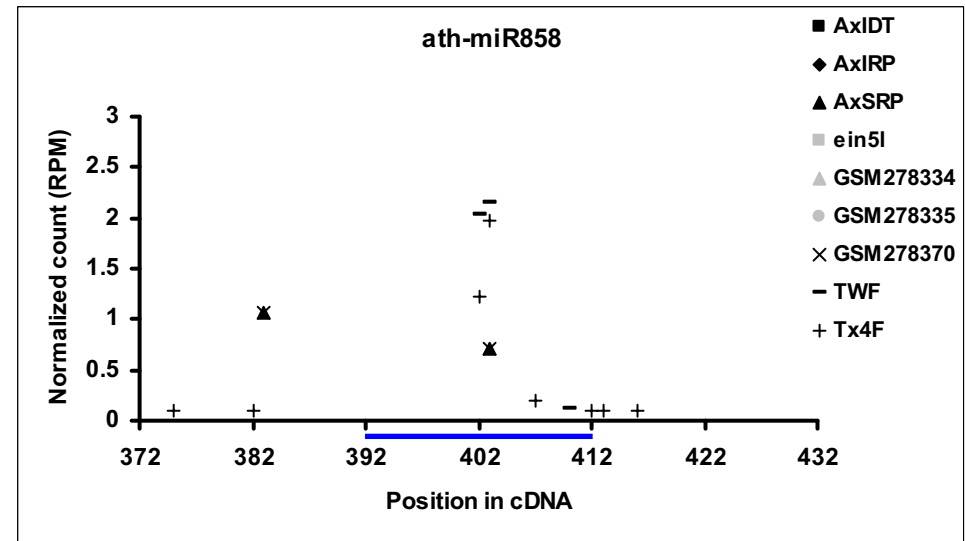

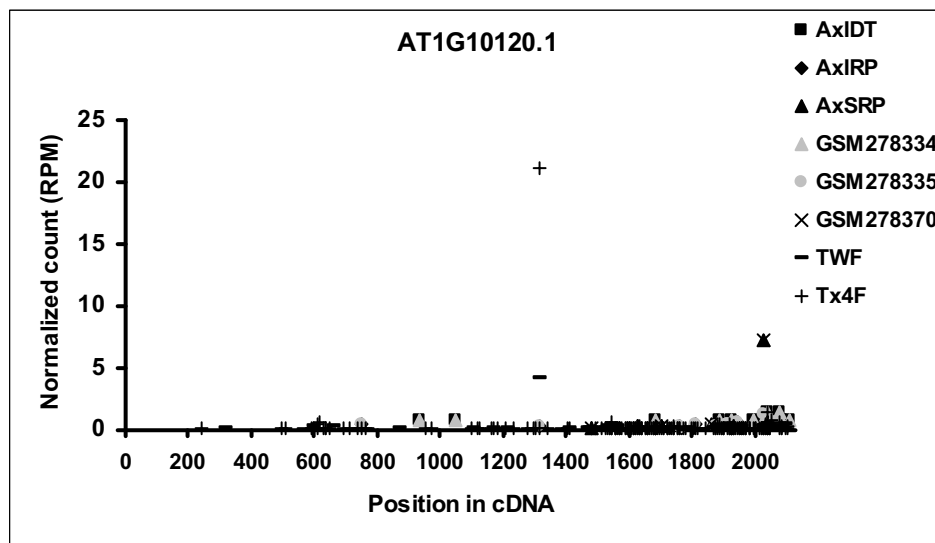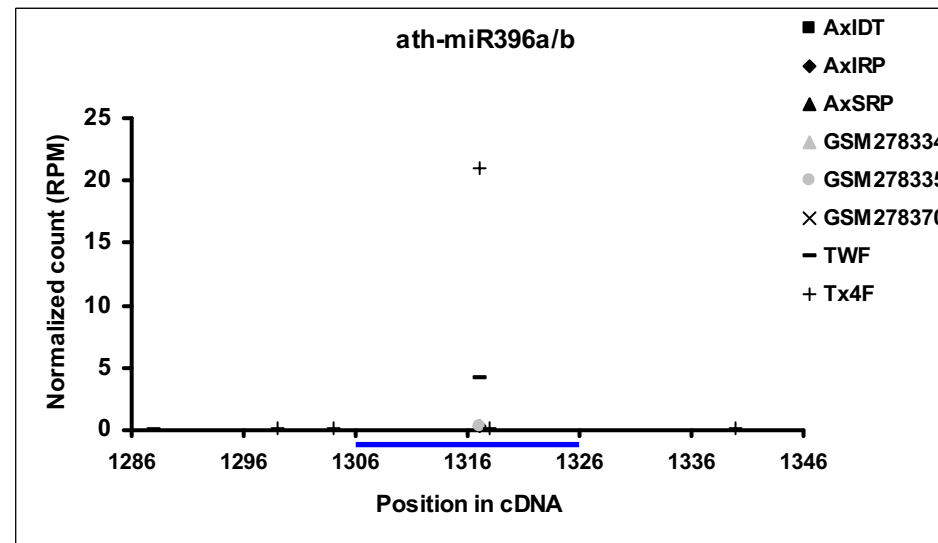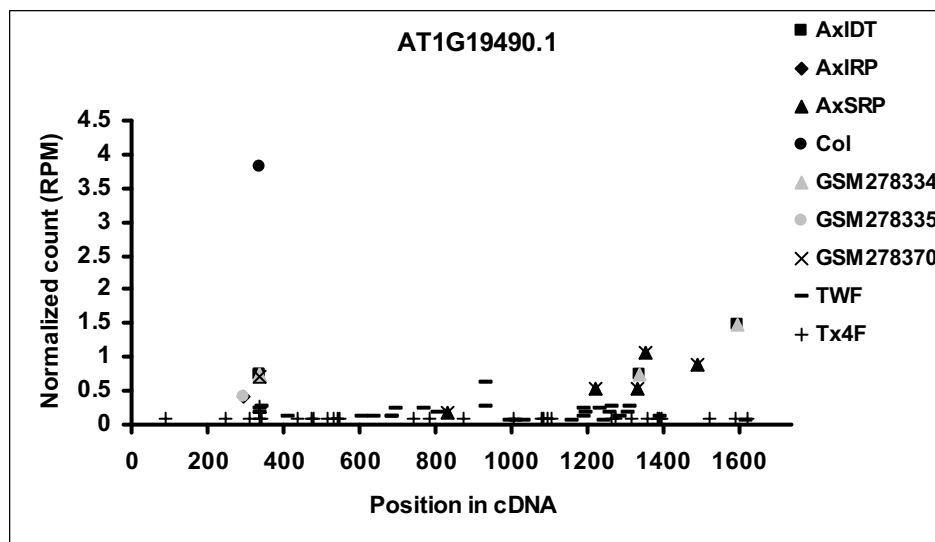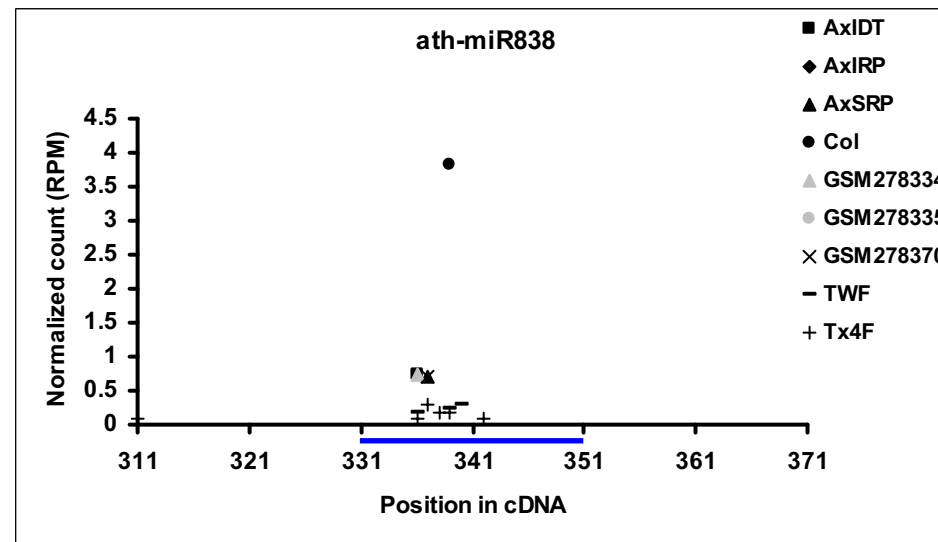

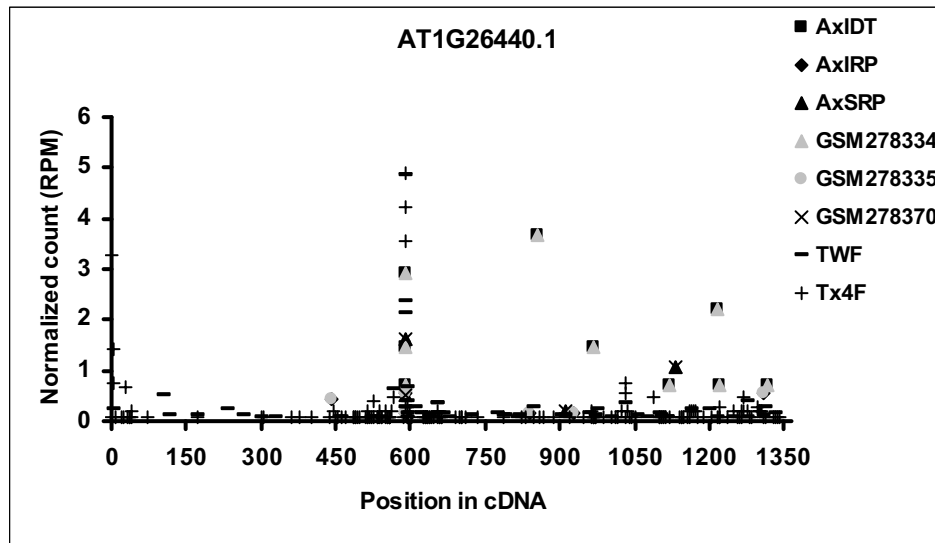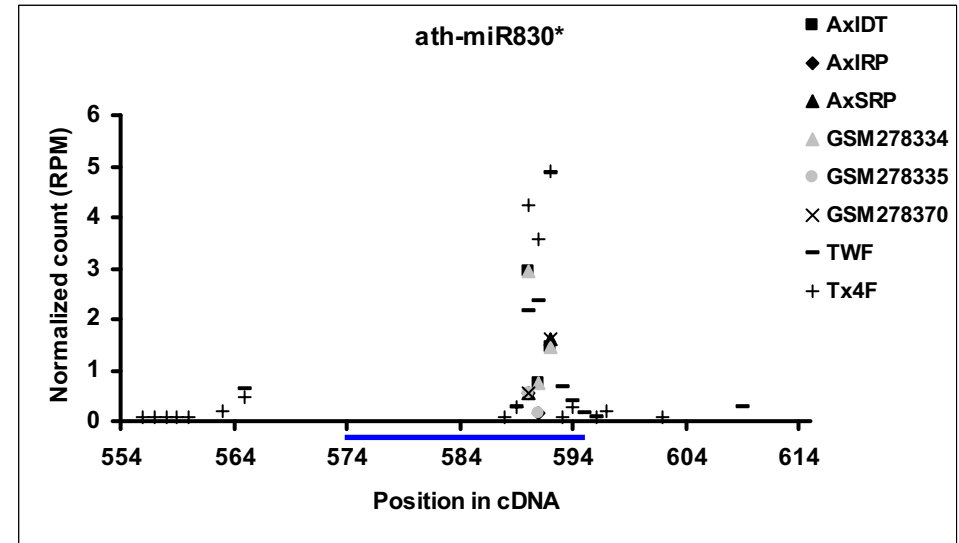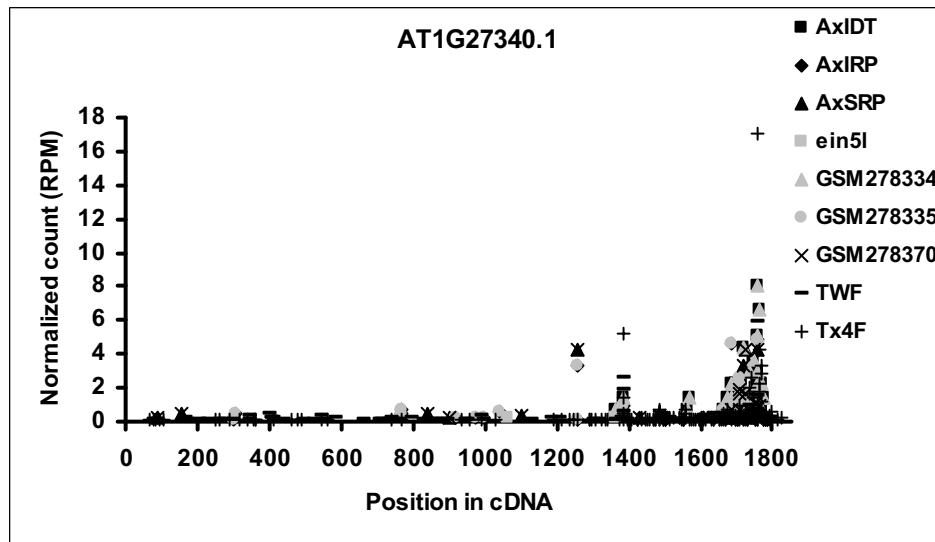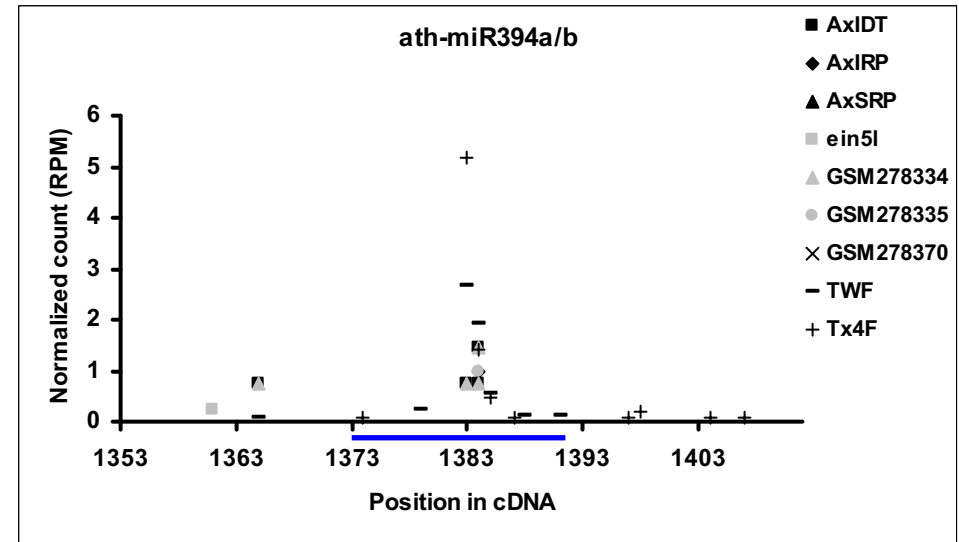

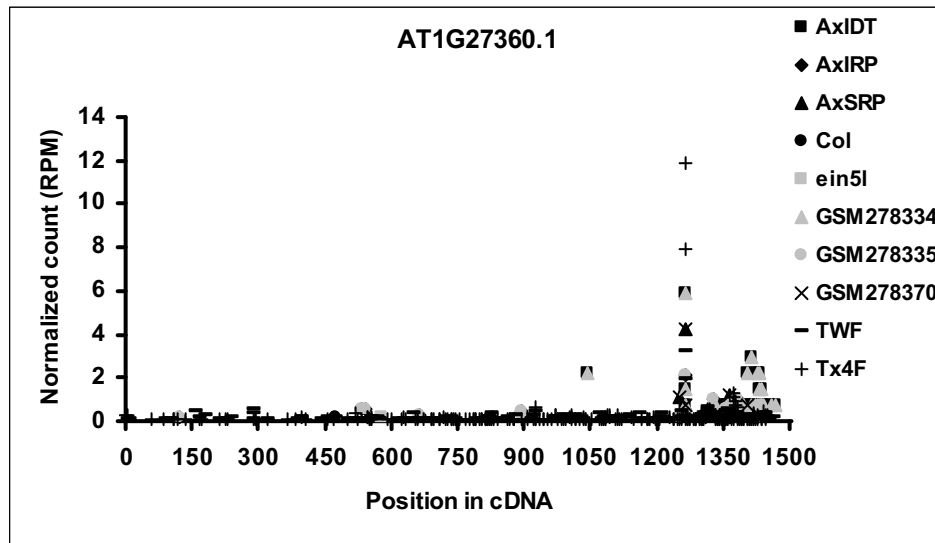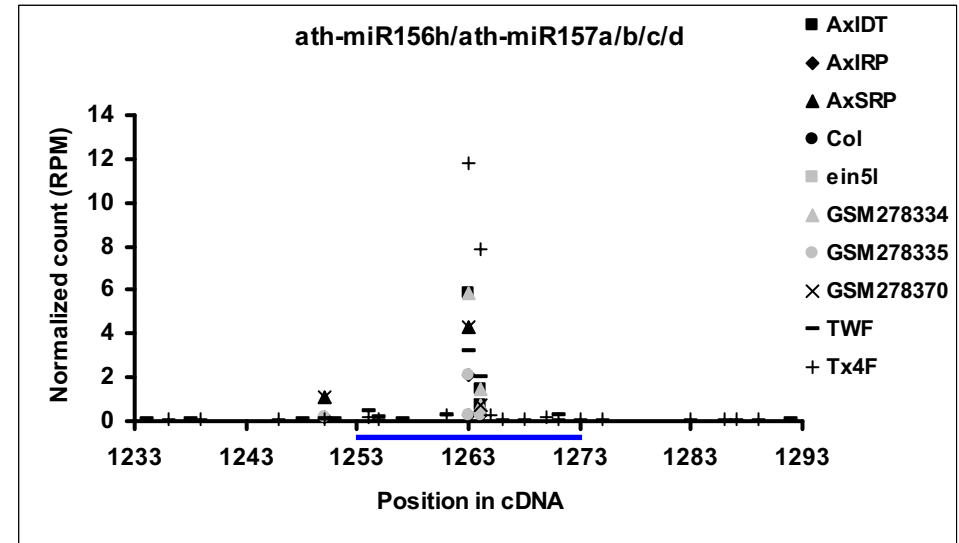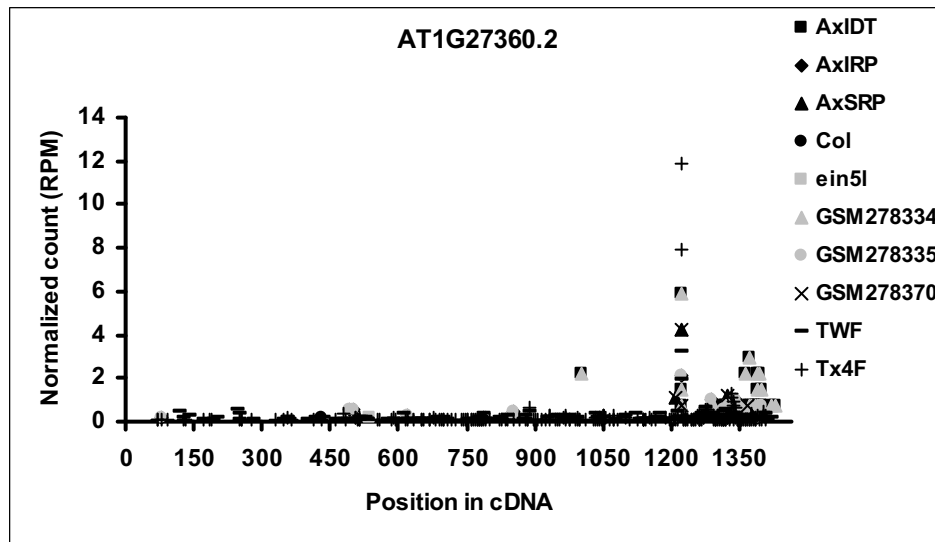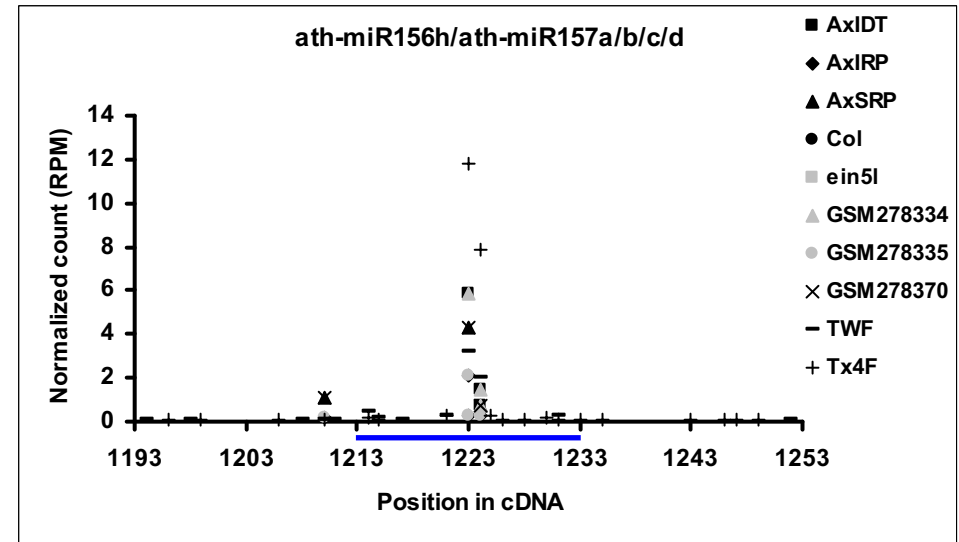

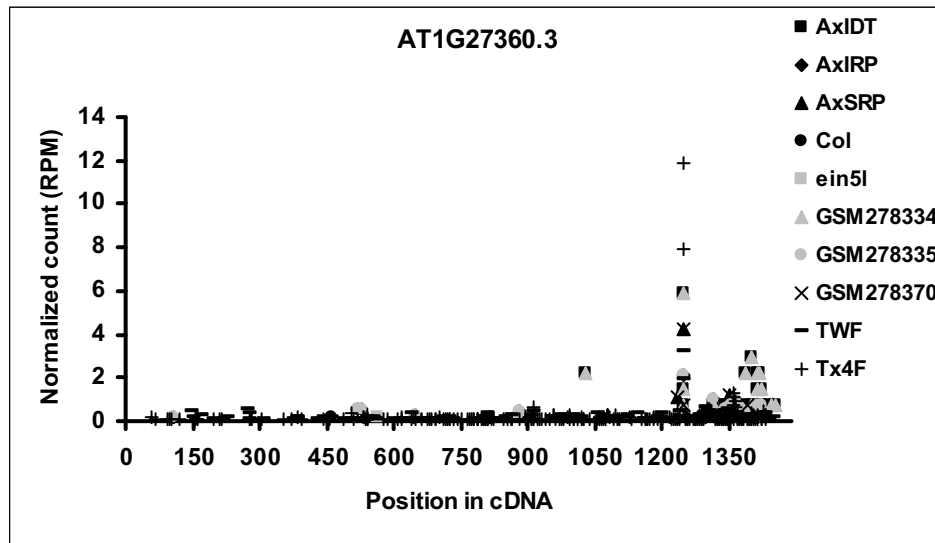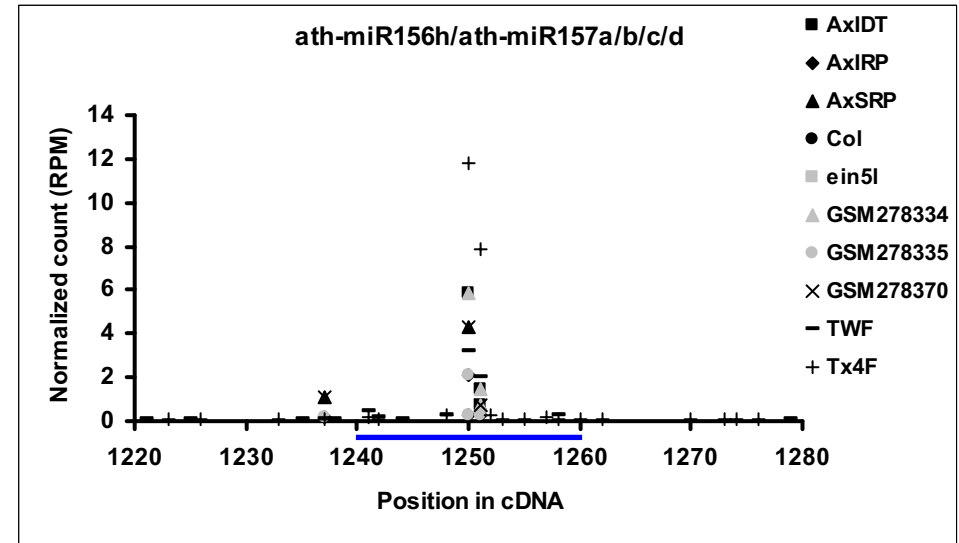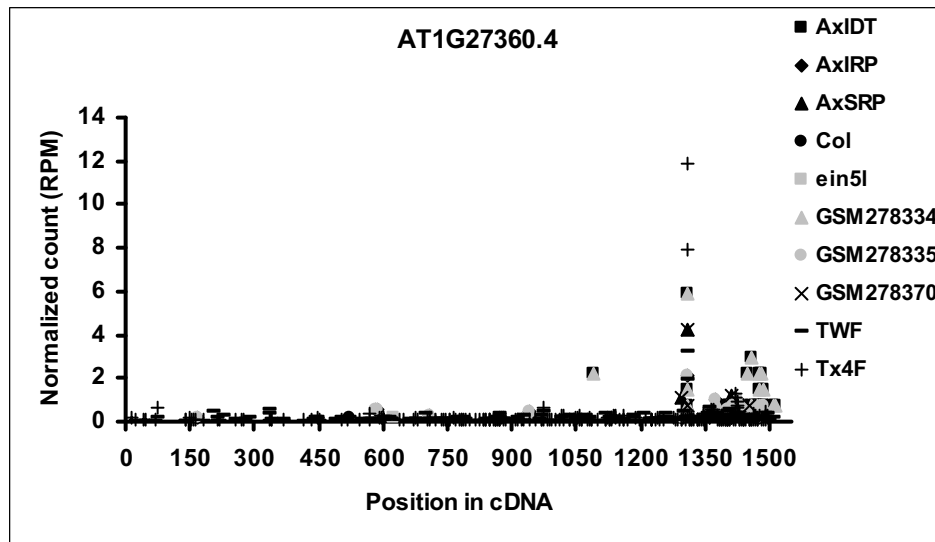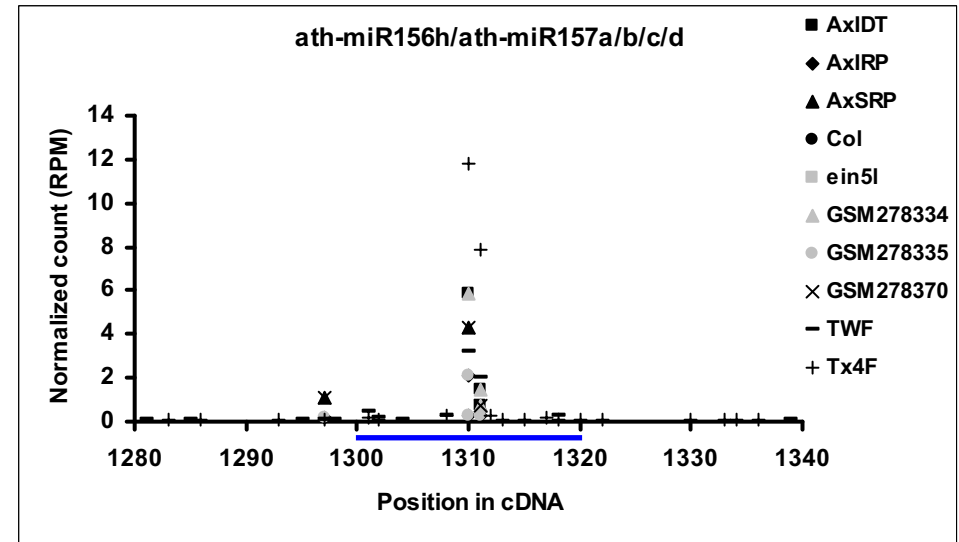

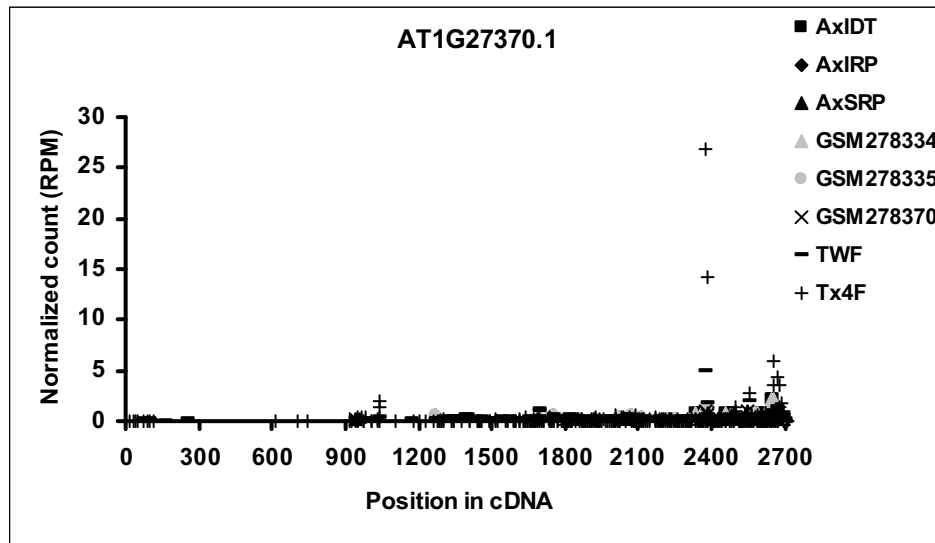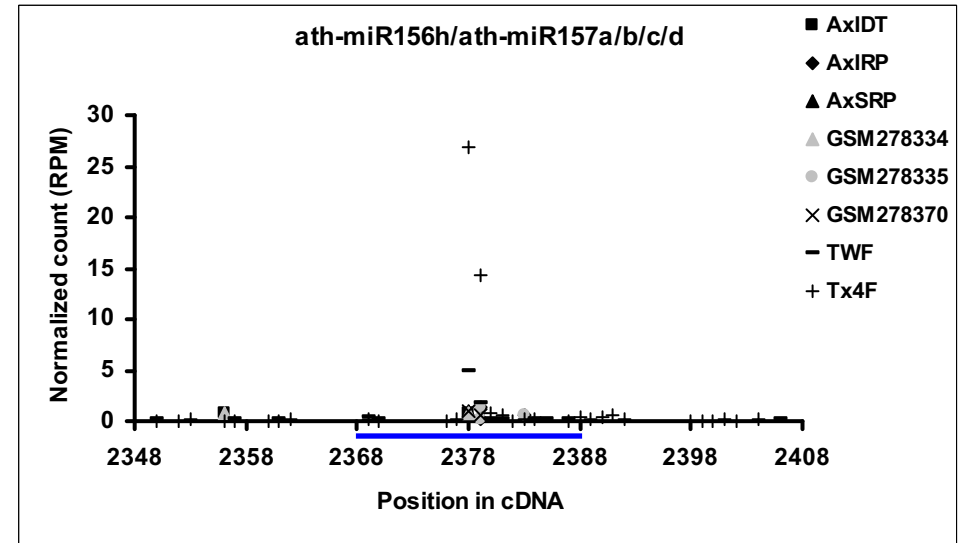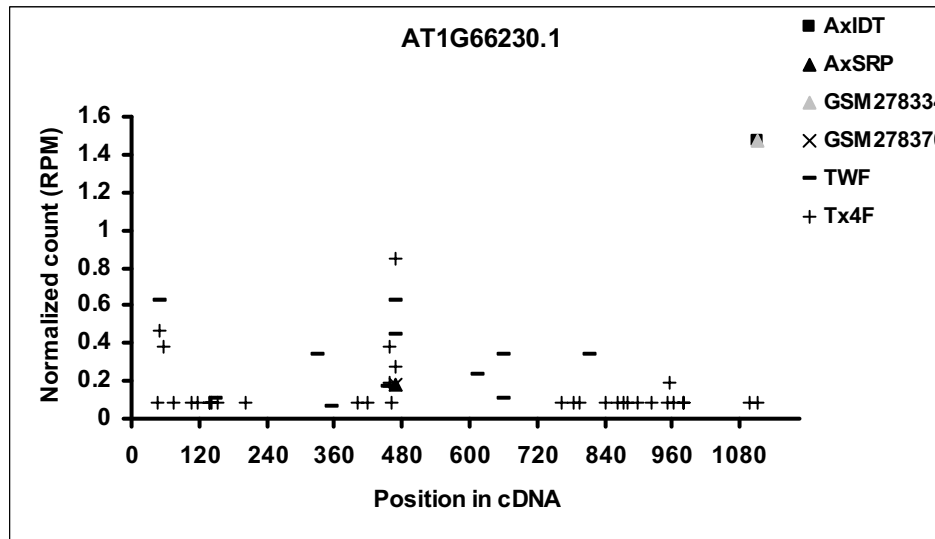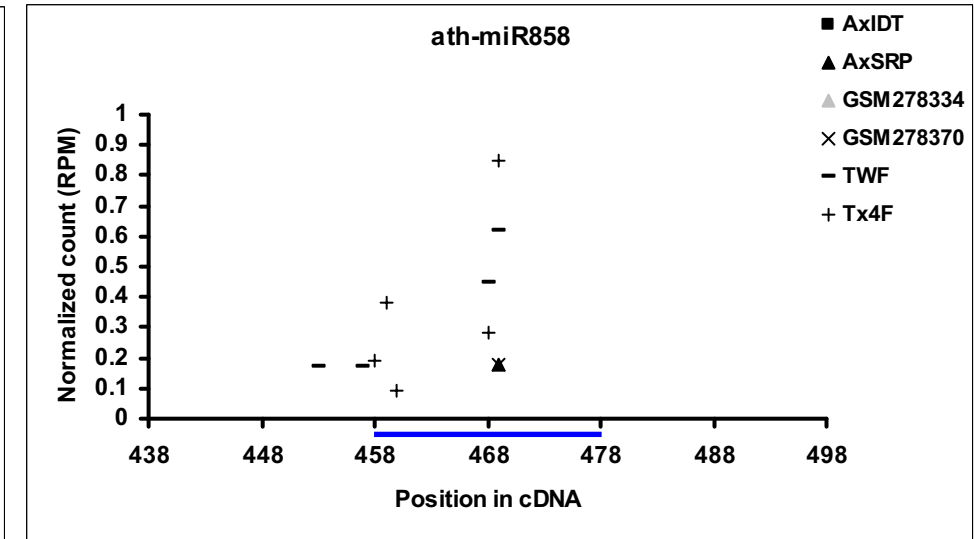

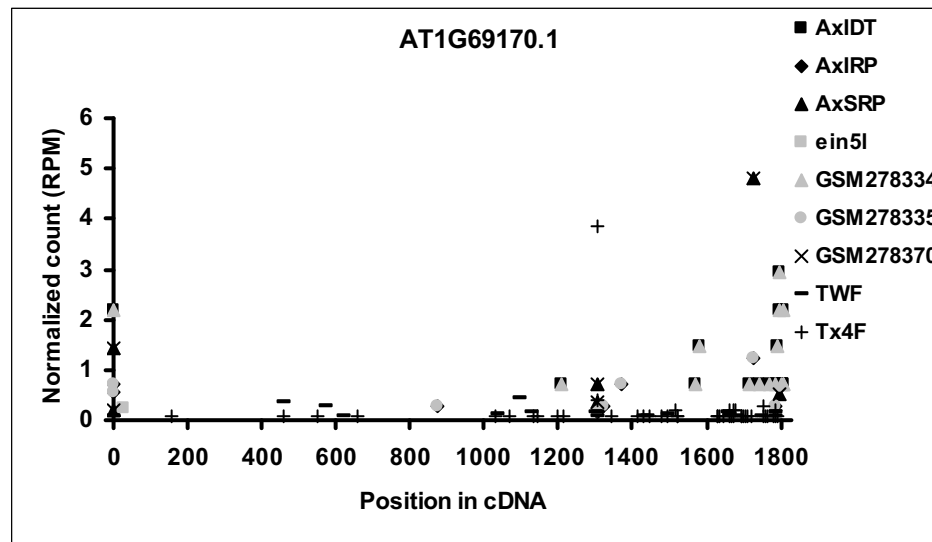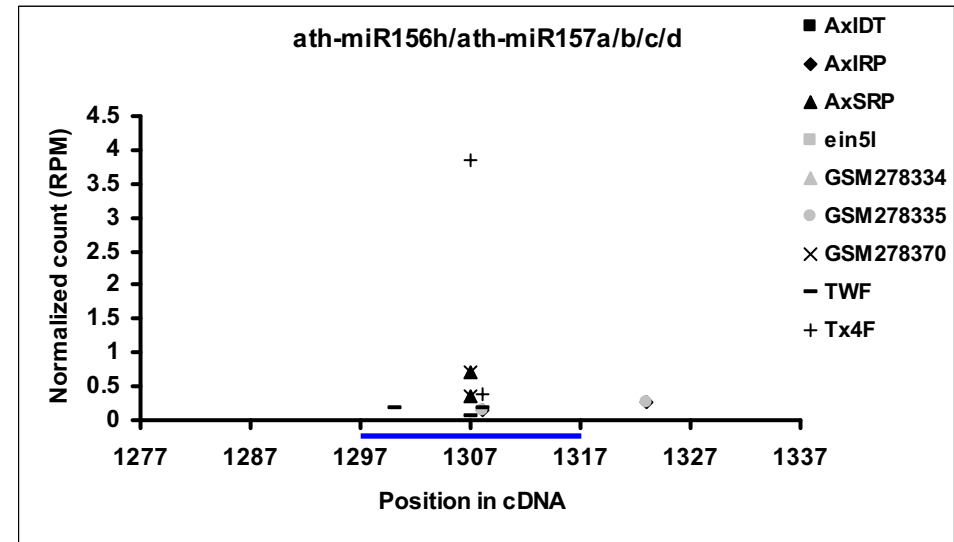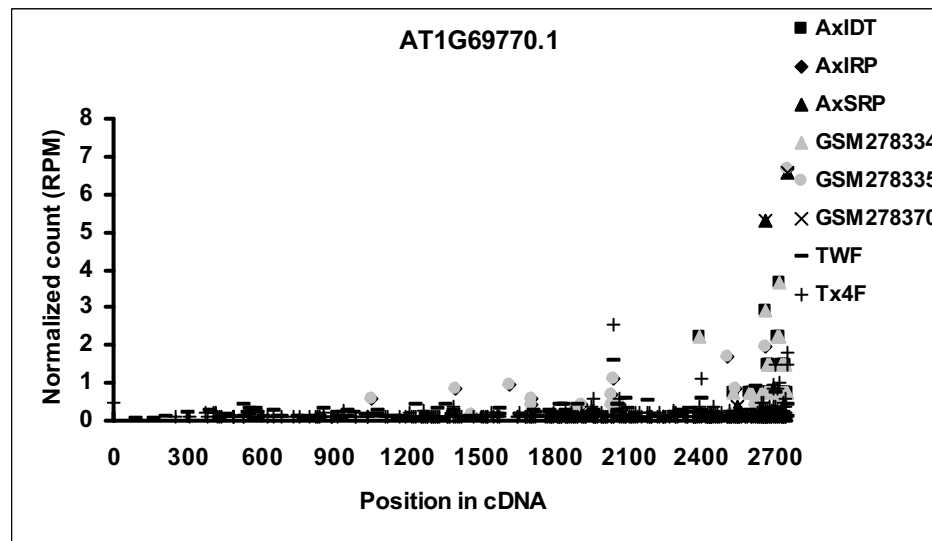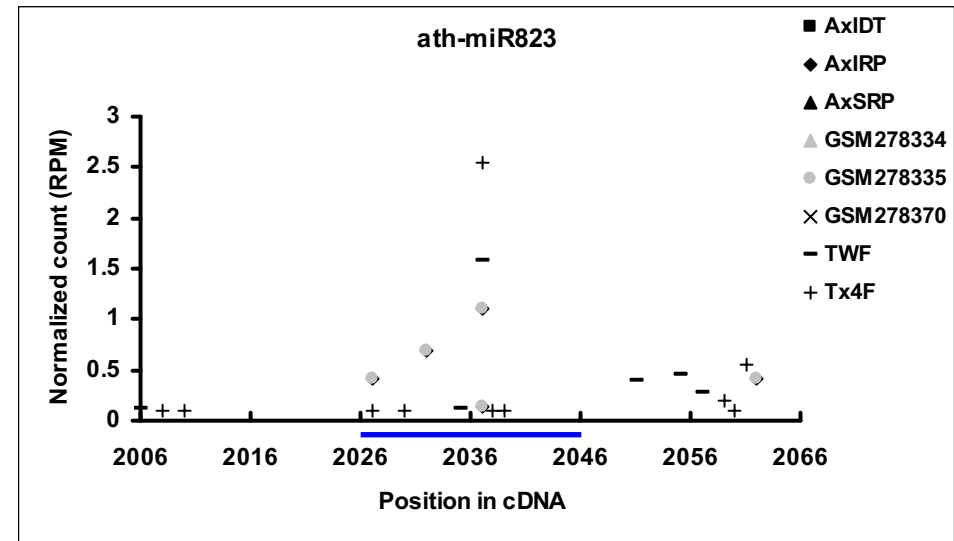

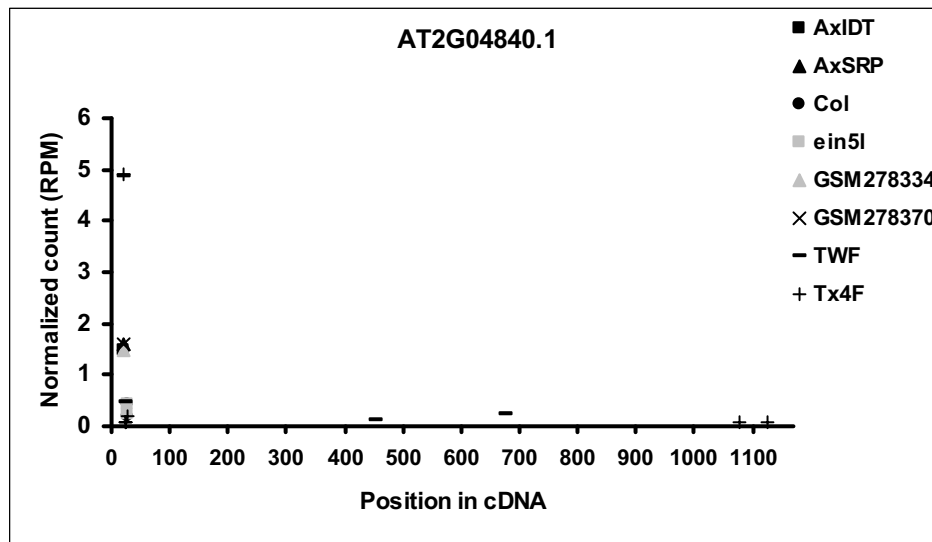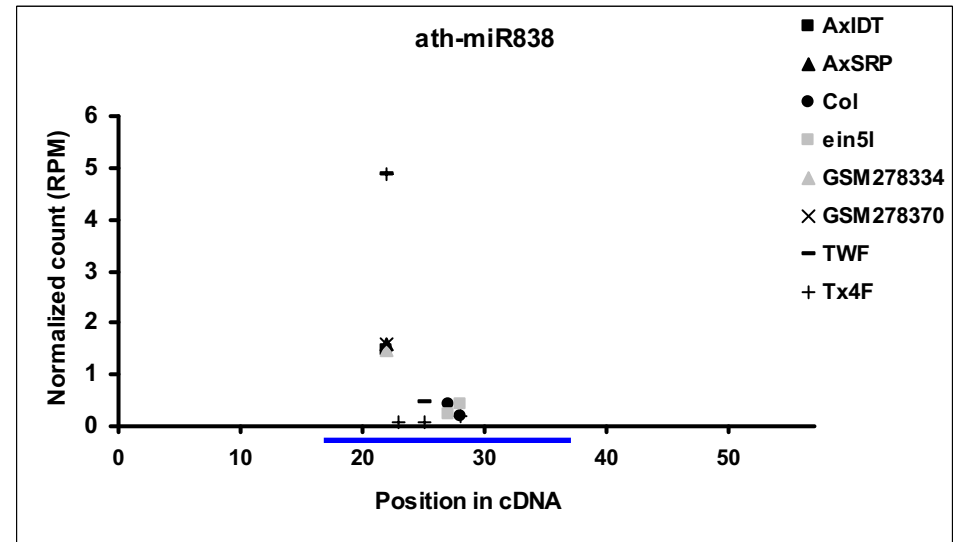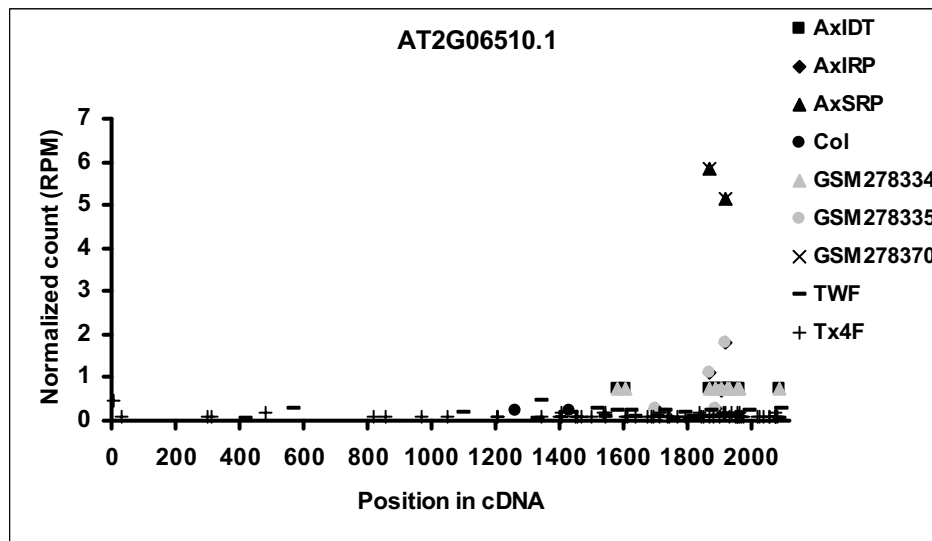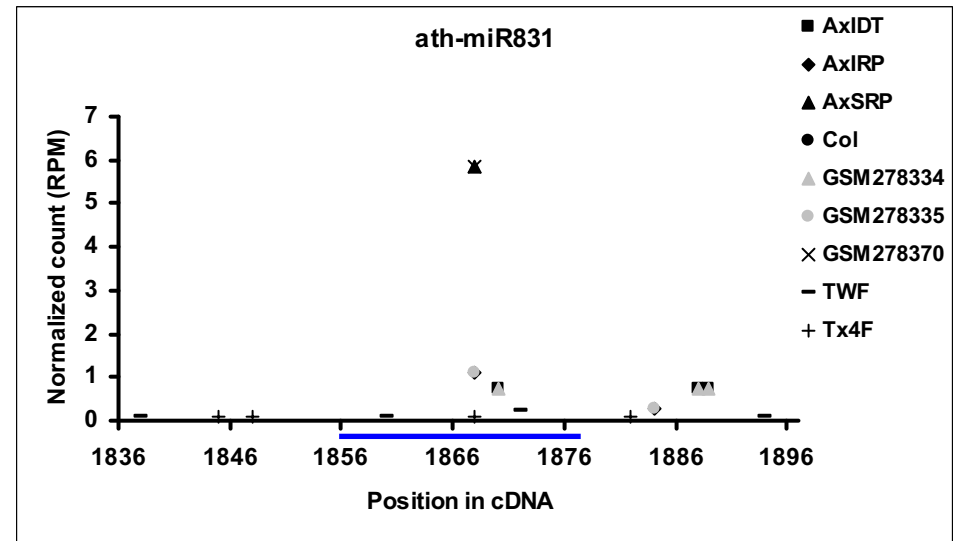

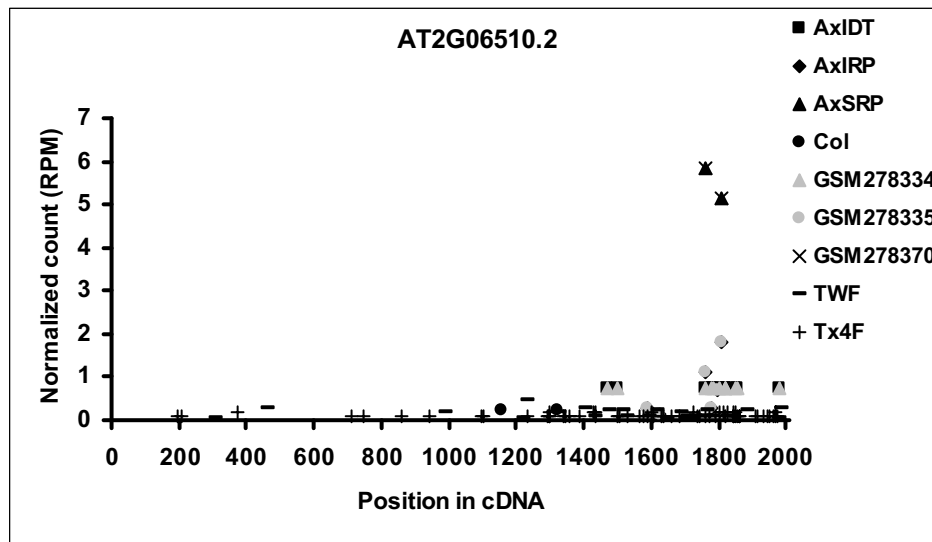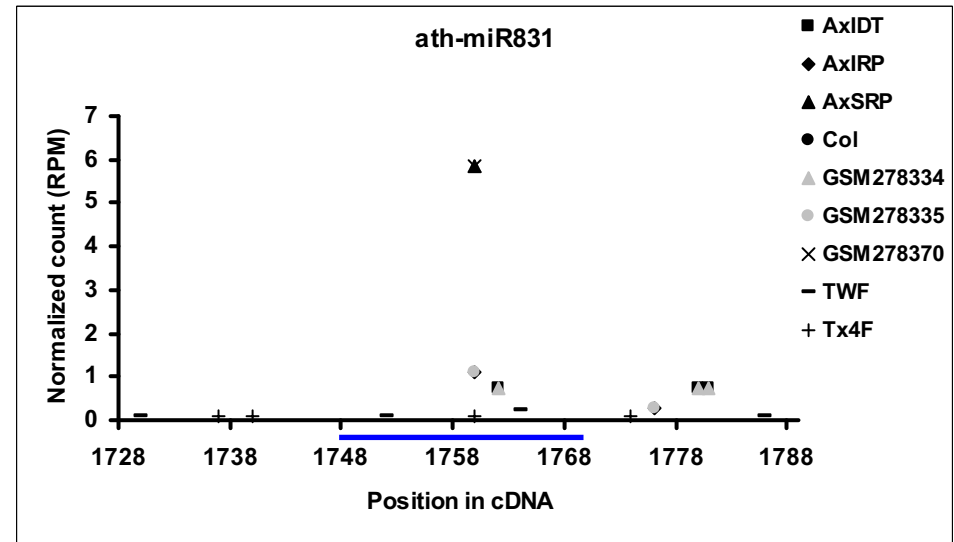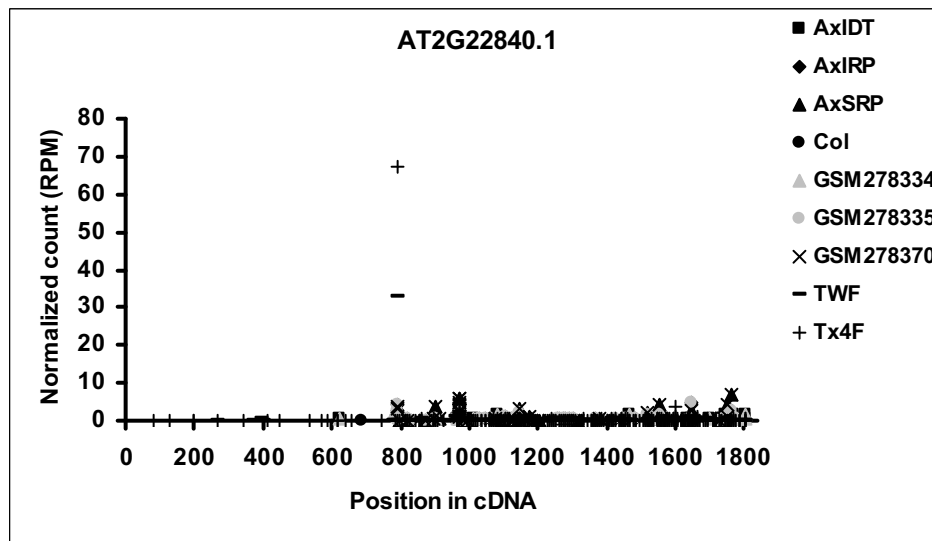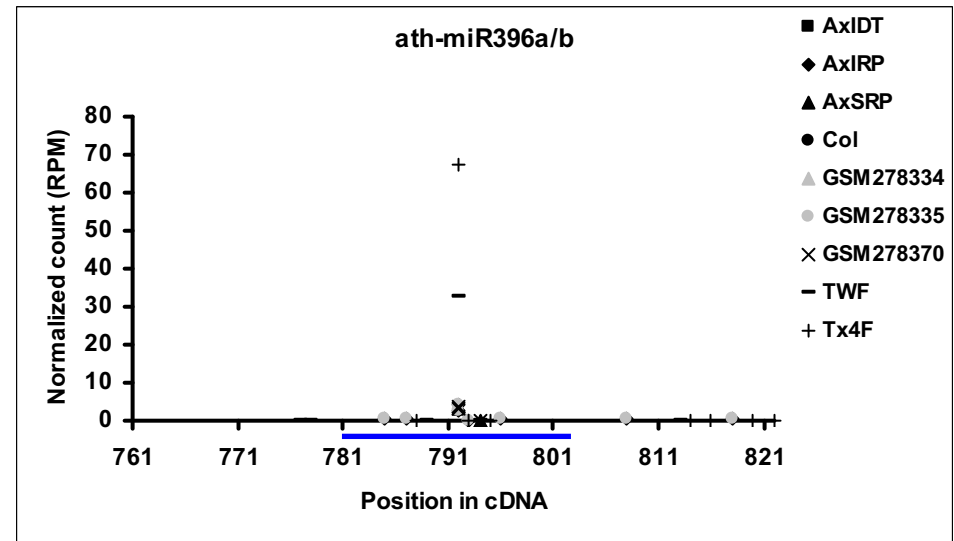

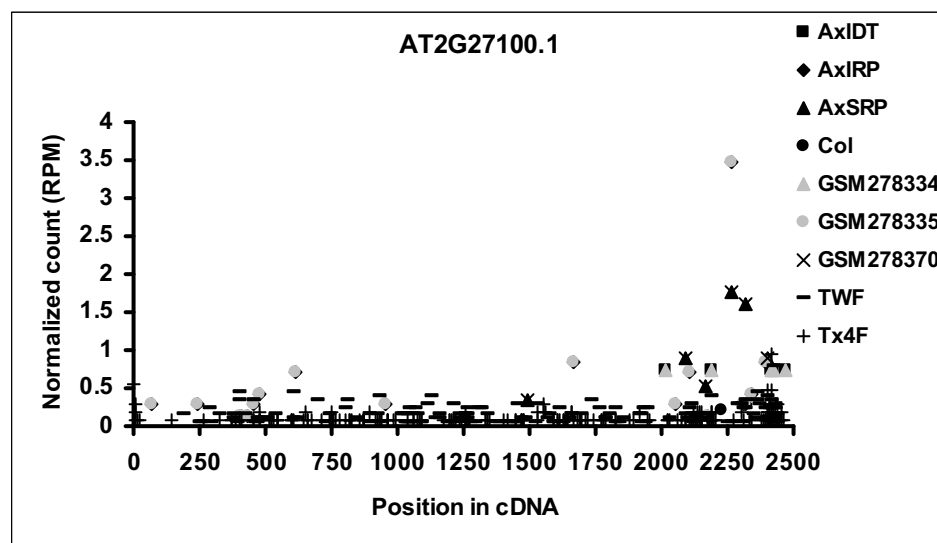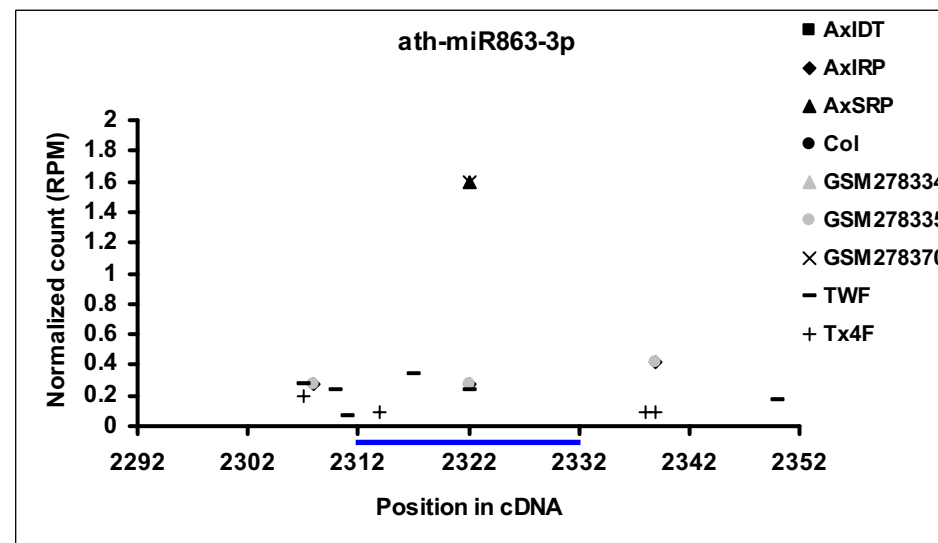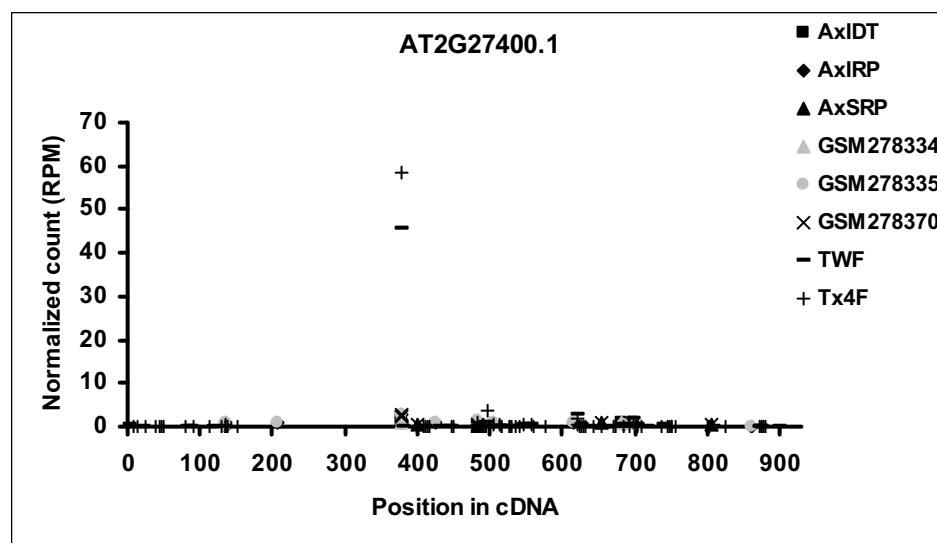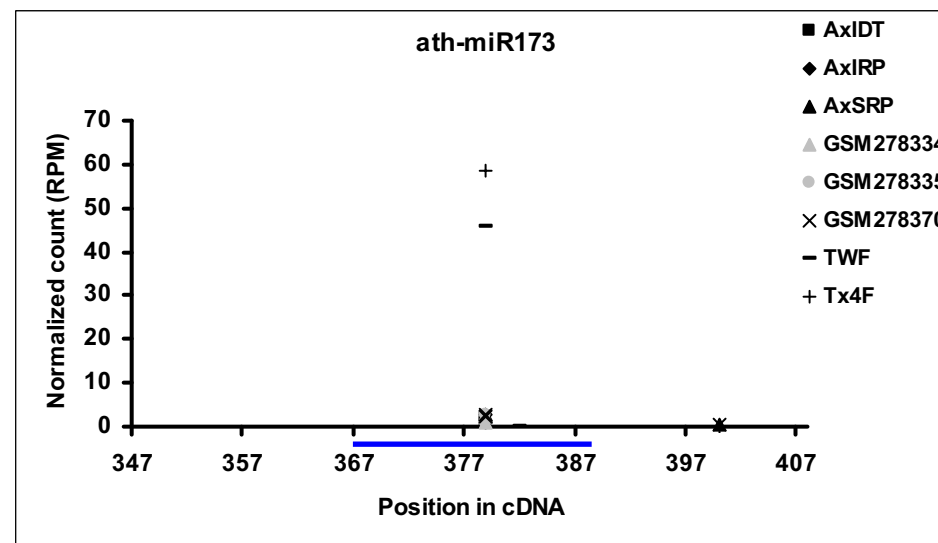

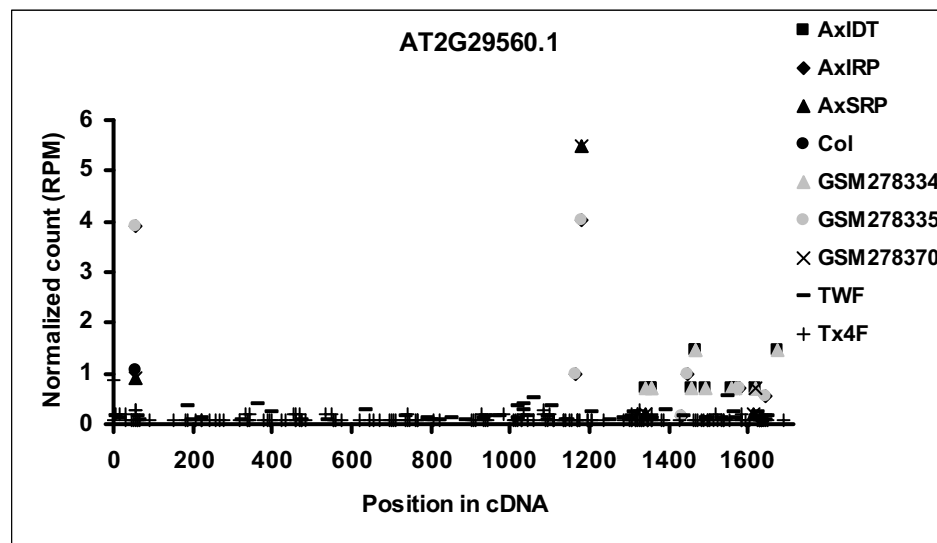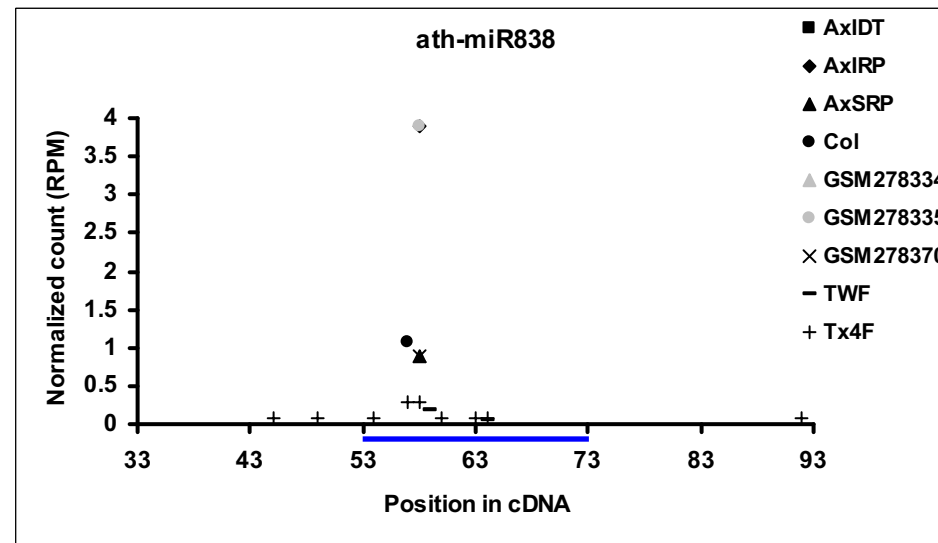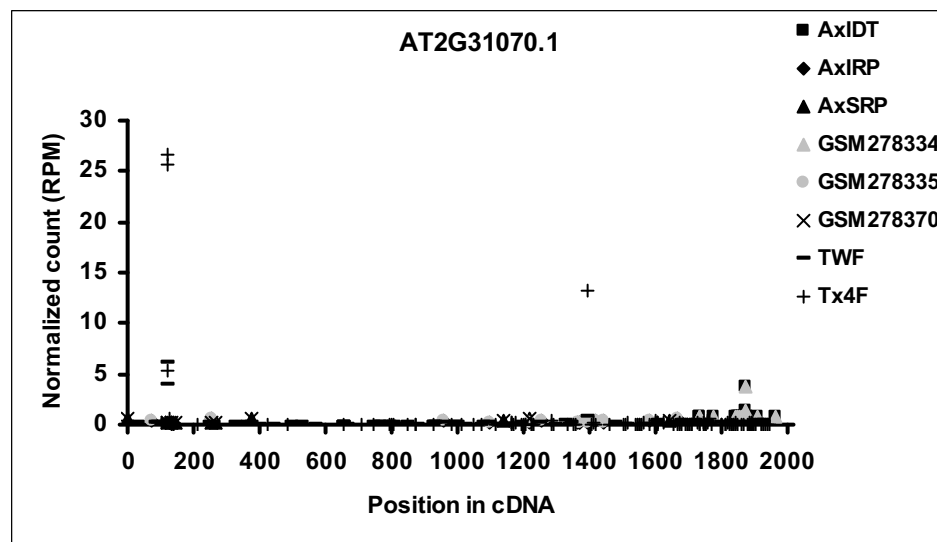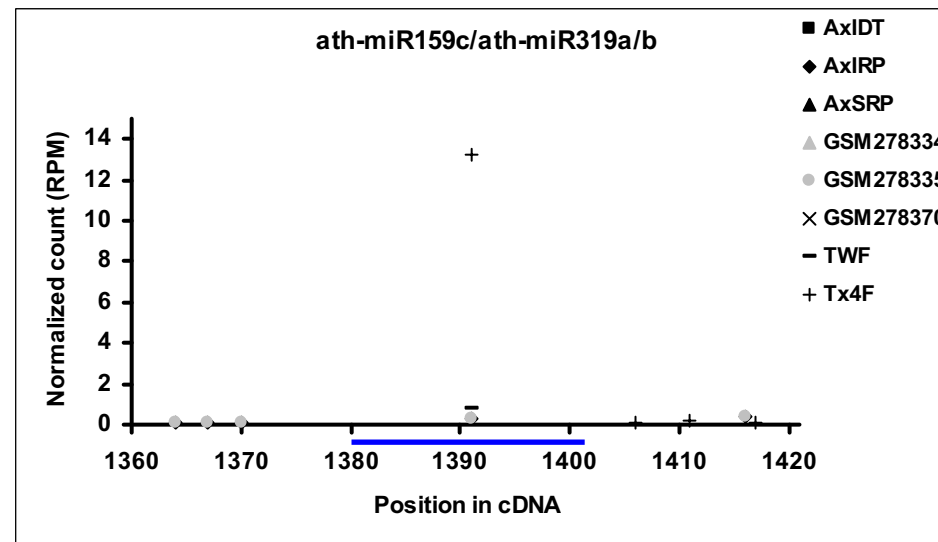

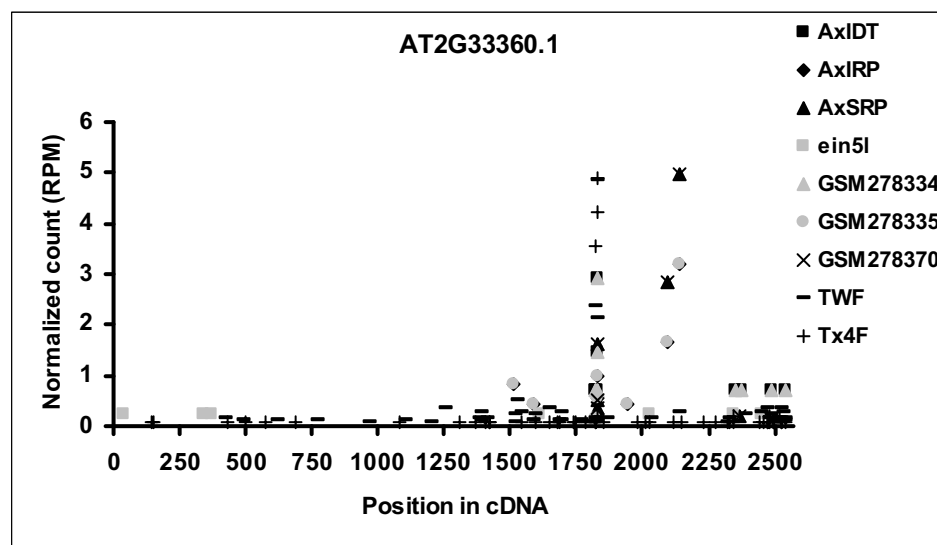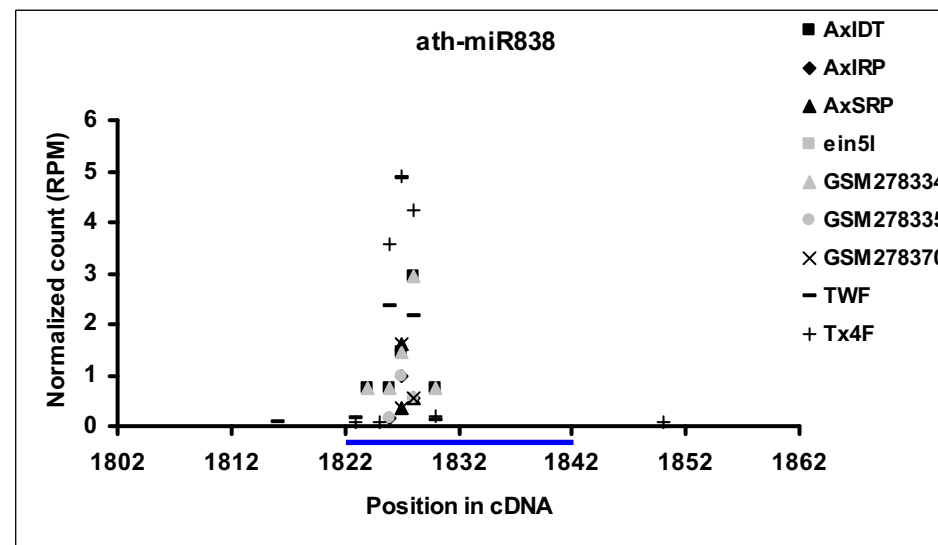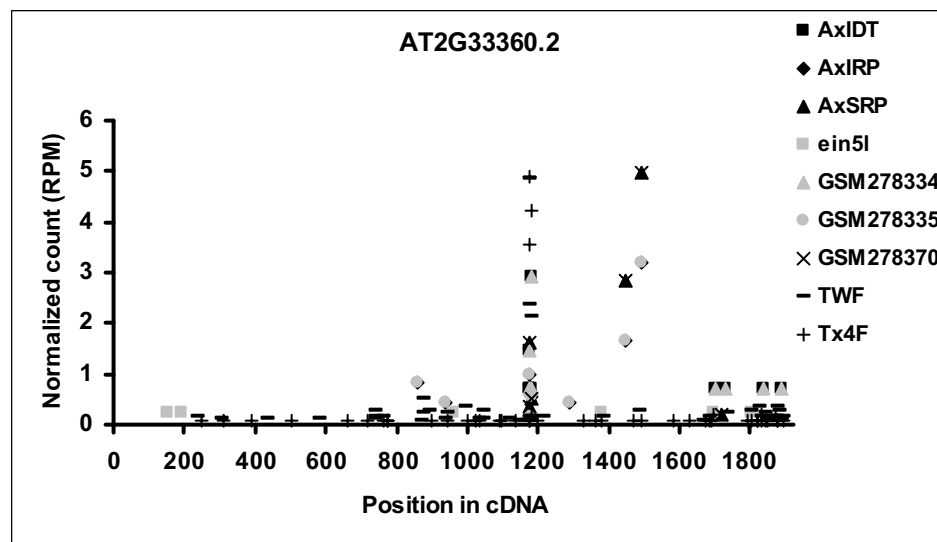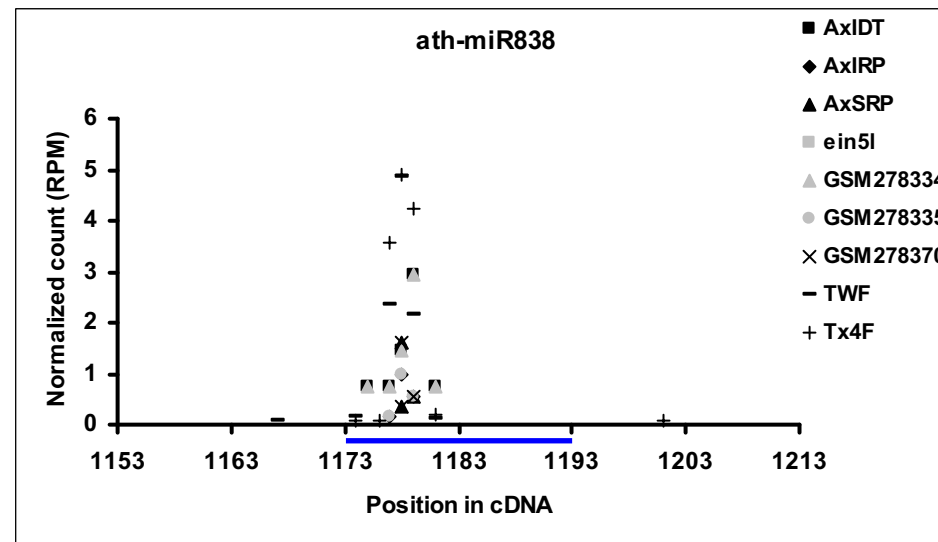

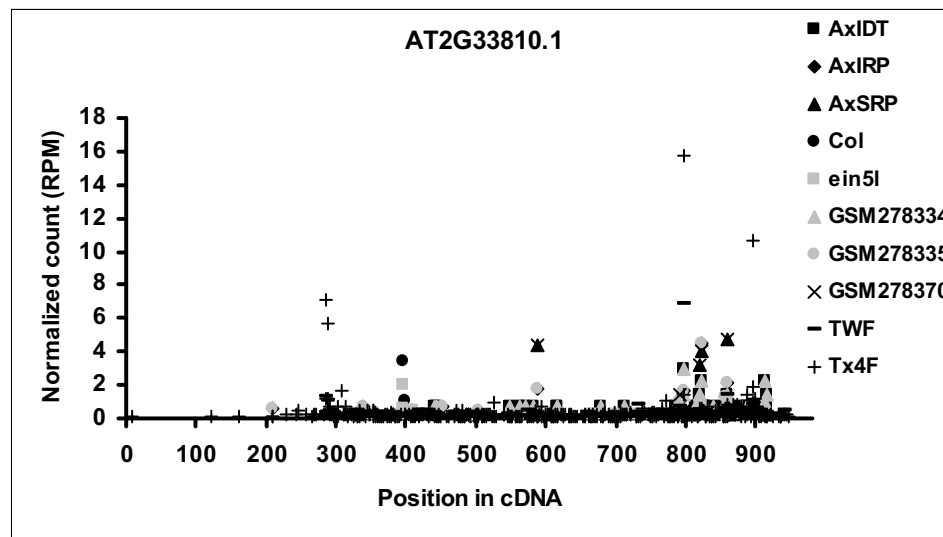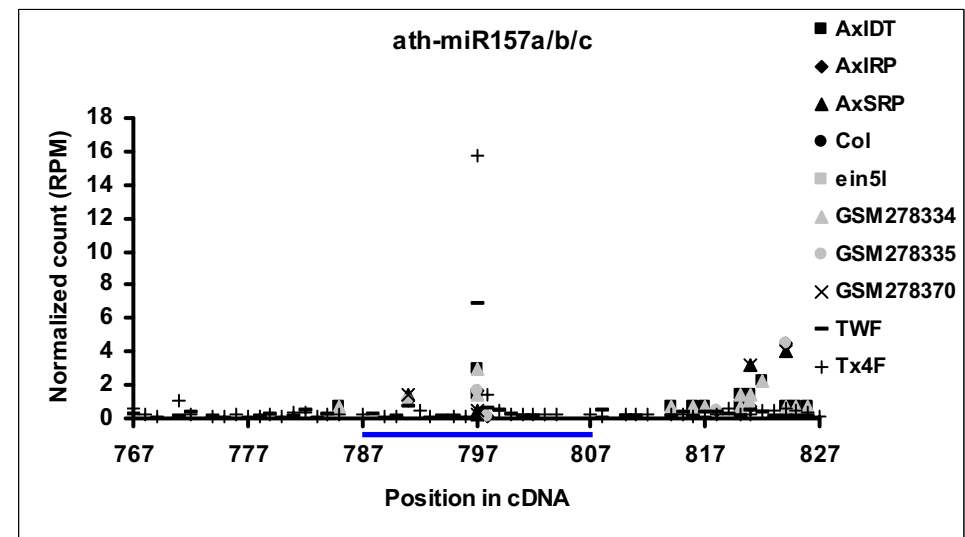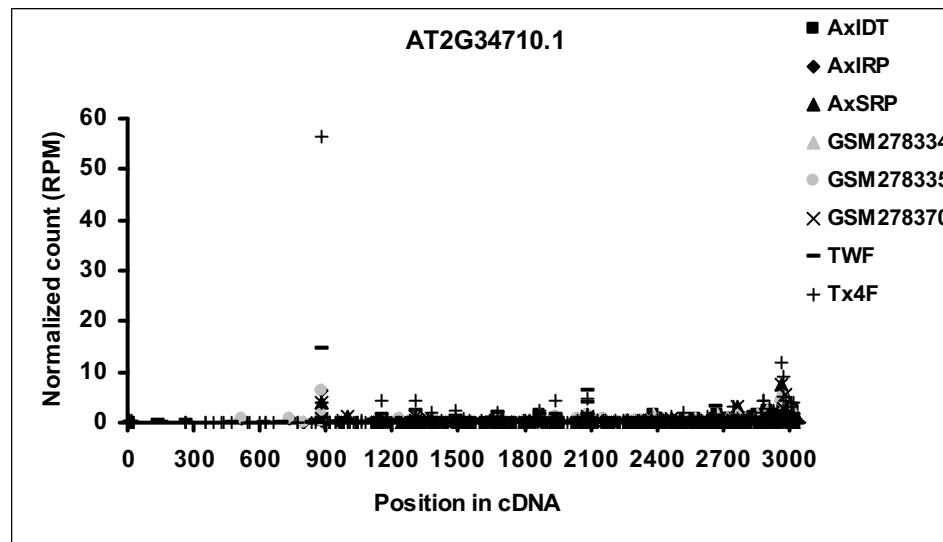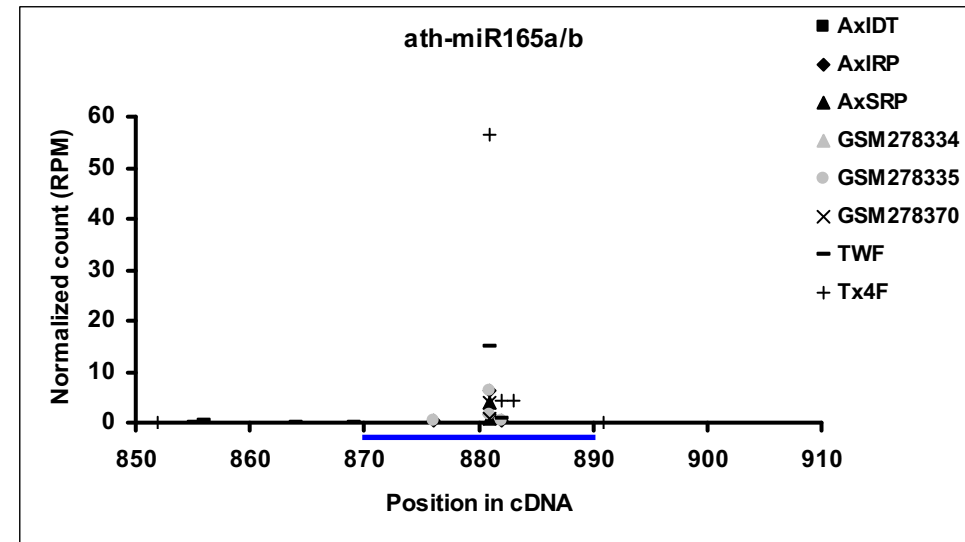

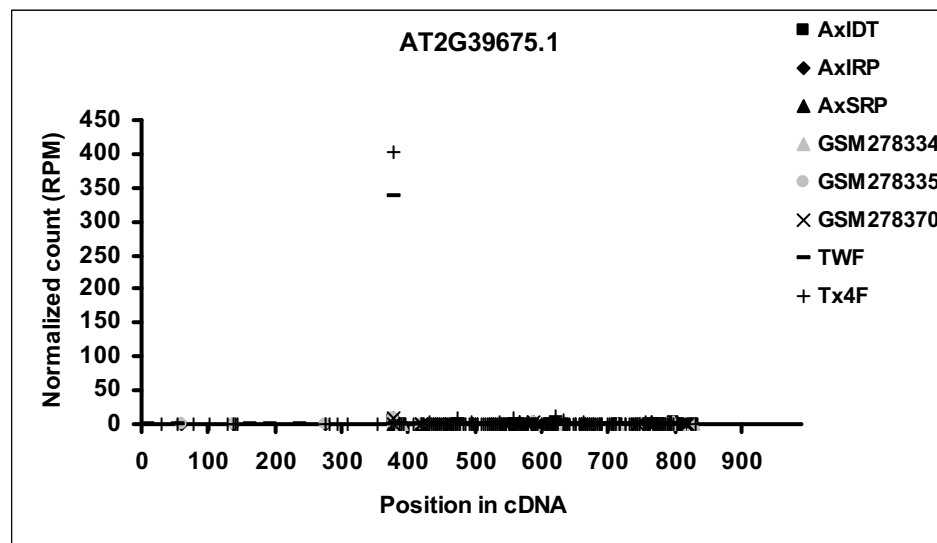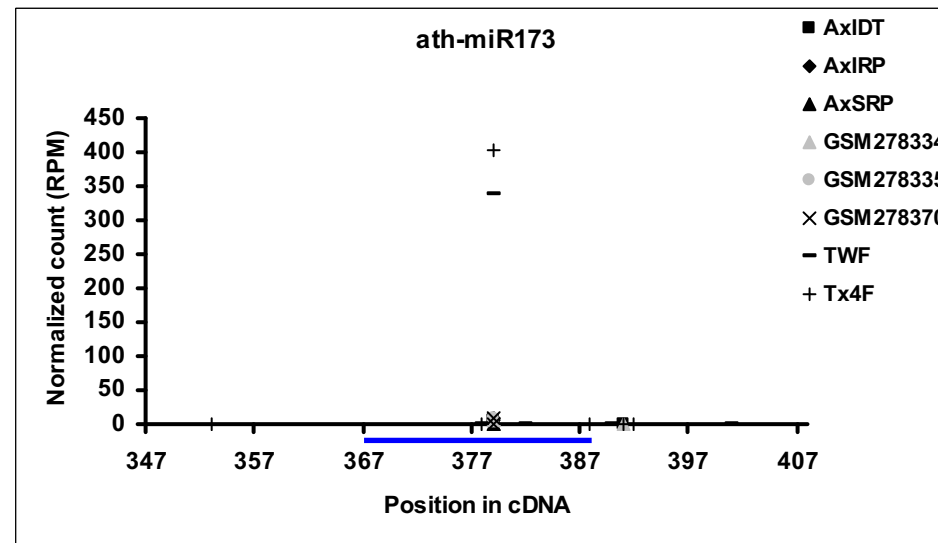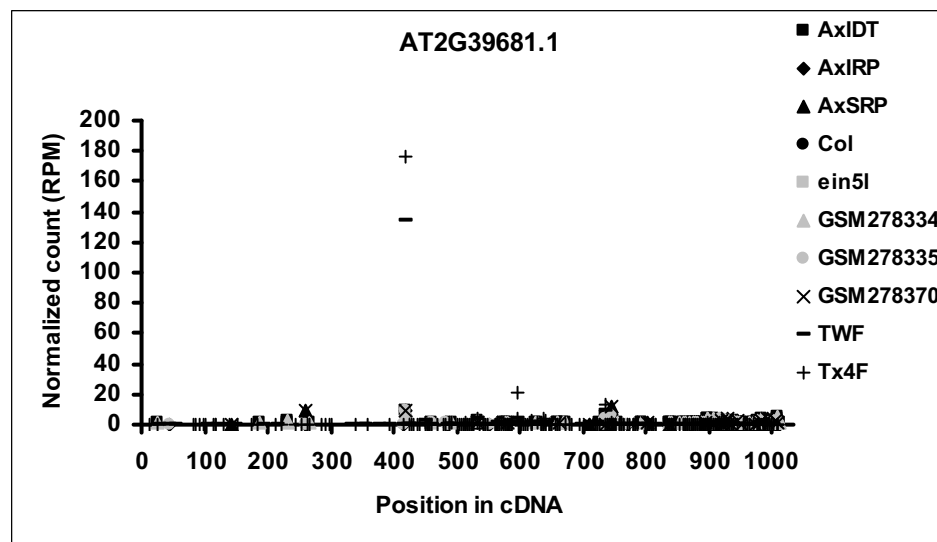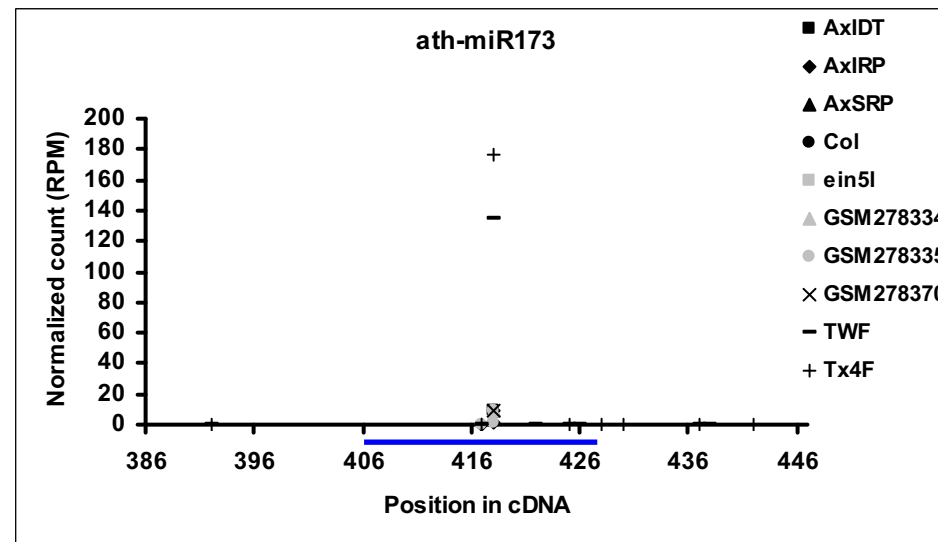

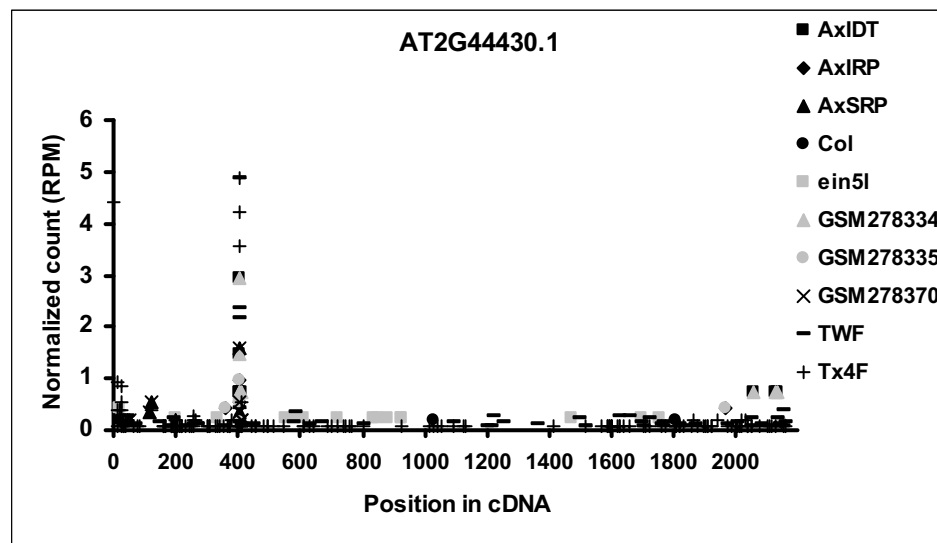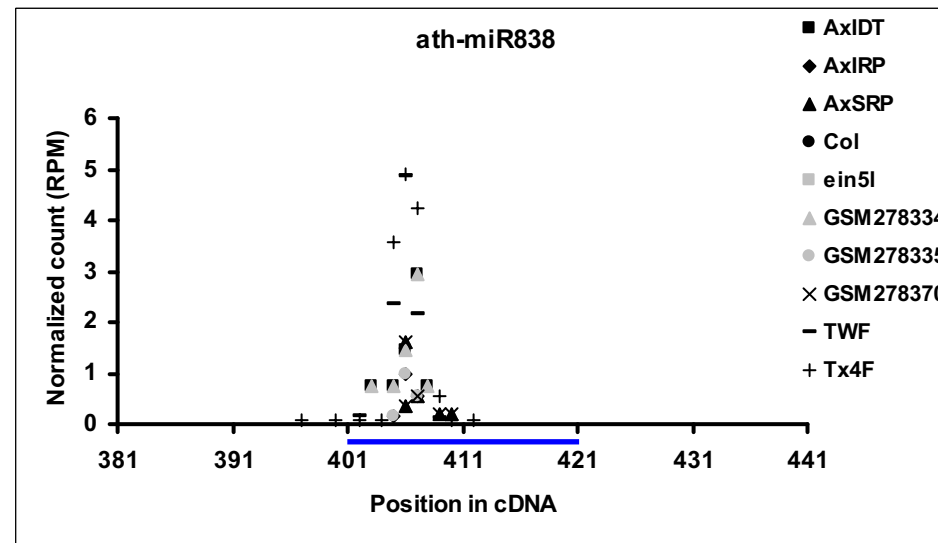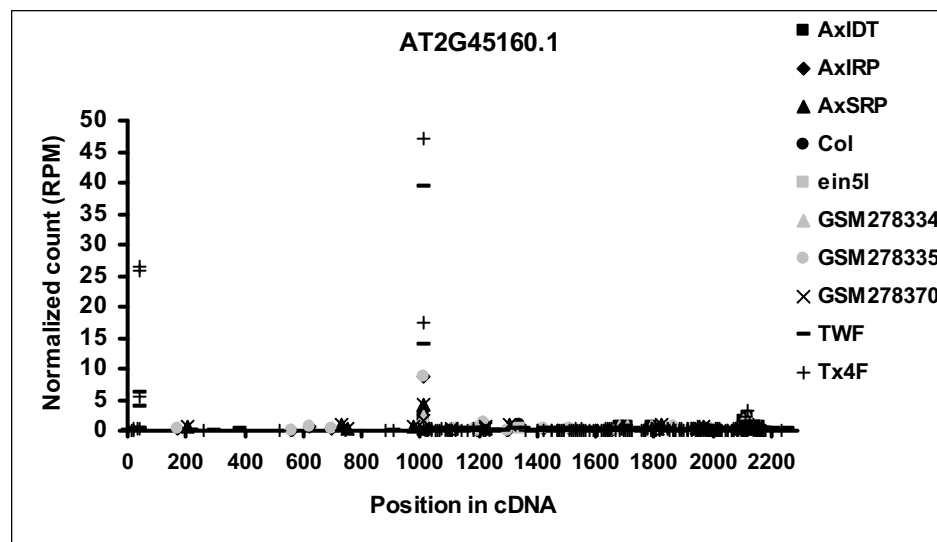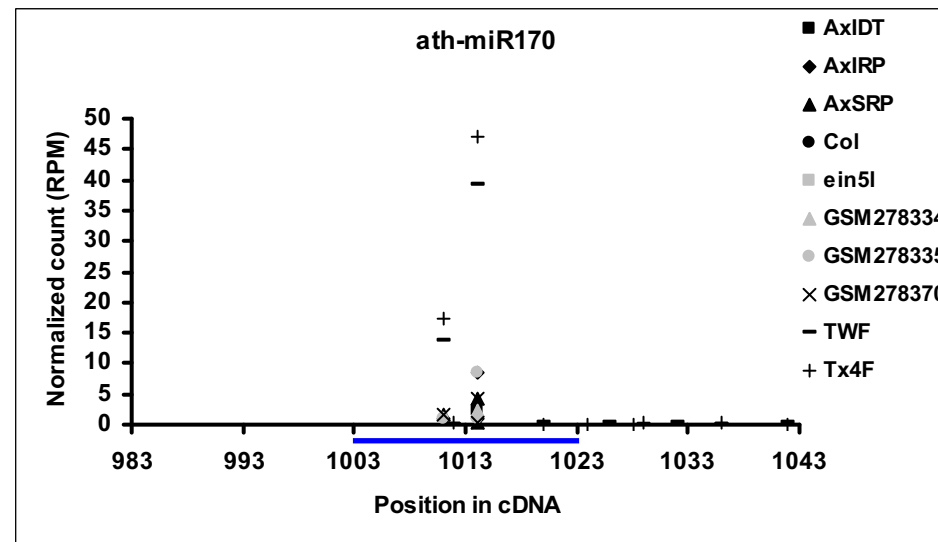

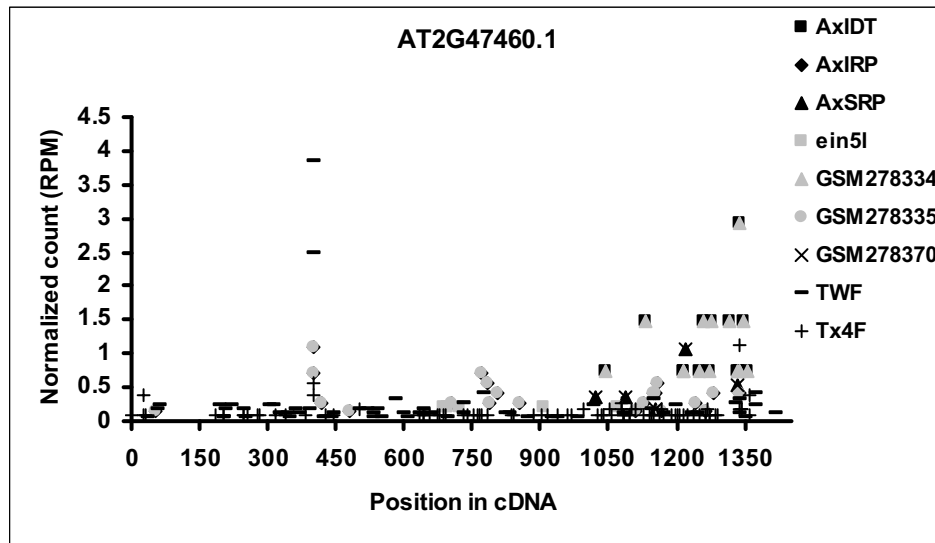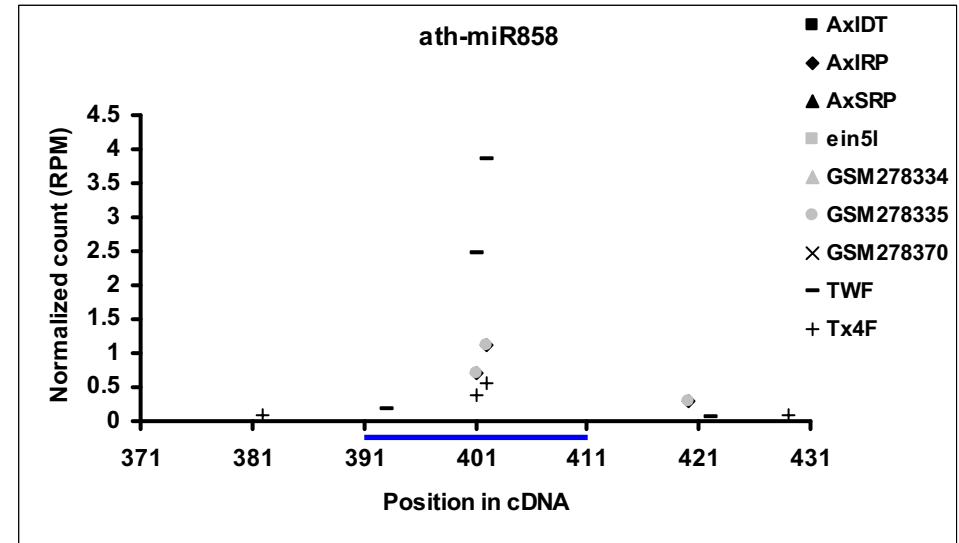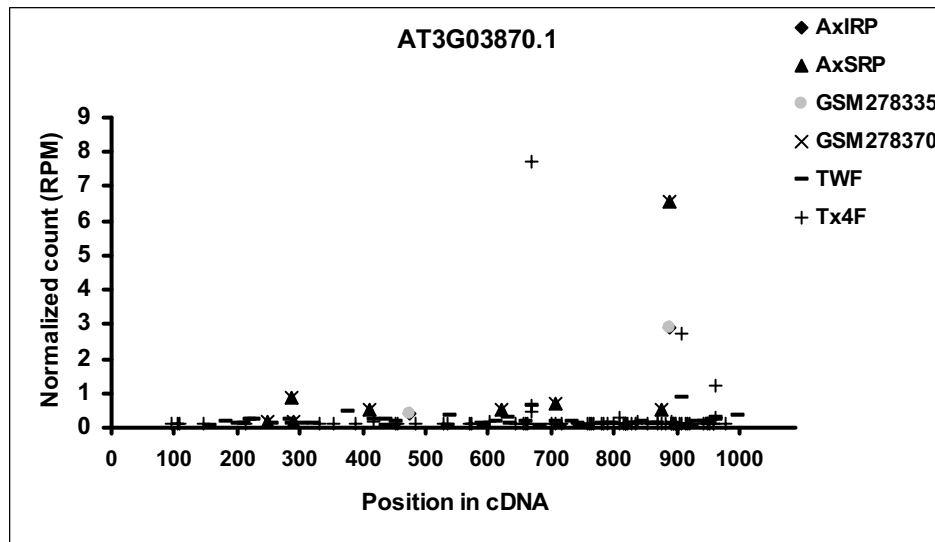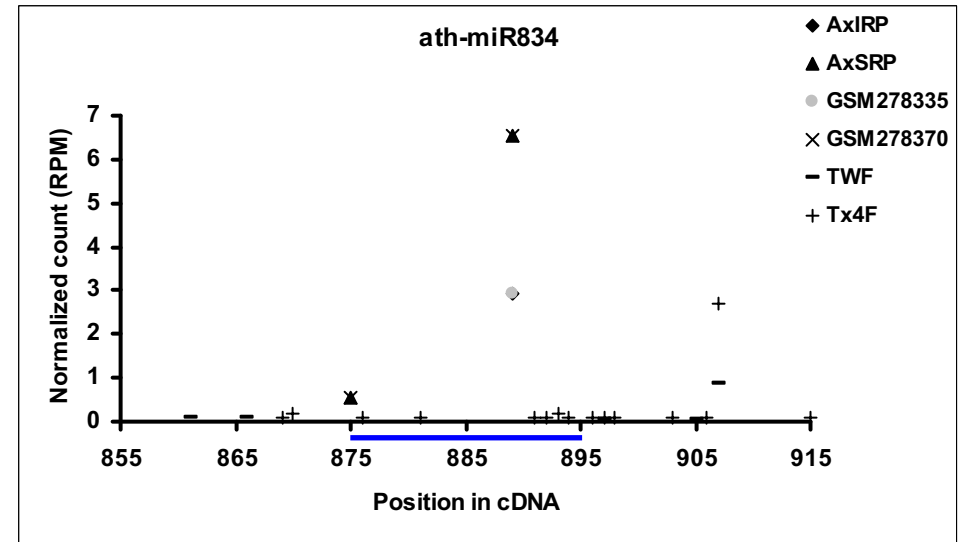

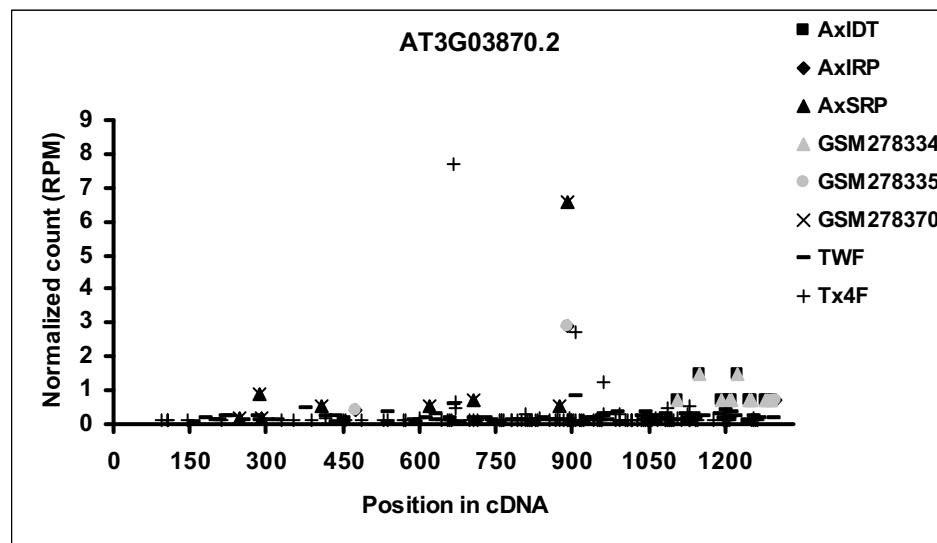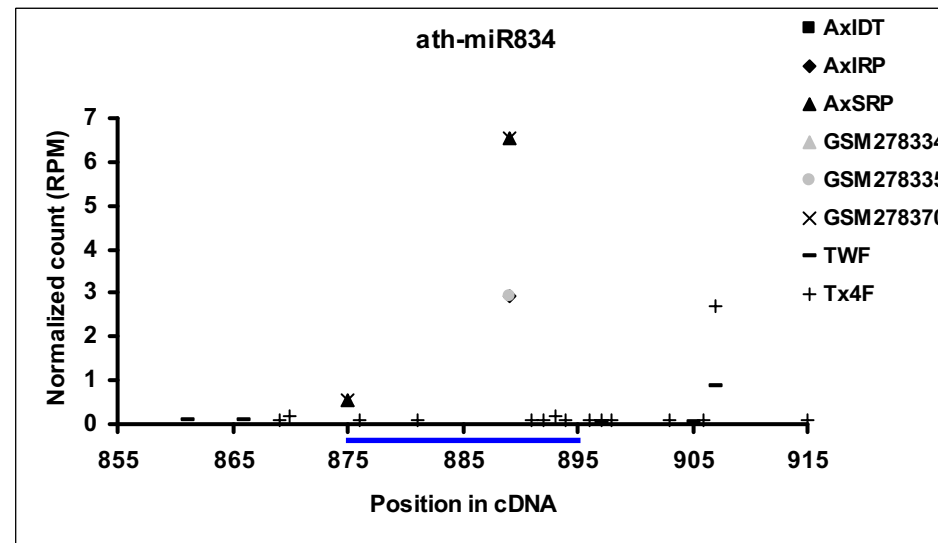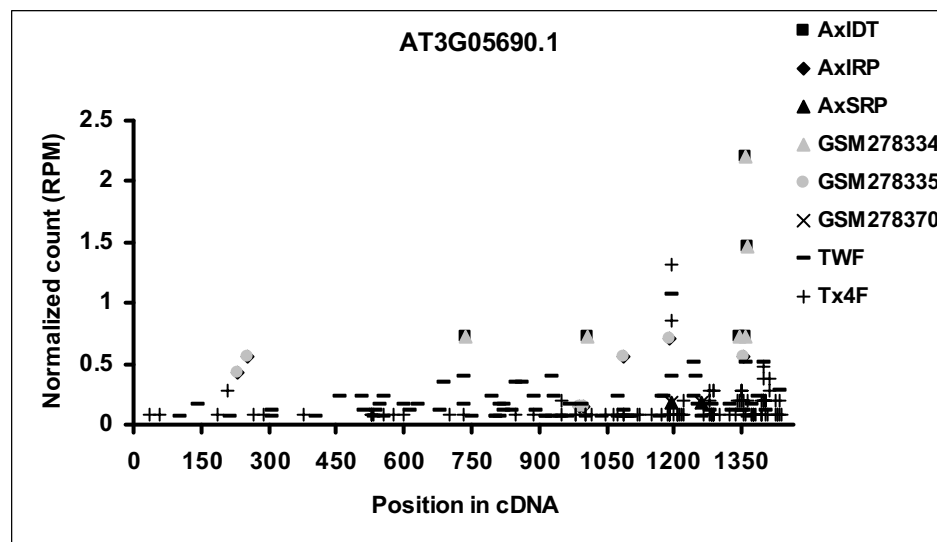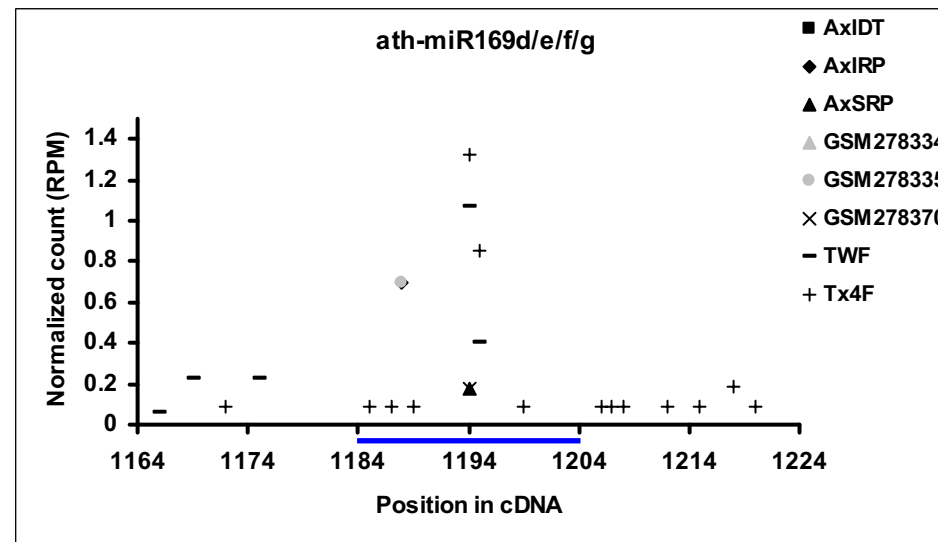

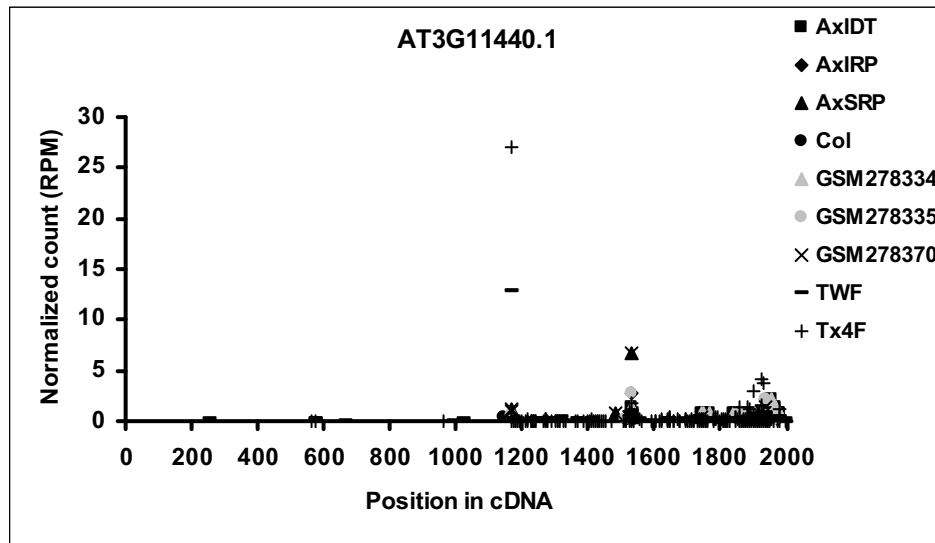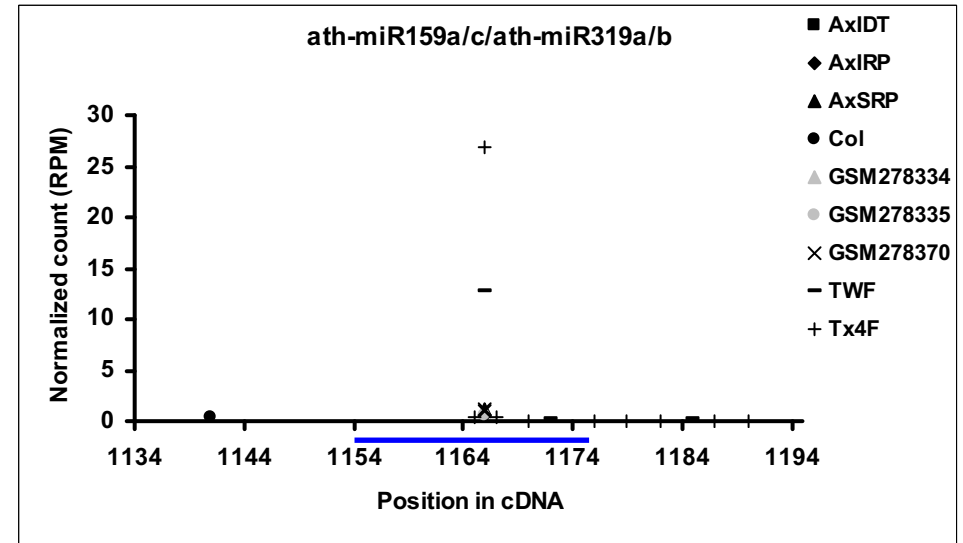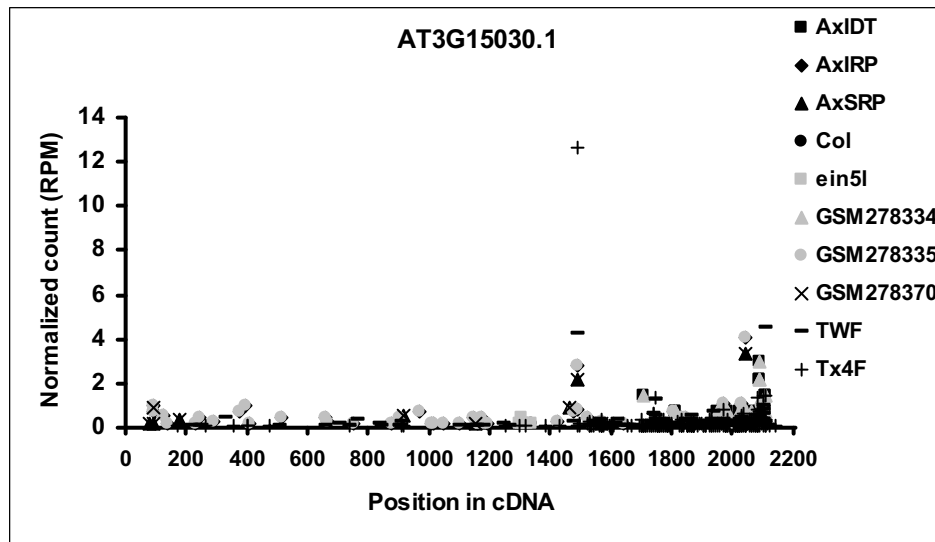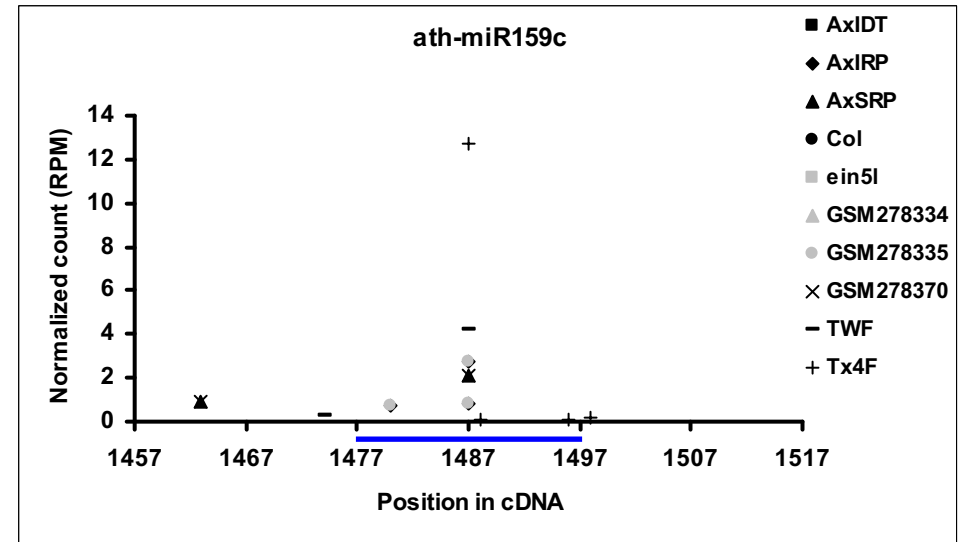

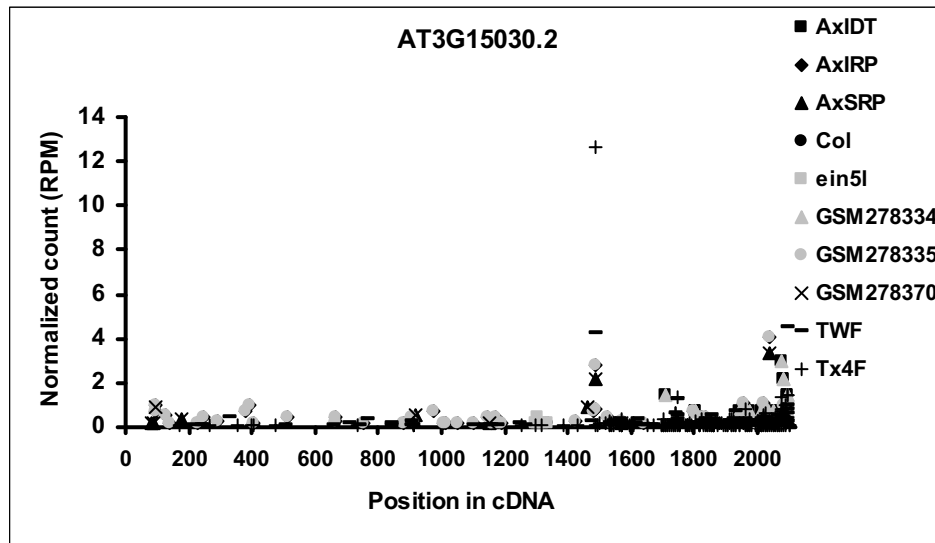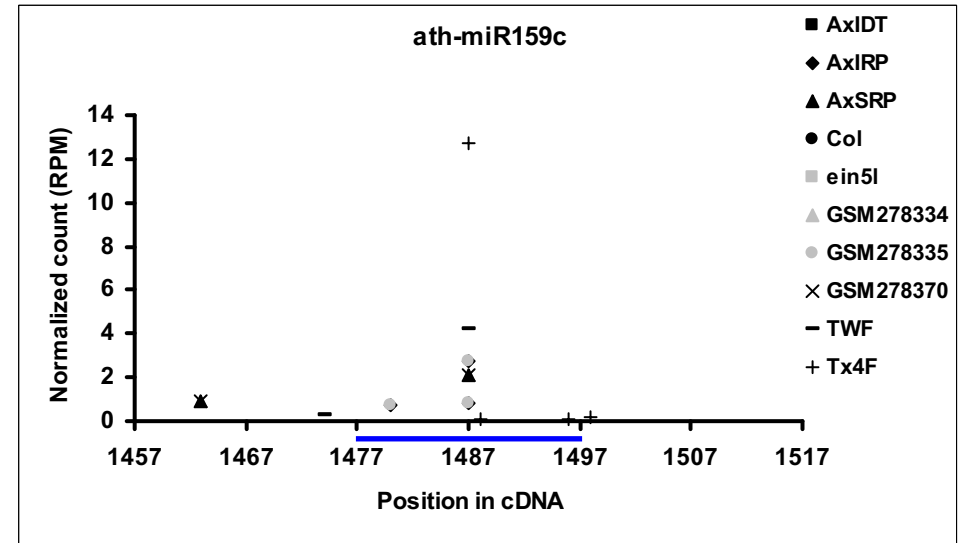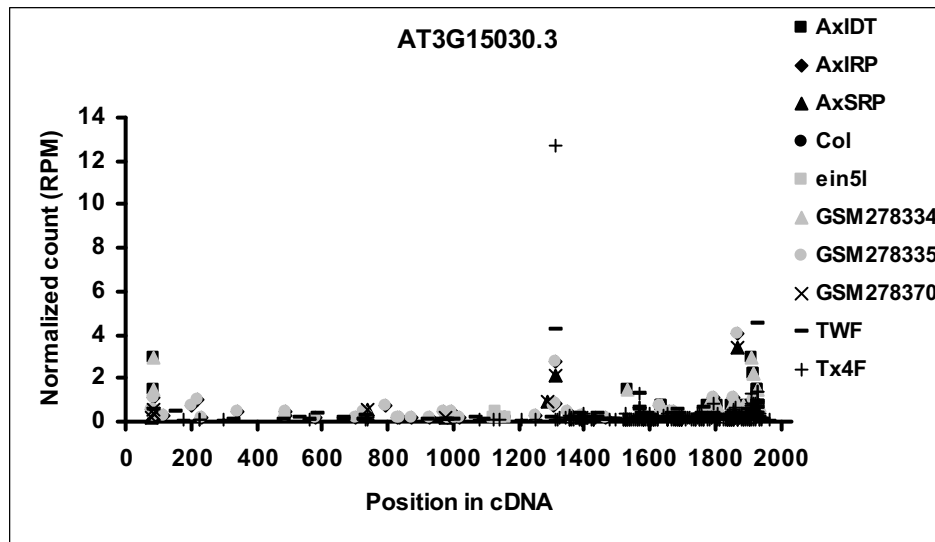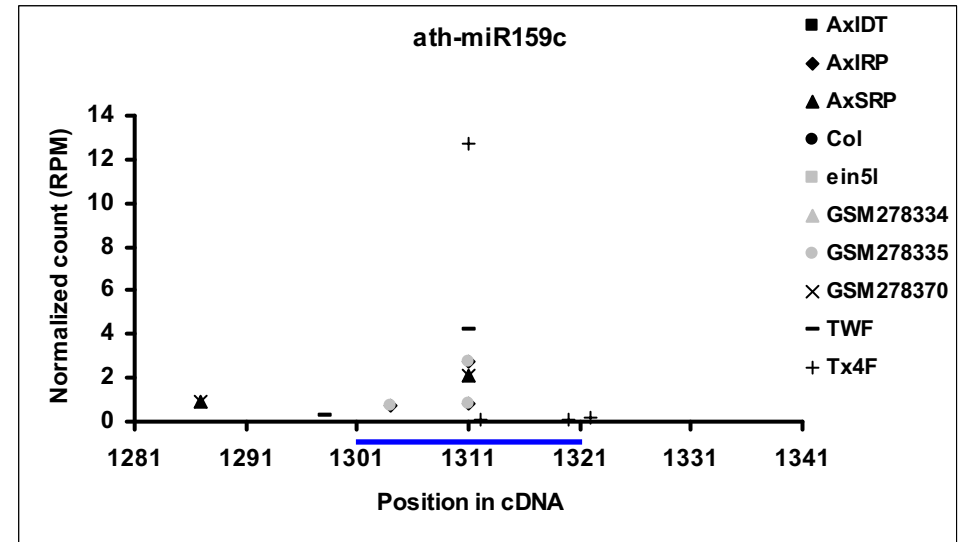

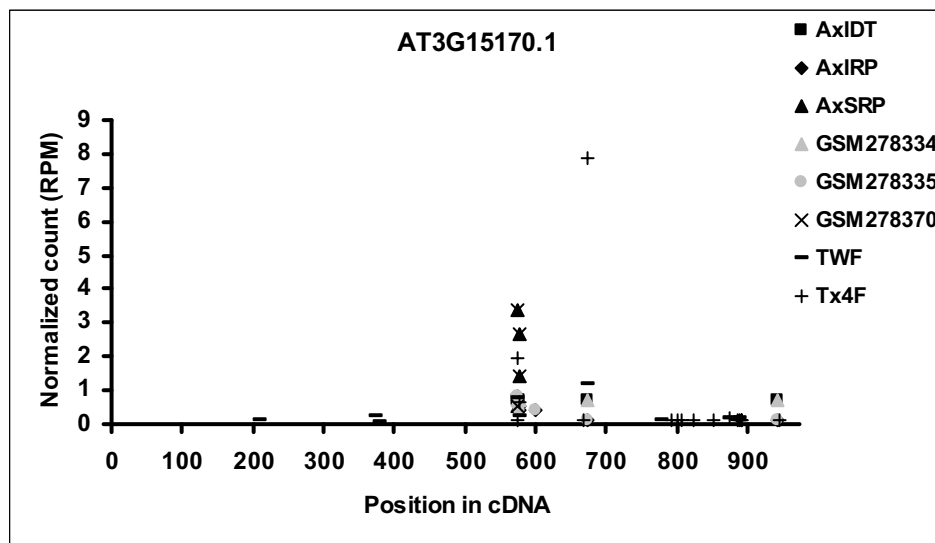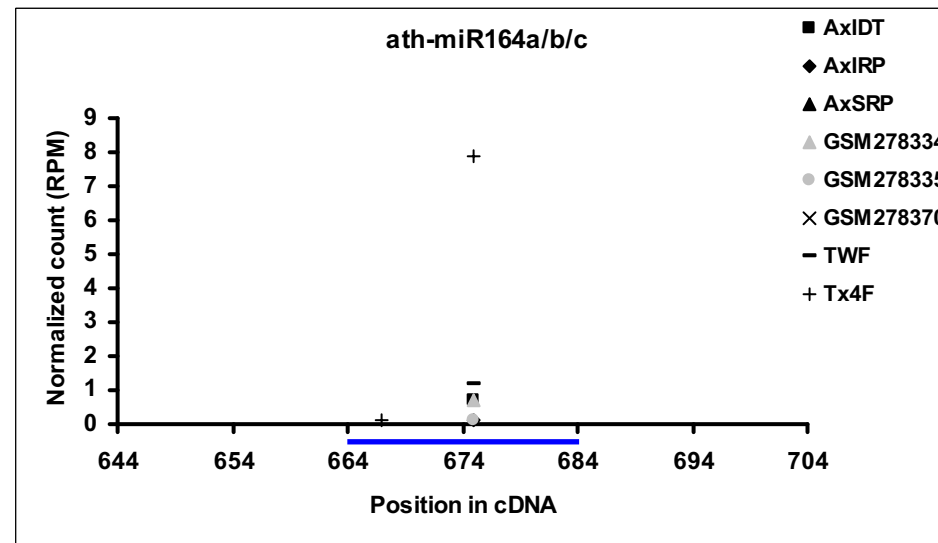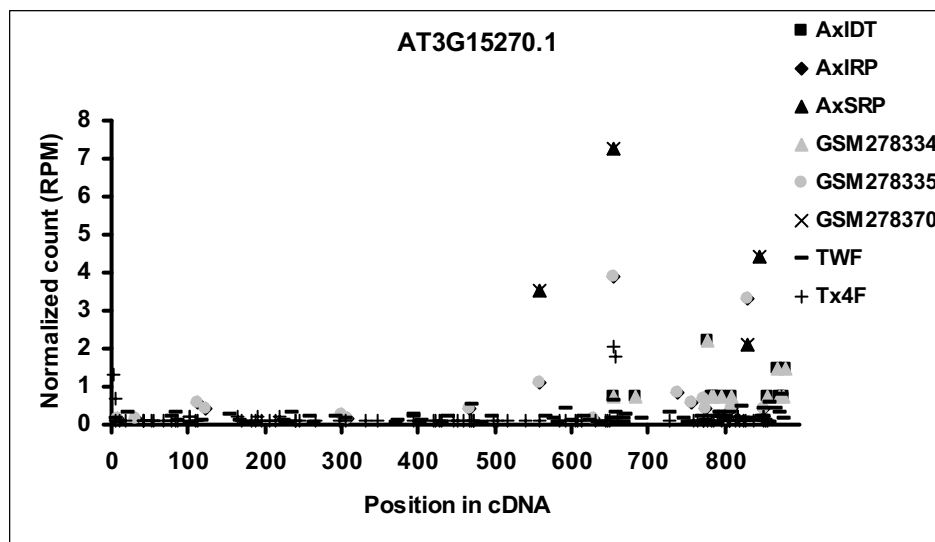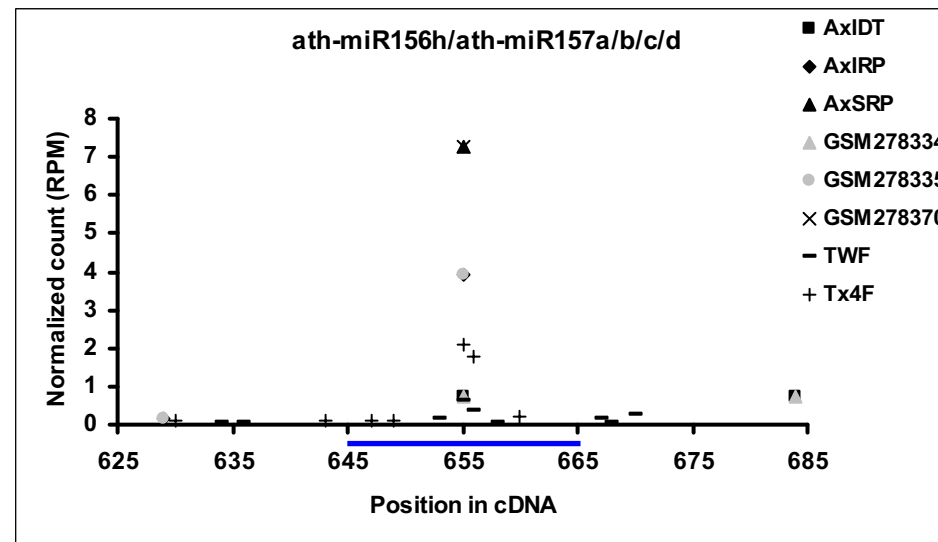

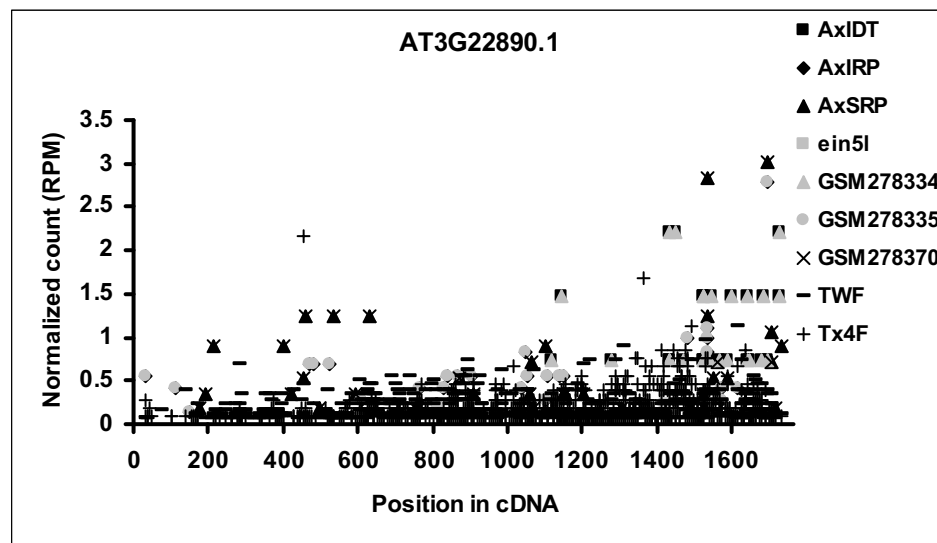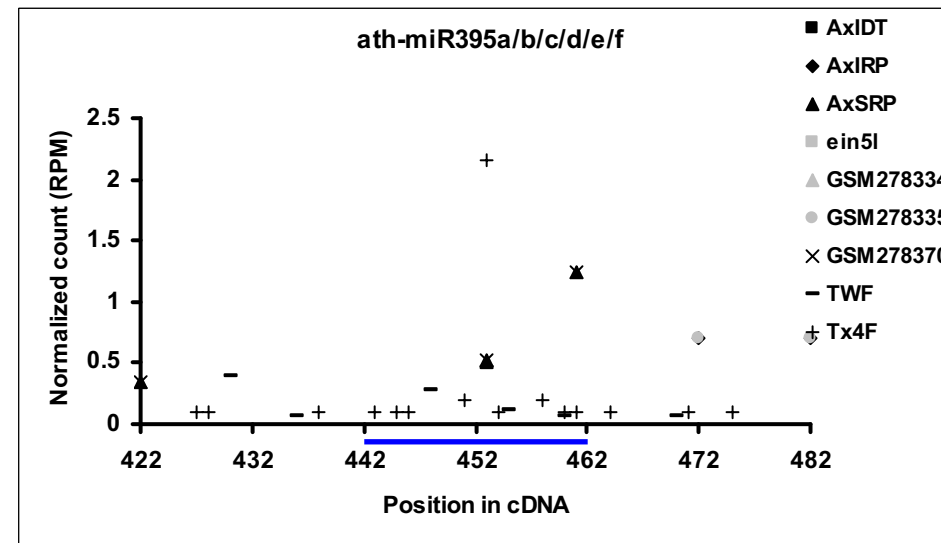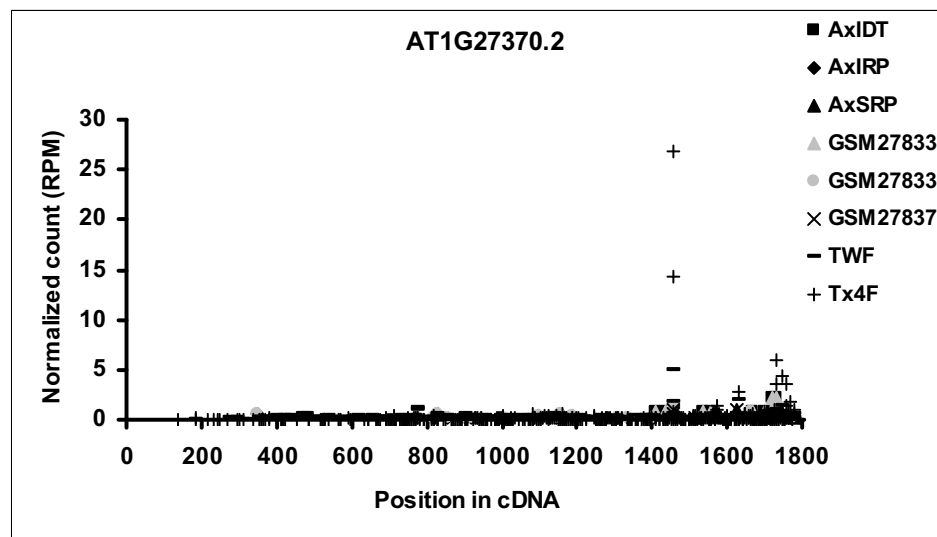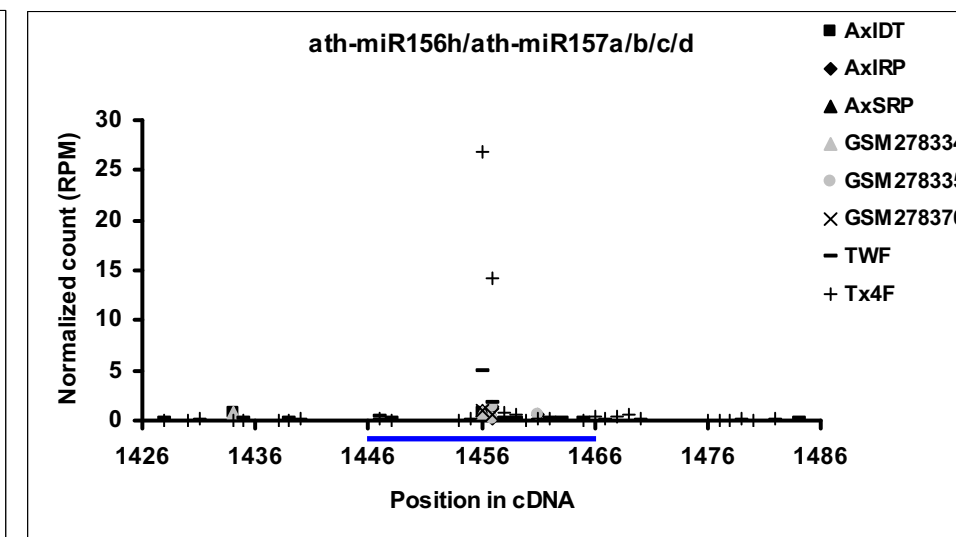

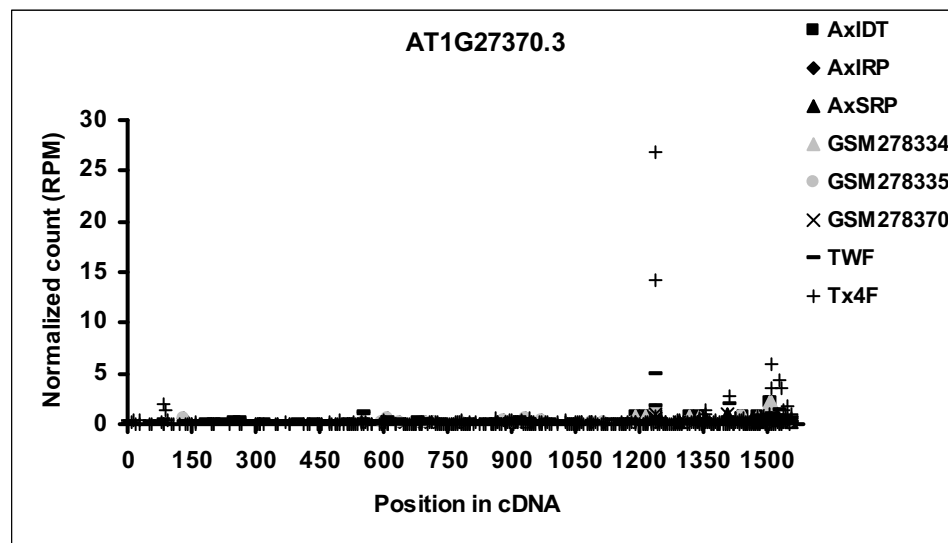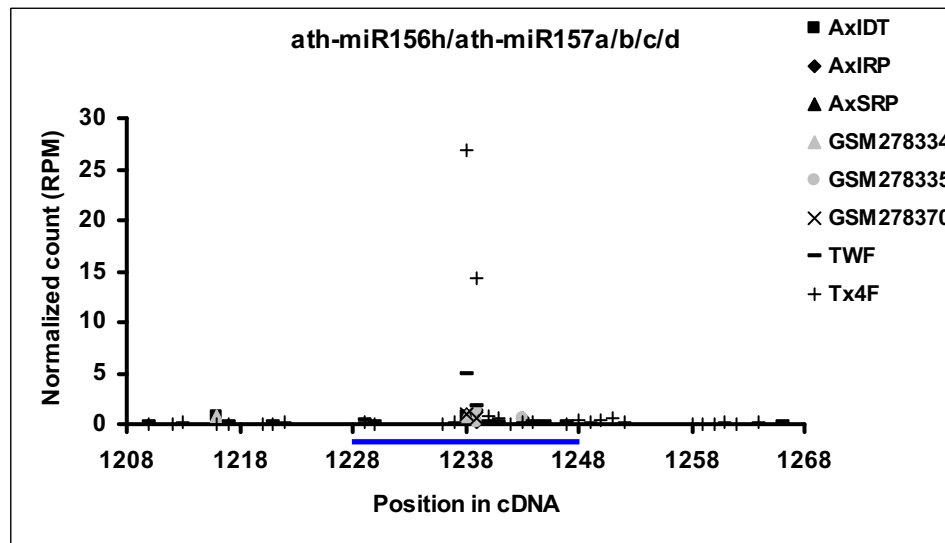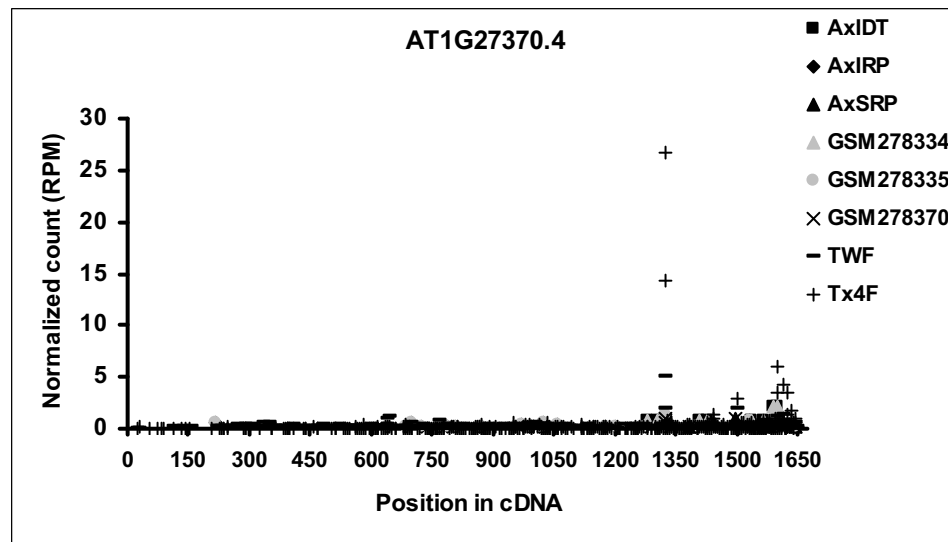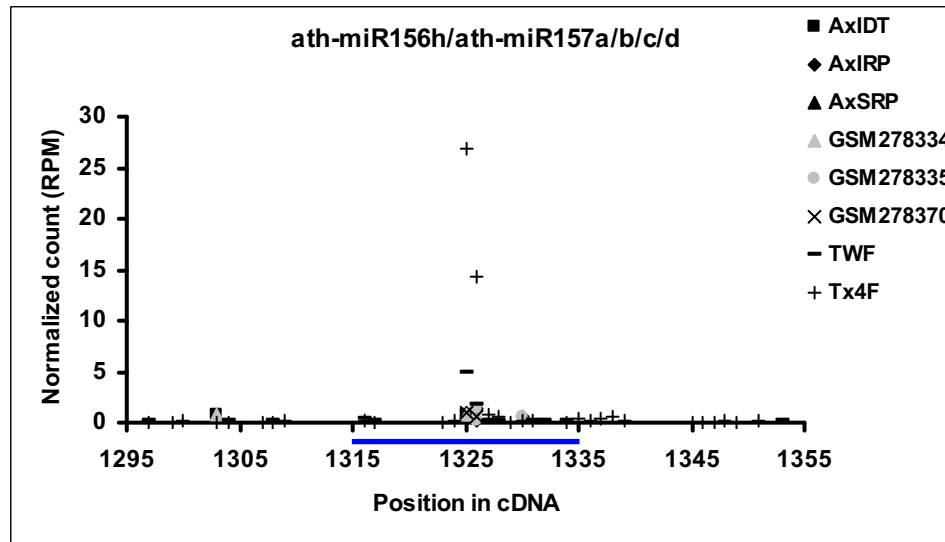

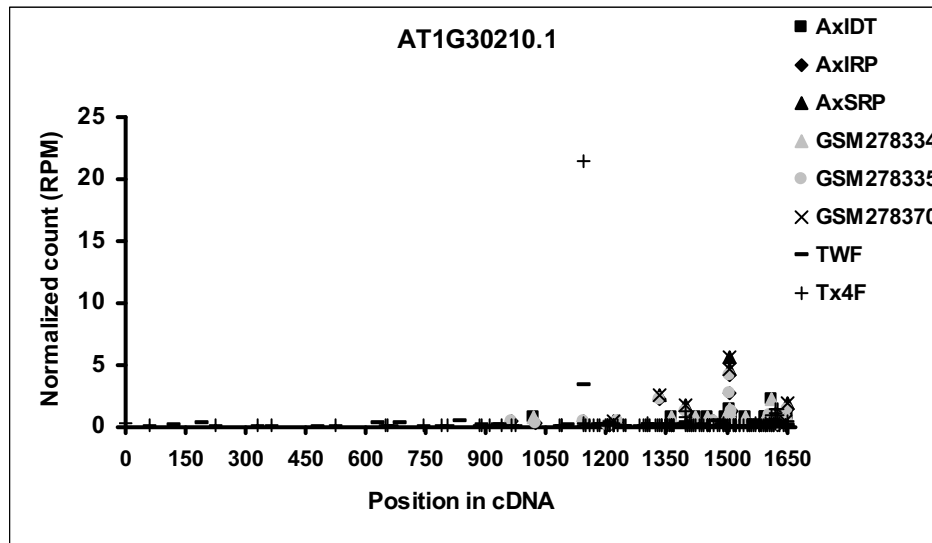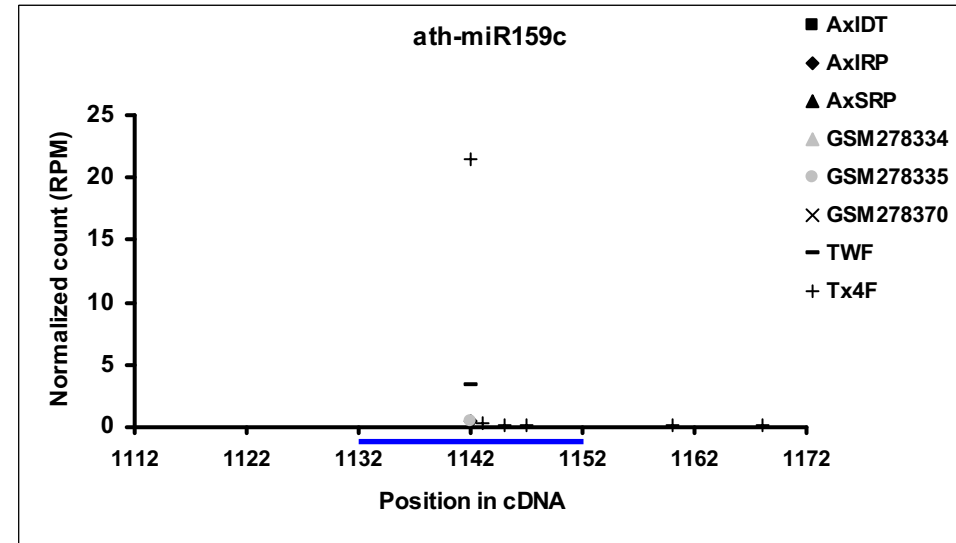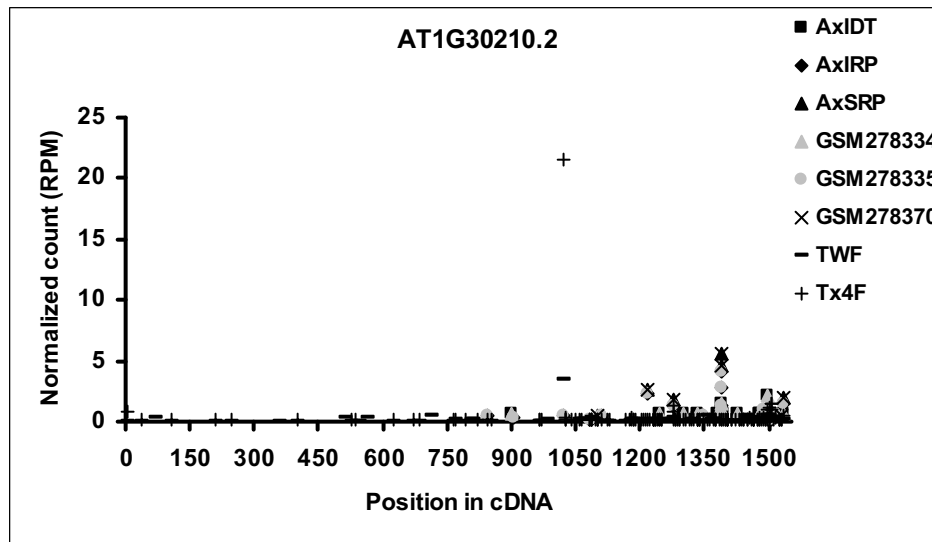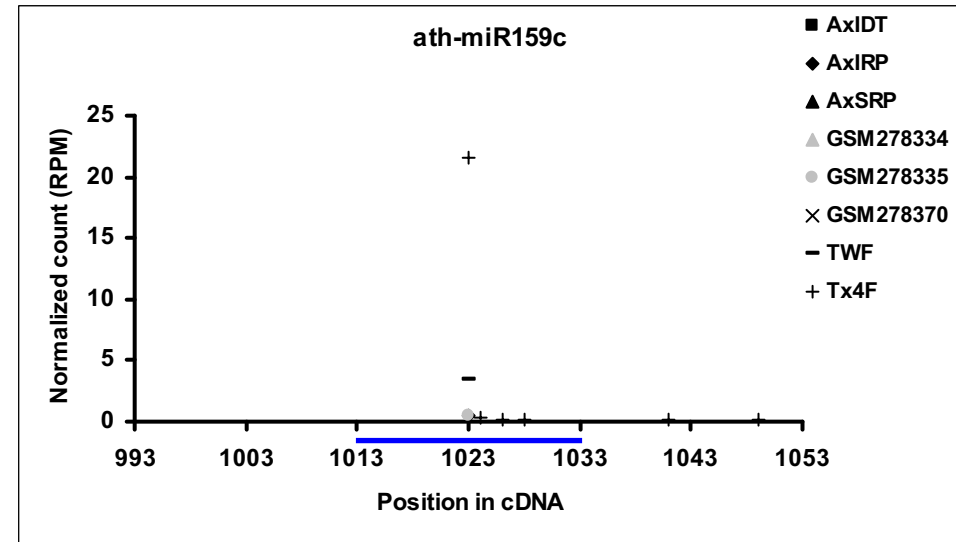

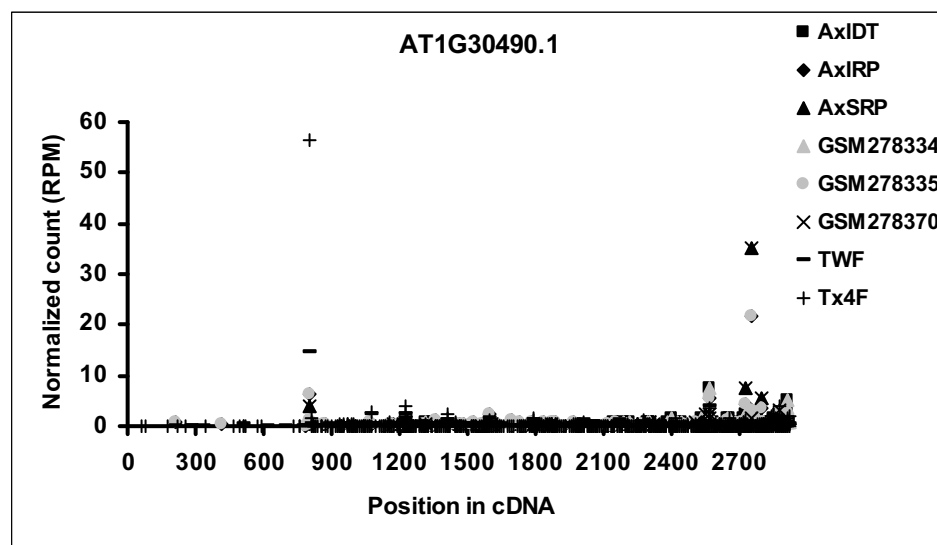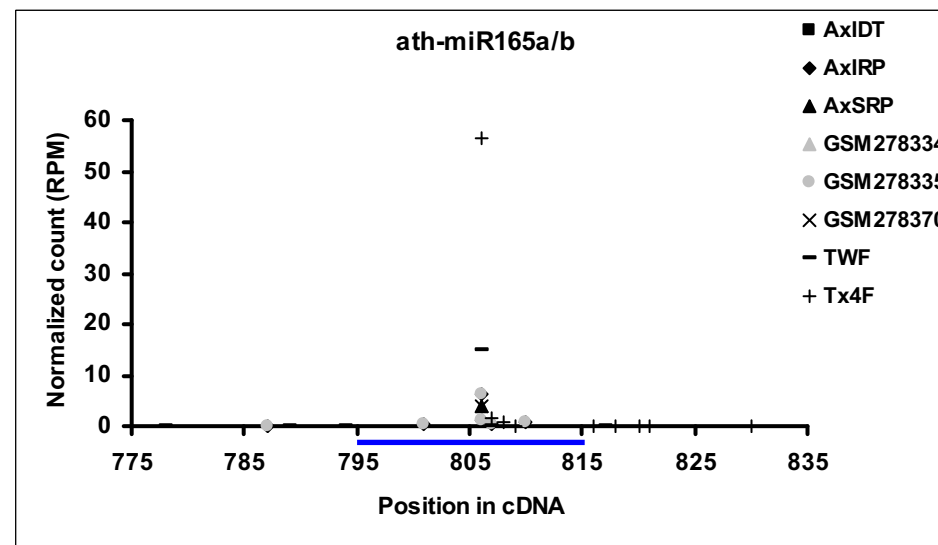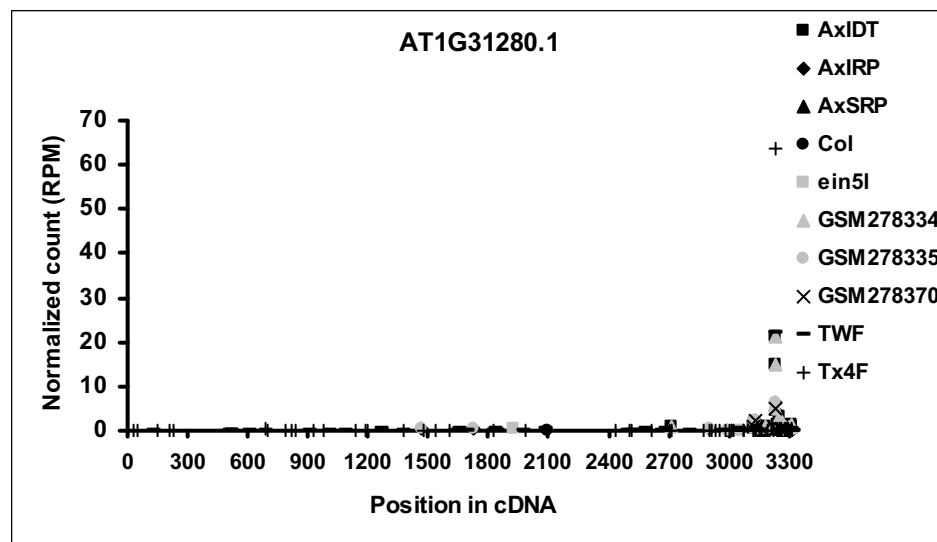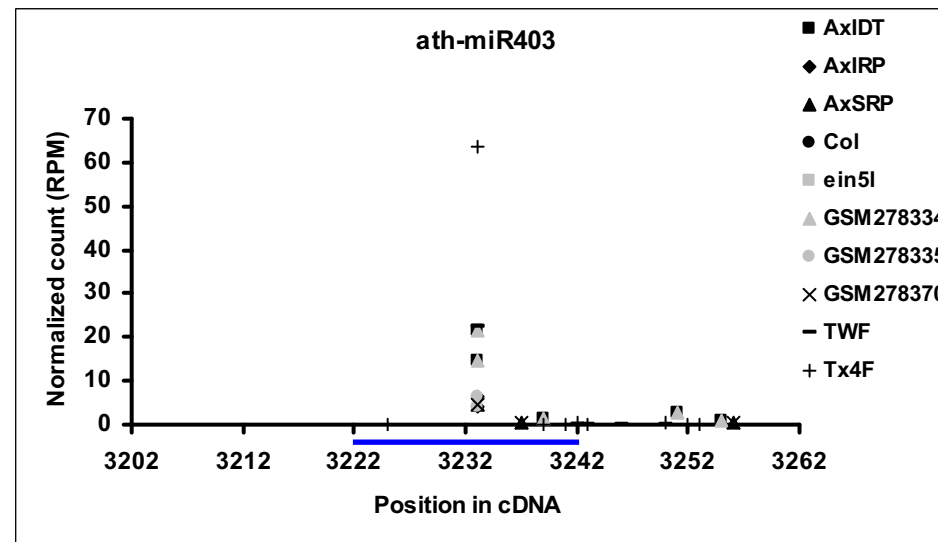

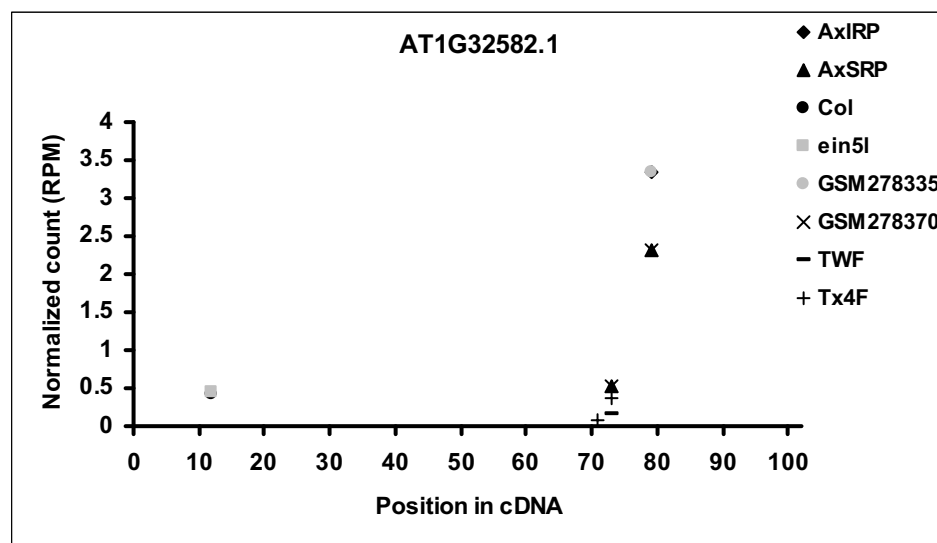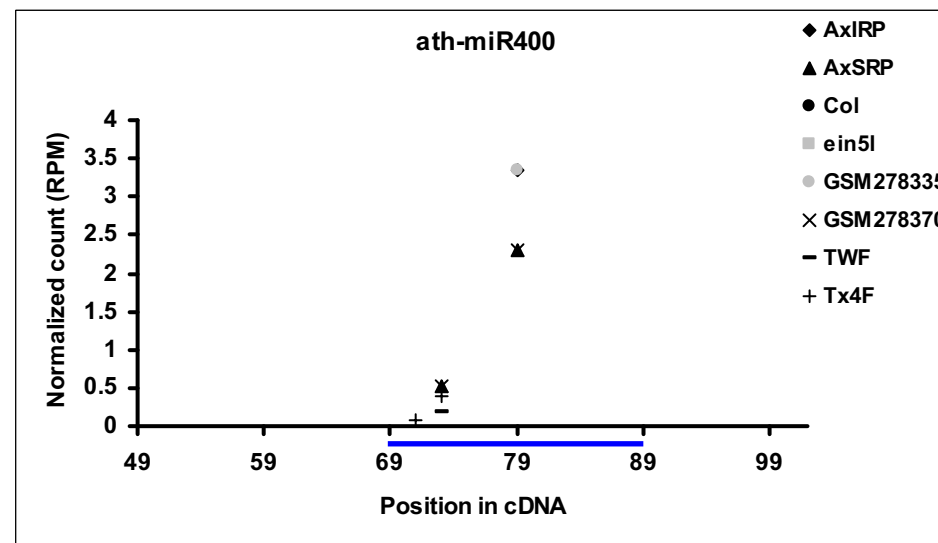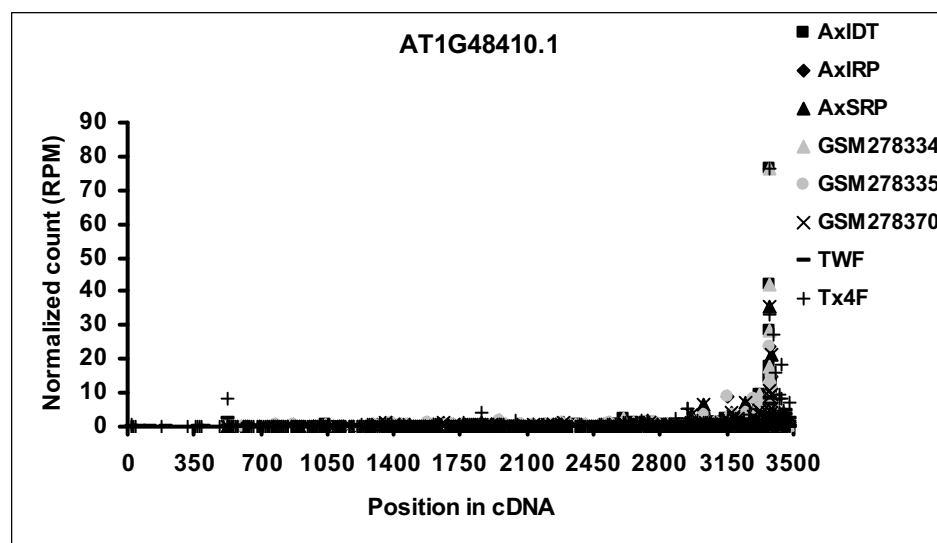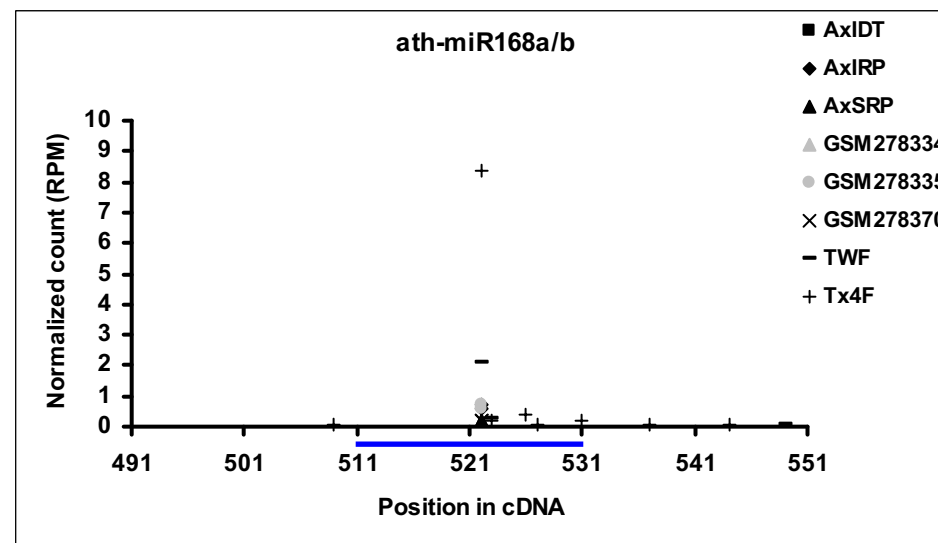

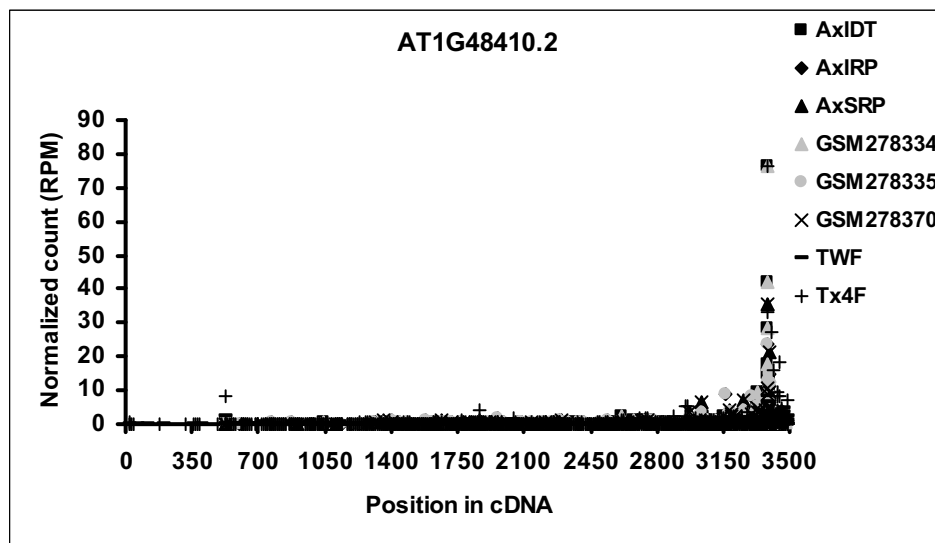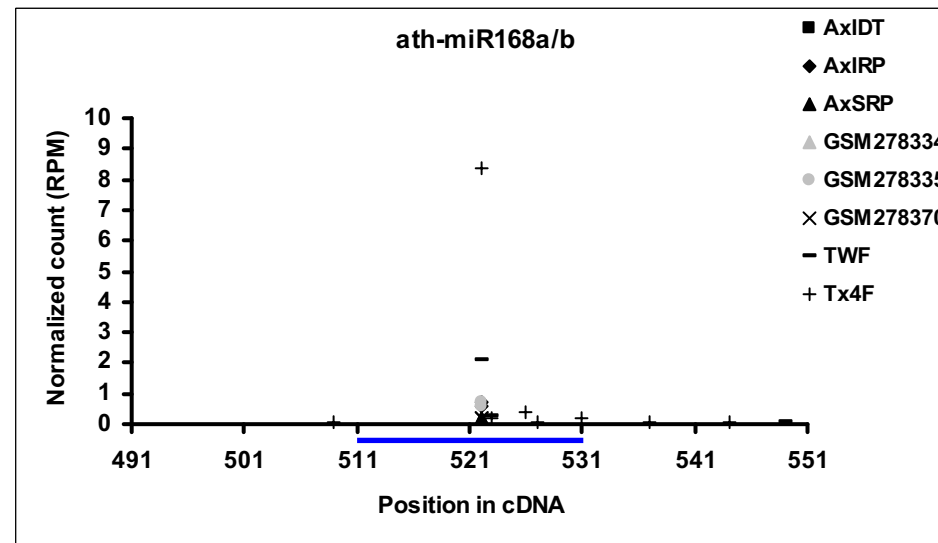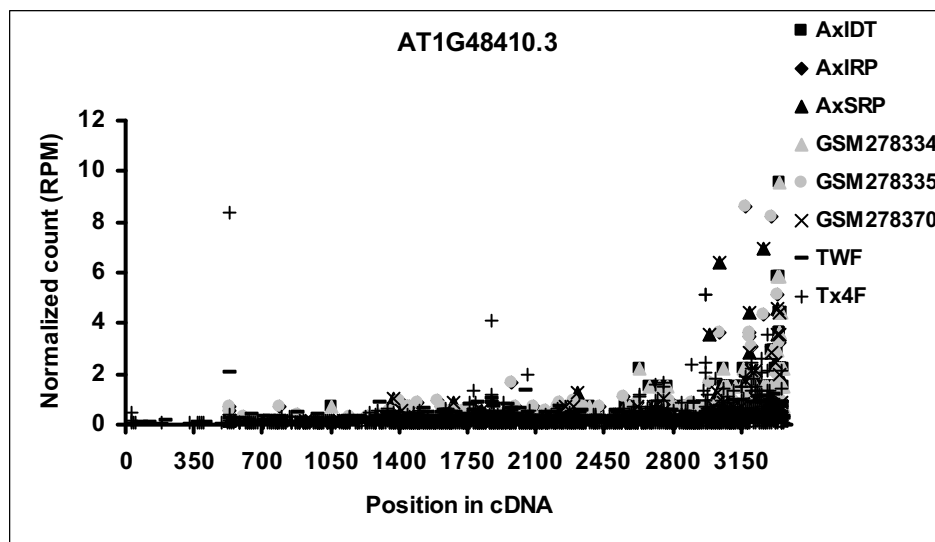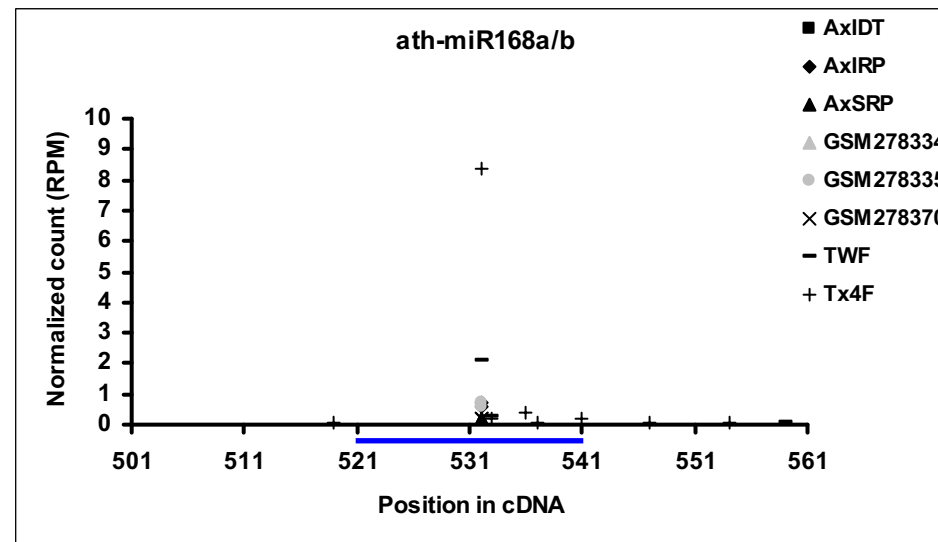

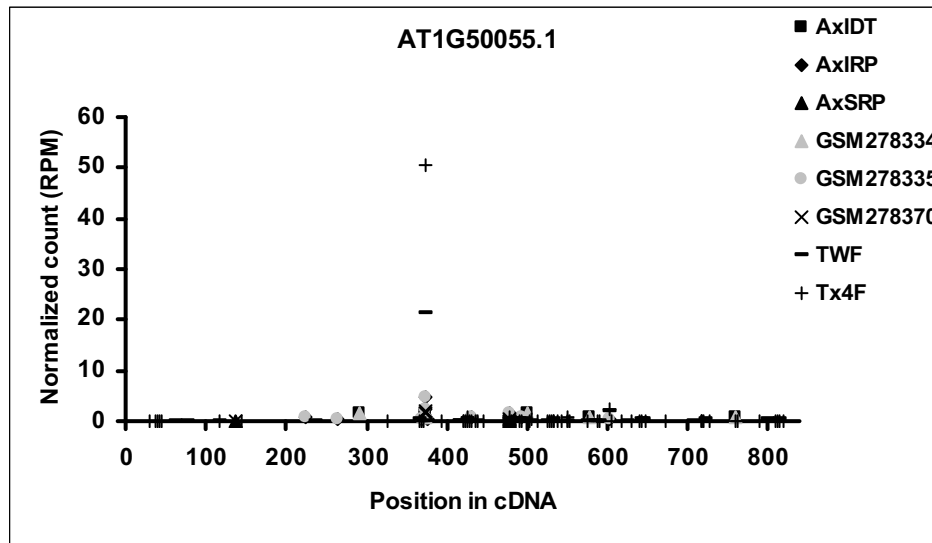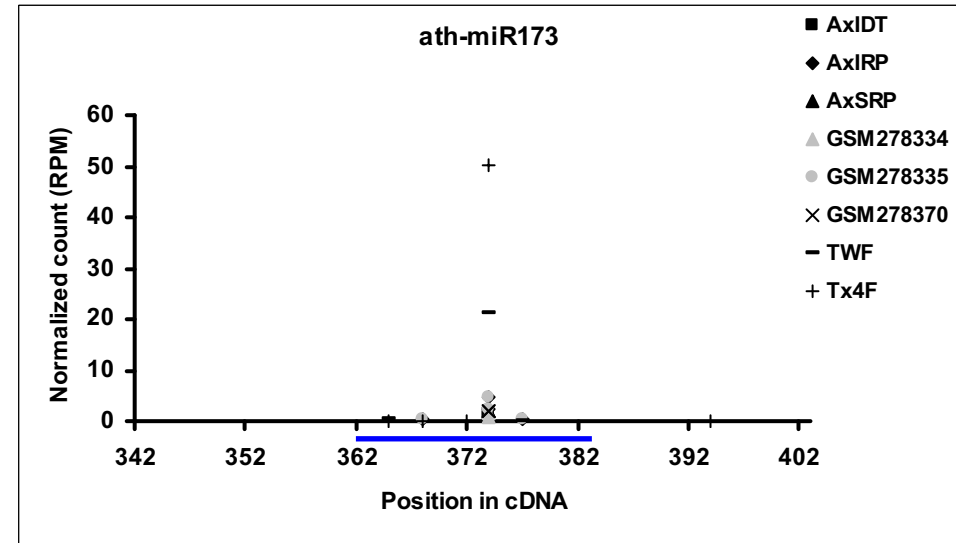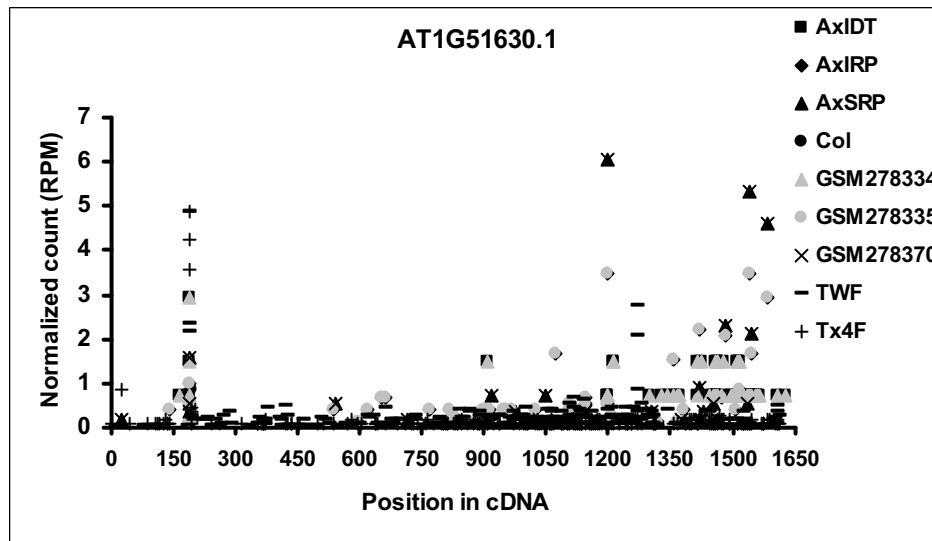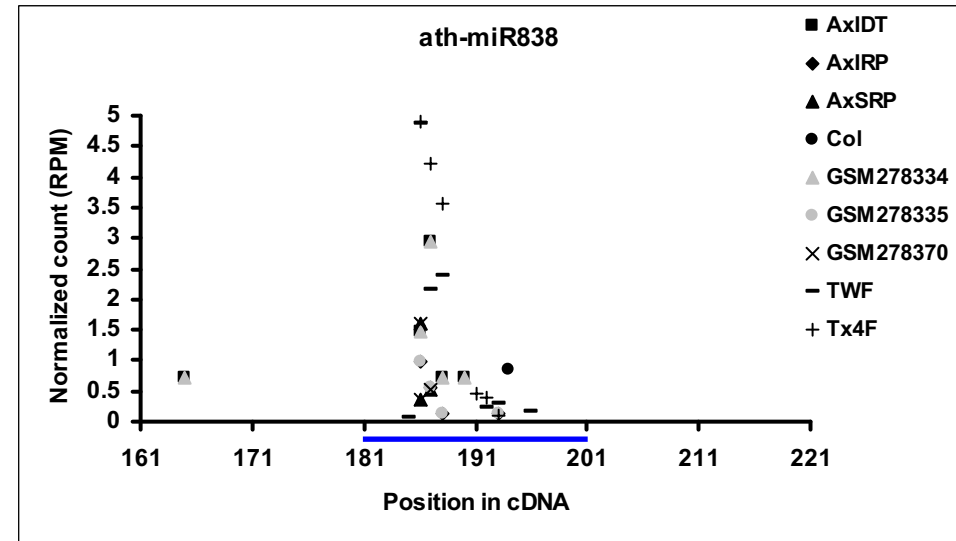

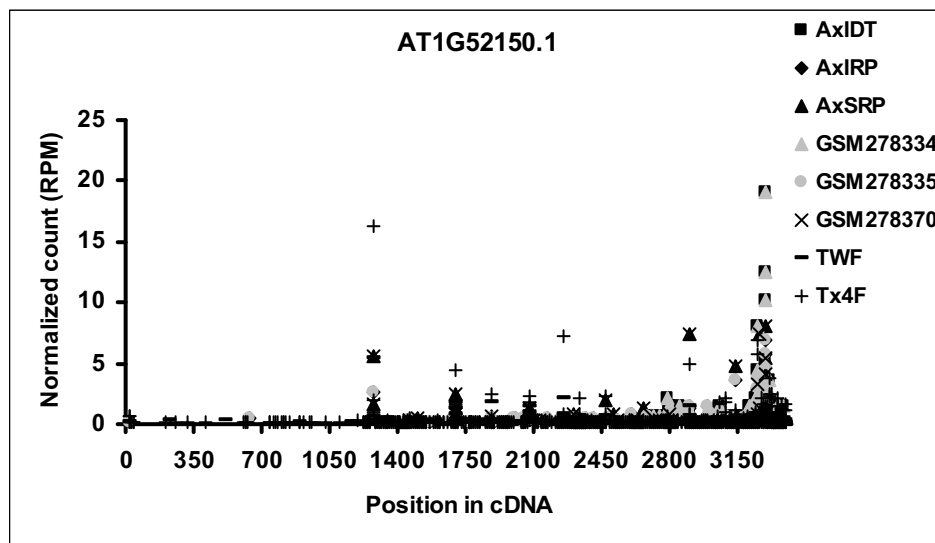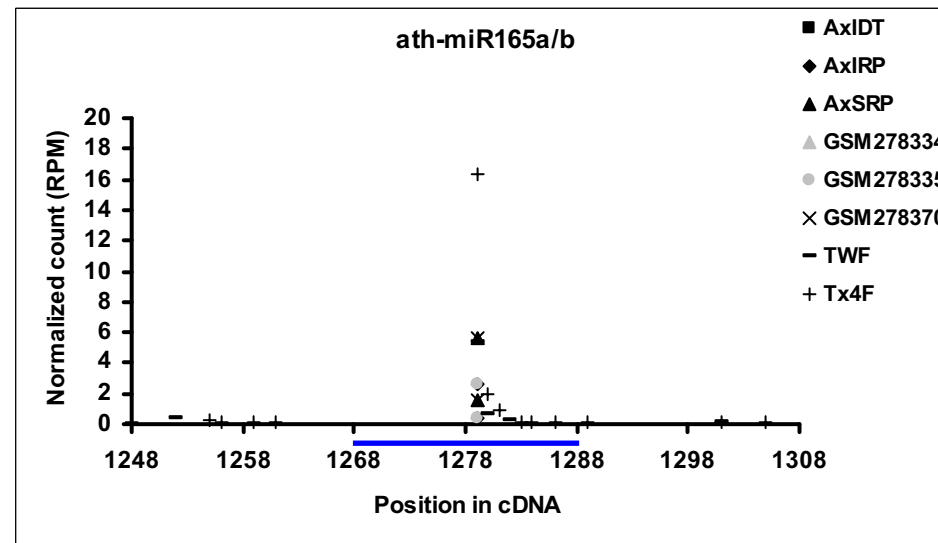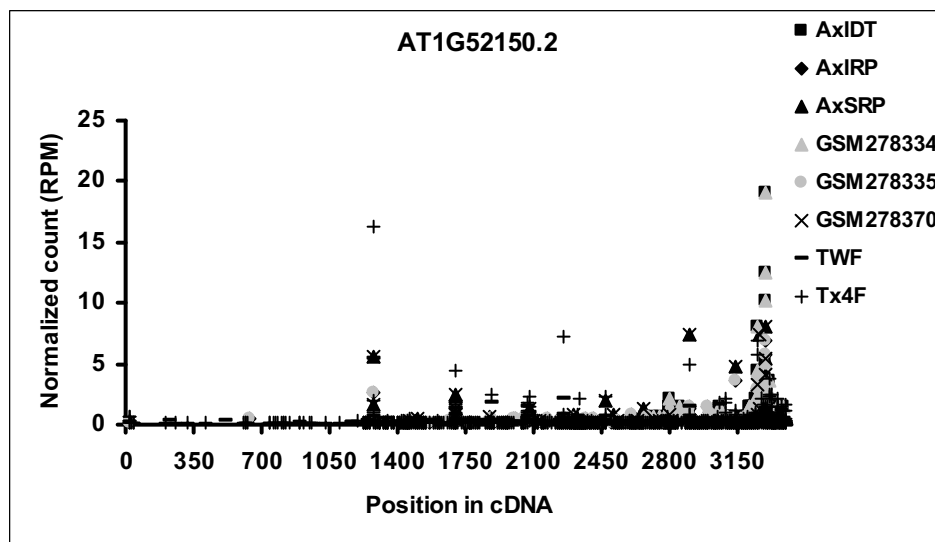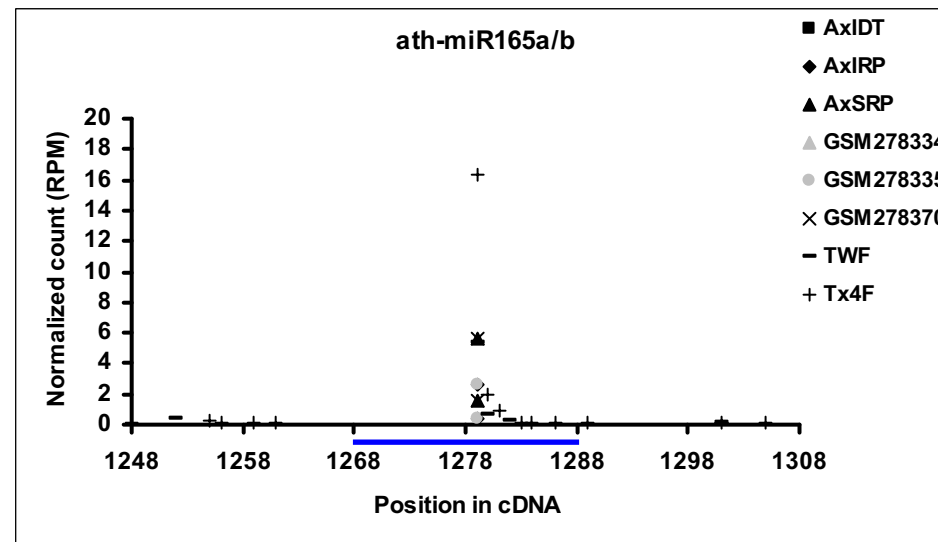

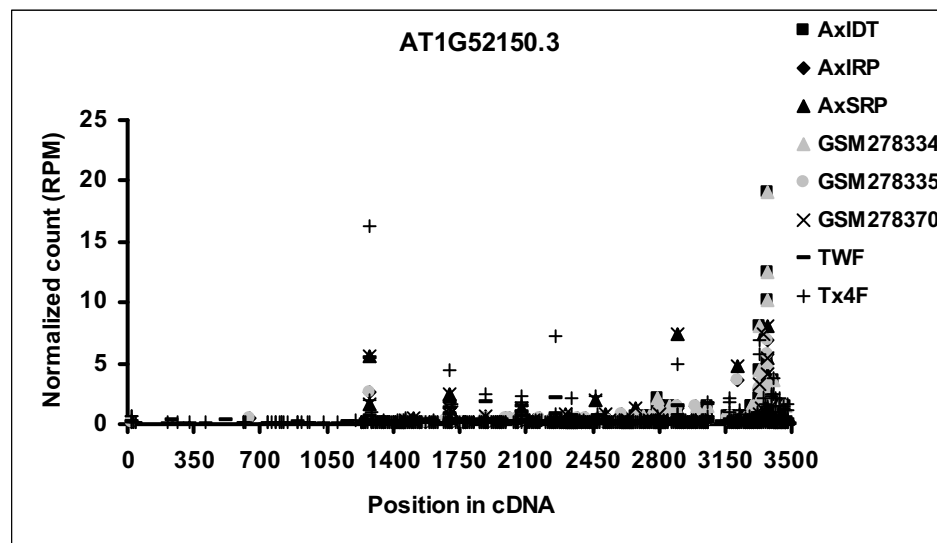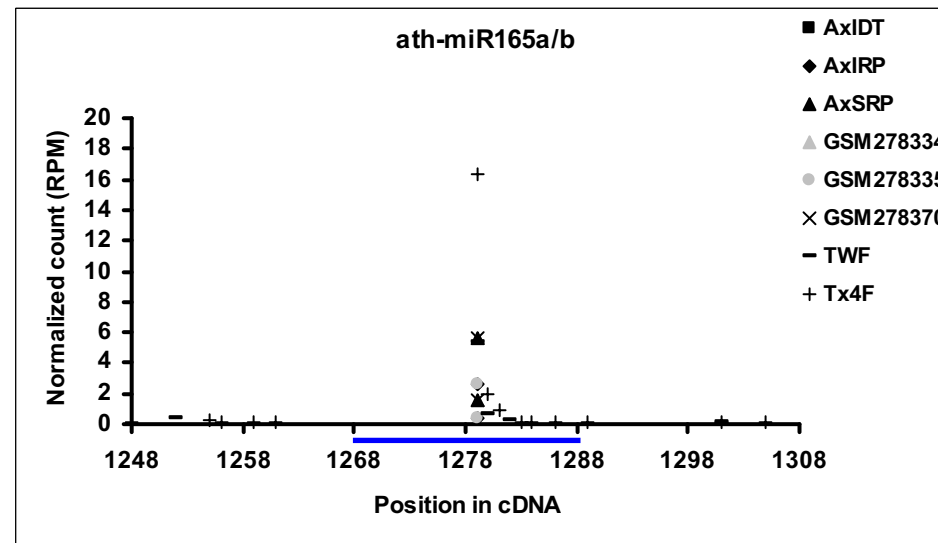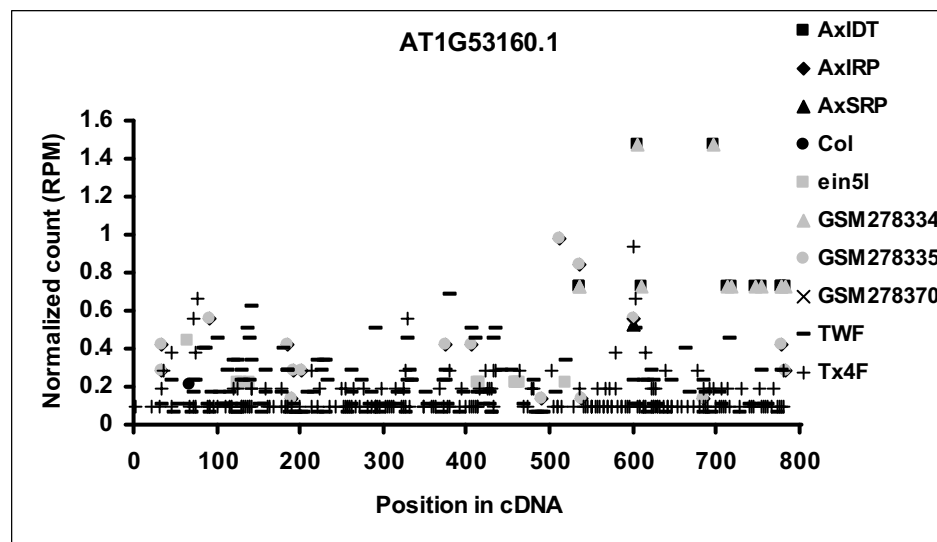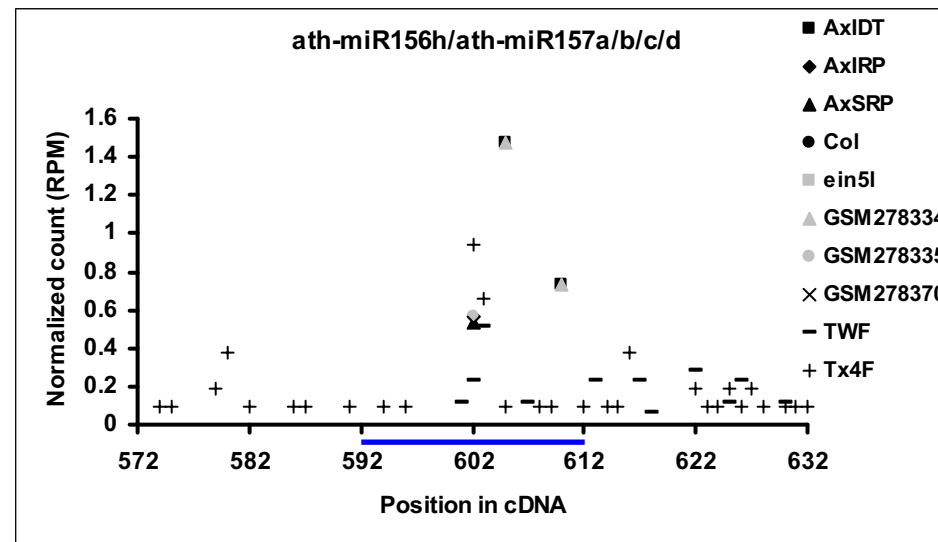

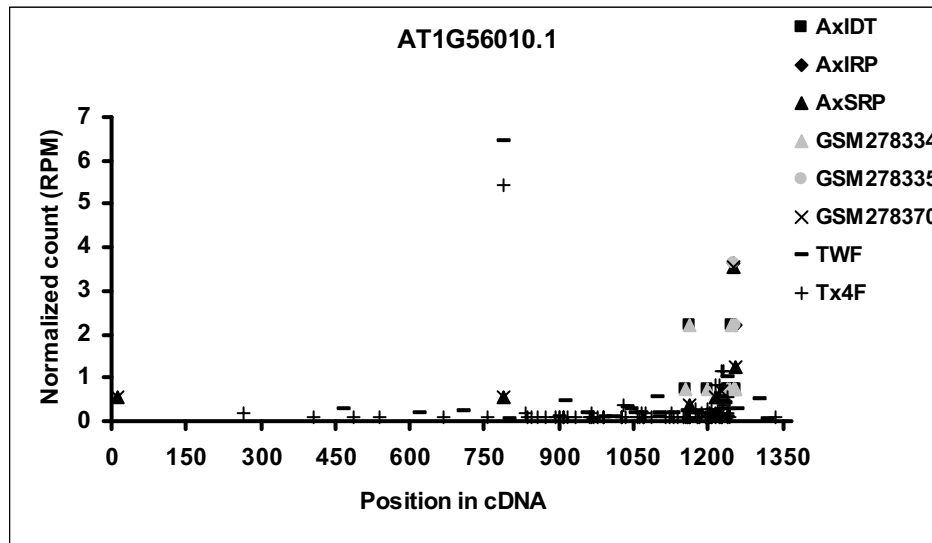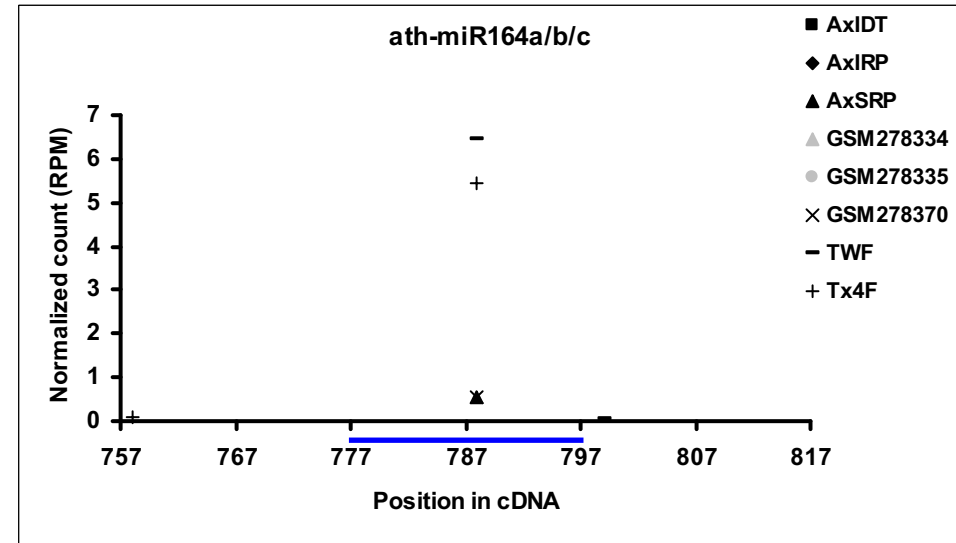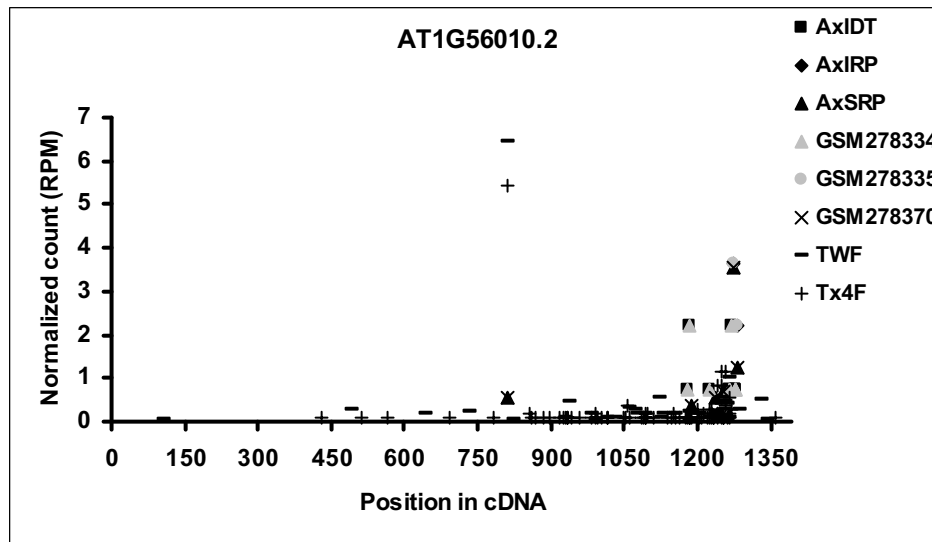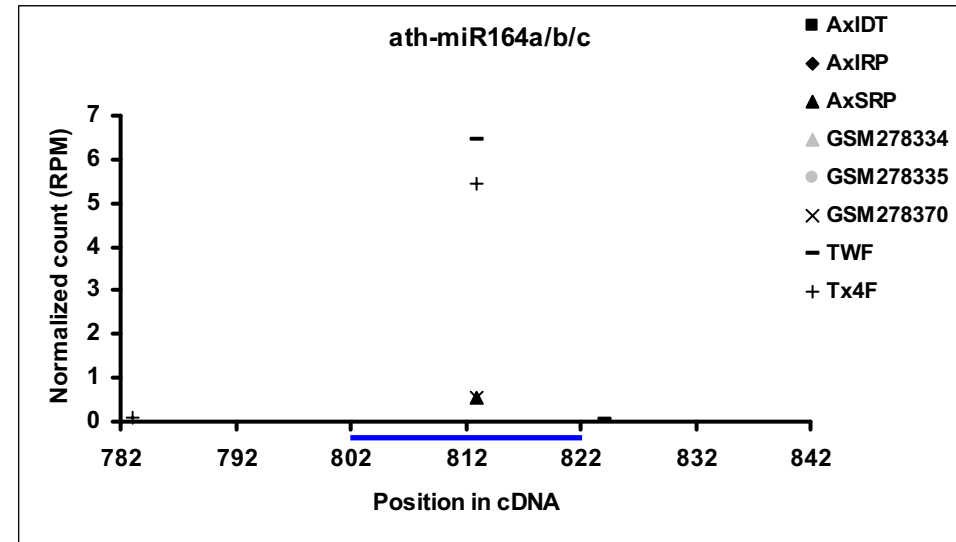

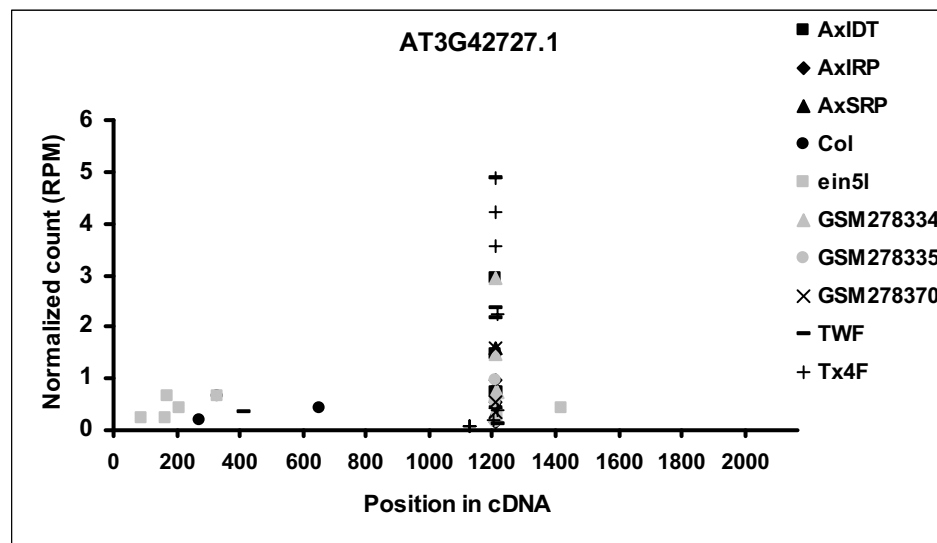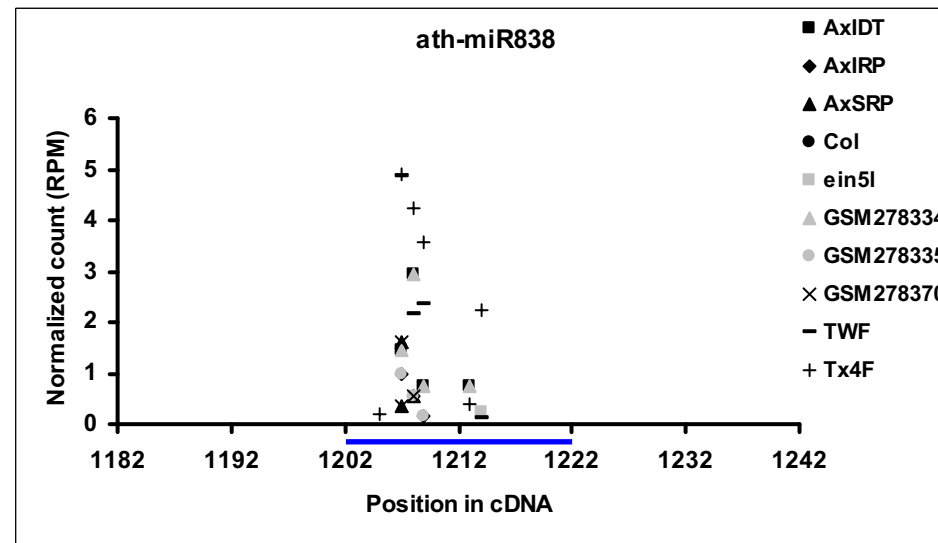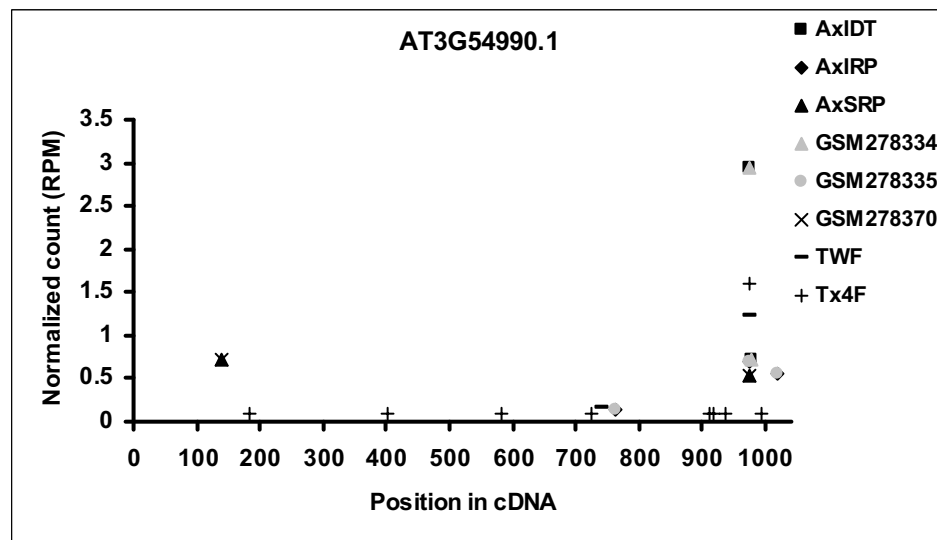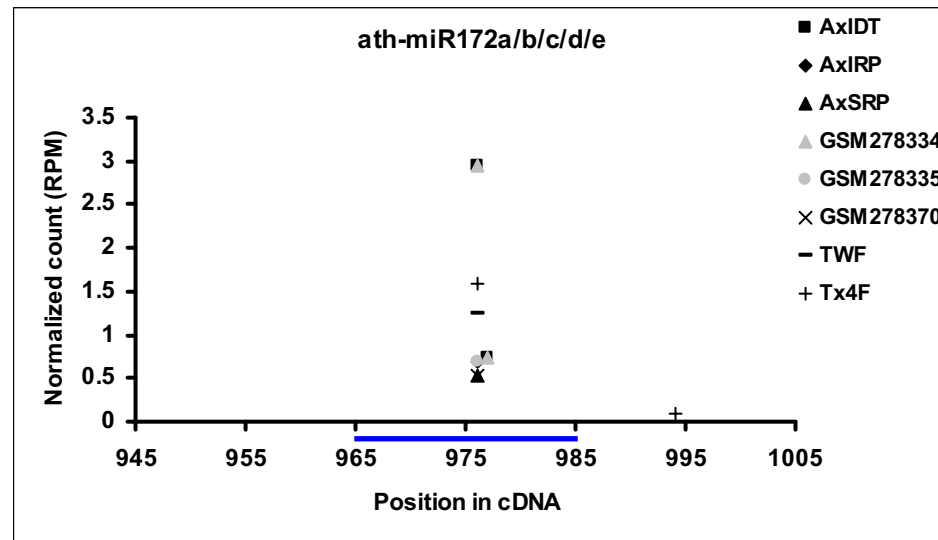

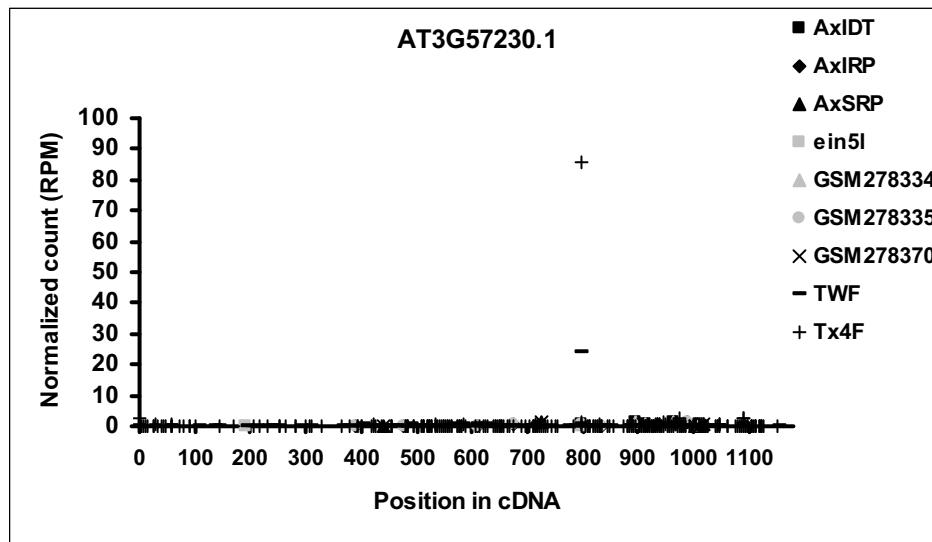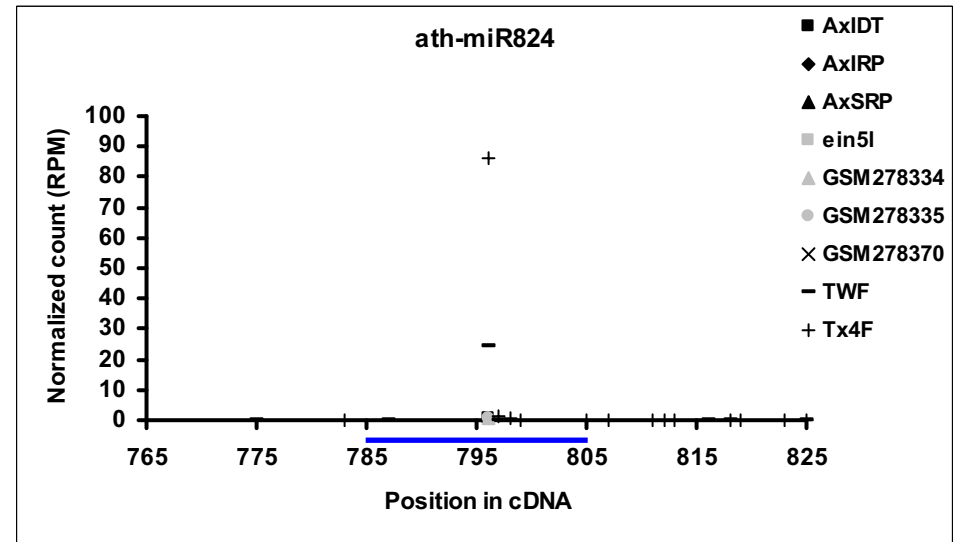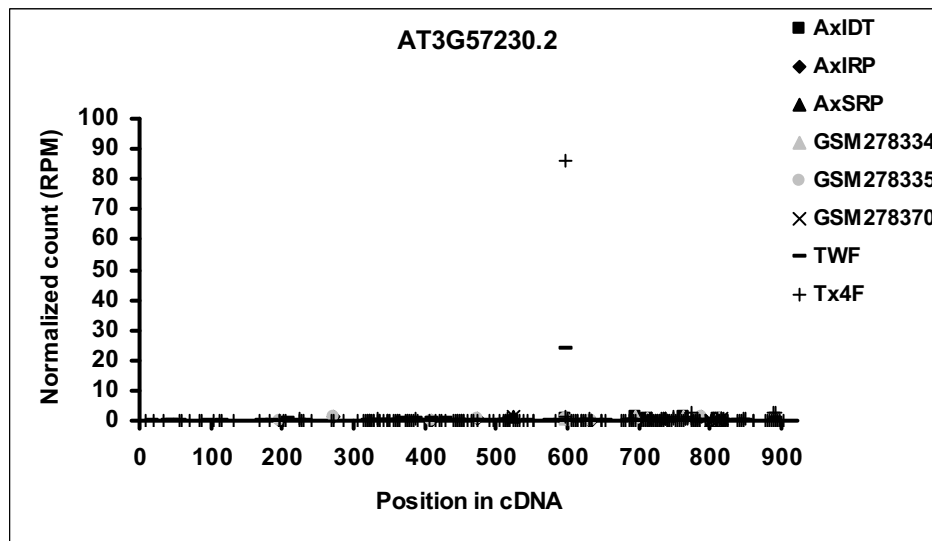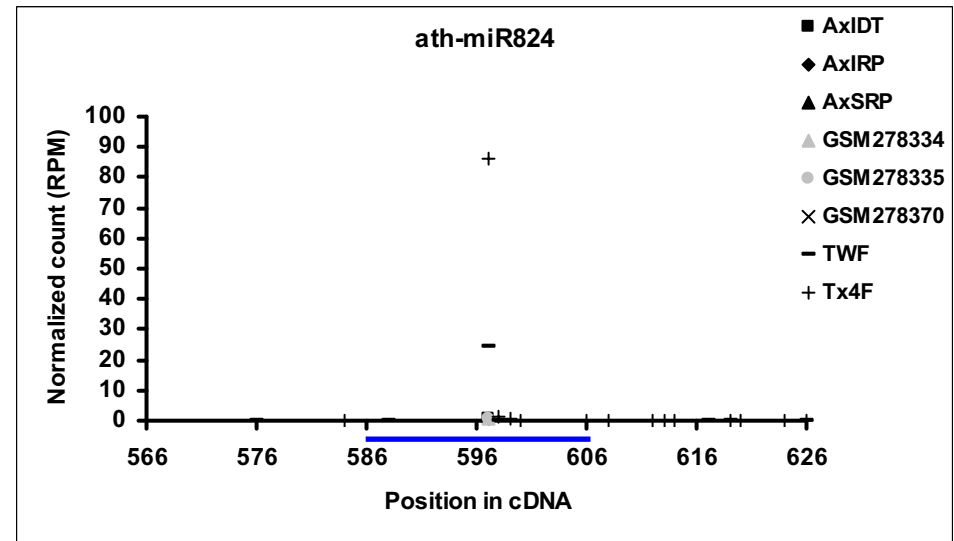

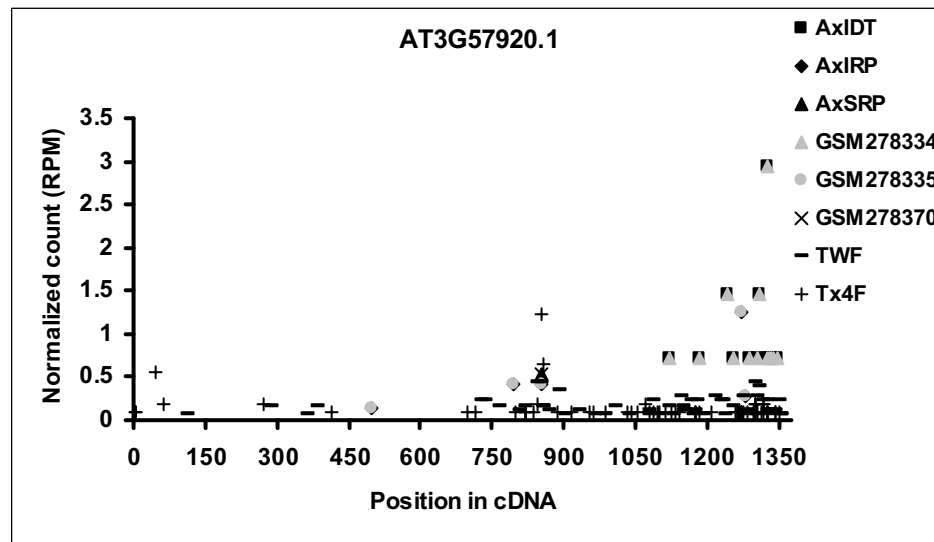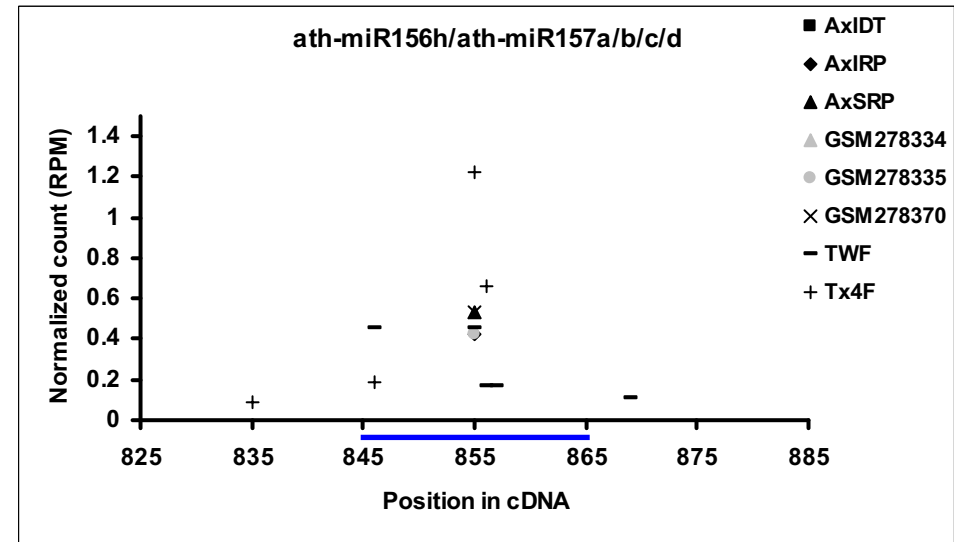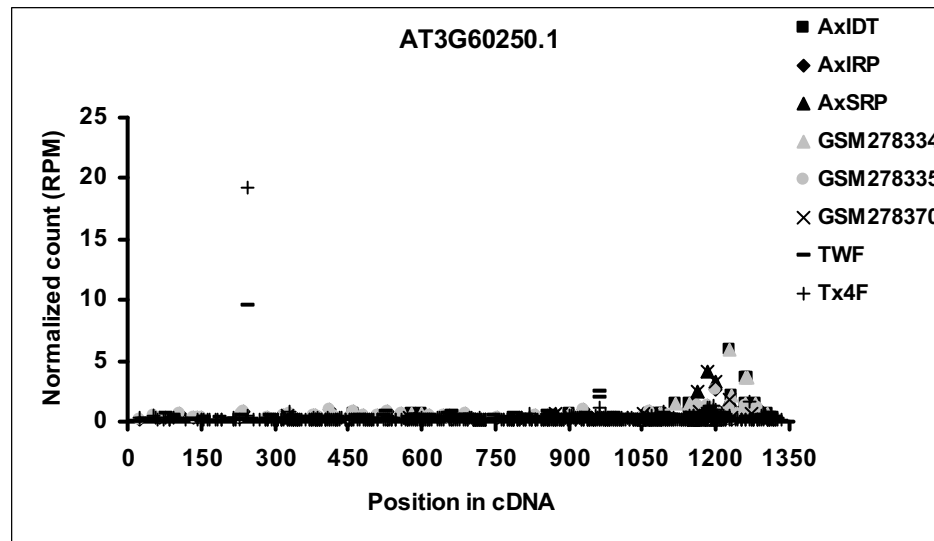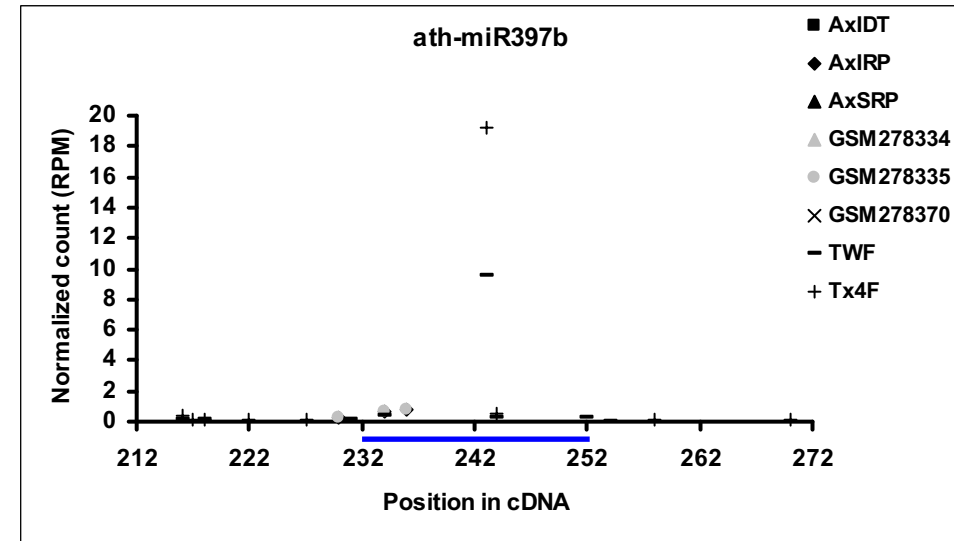

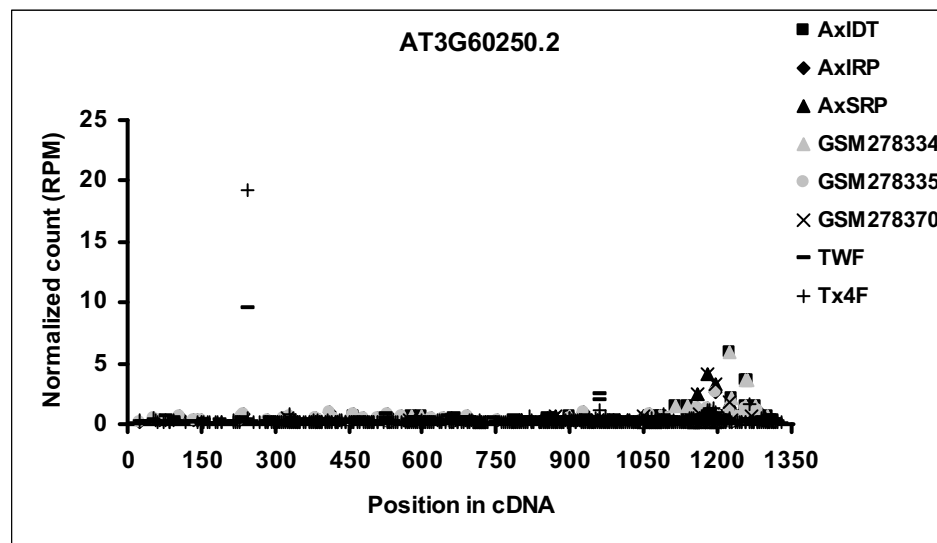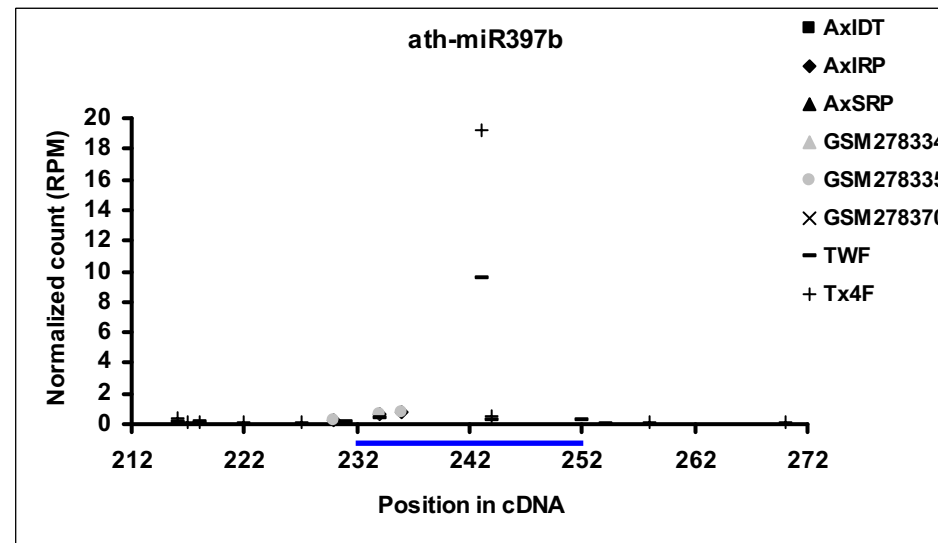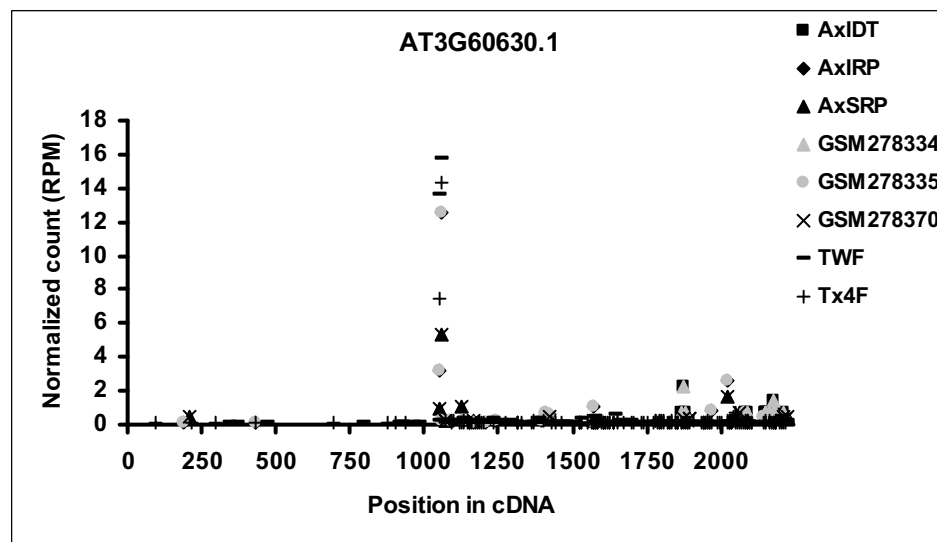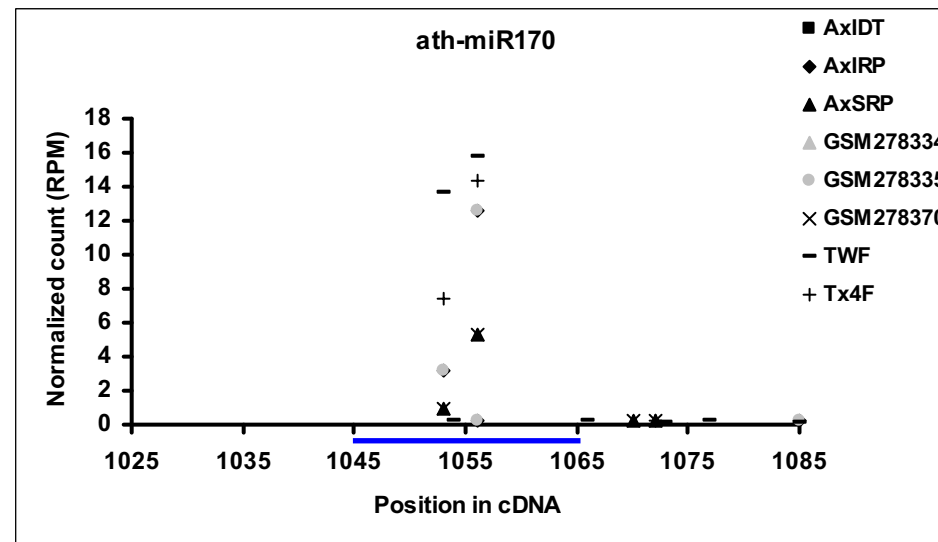

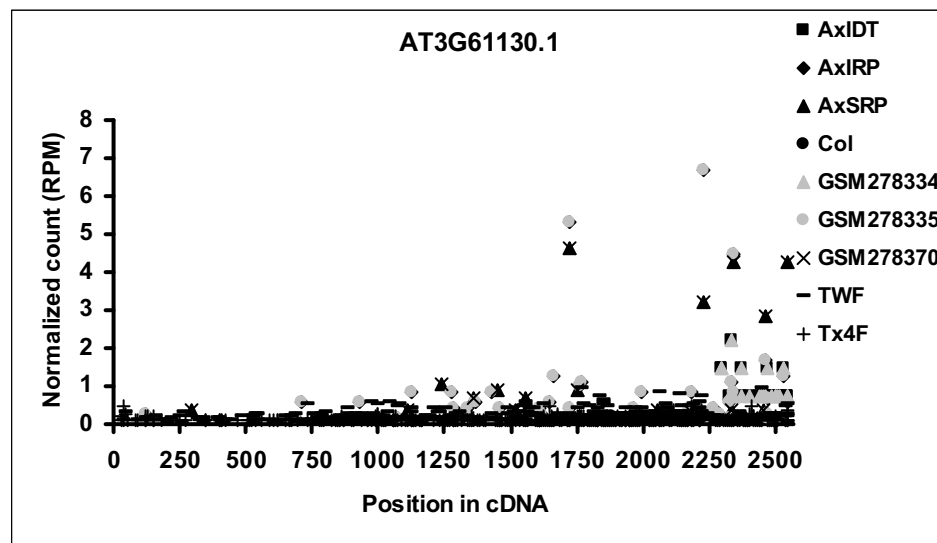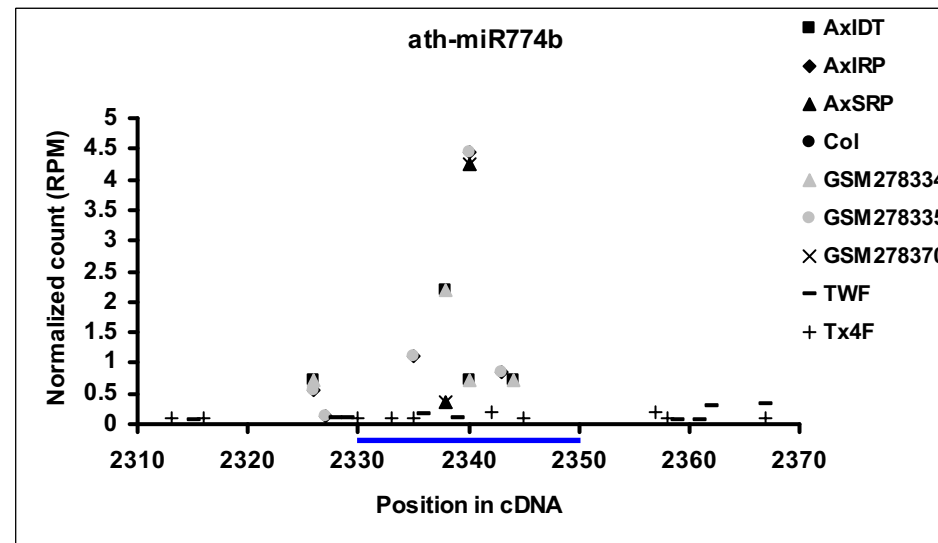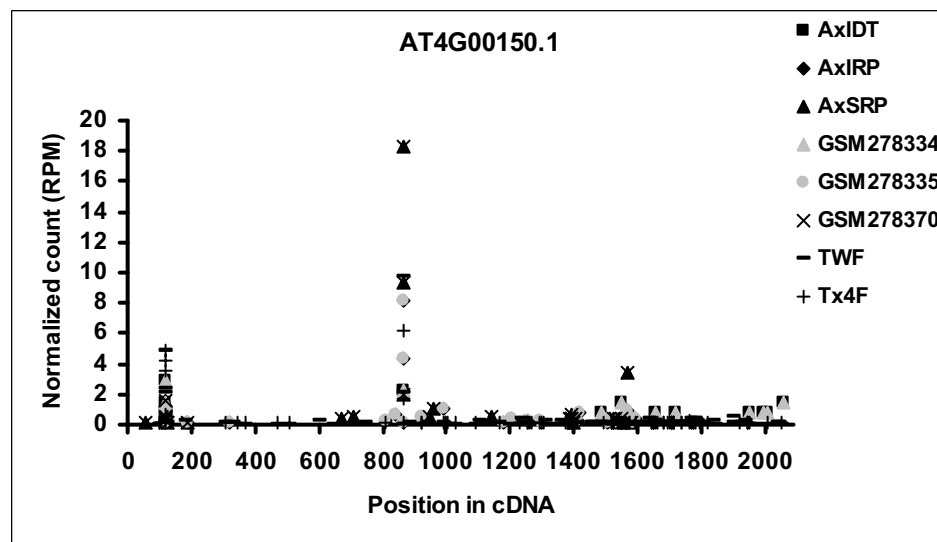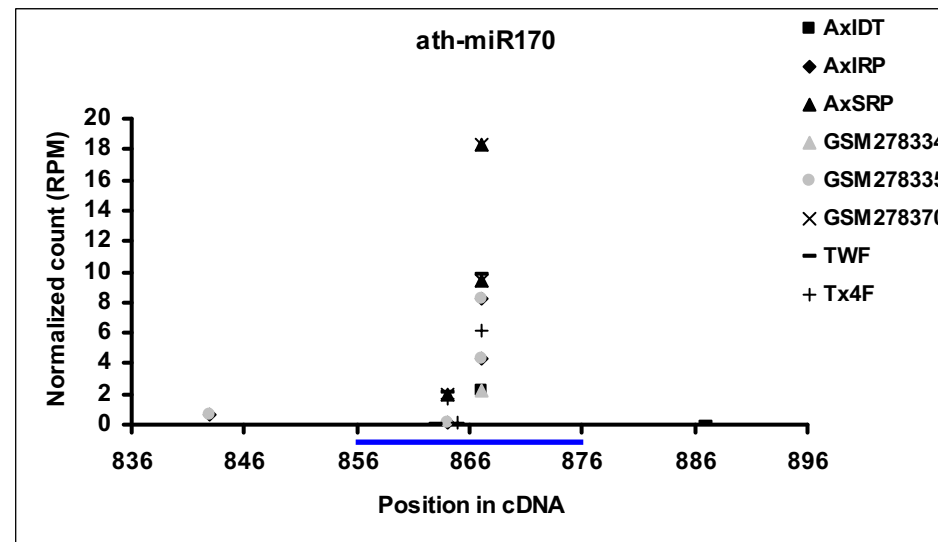

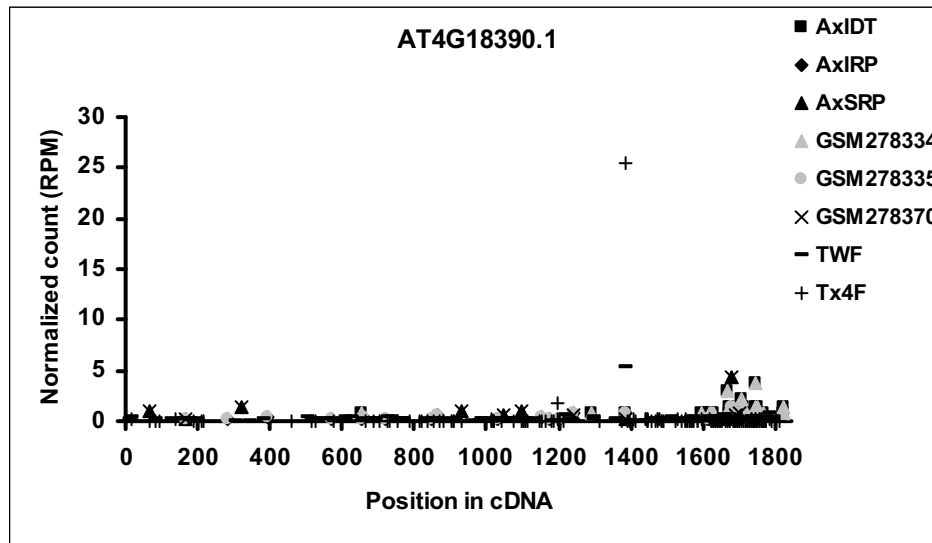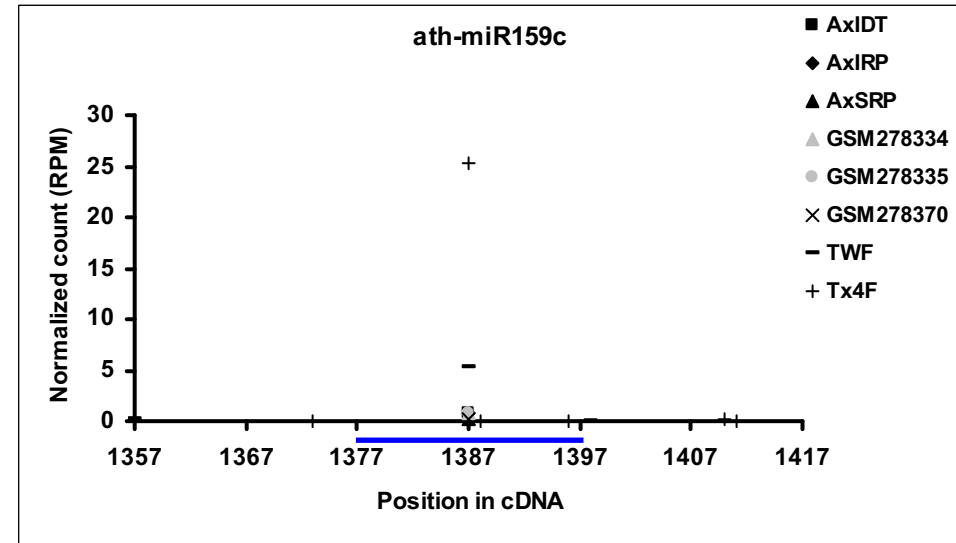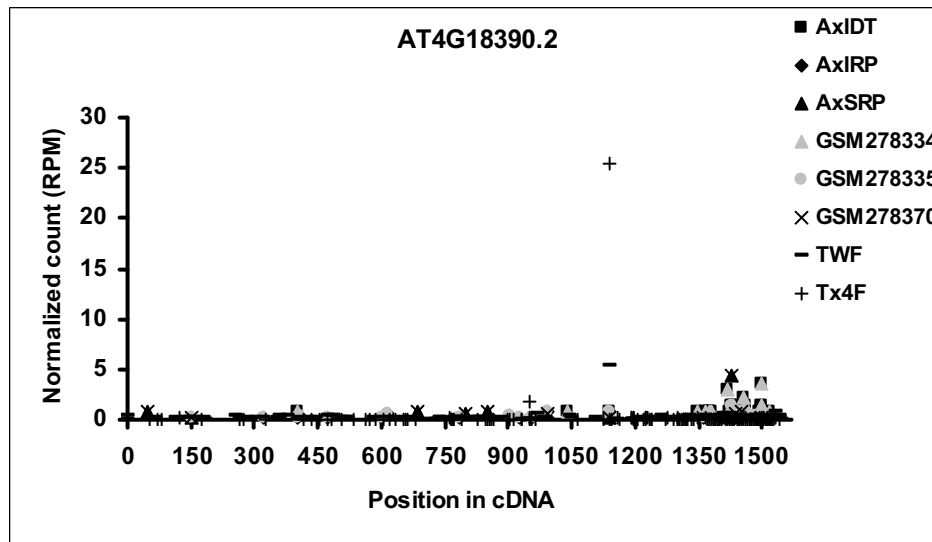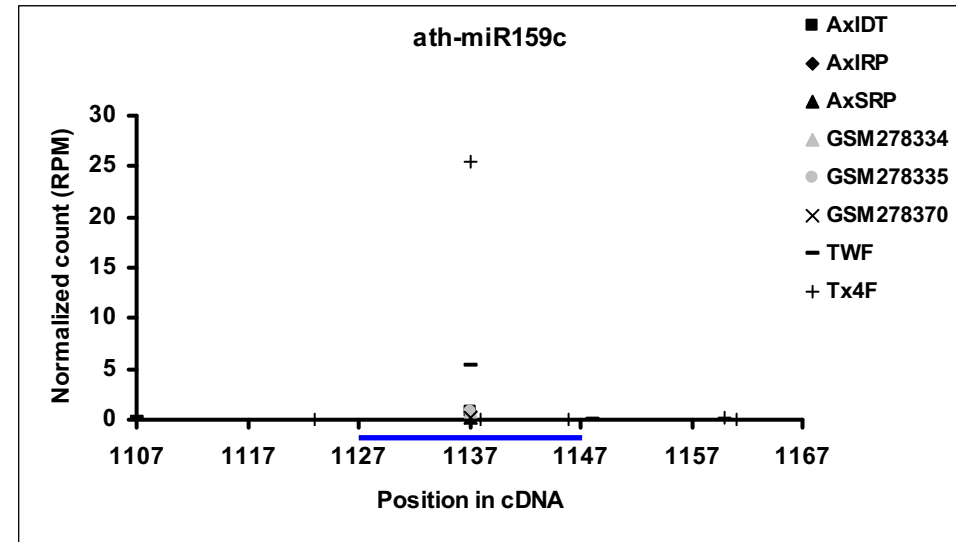

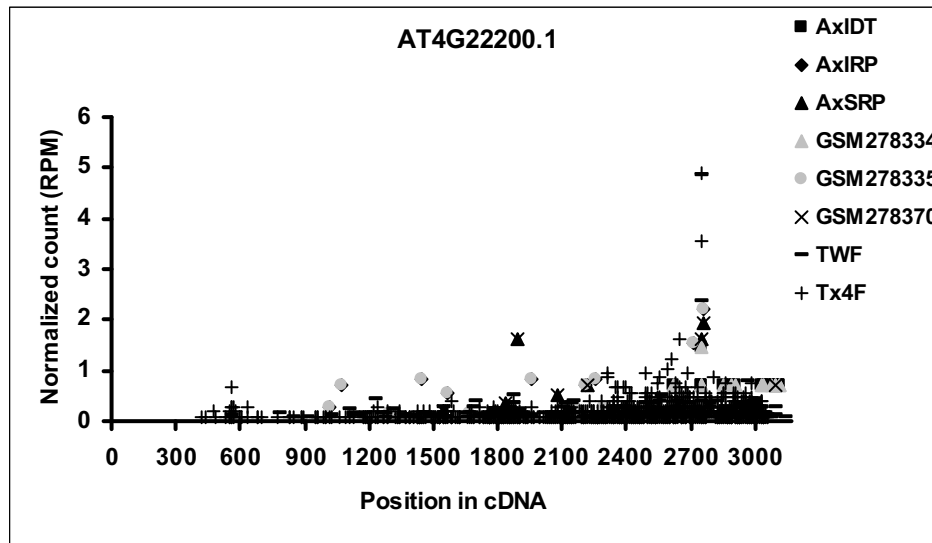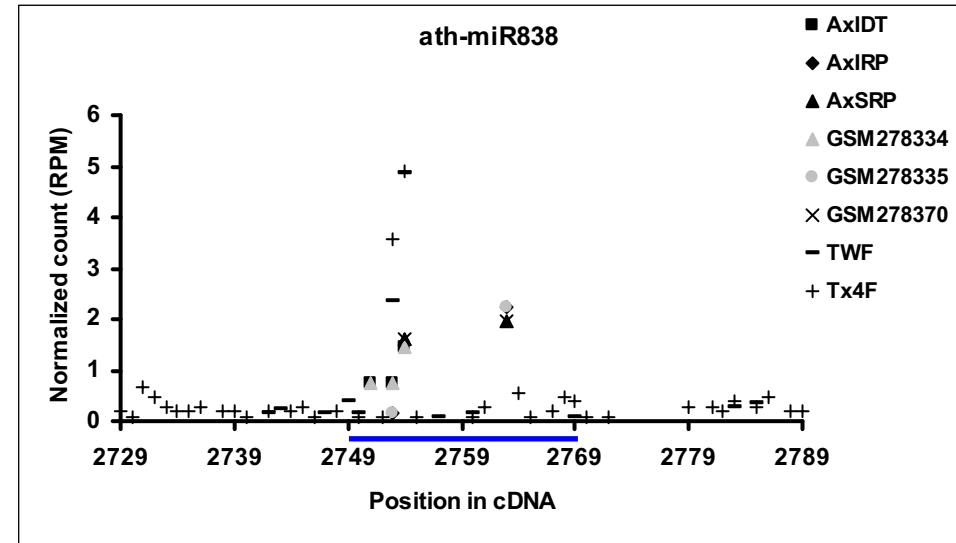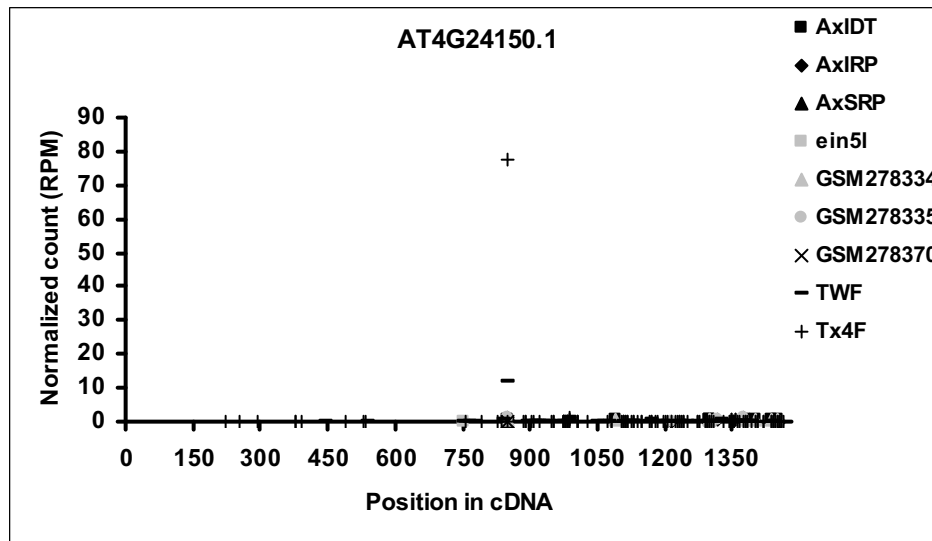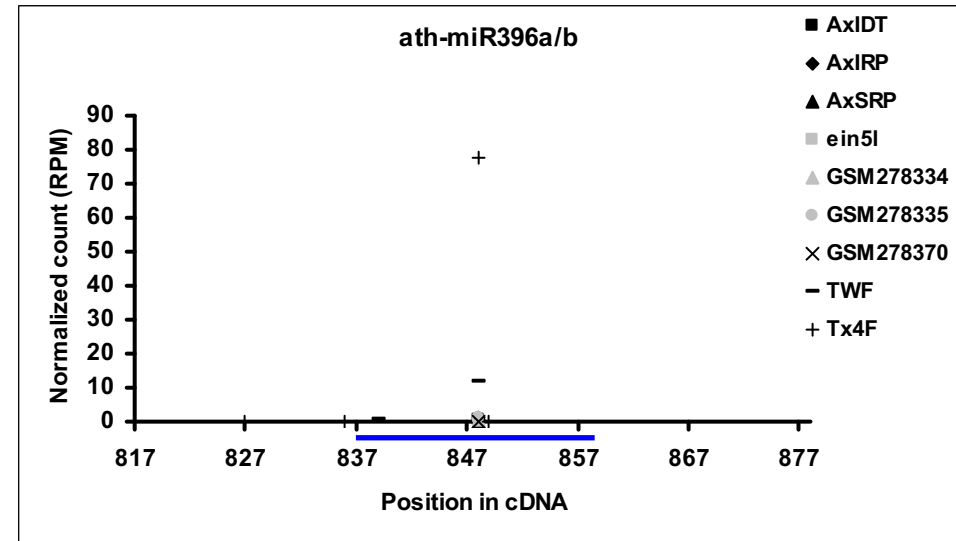

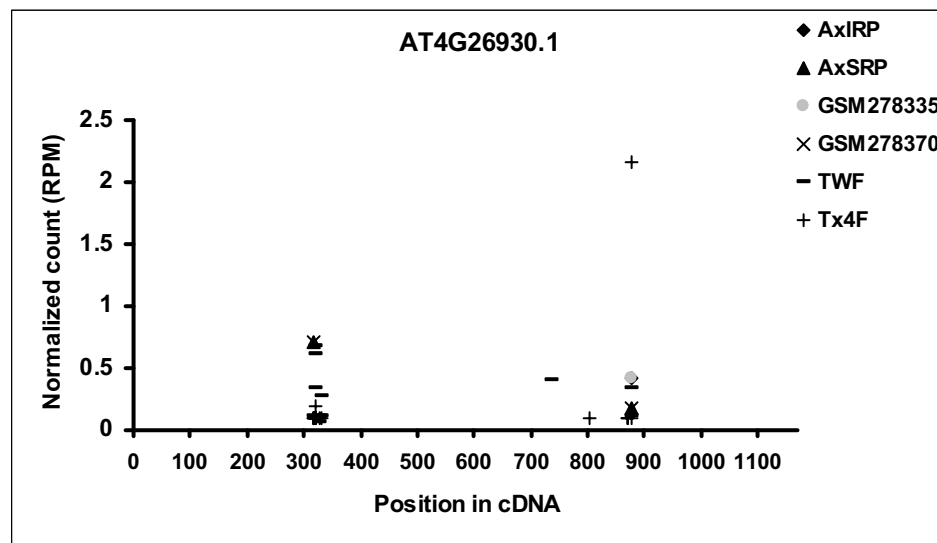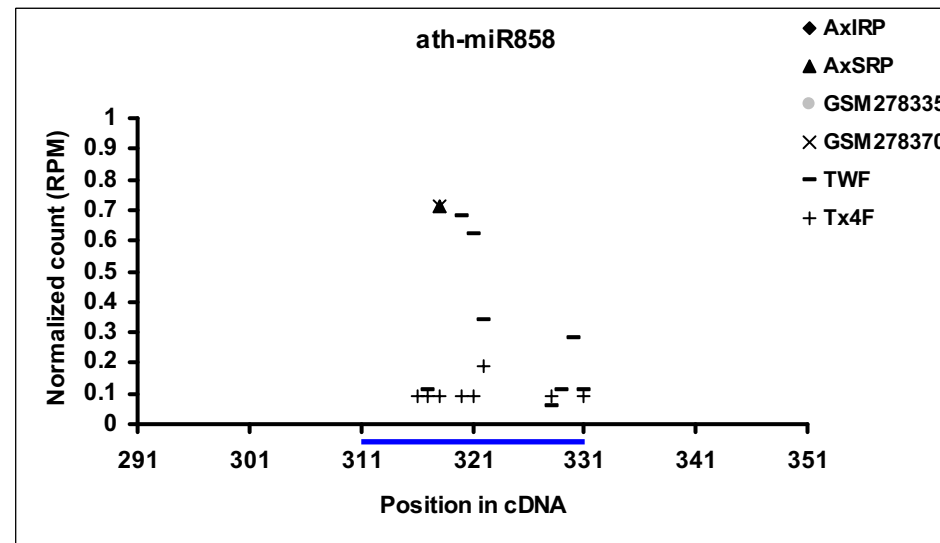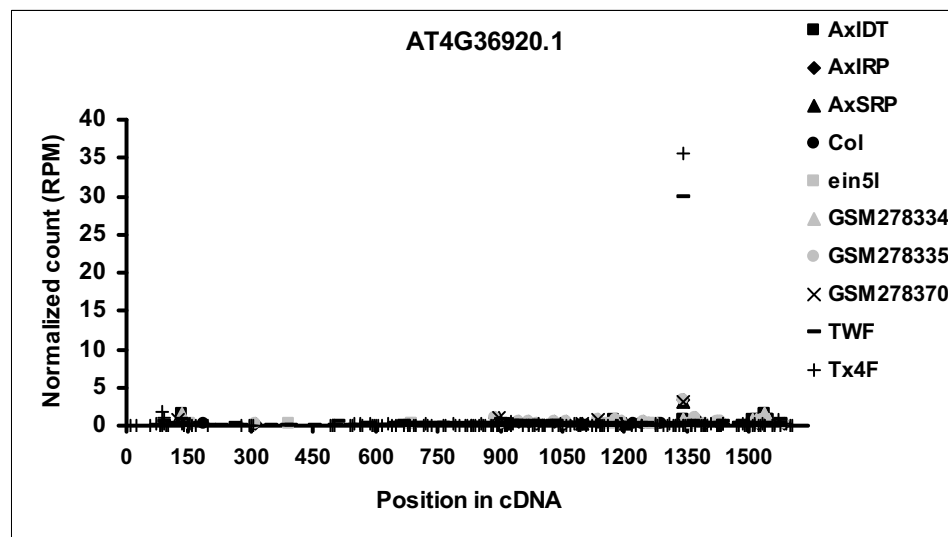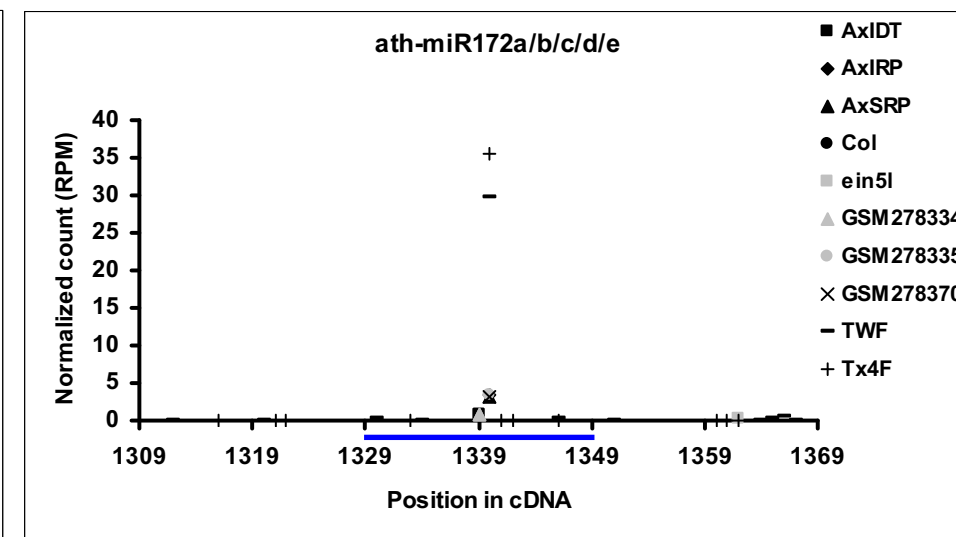

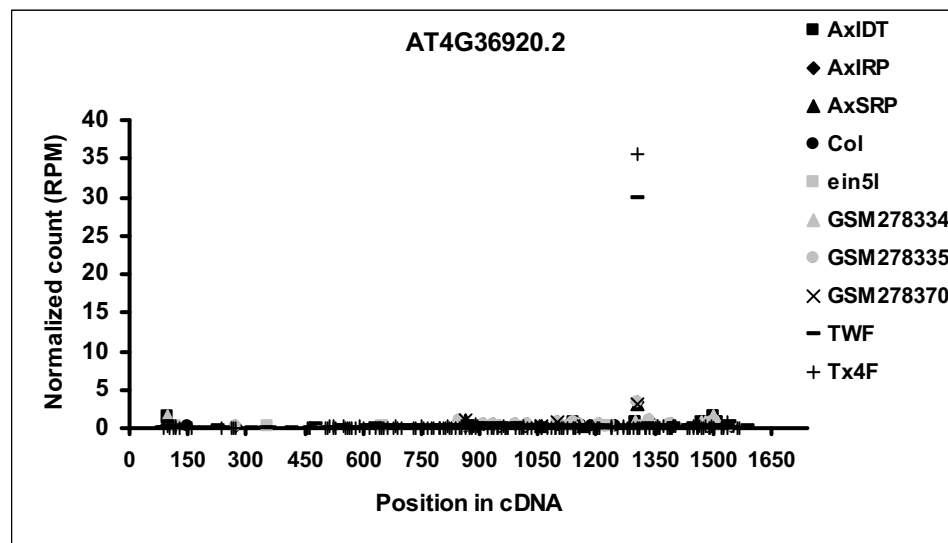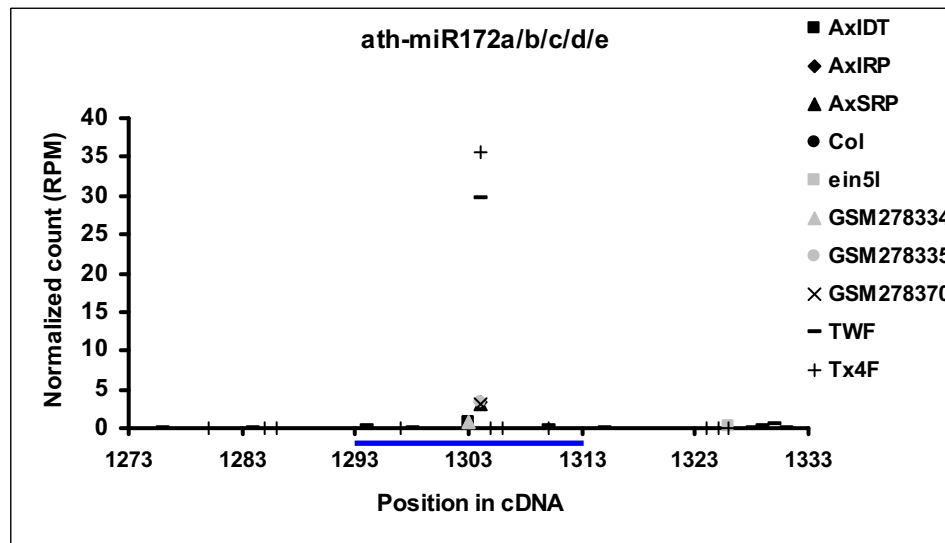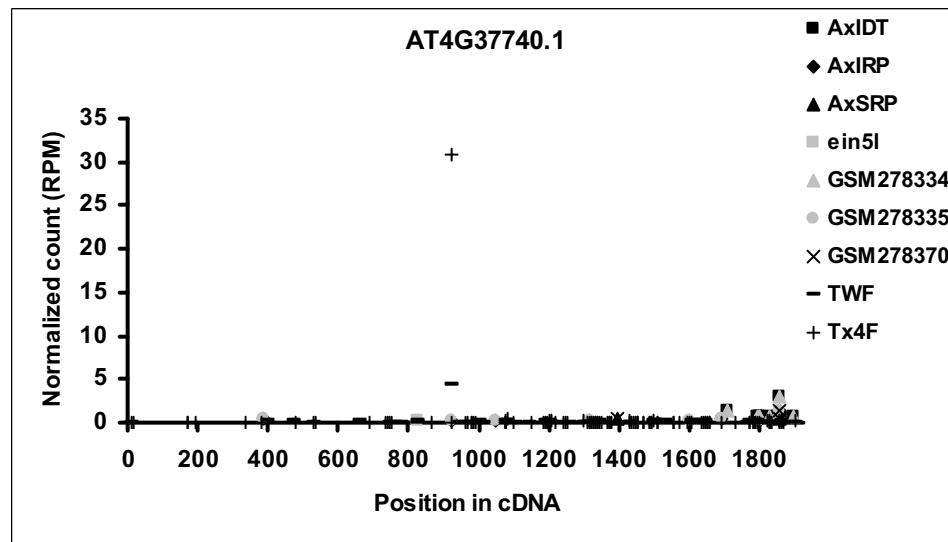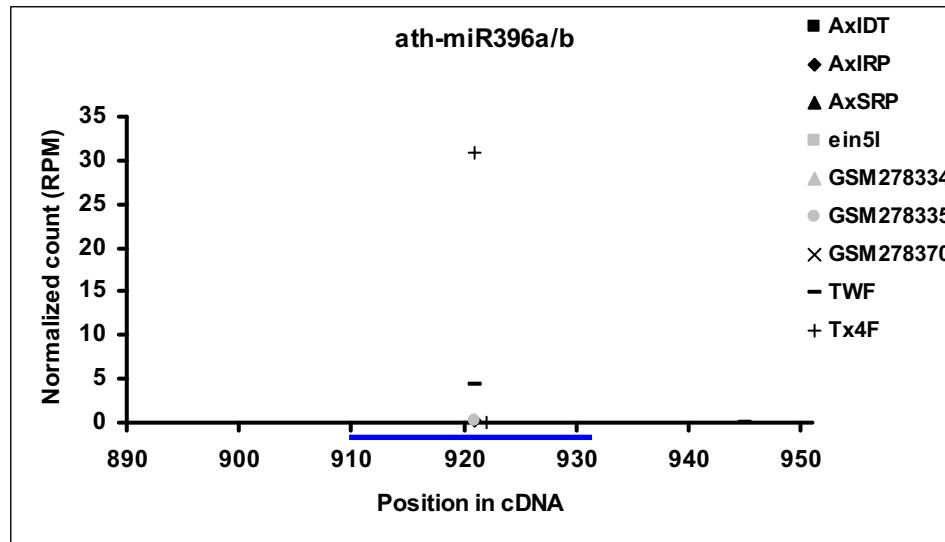

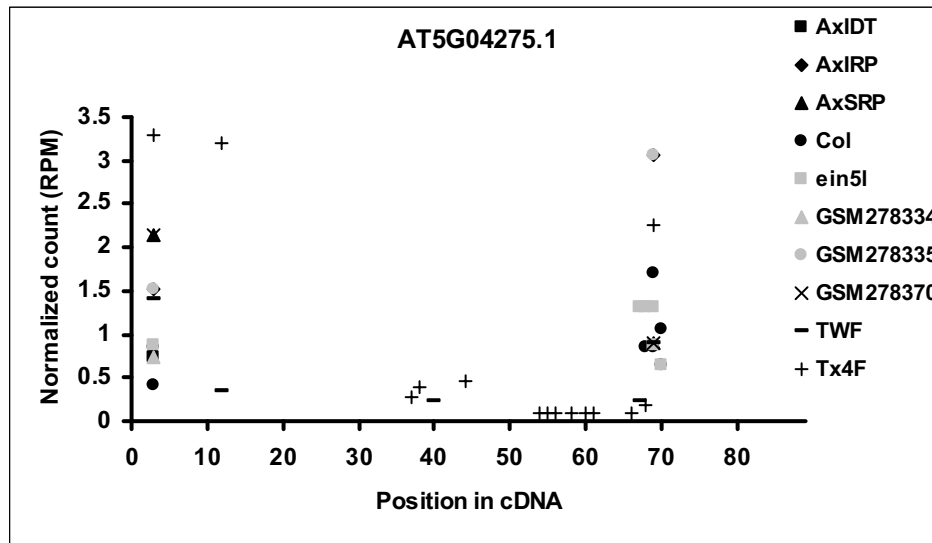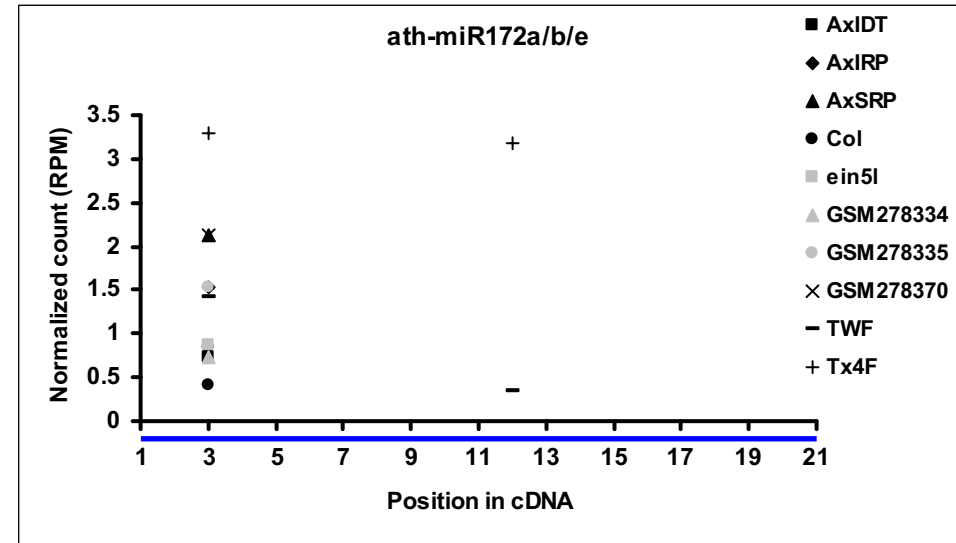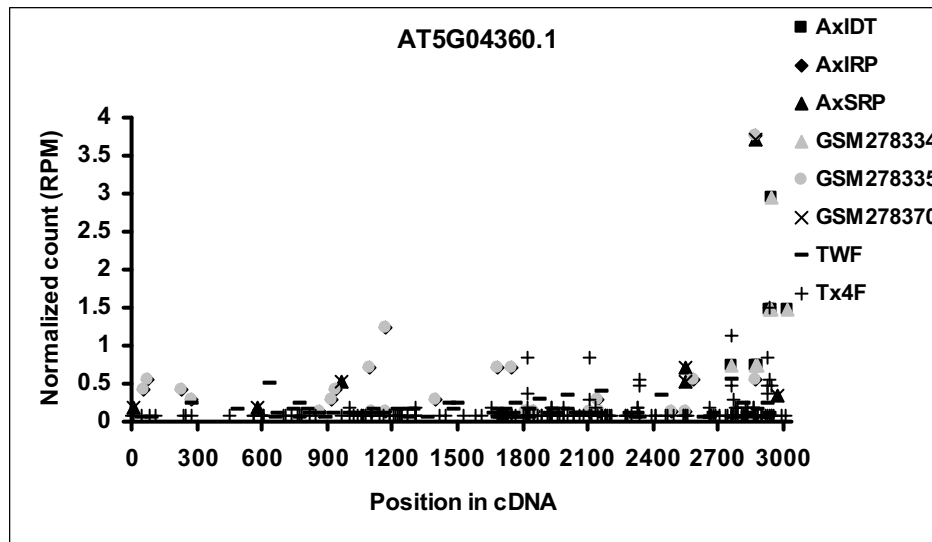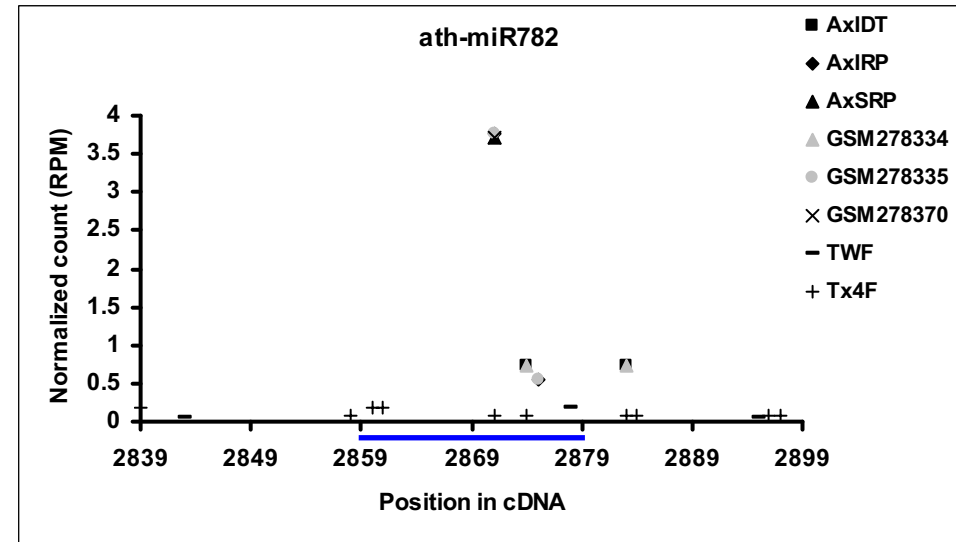

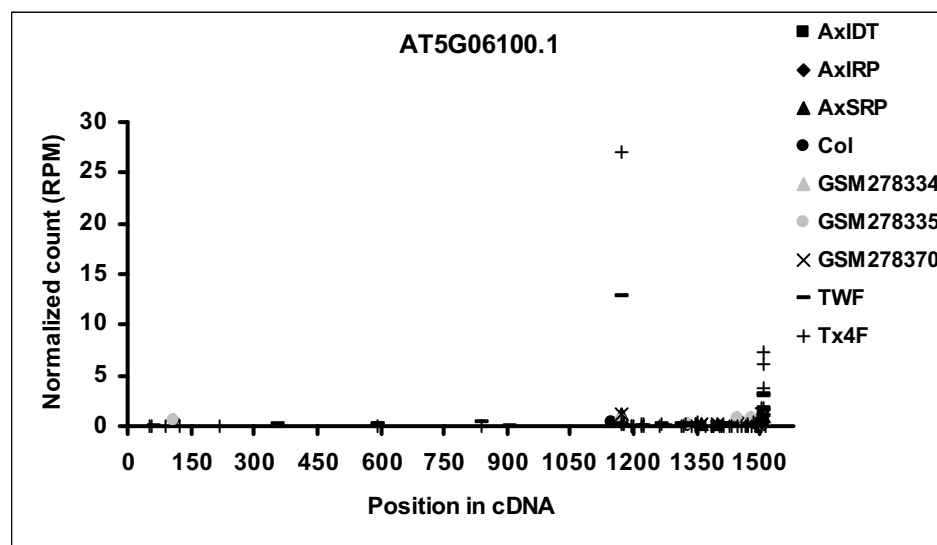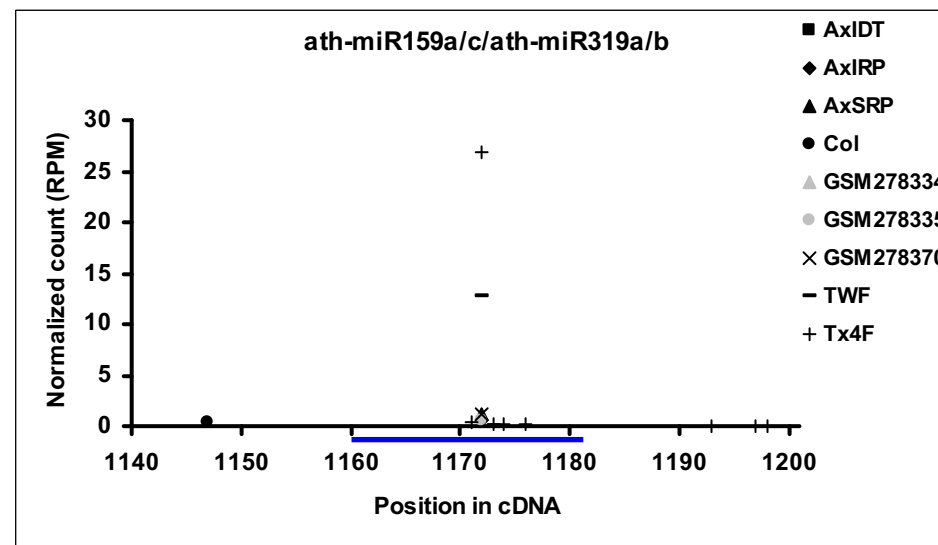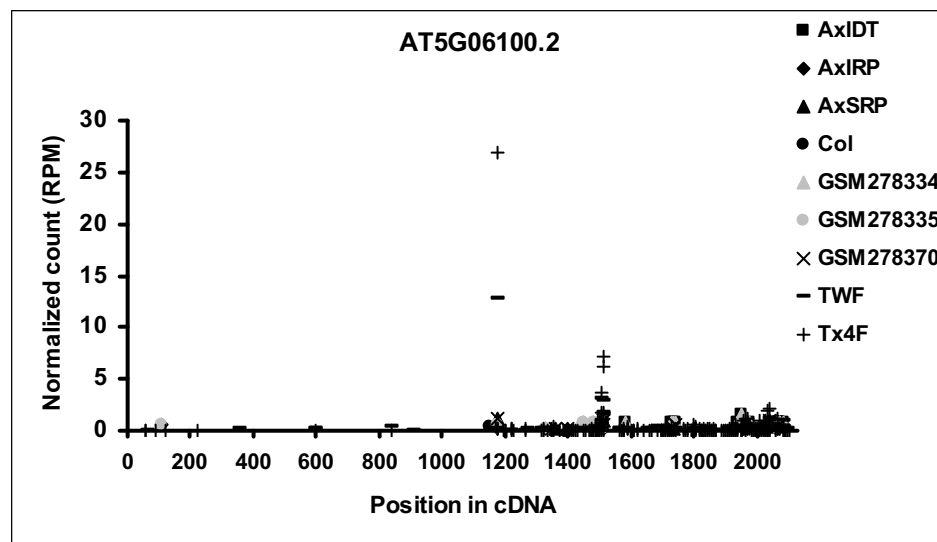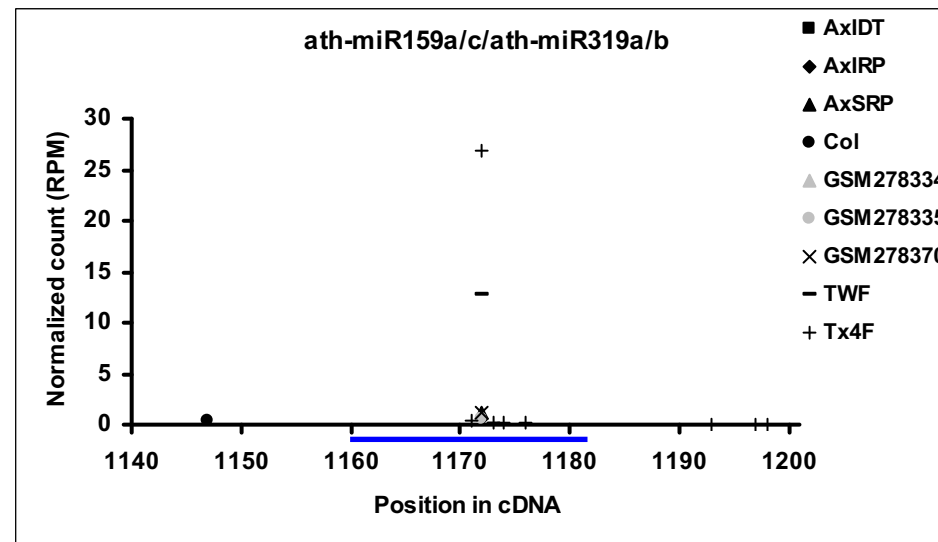

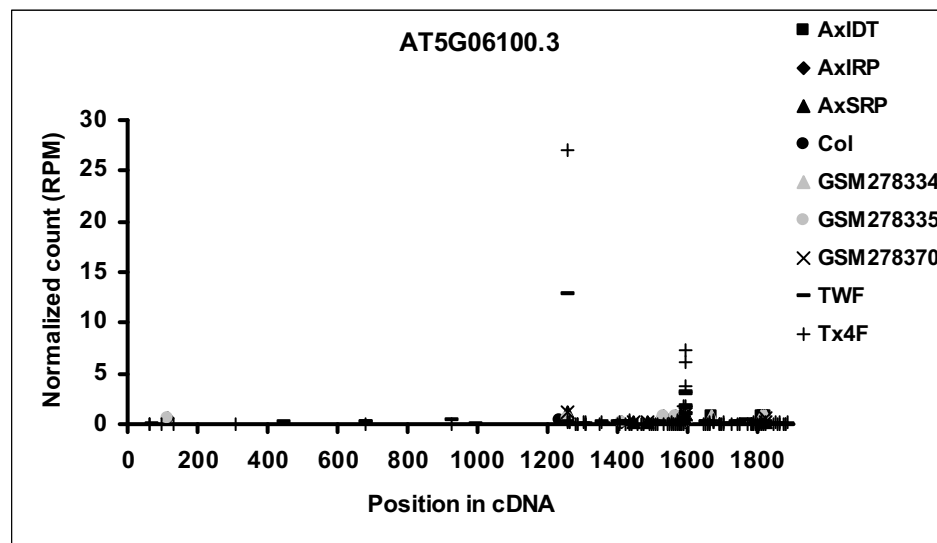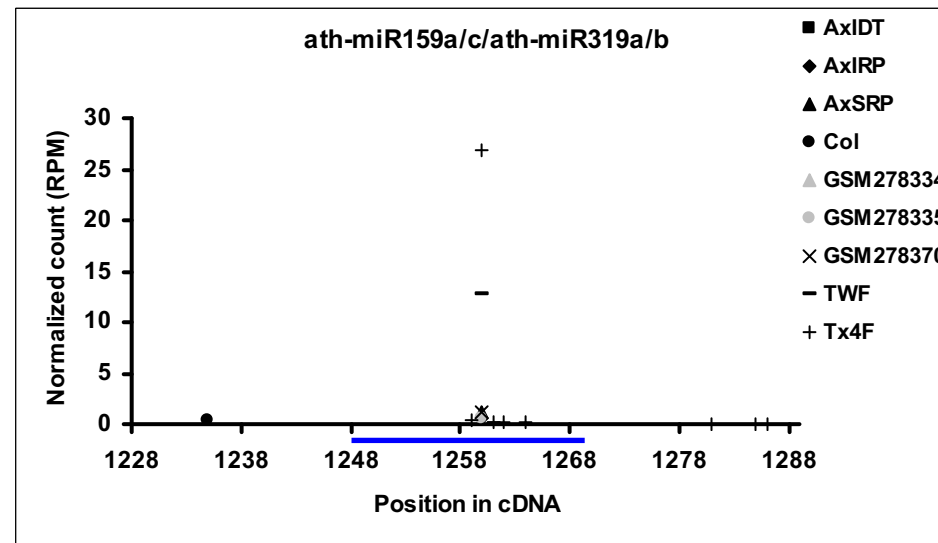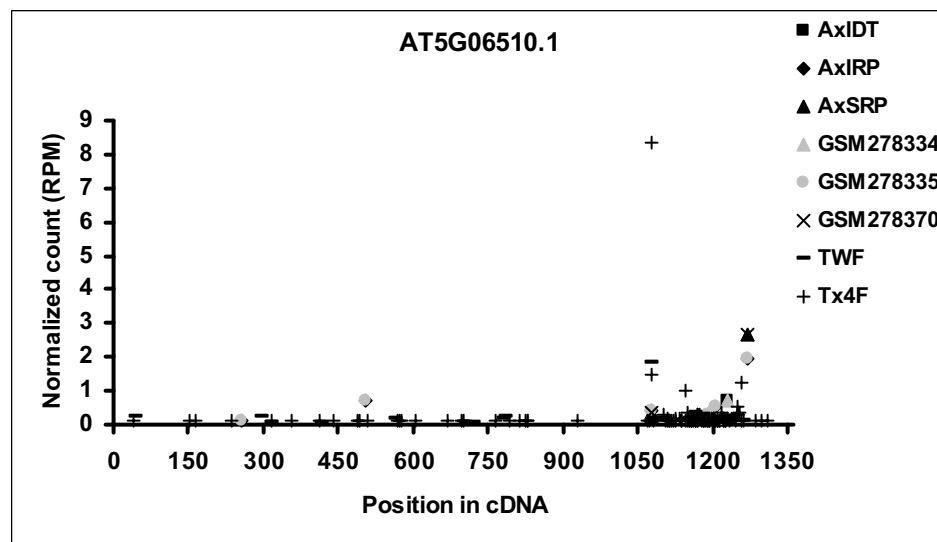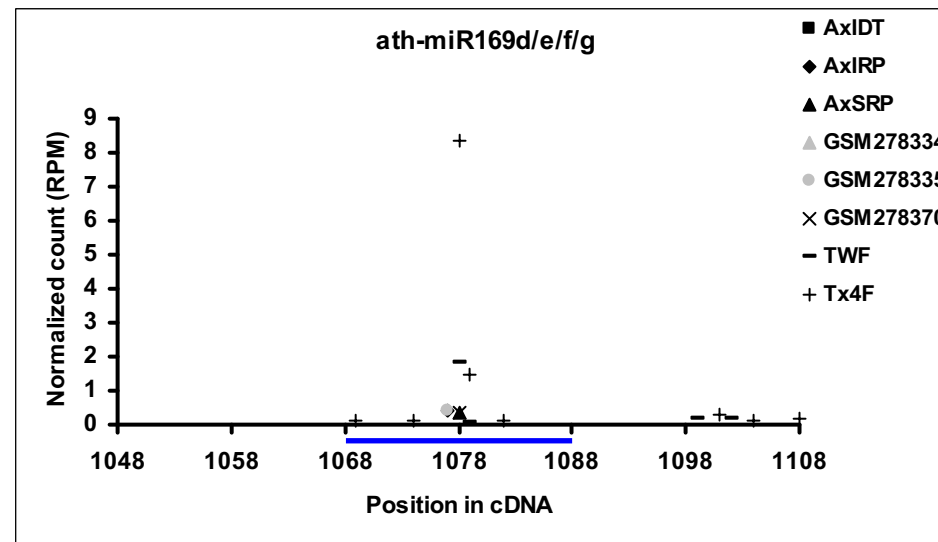

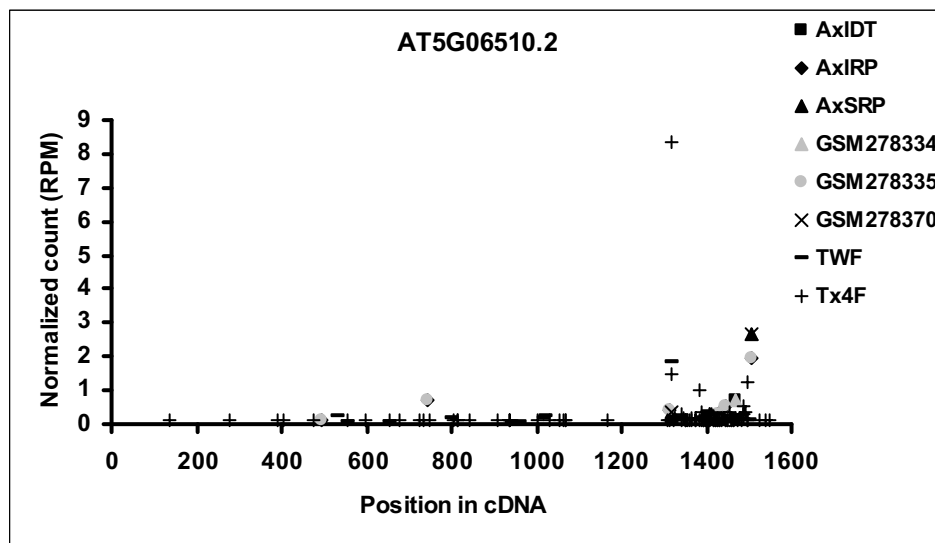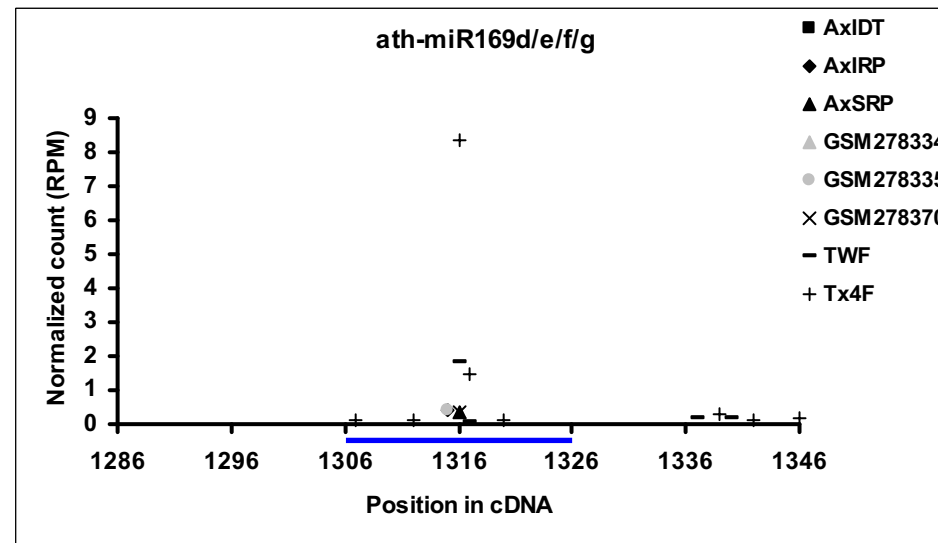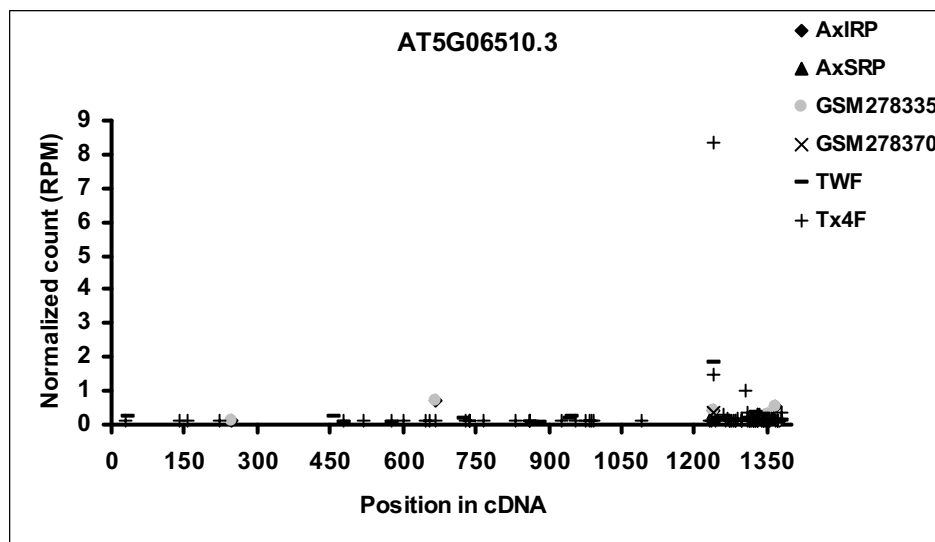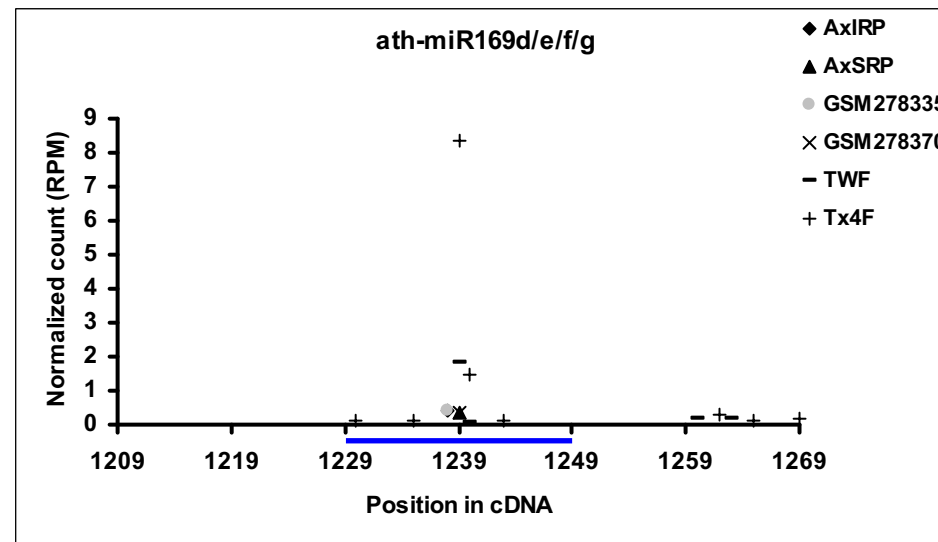

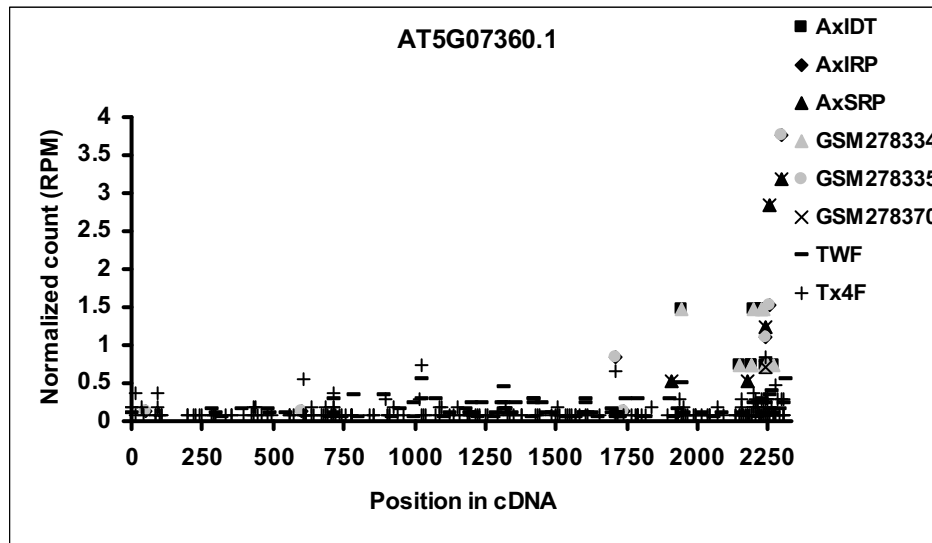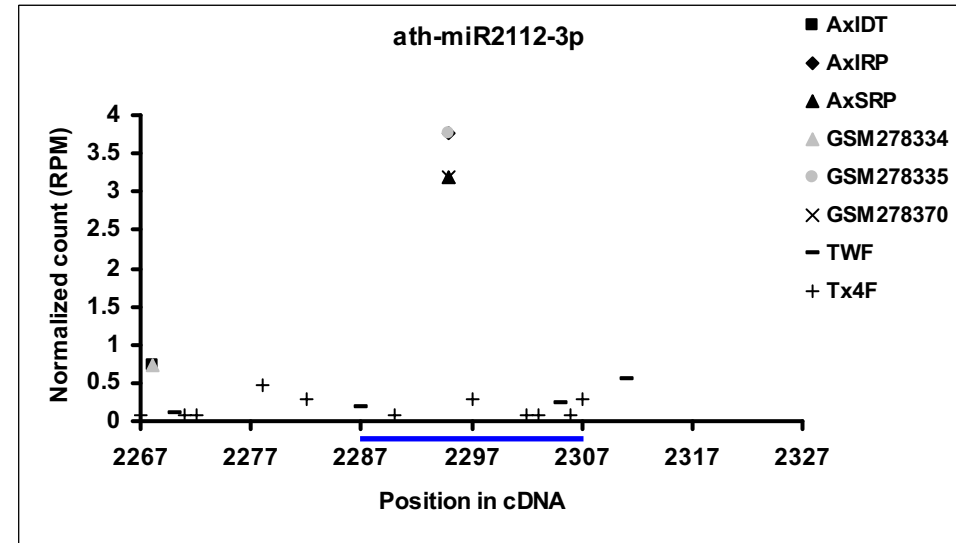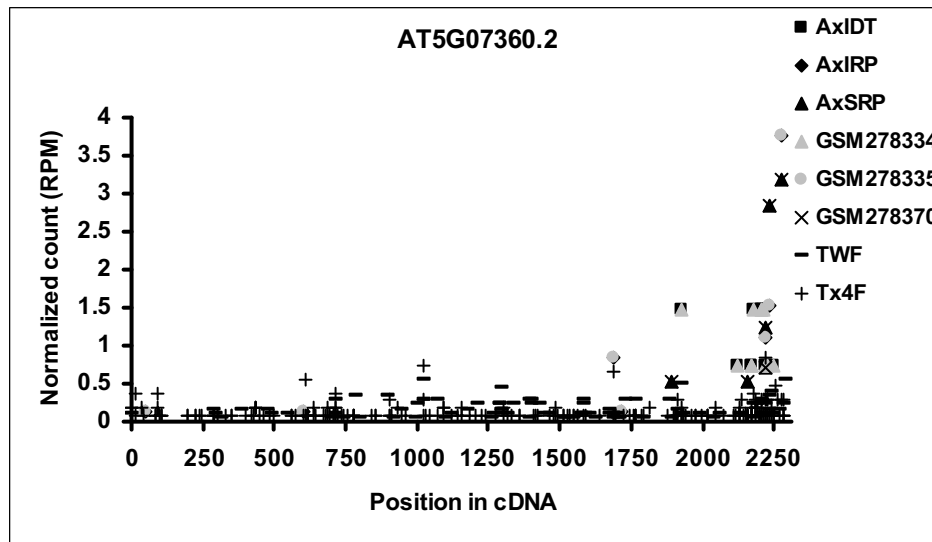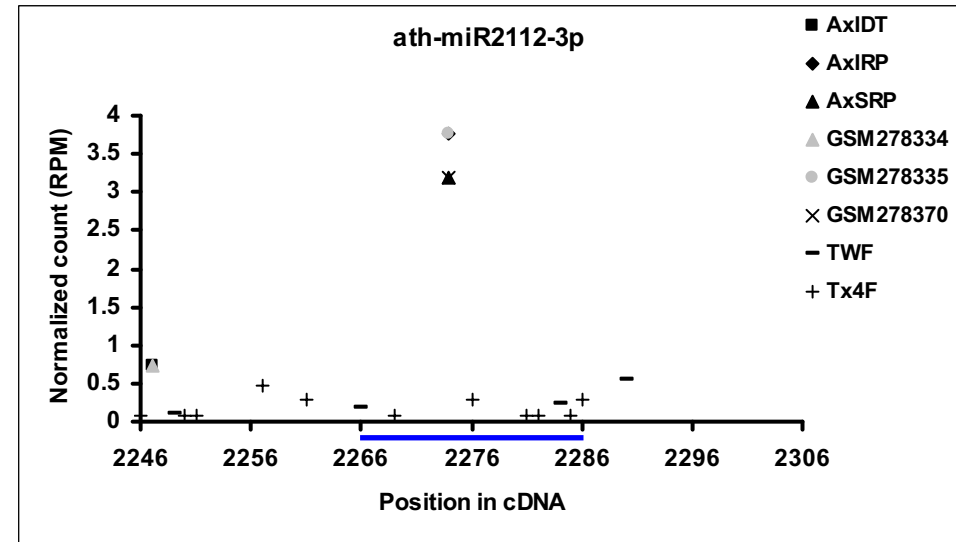

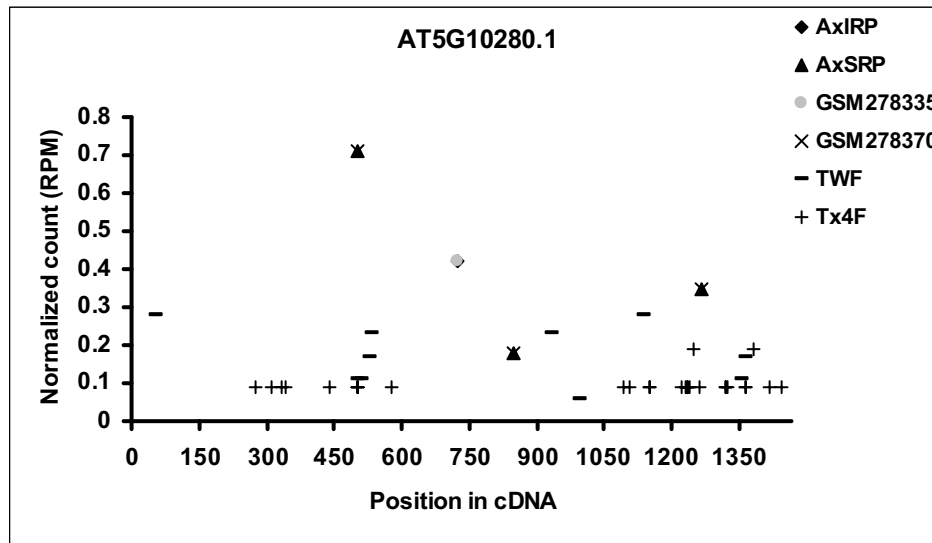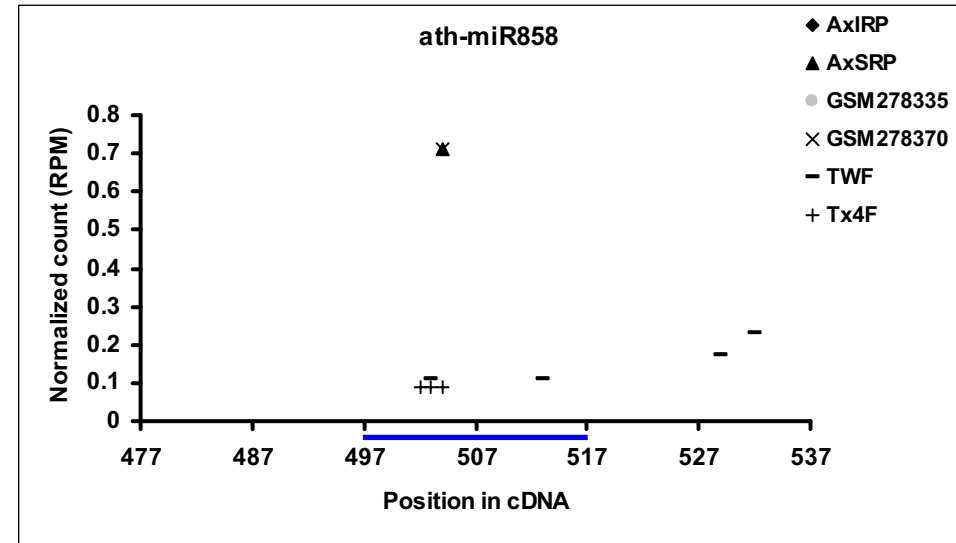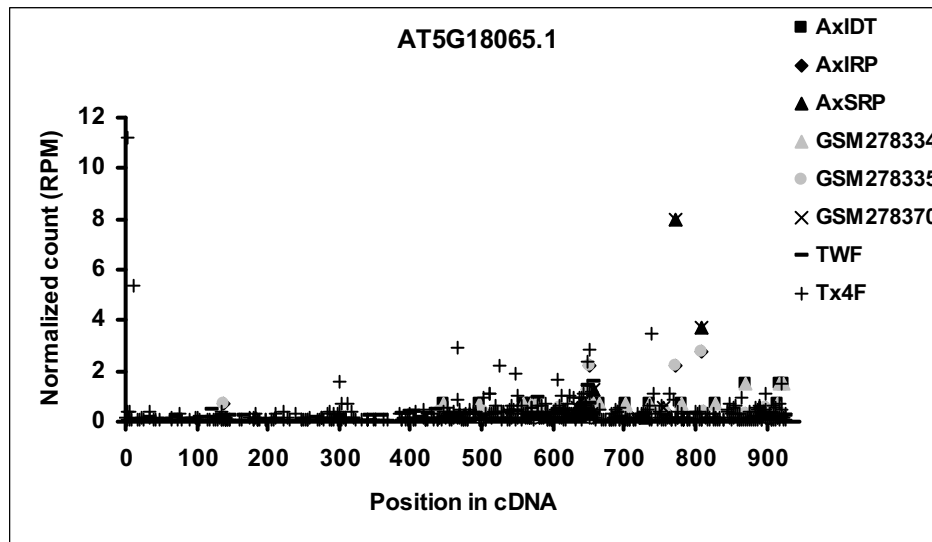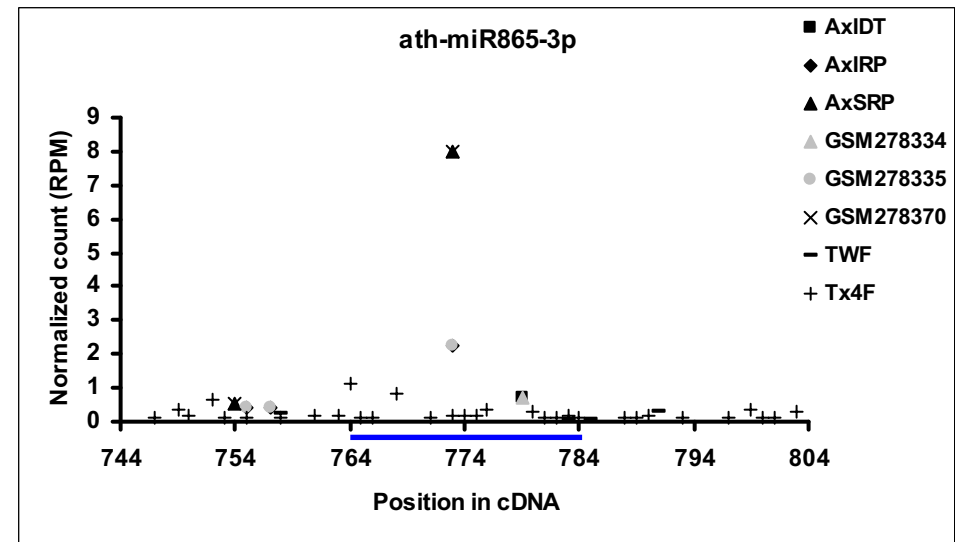

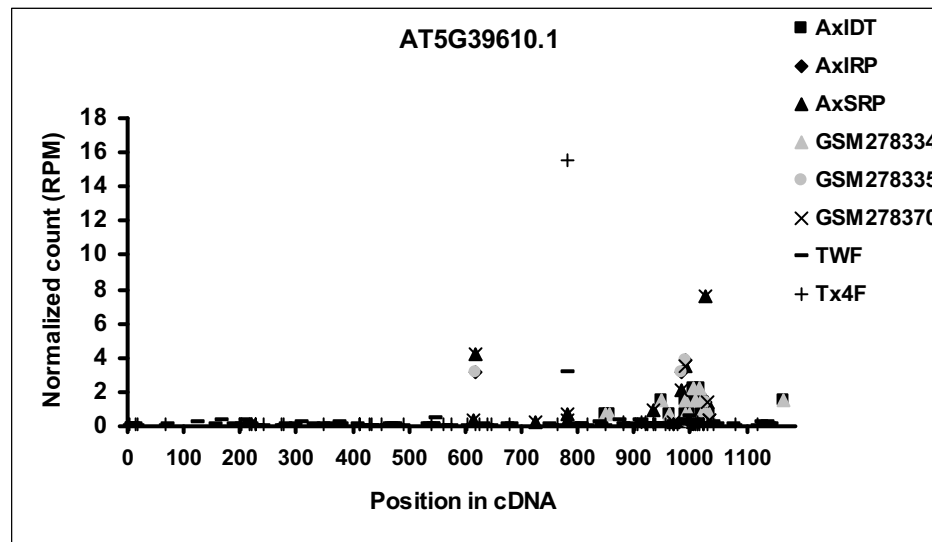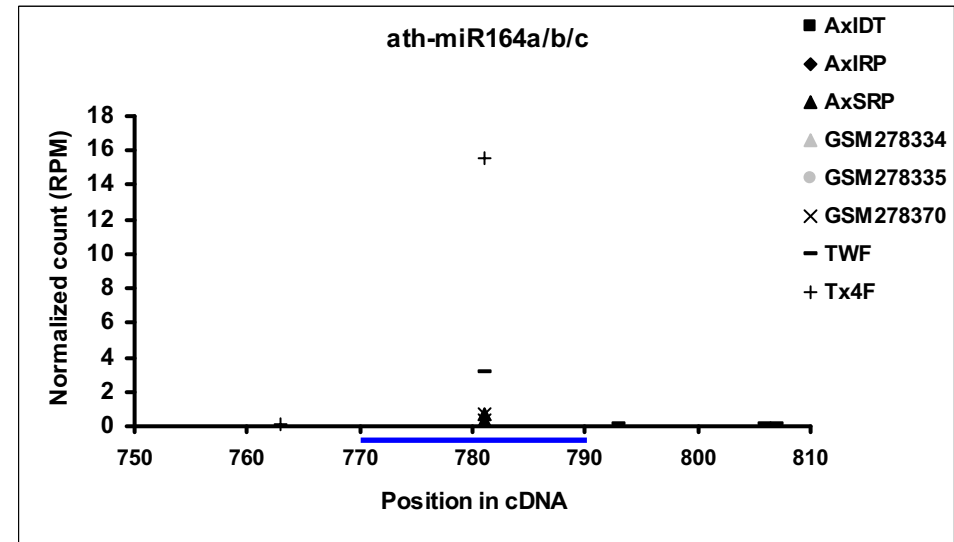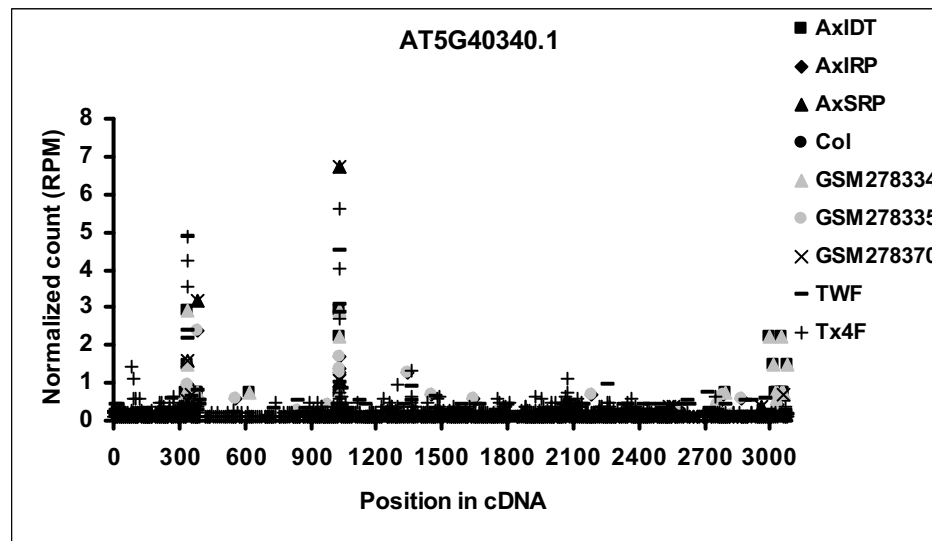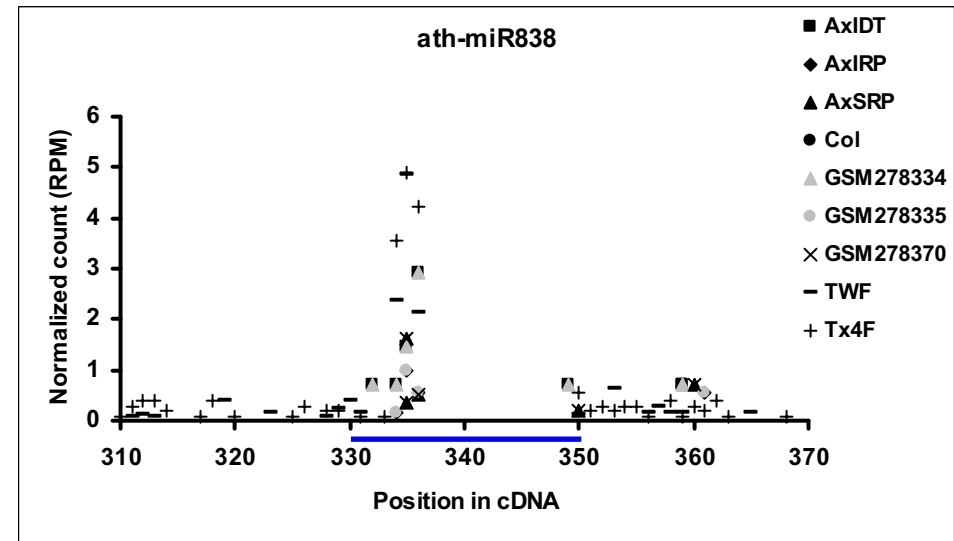

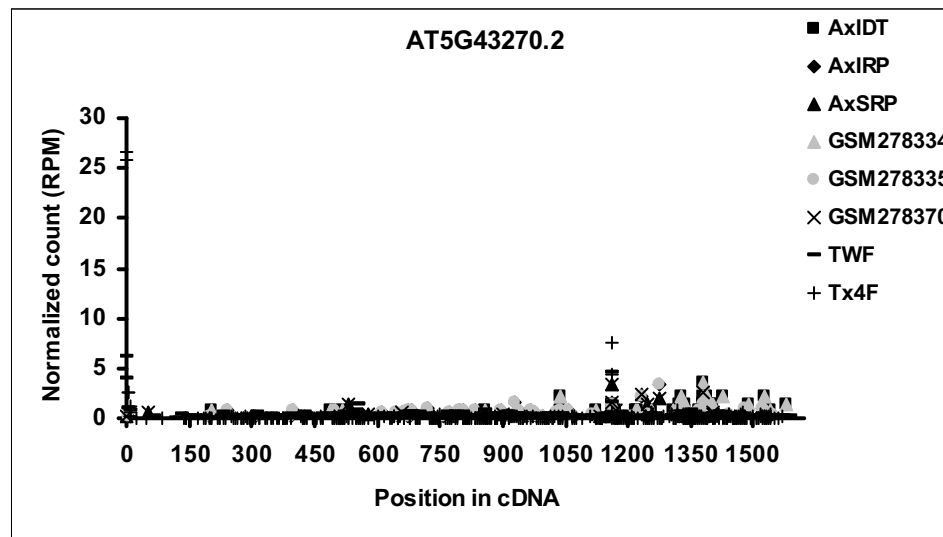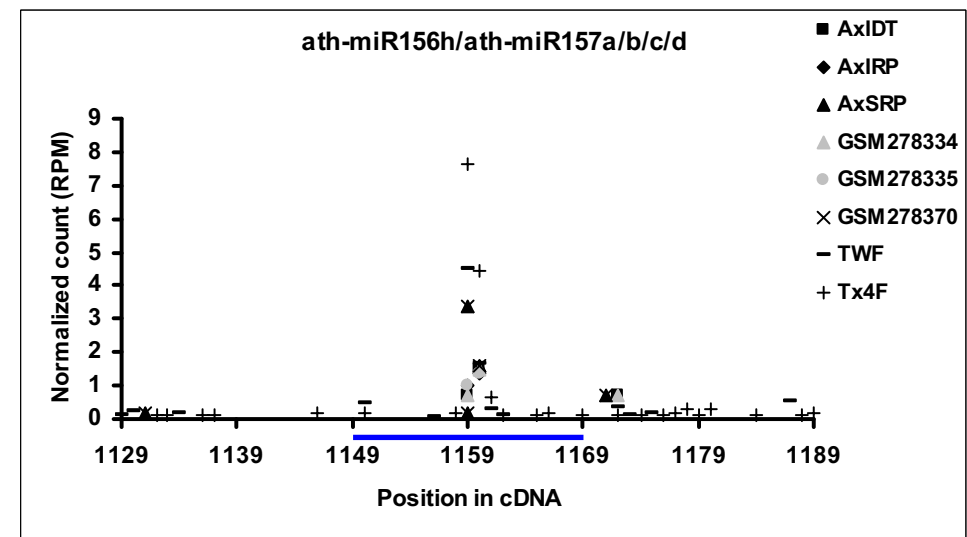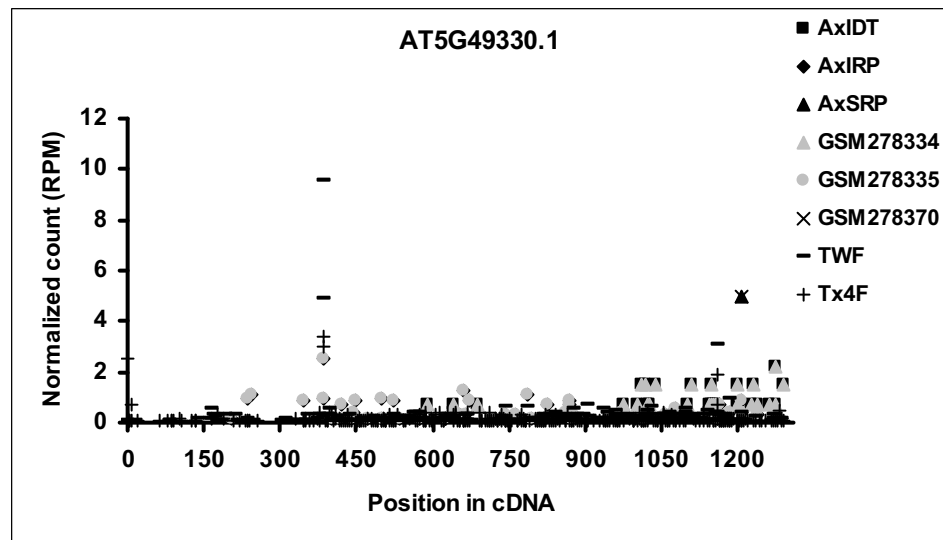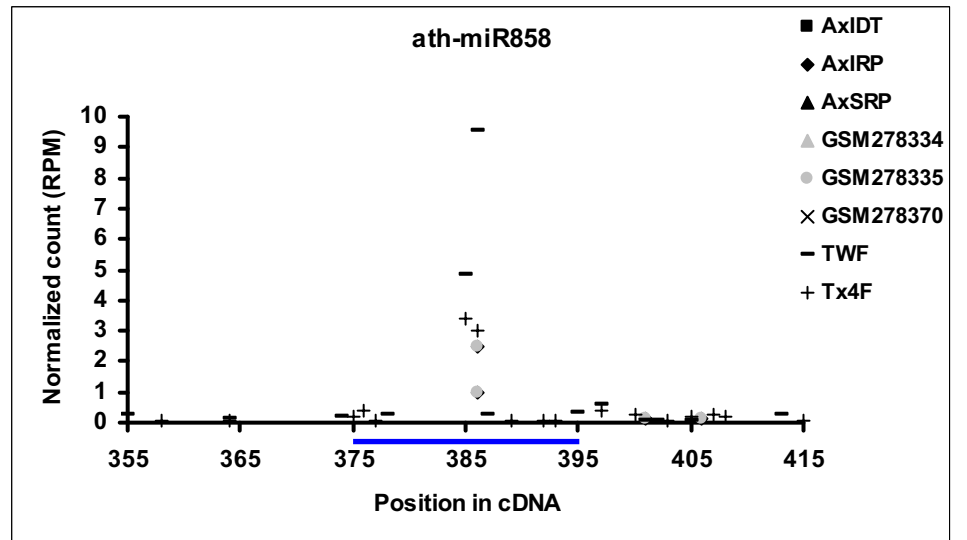

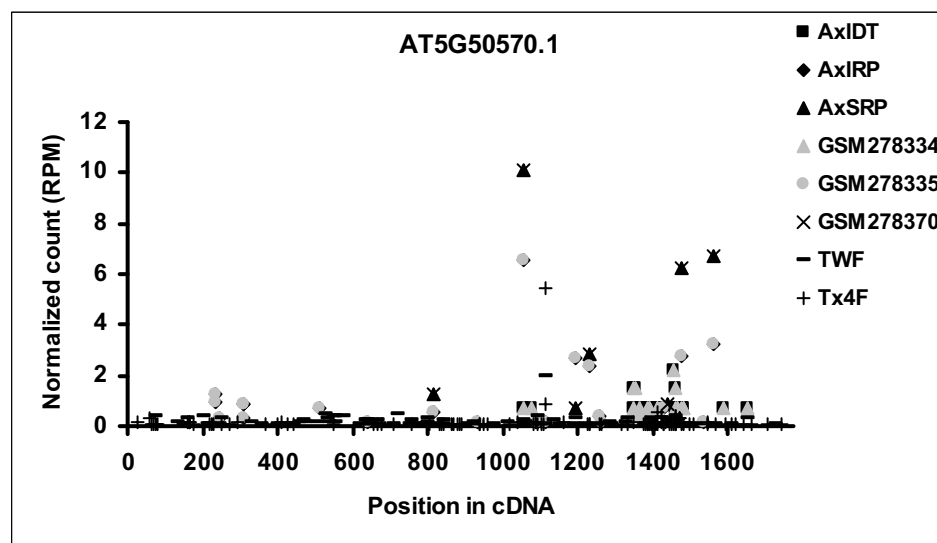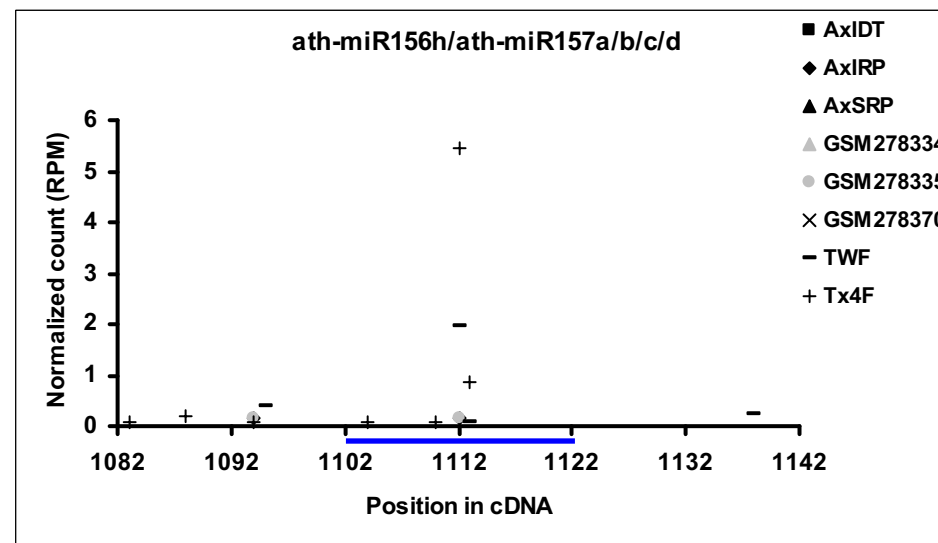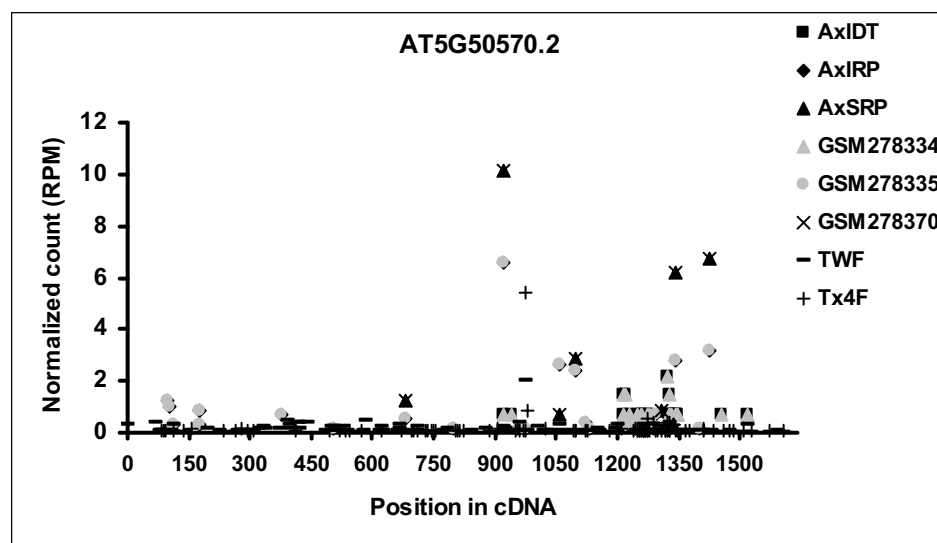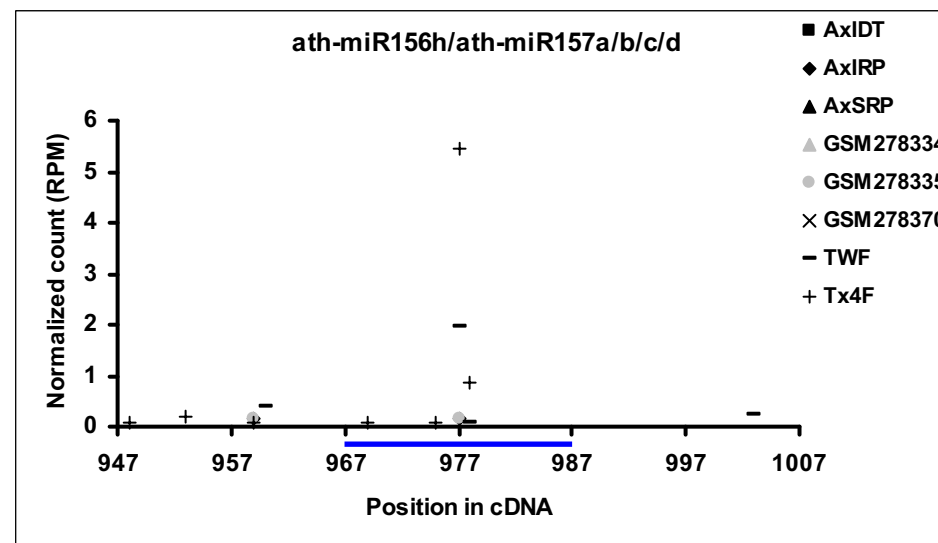

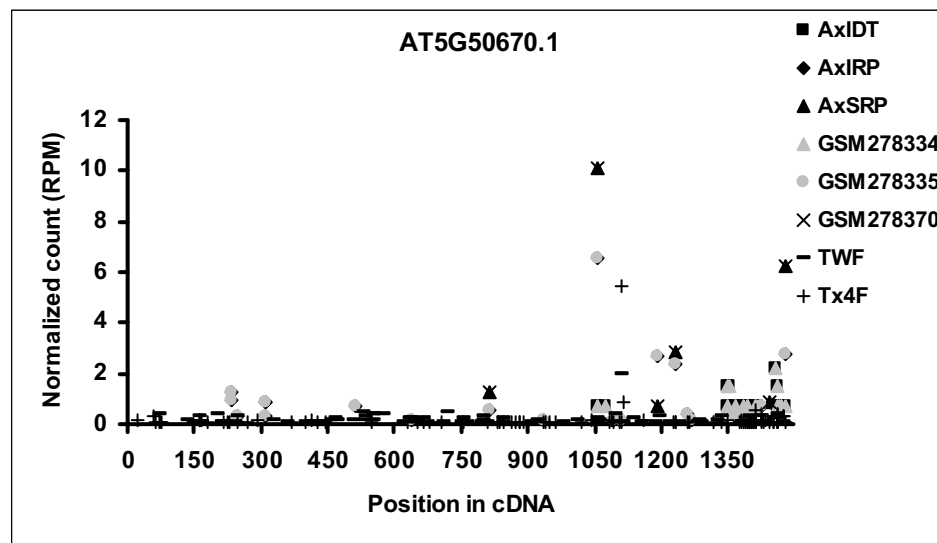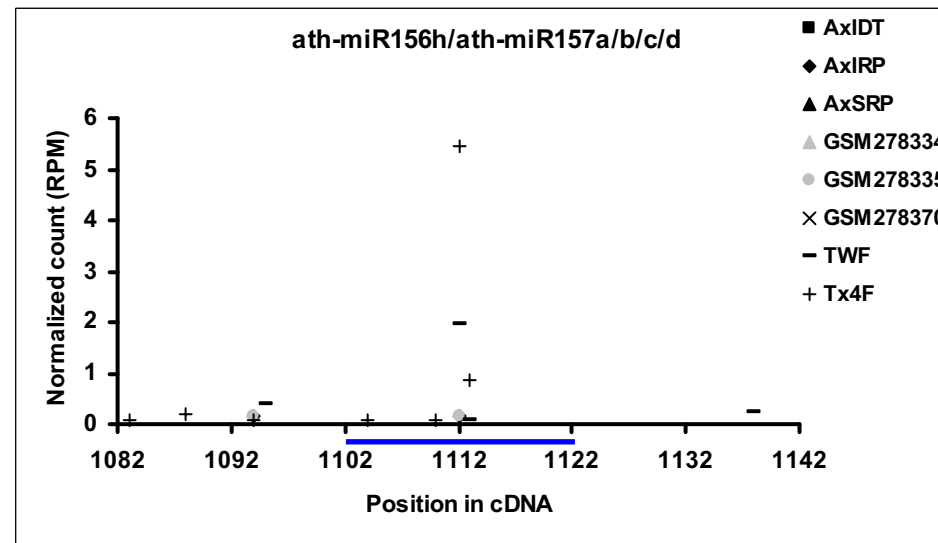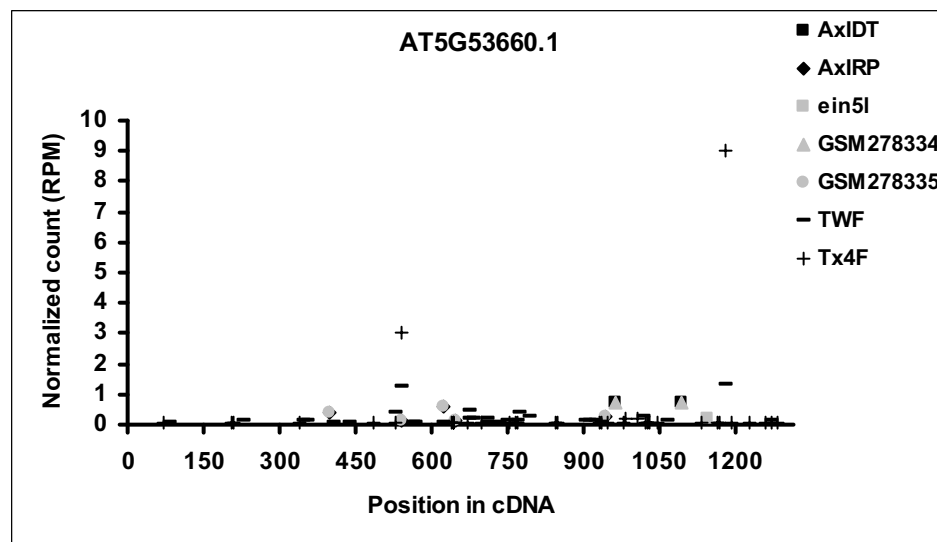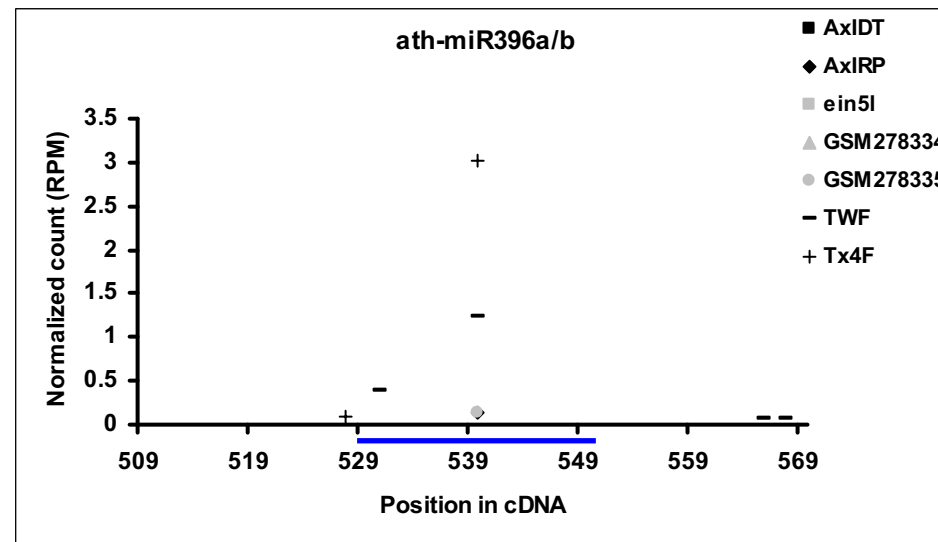

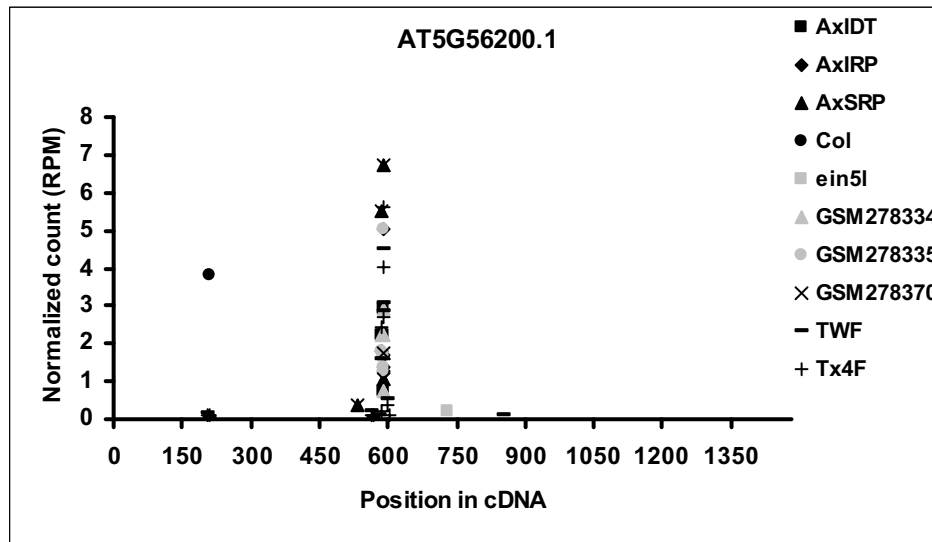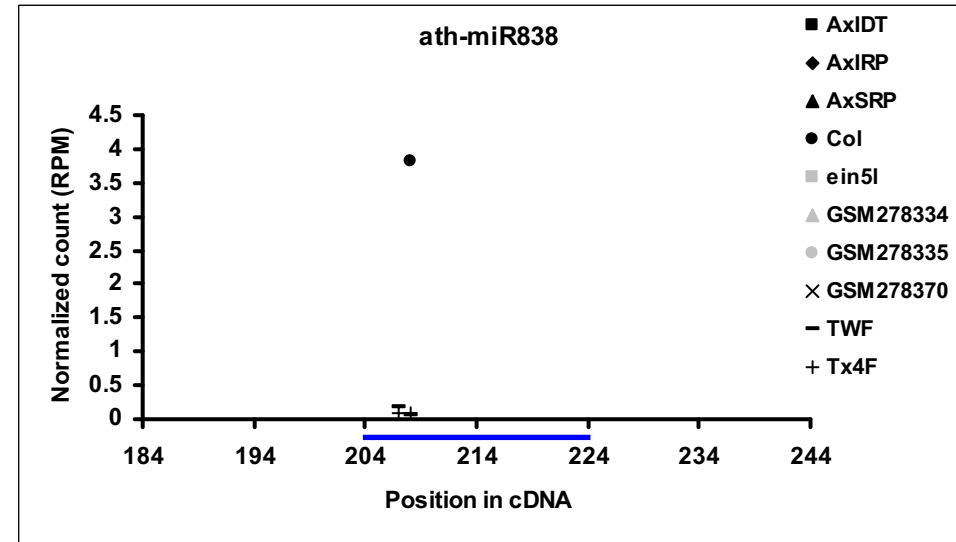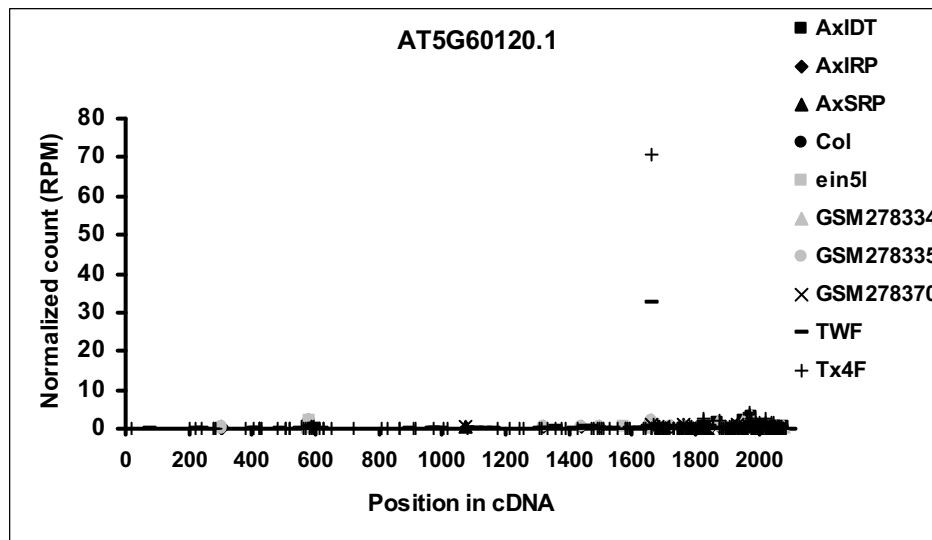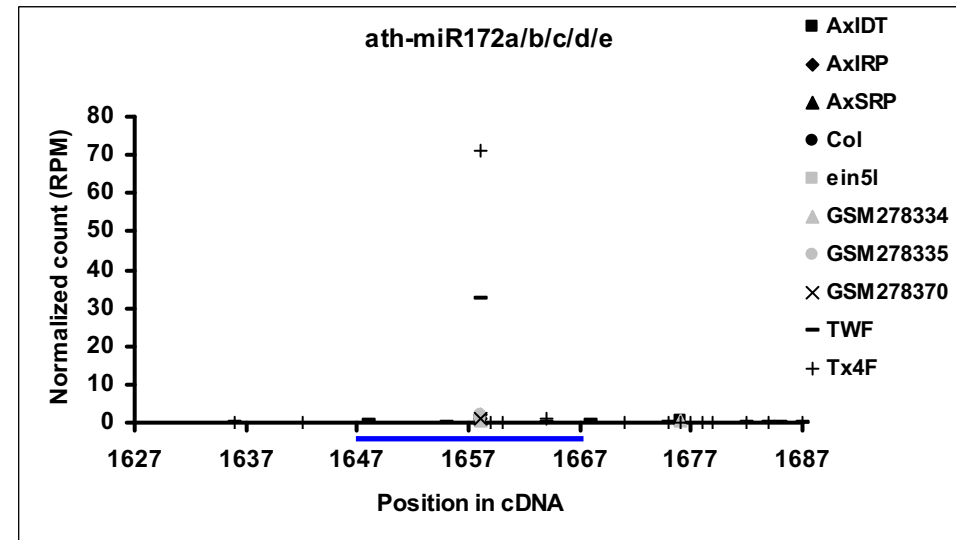

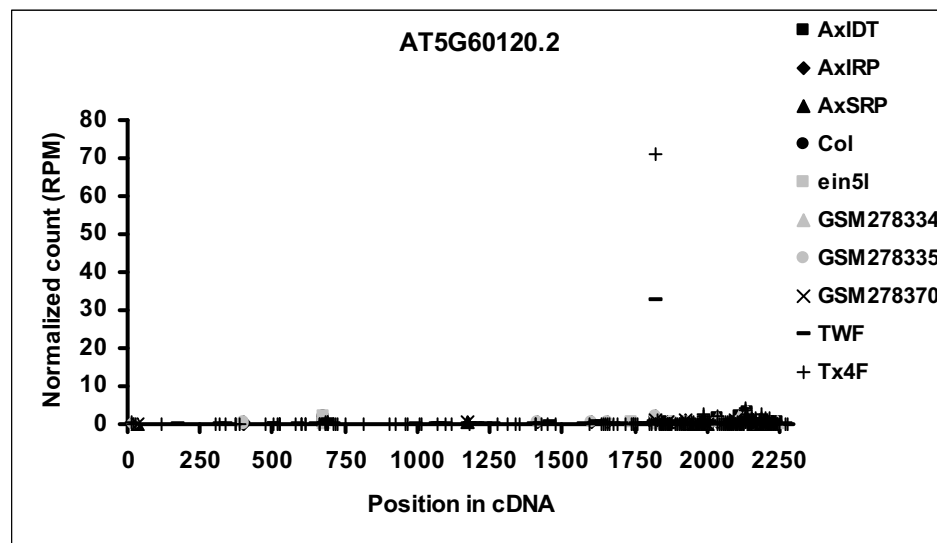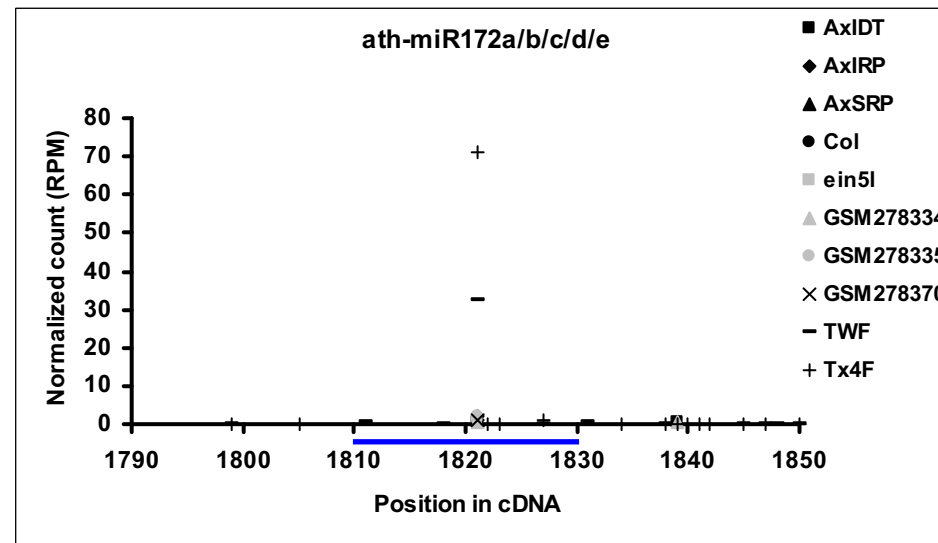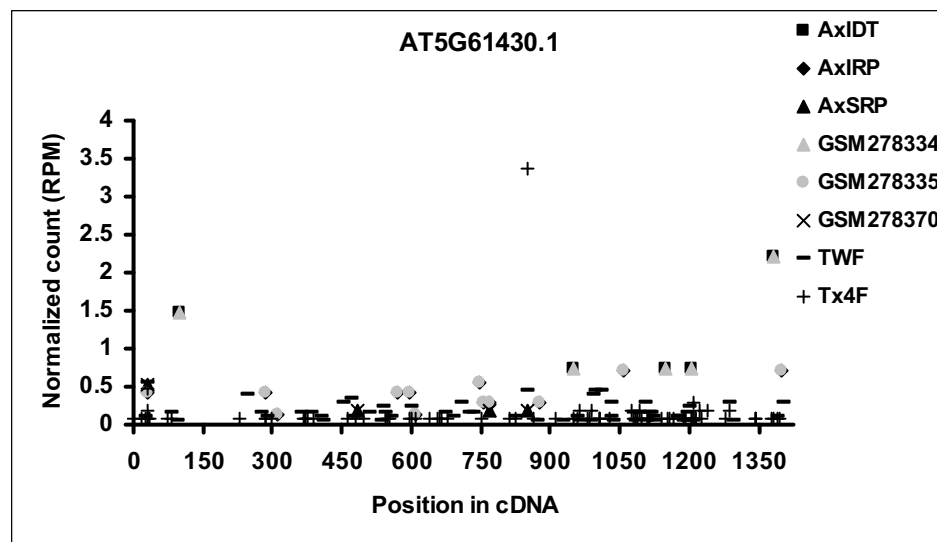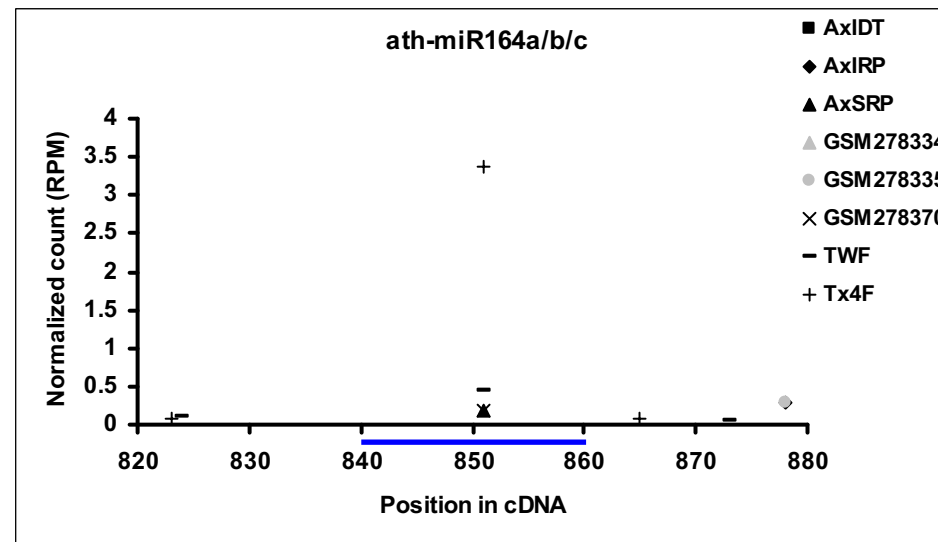

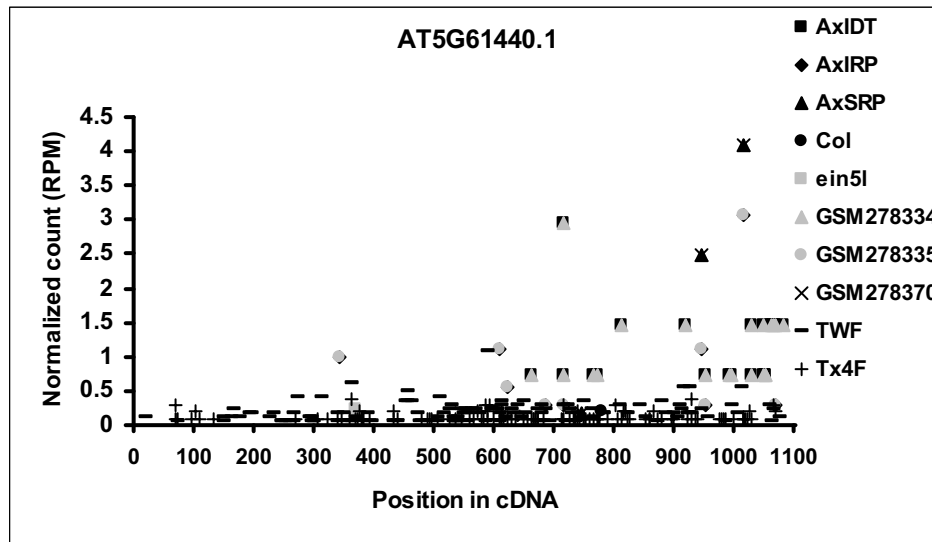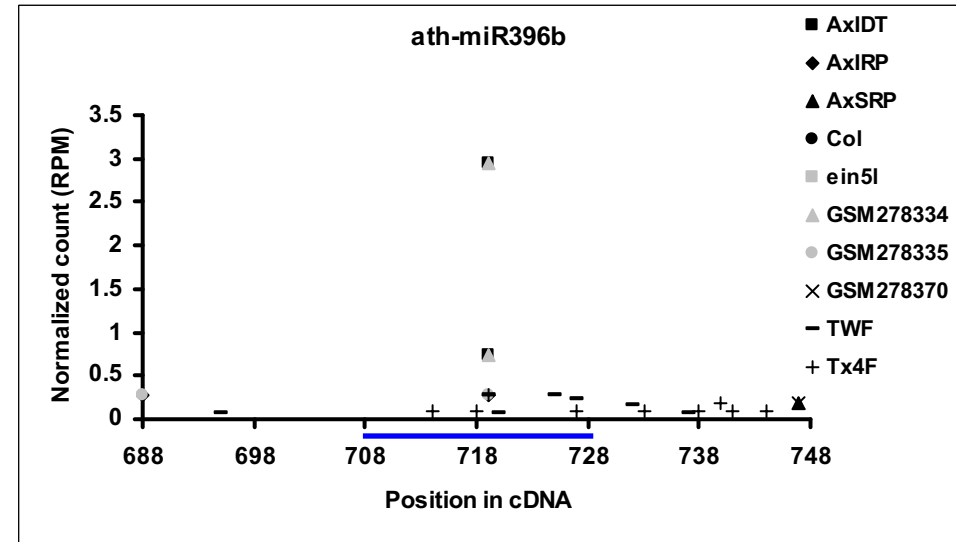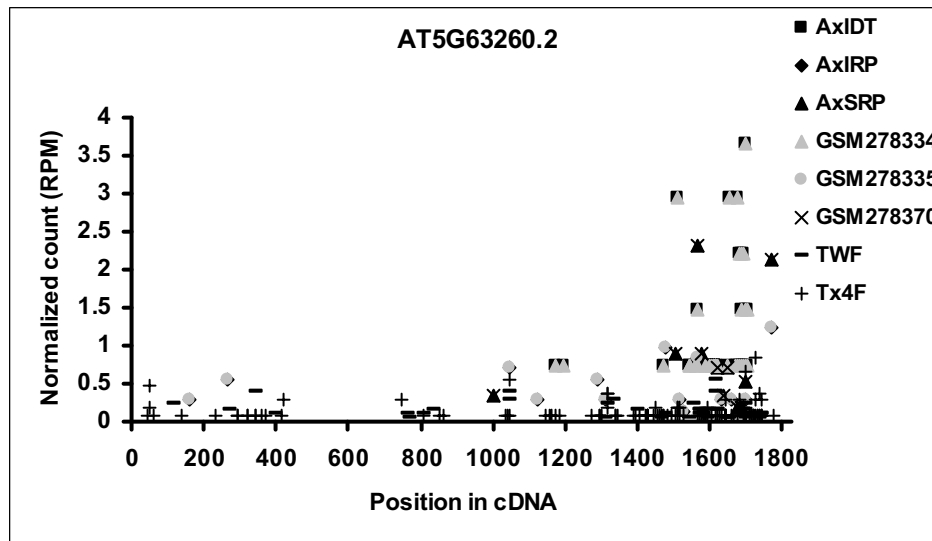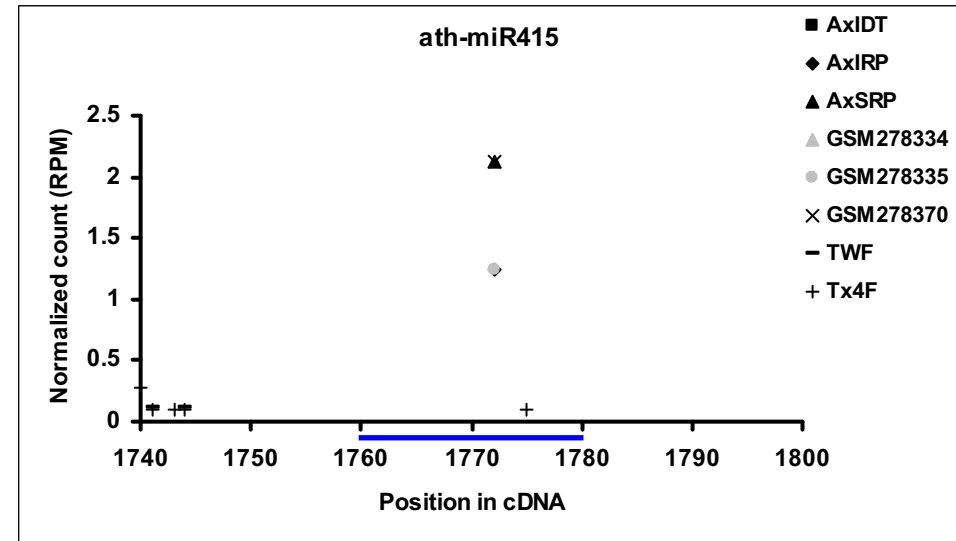

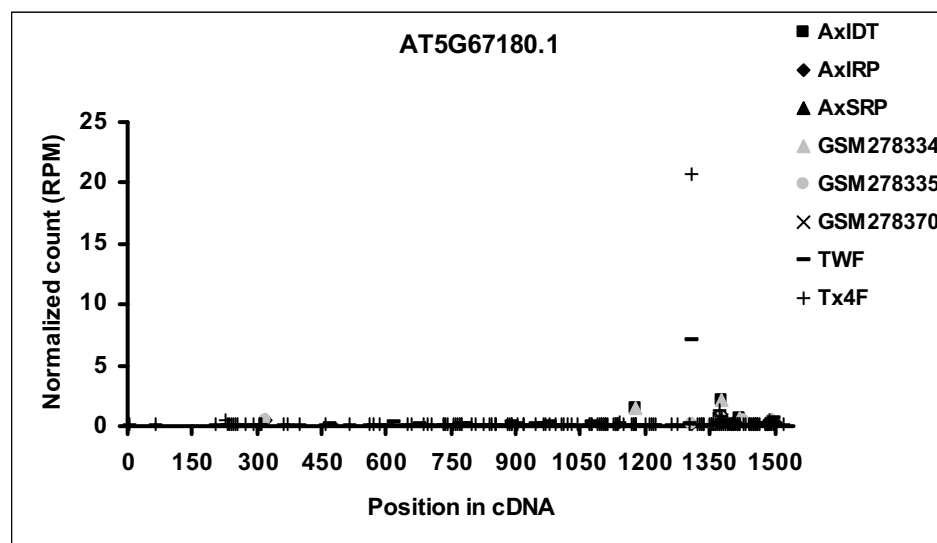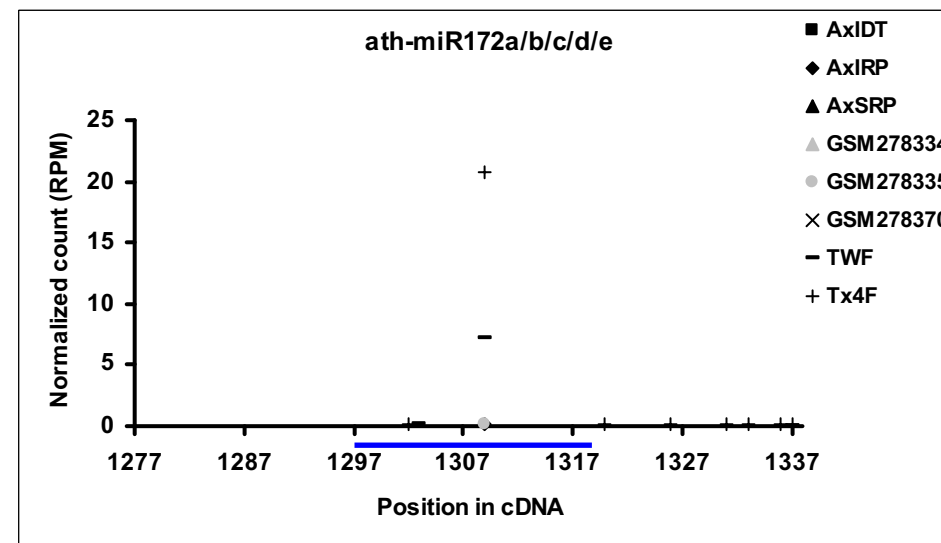

Supplement: Additional file 4 — Figure S1. Degradome sequencing data-based identification of the targets of sequestered microRNAs in Arabidopsis. [file 1471-2164-13-197-S4.pdf]

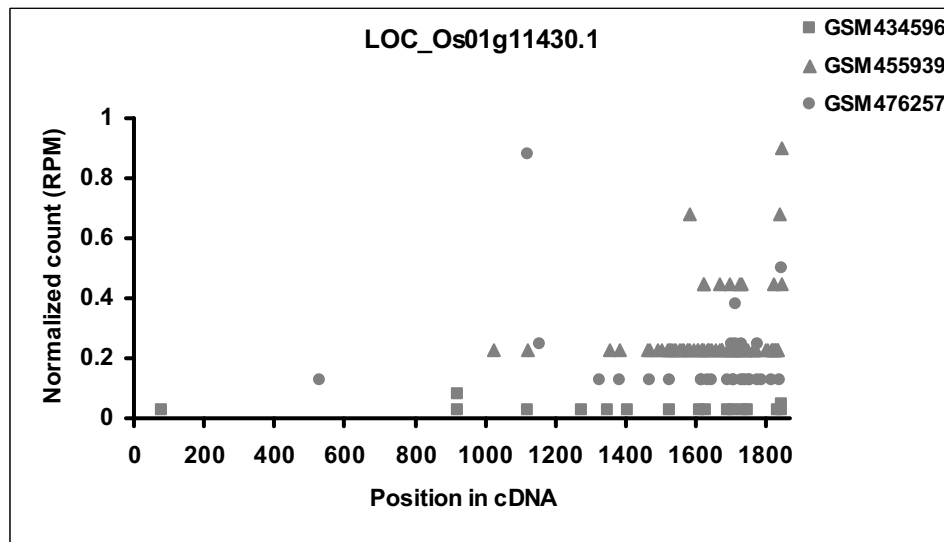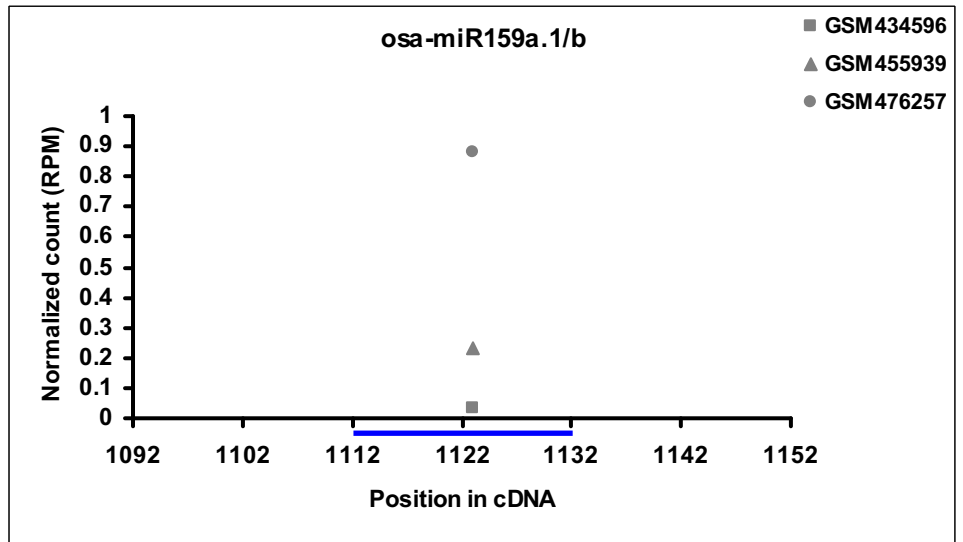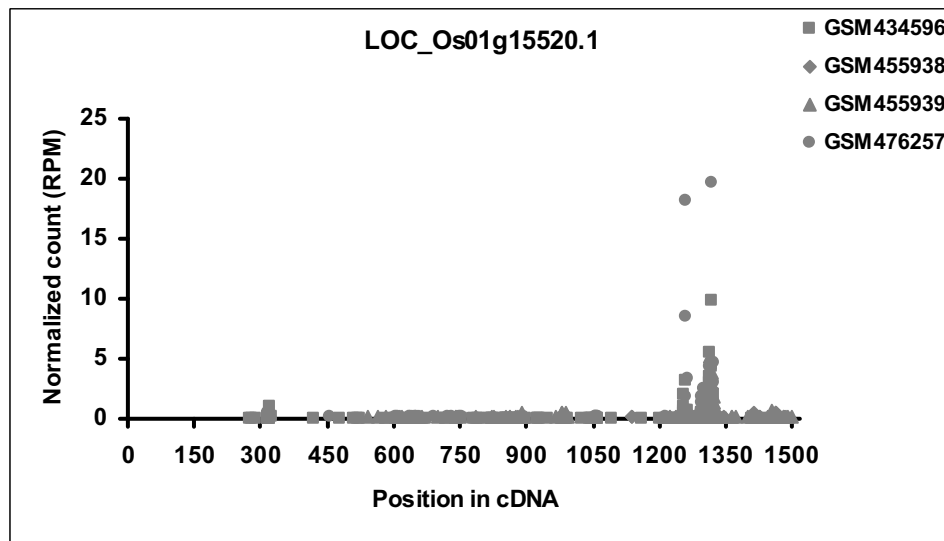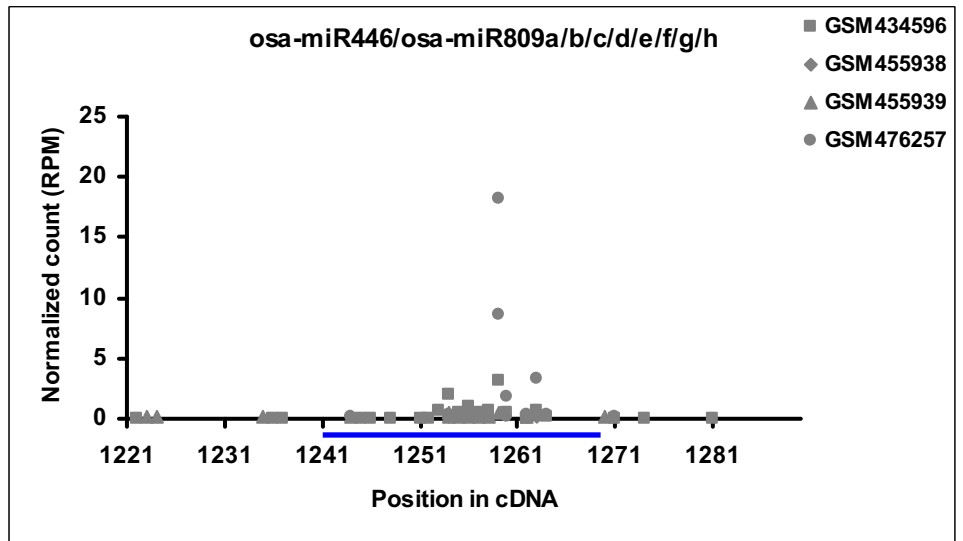

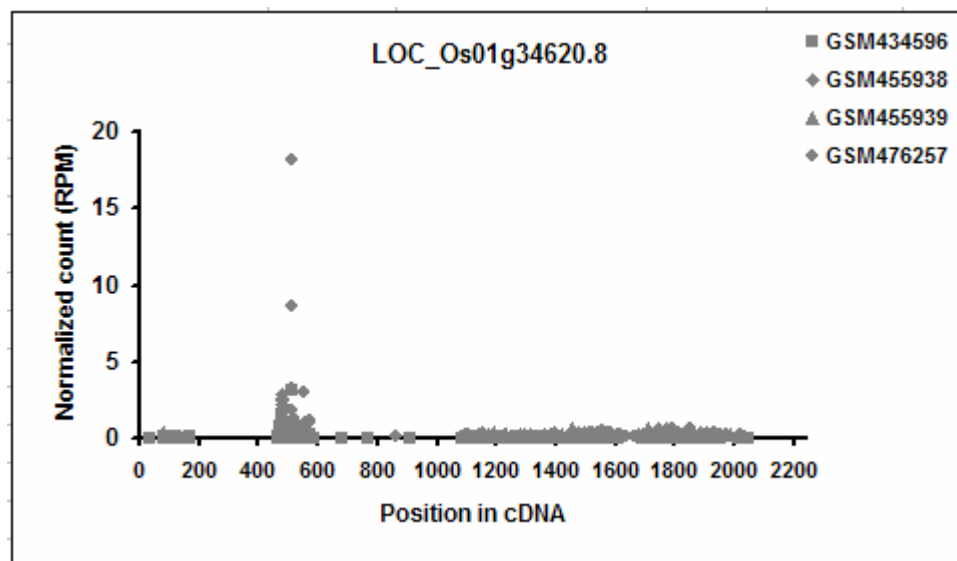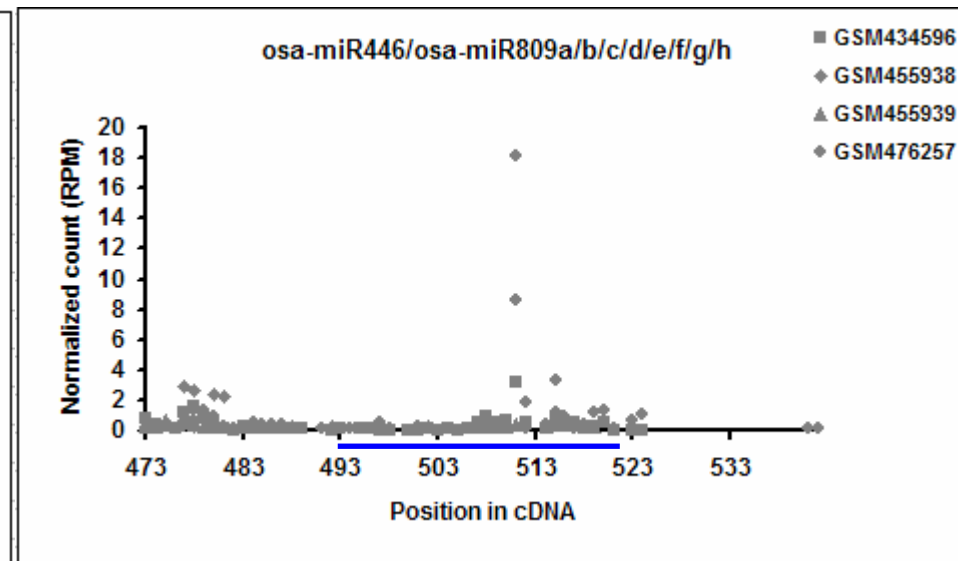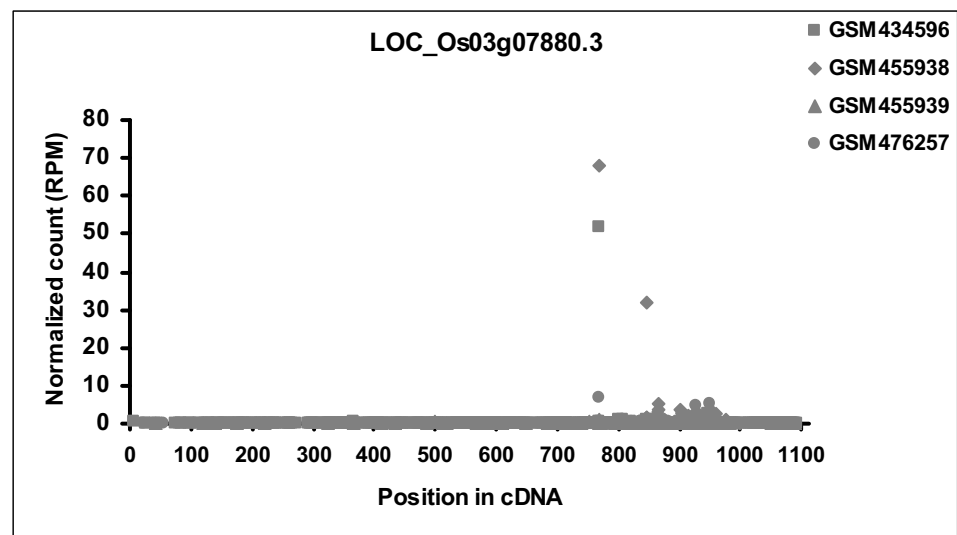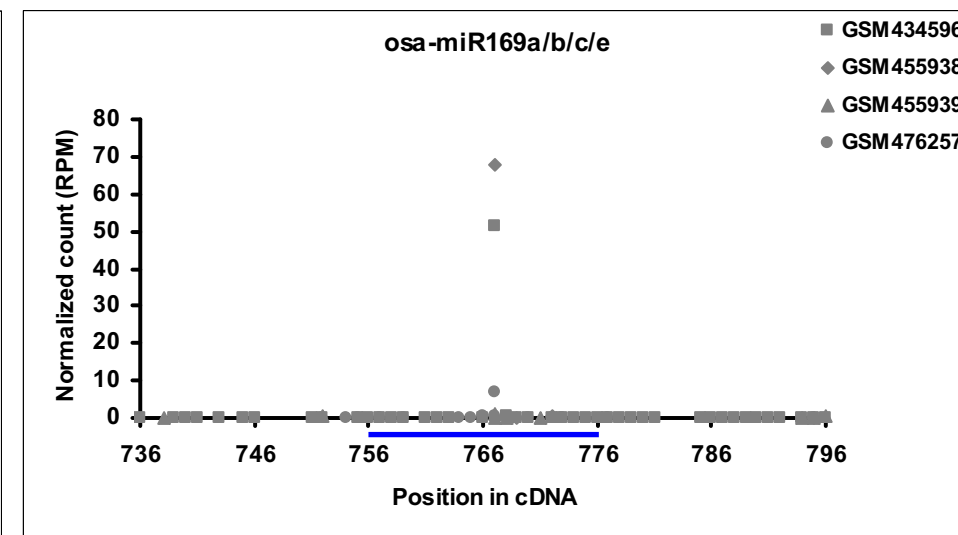

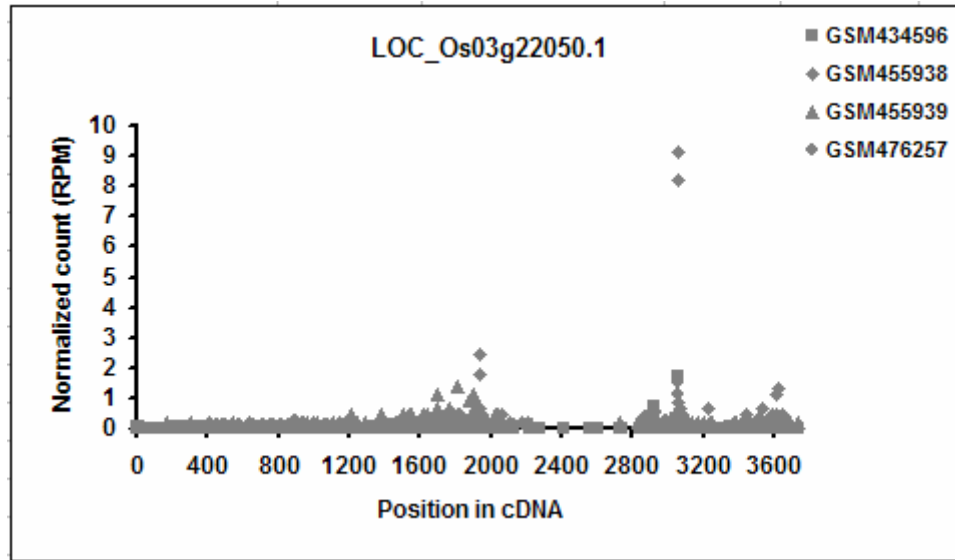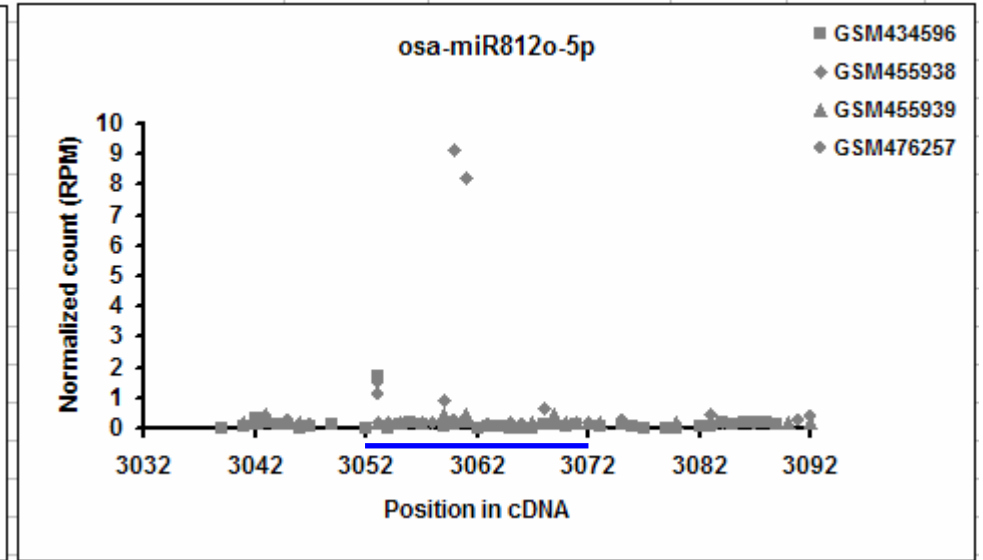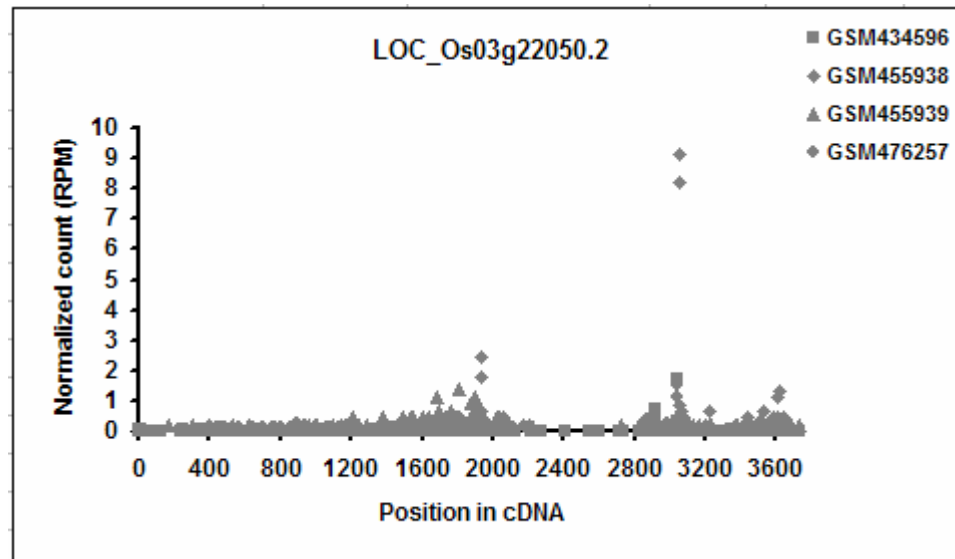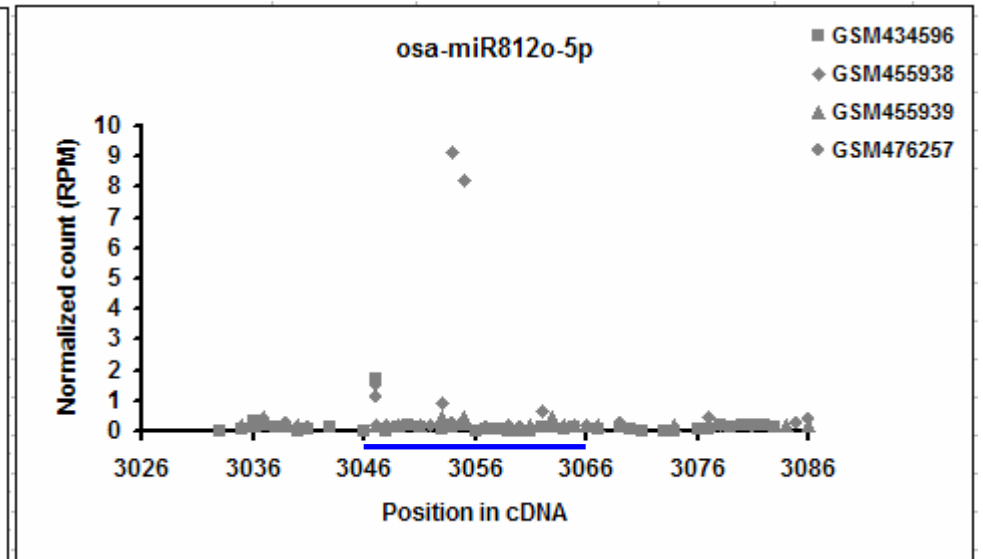

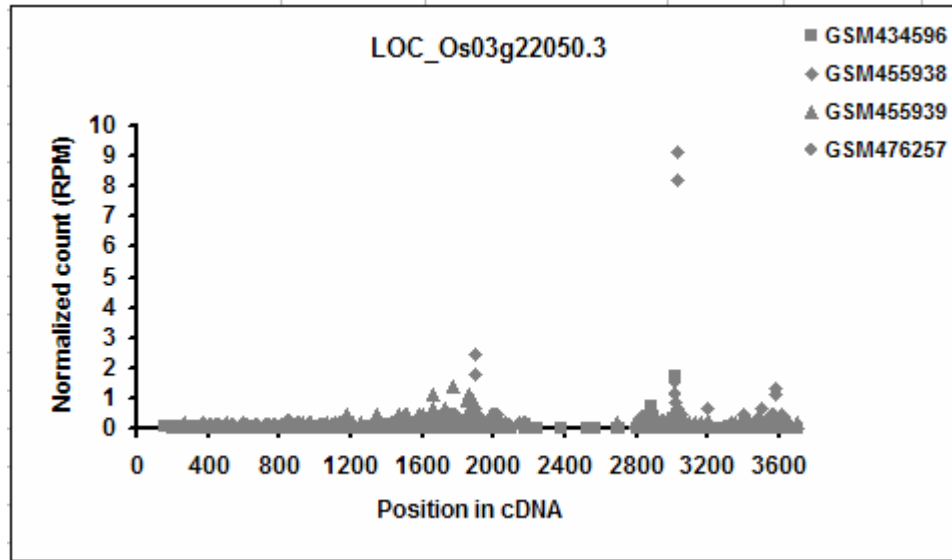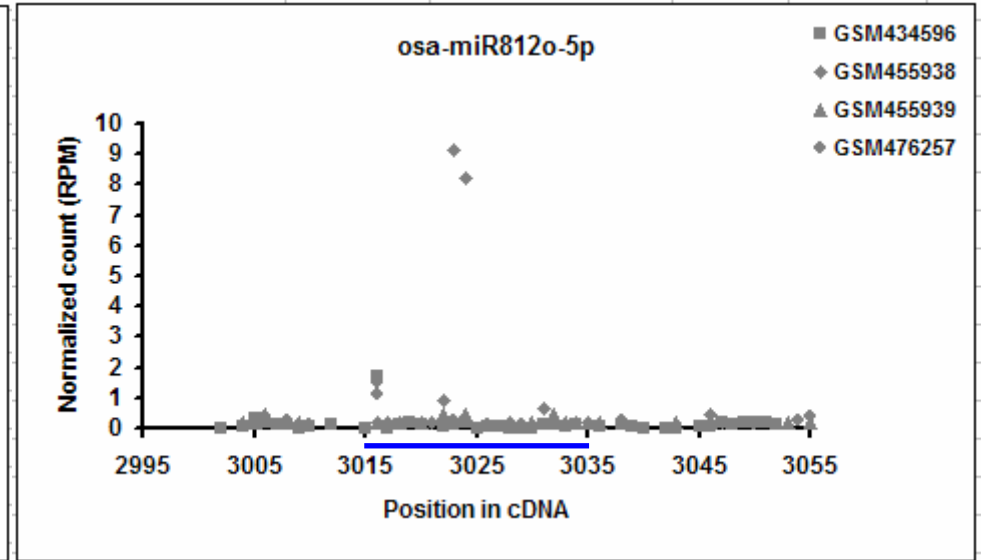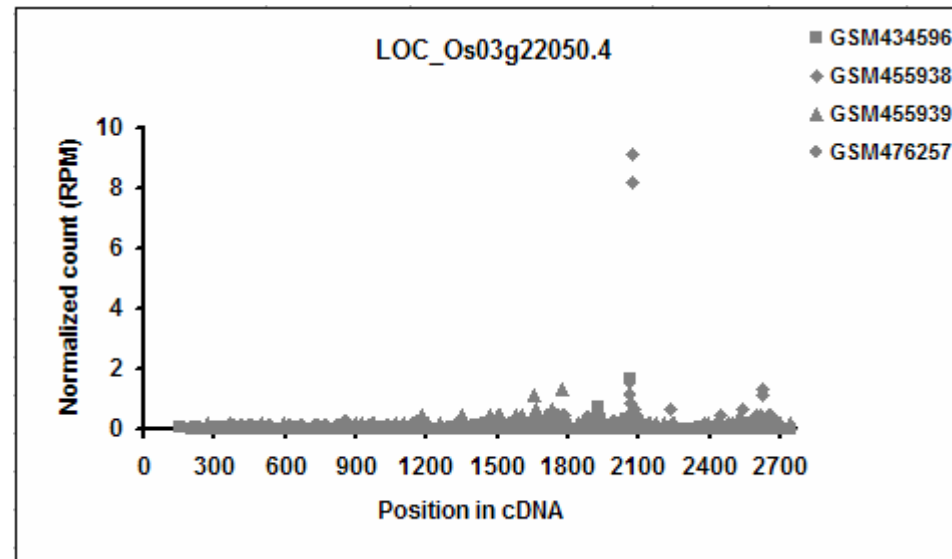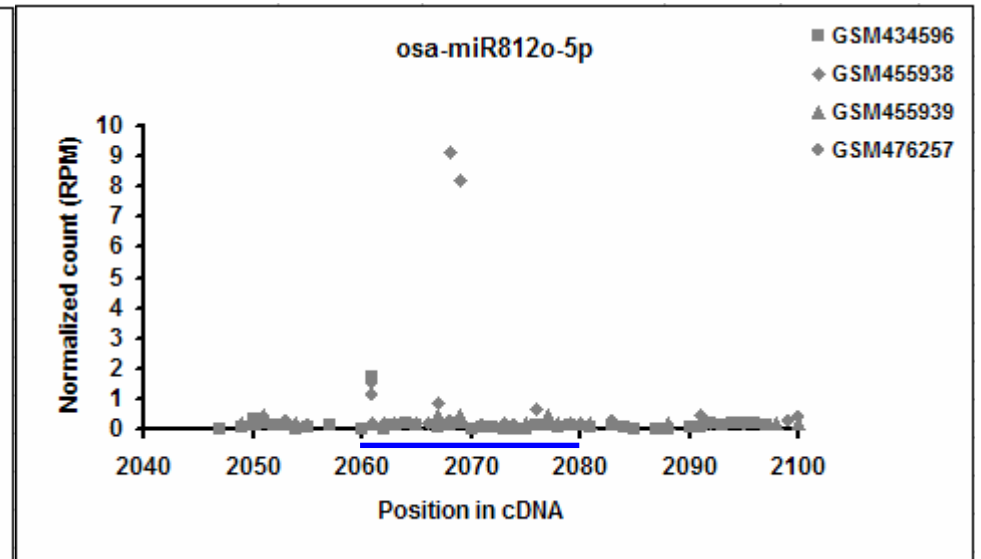

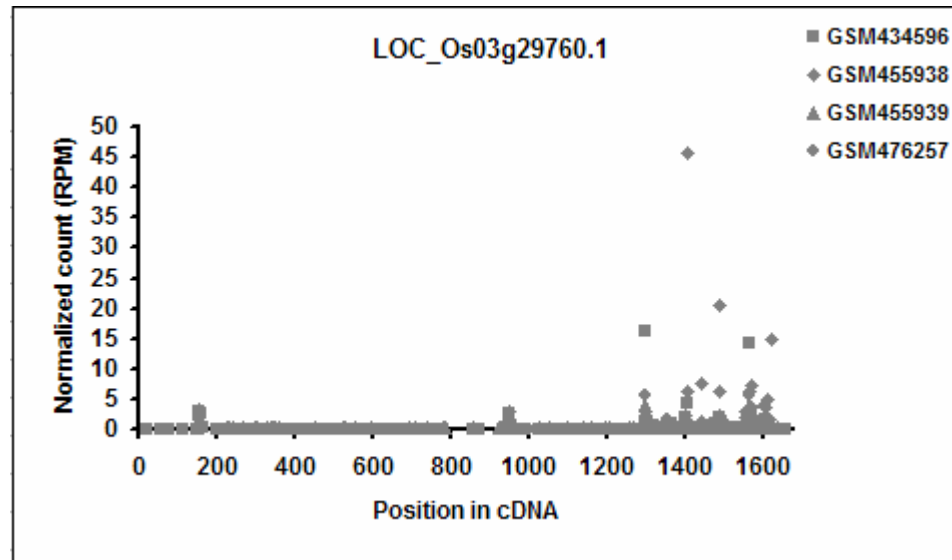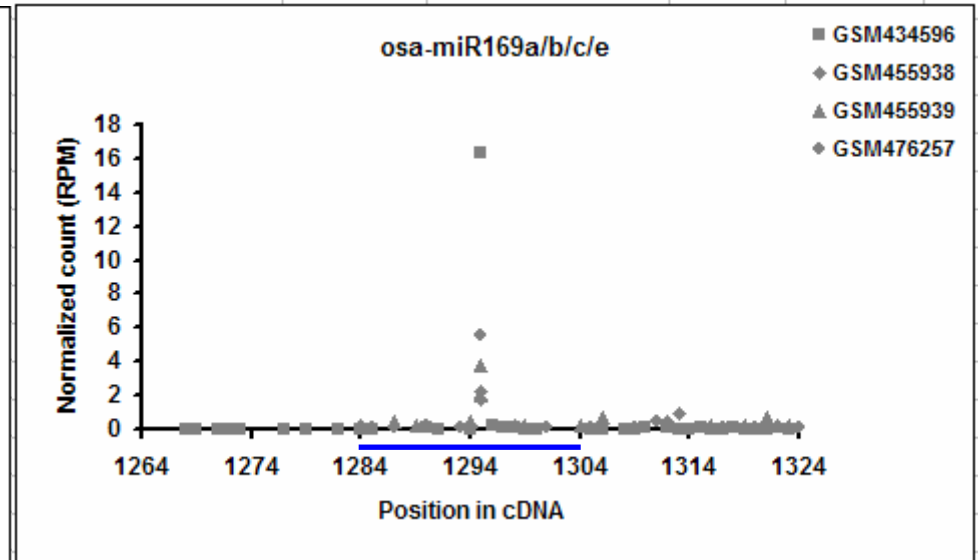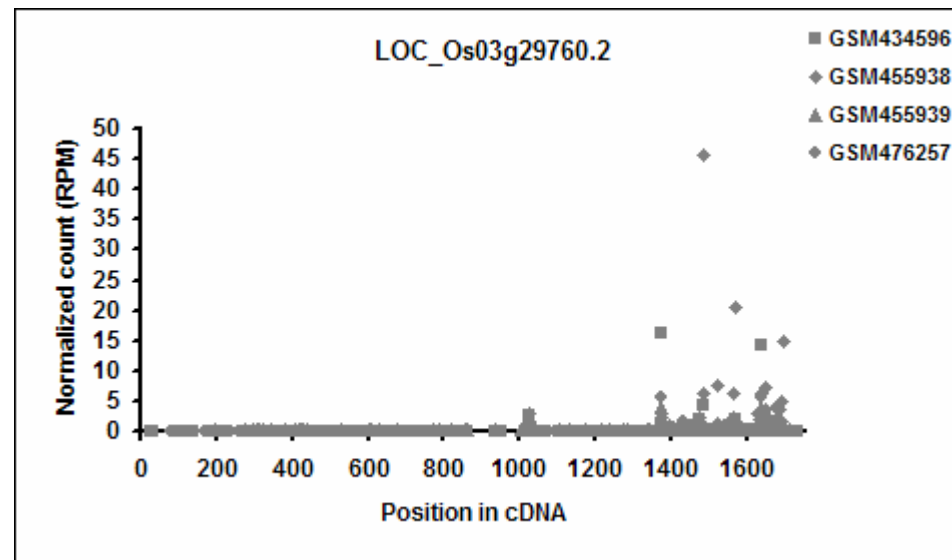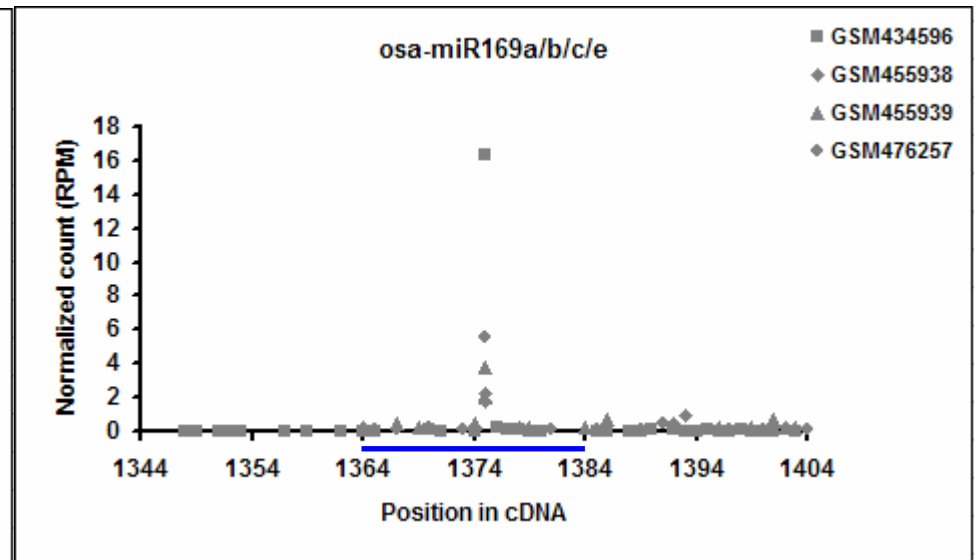

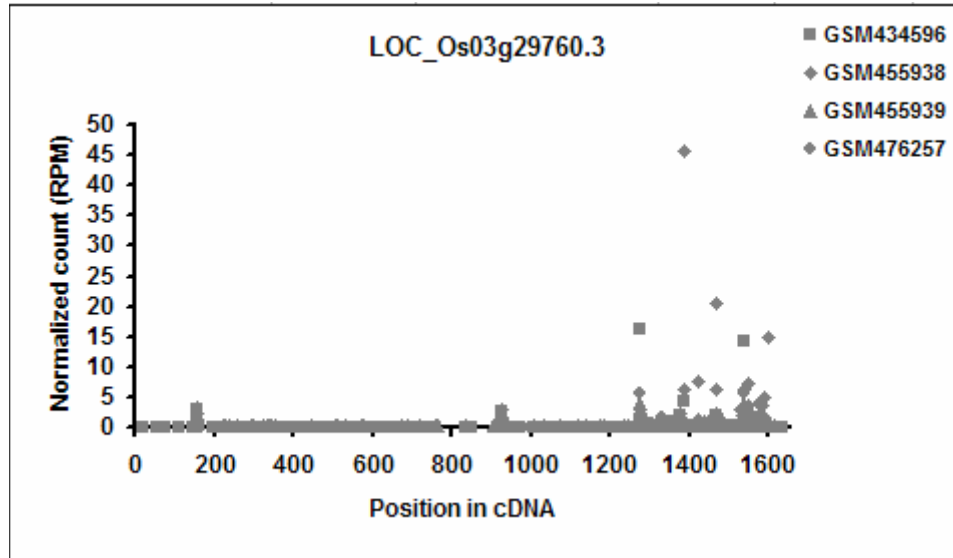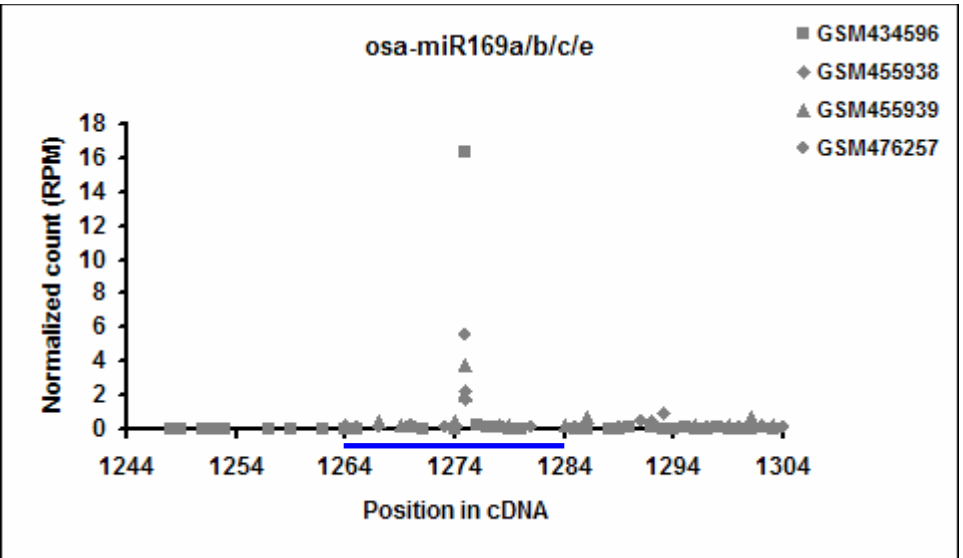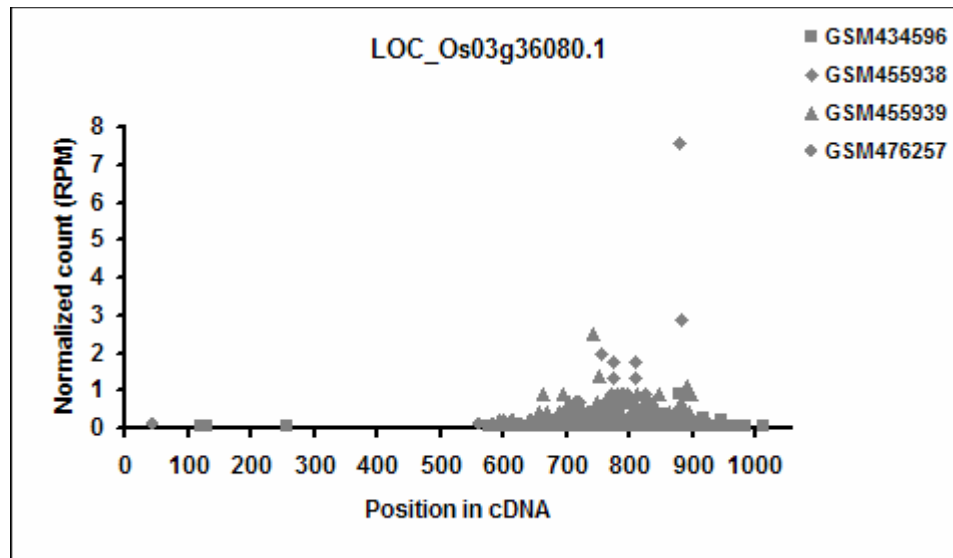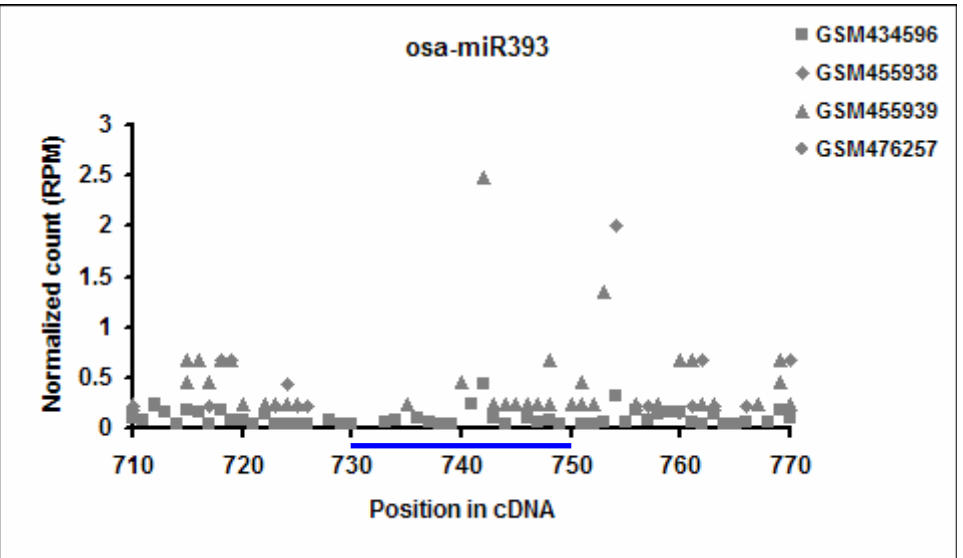

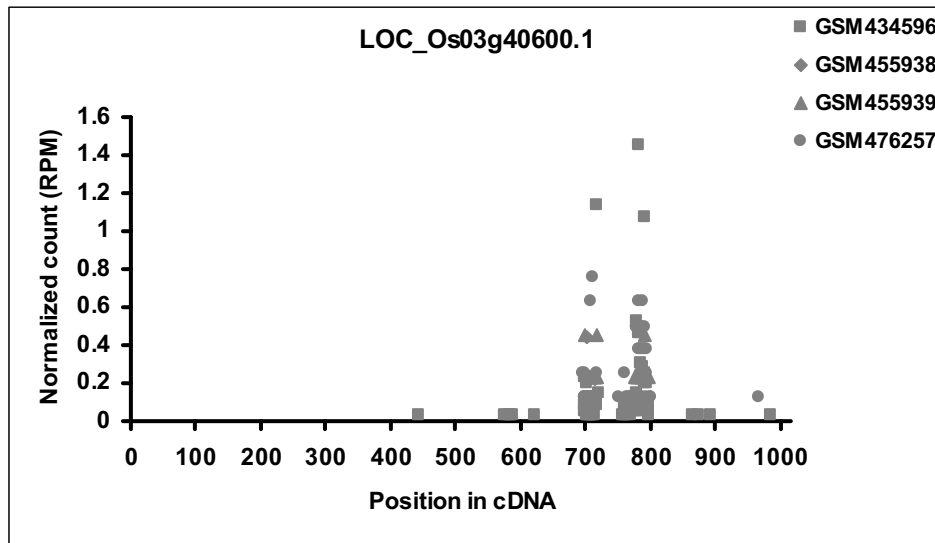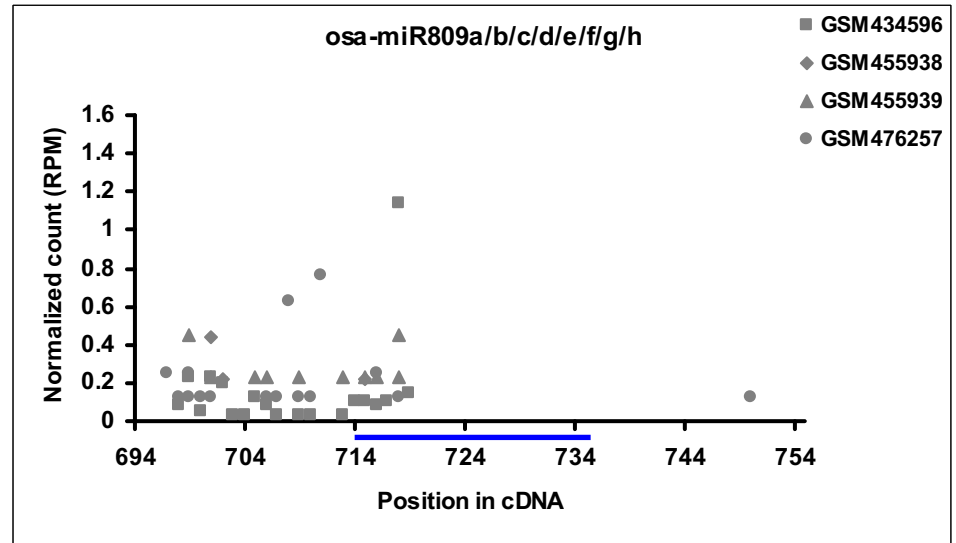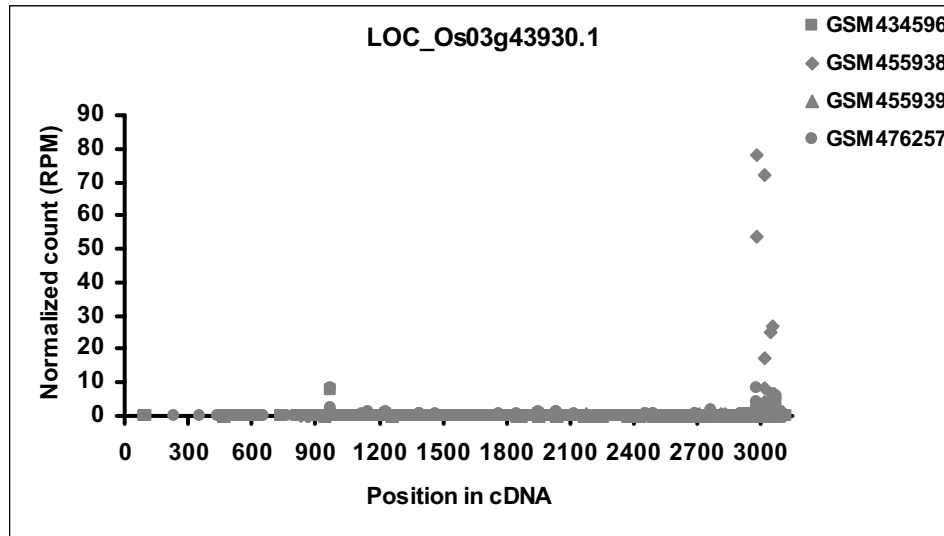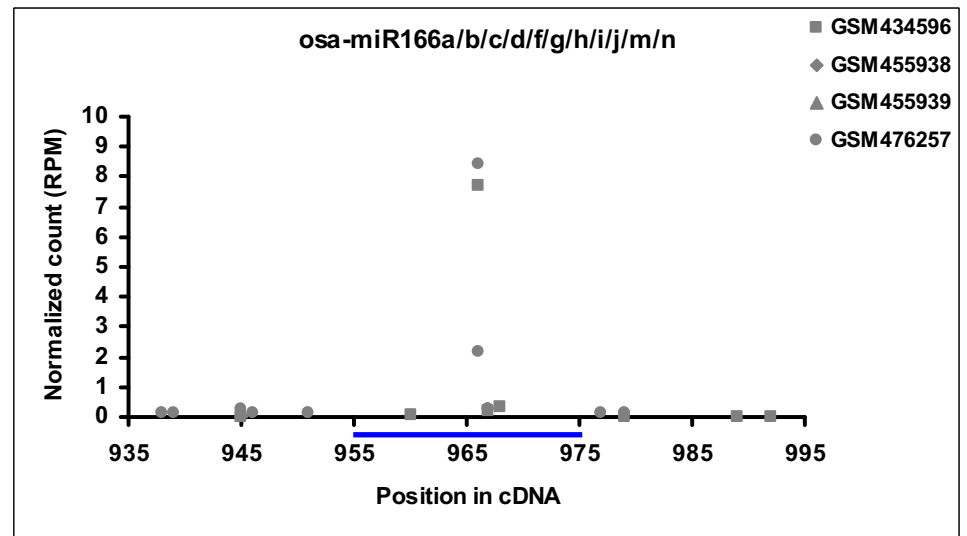

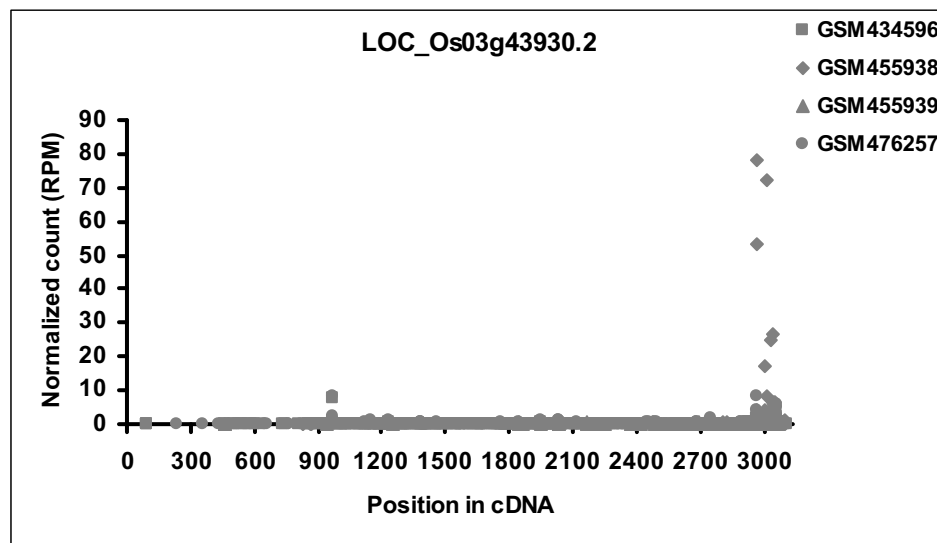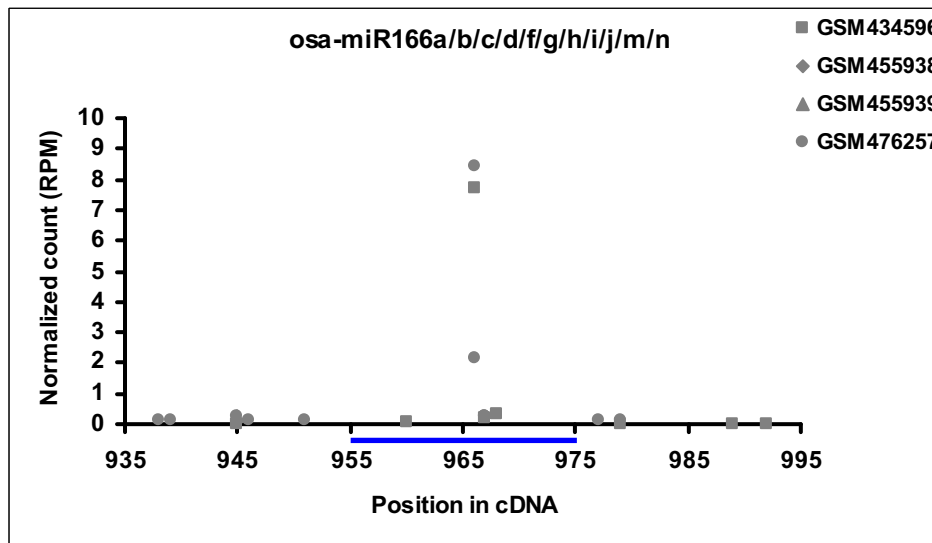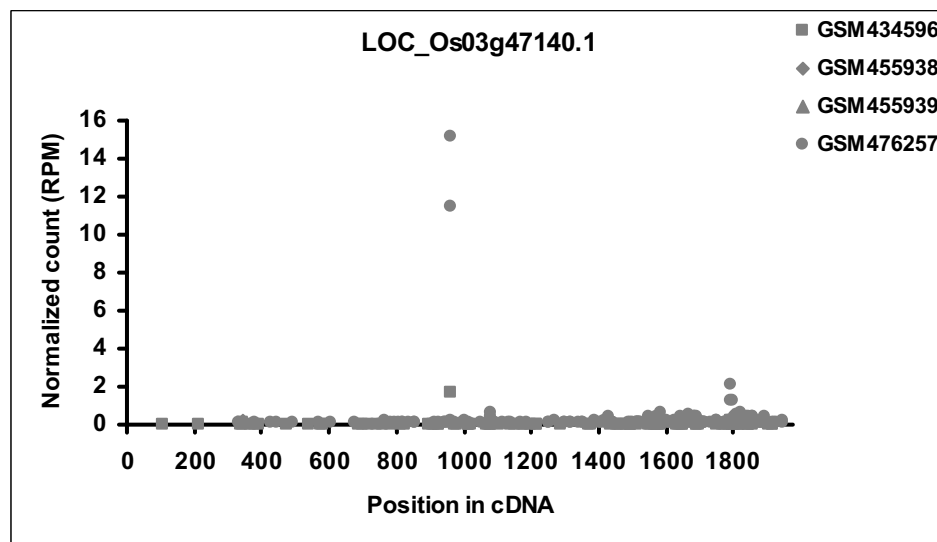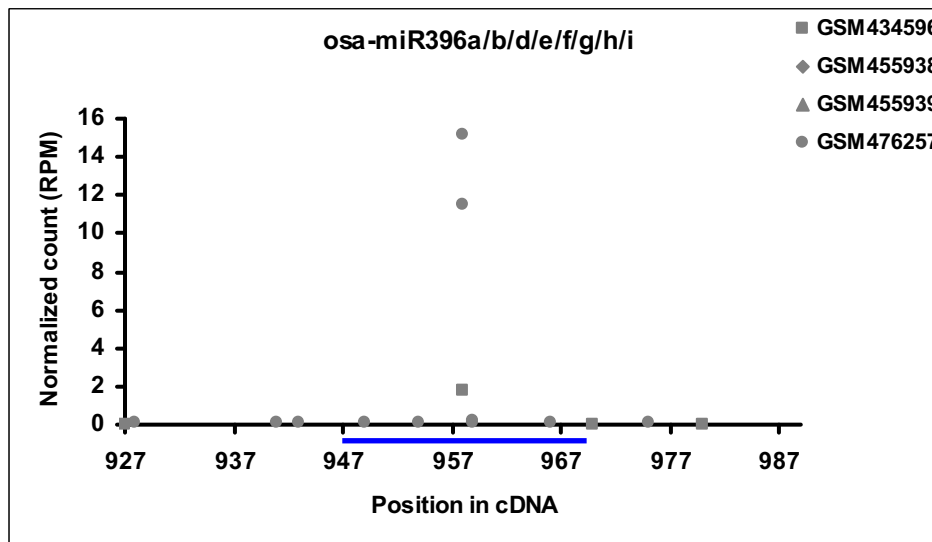

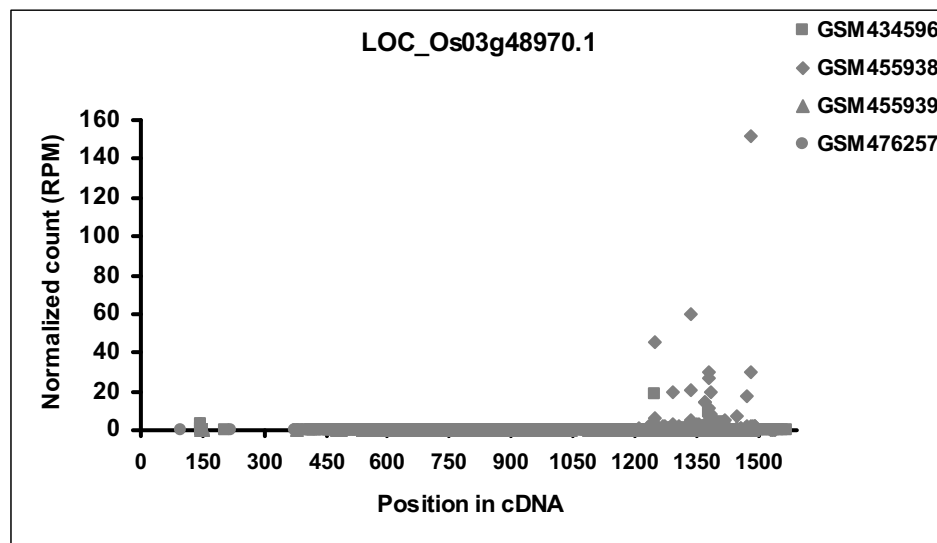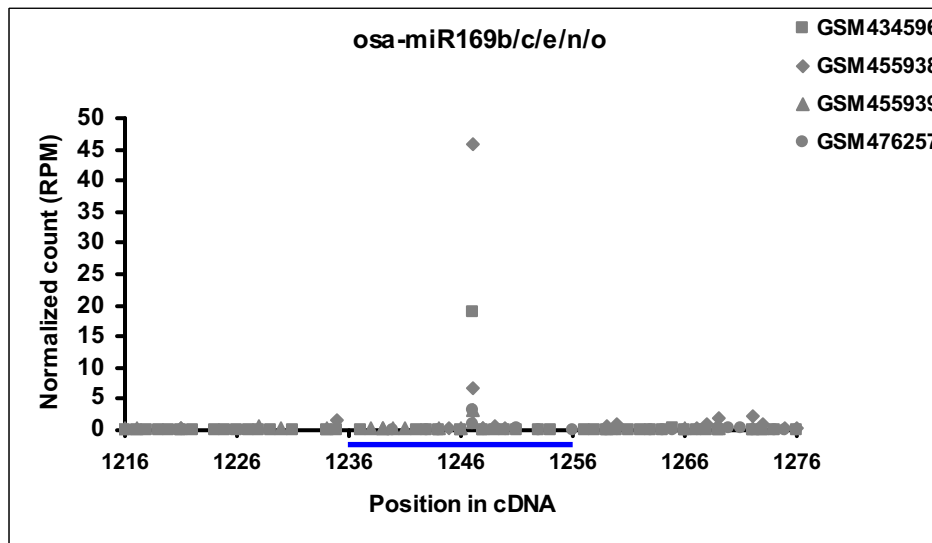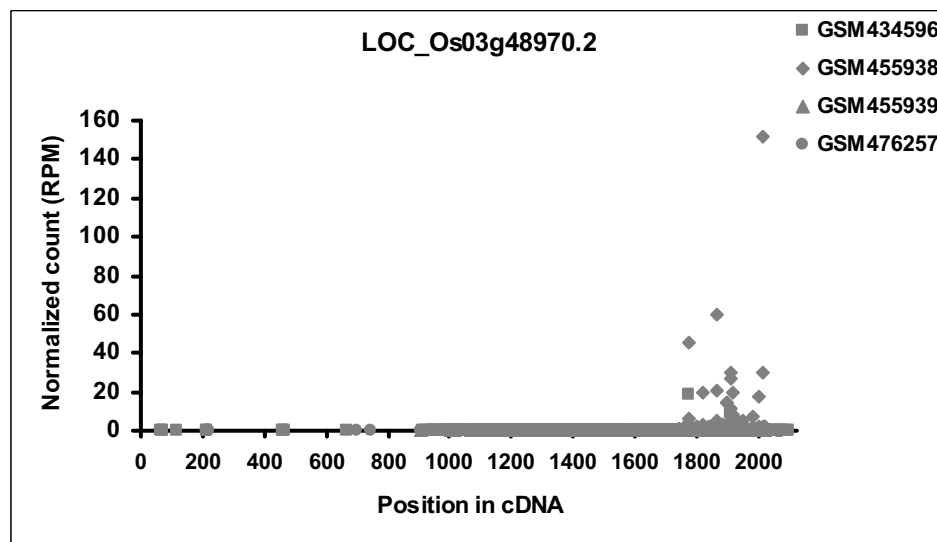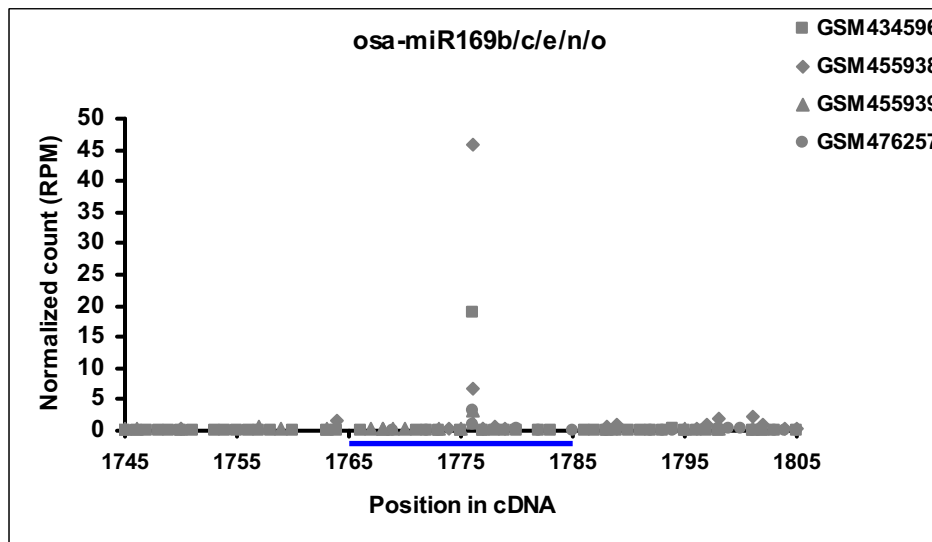

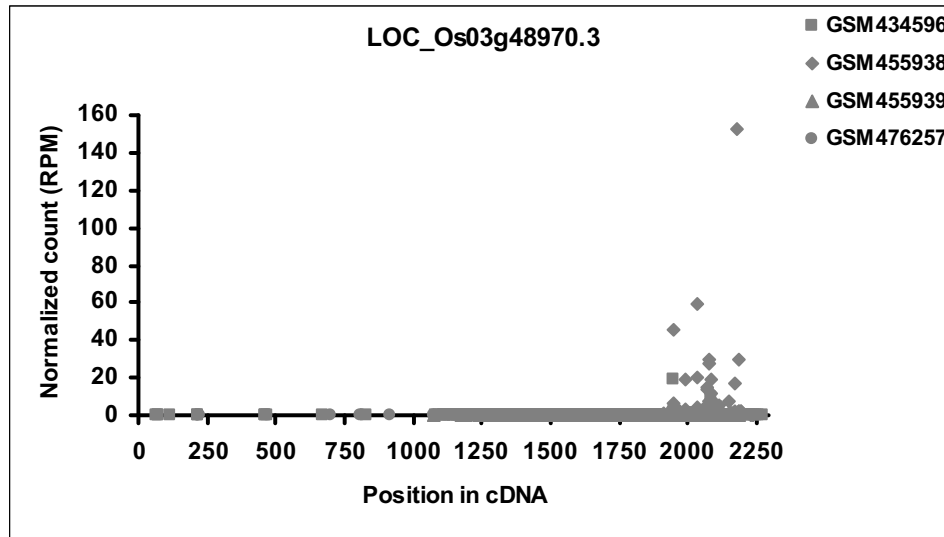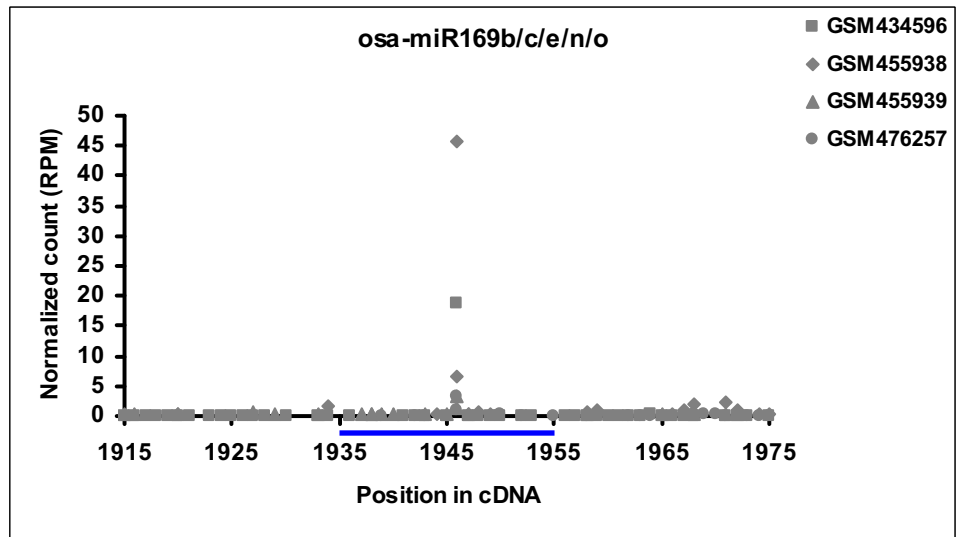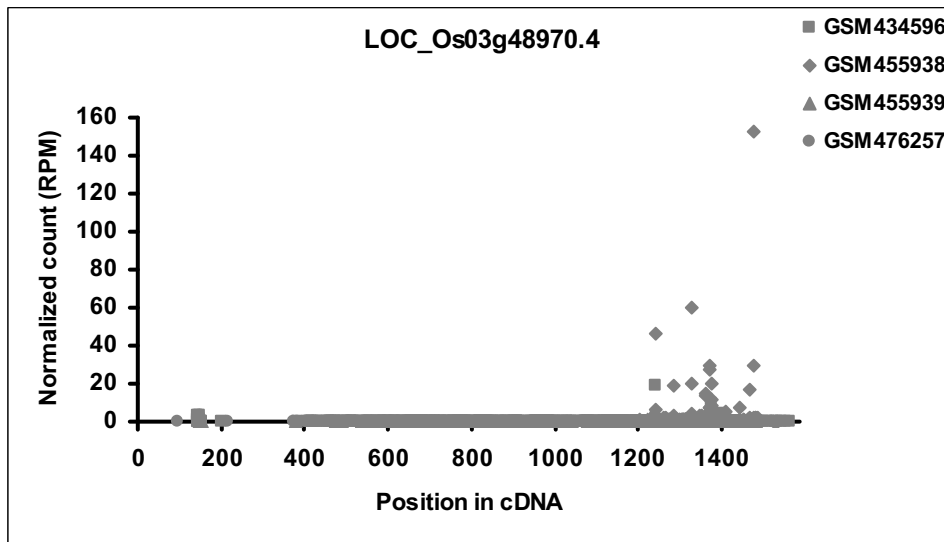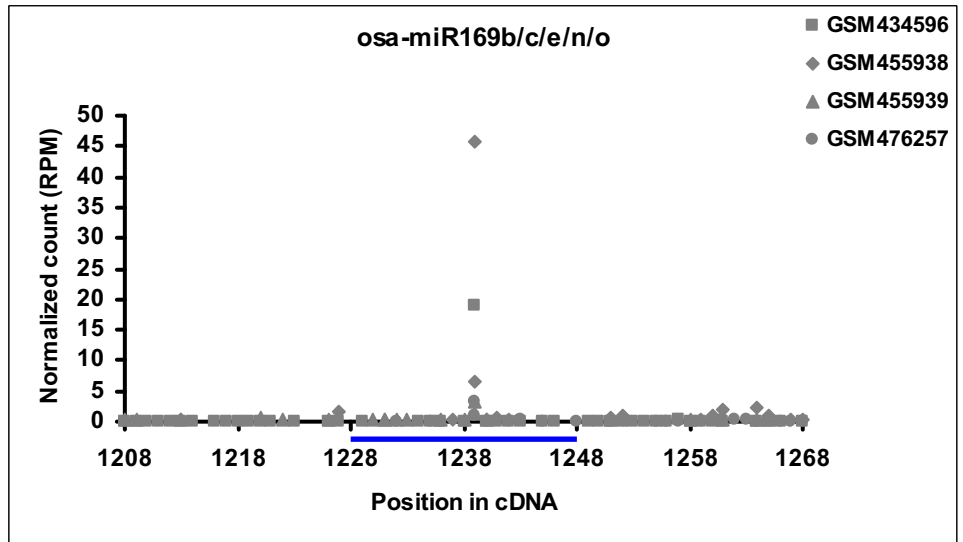

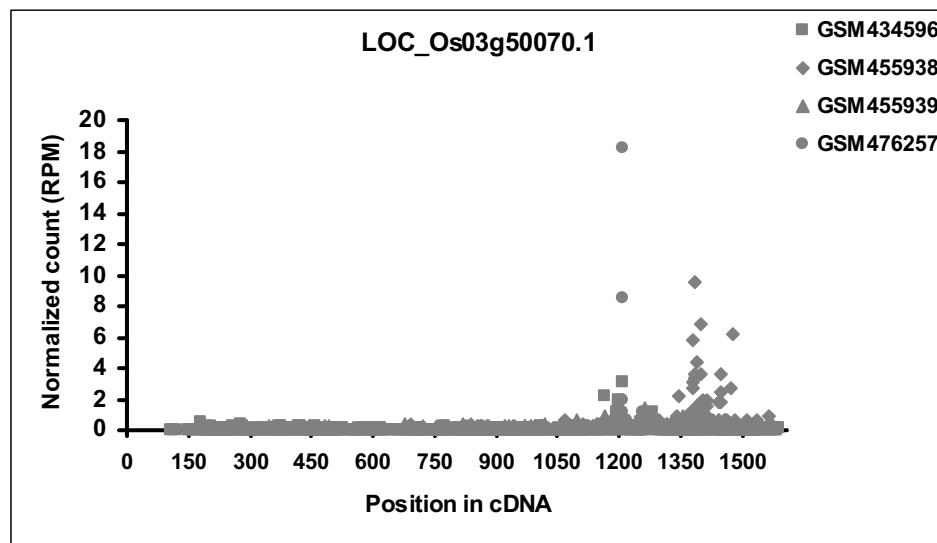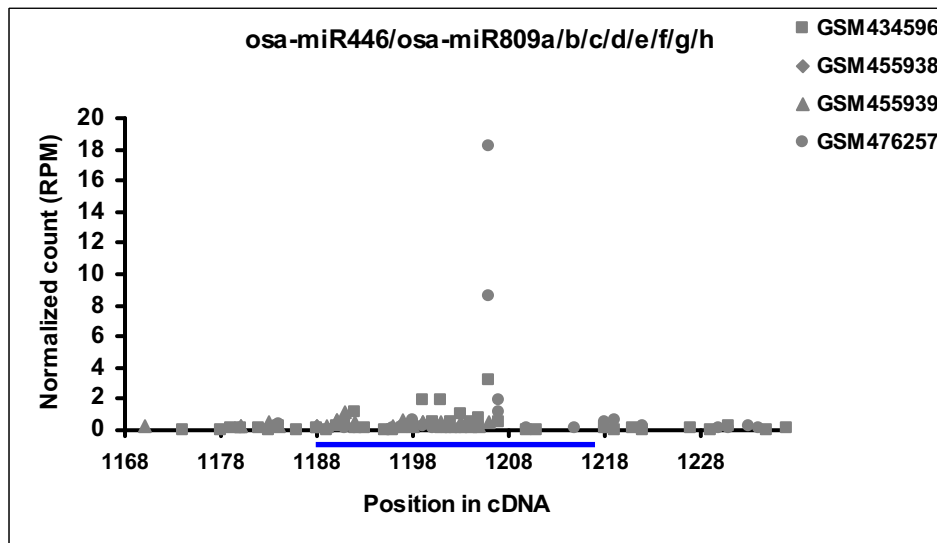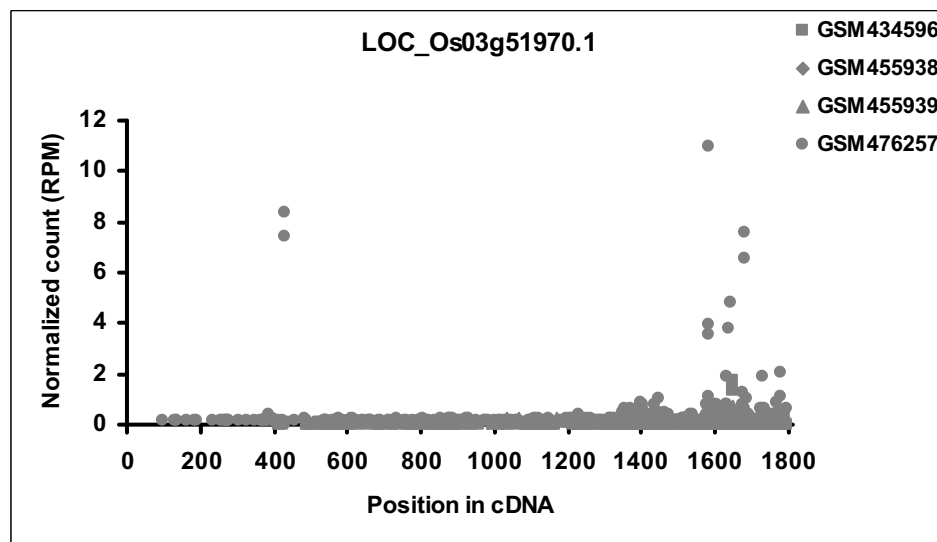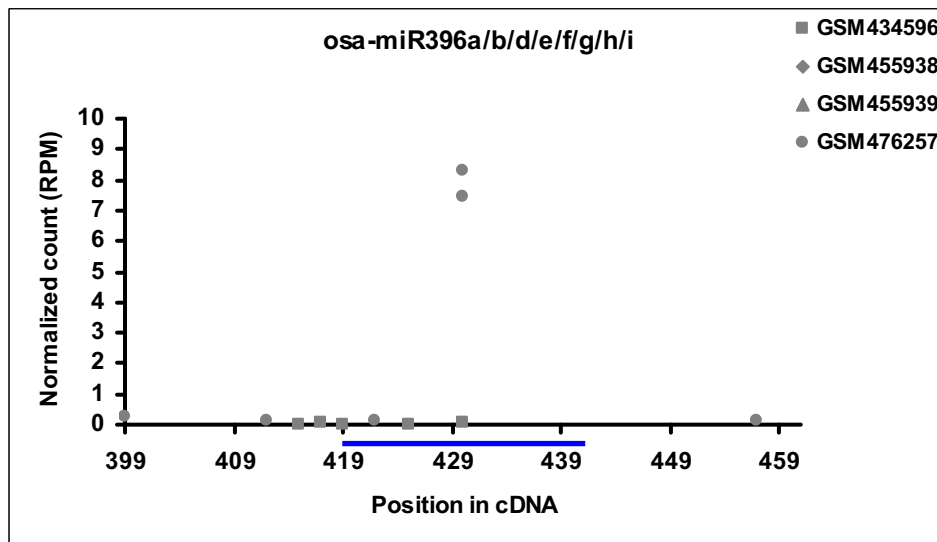

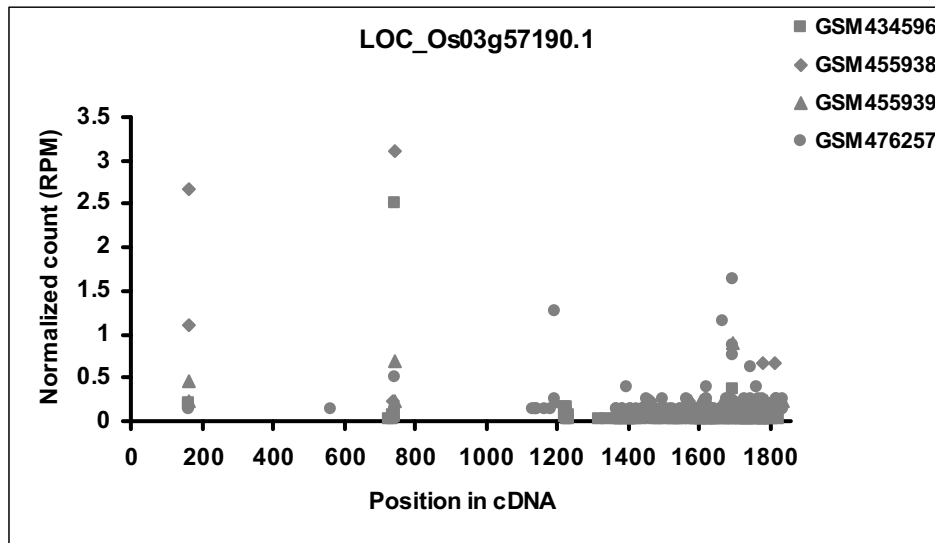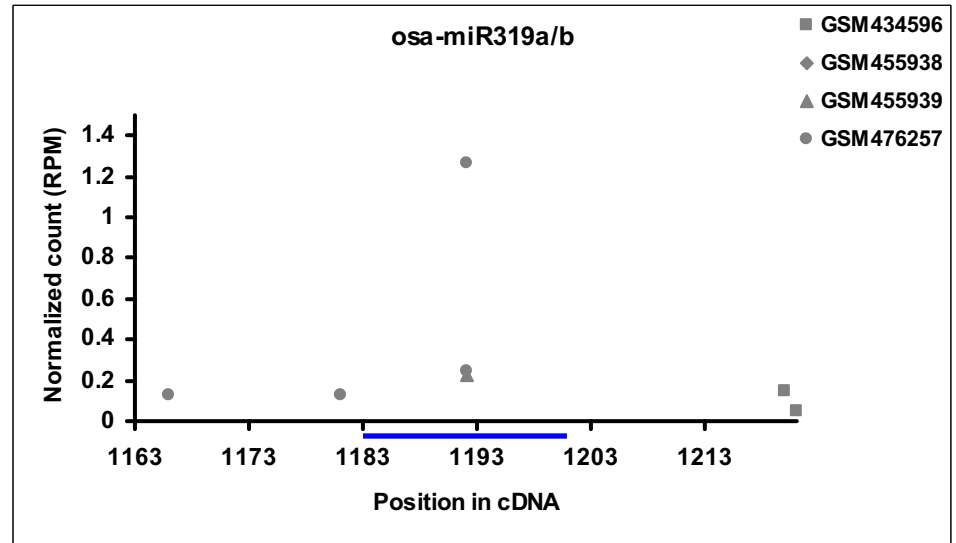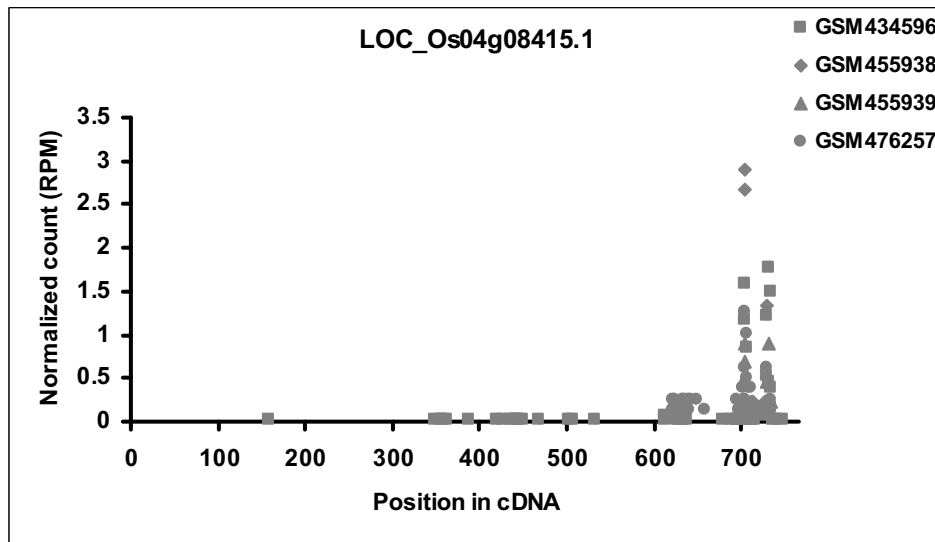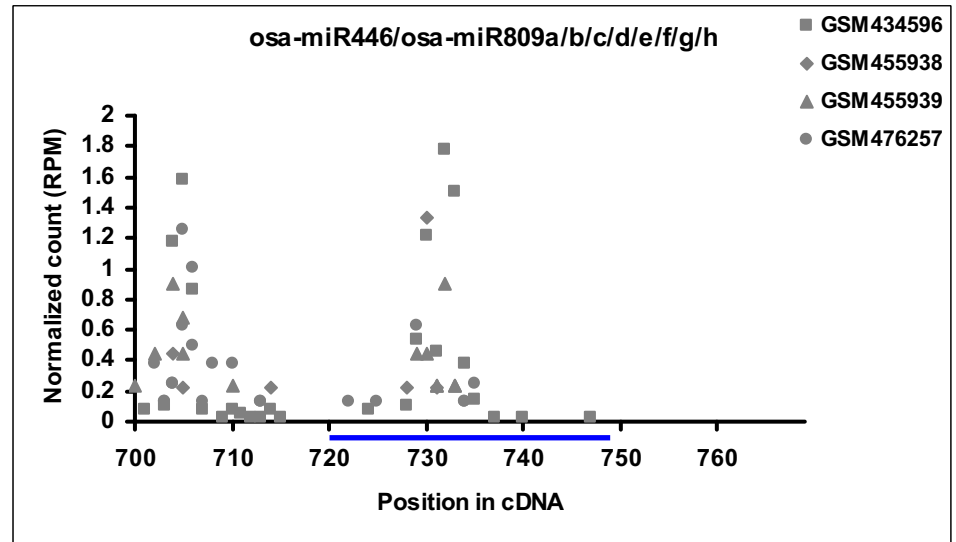

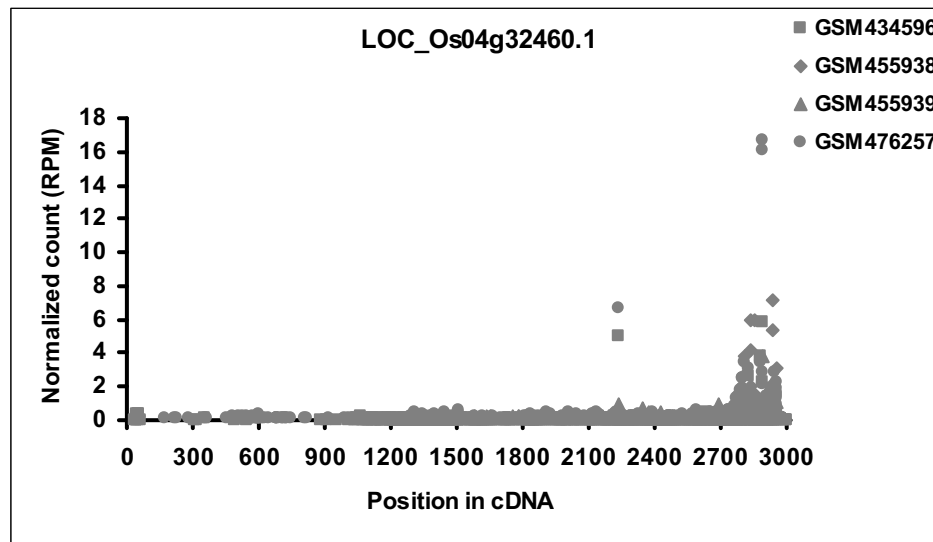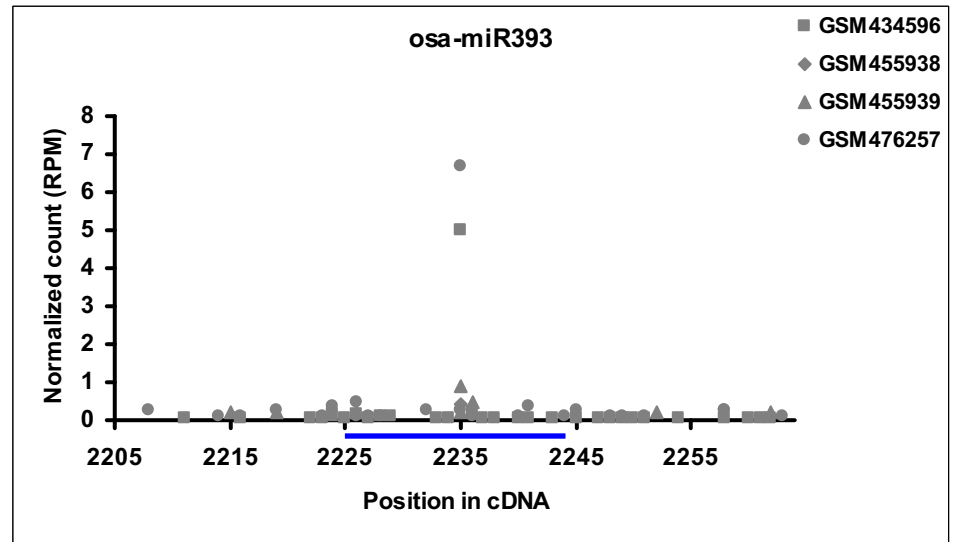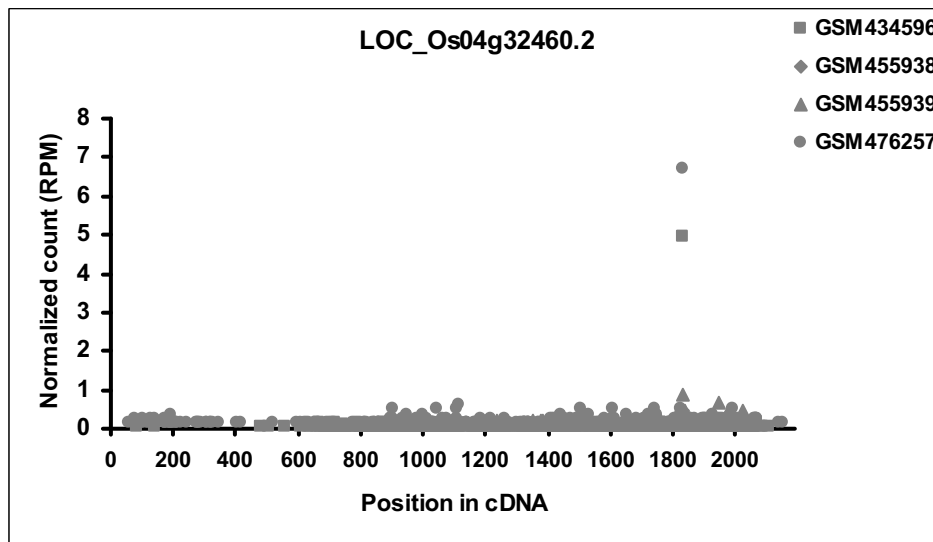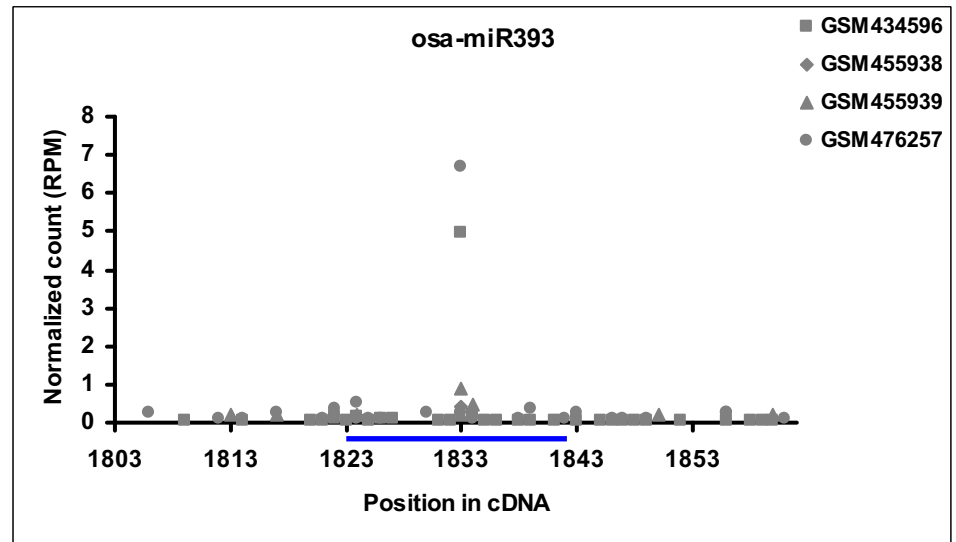

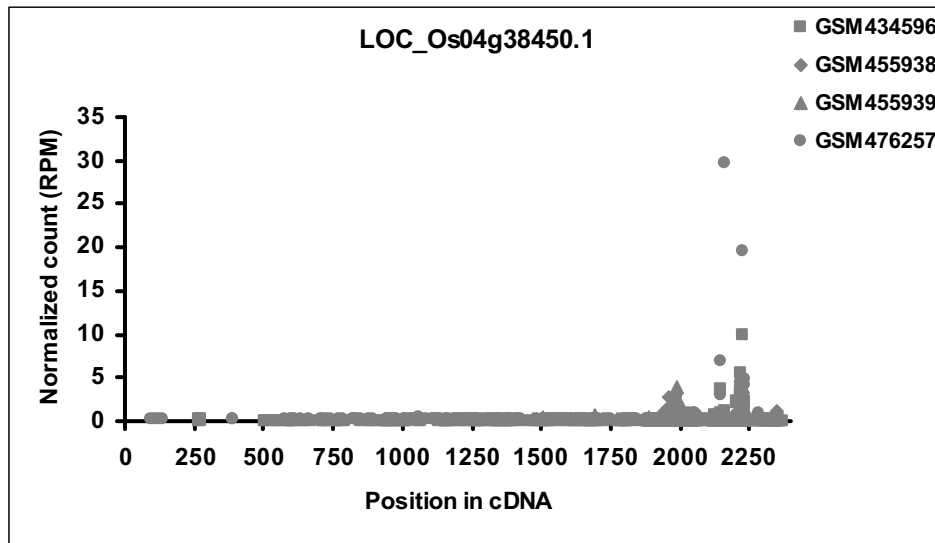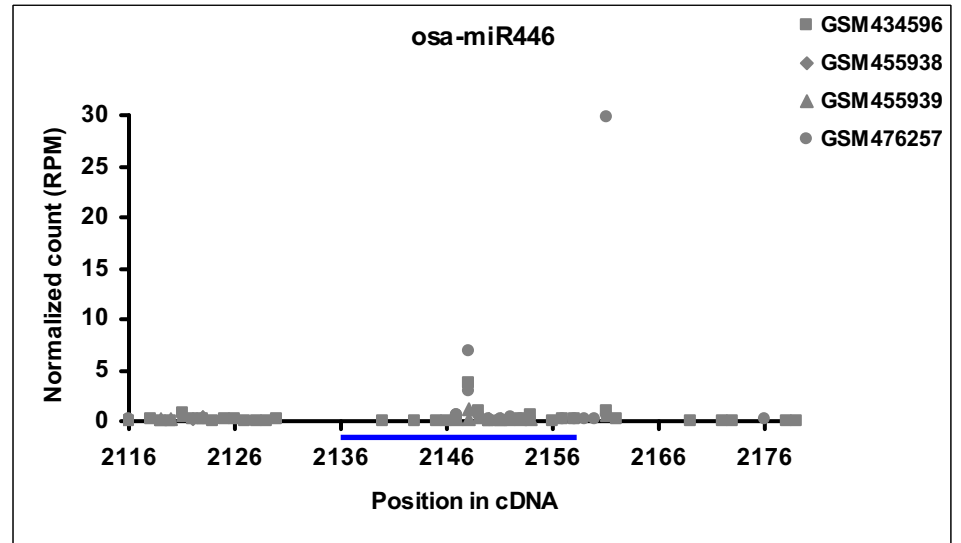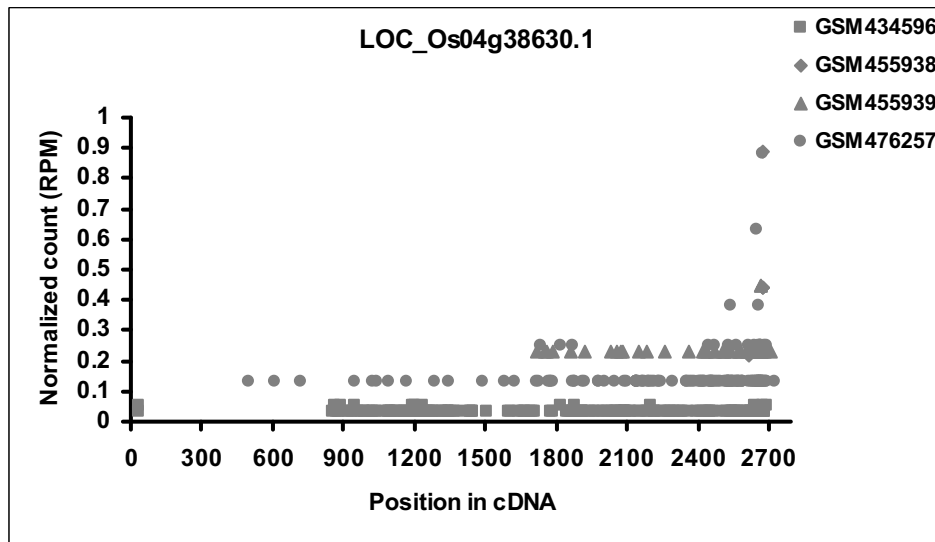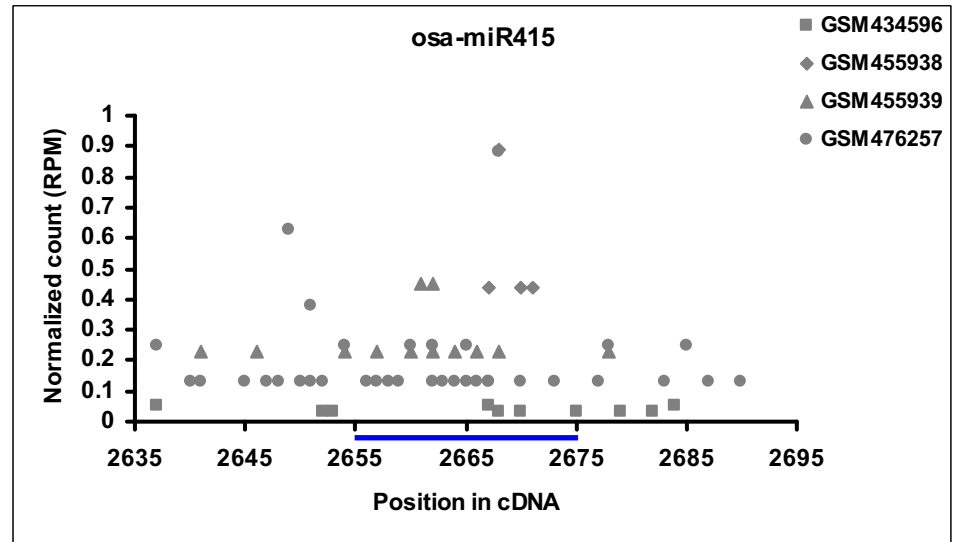

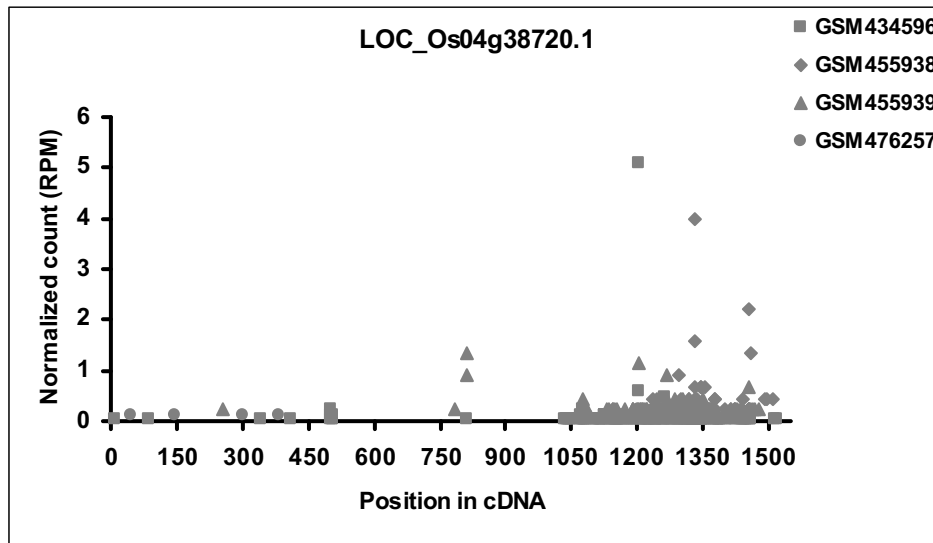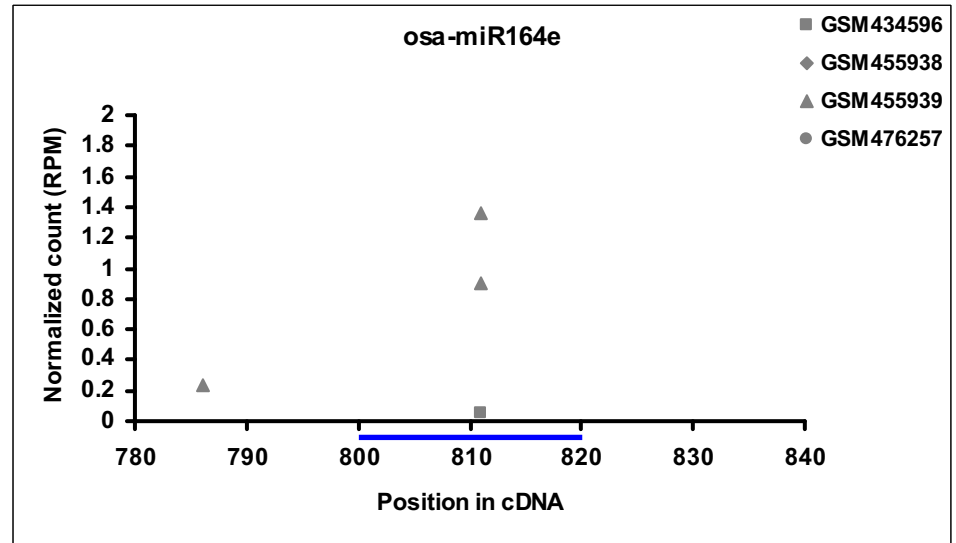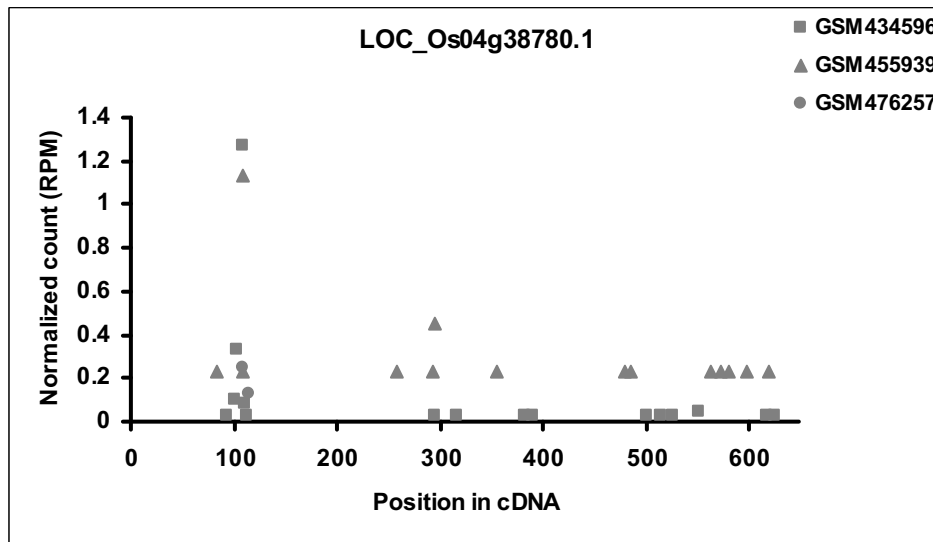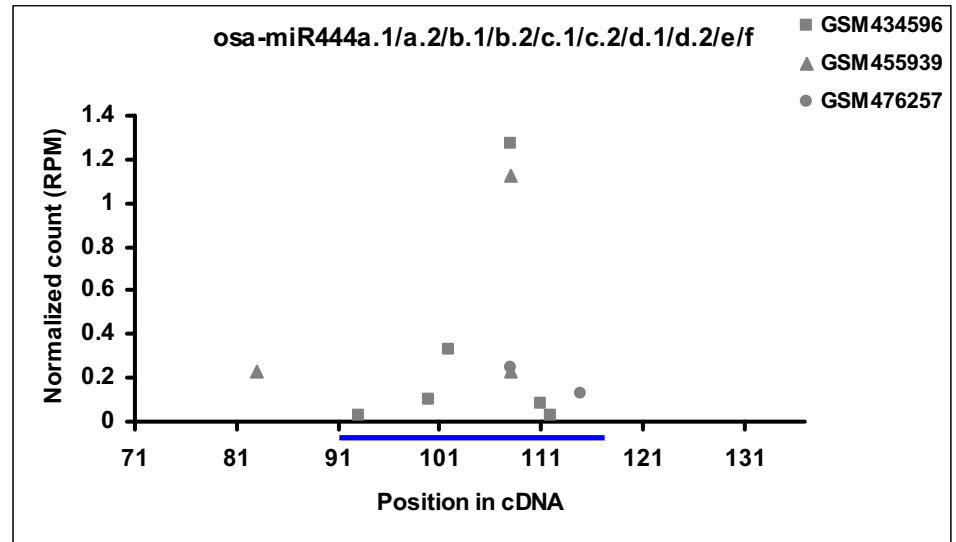

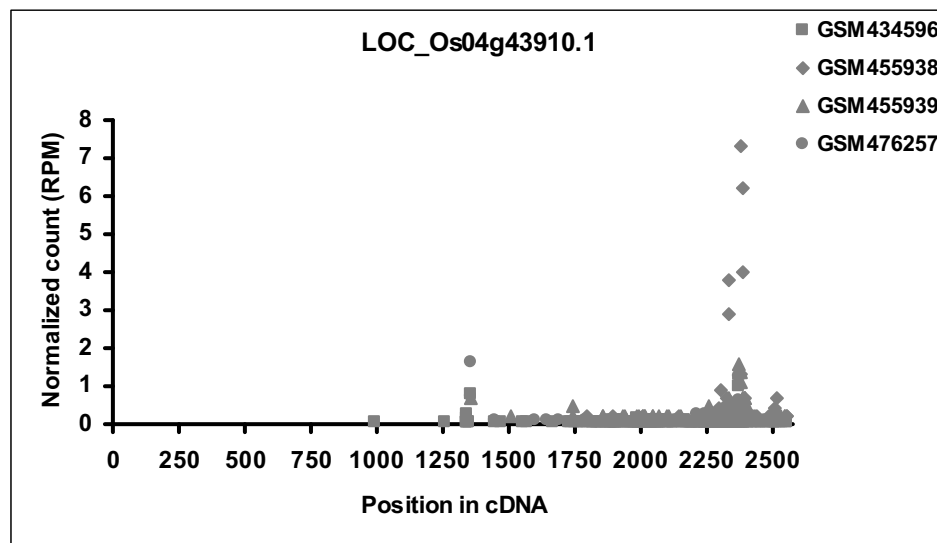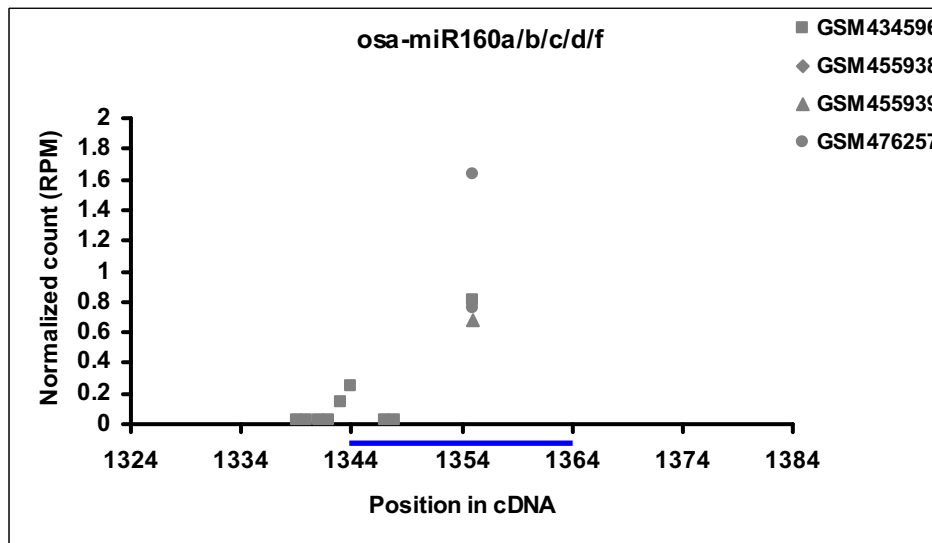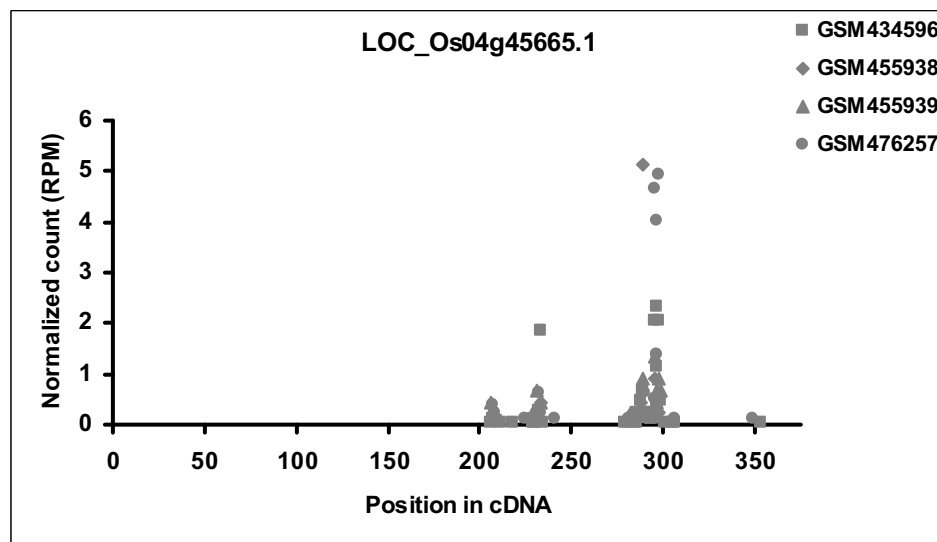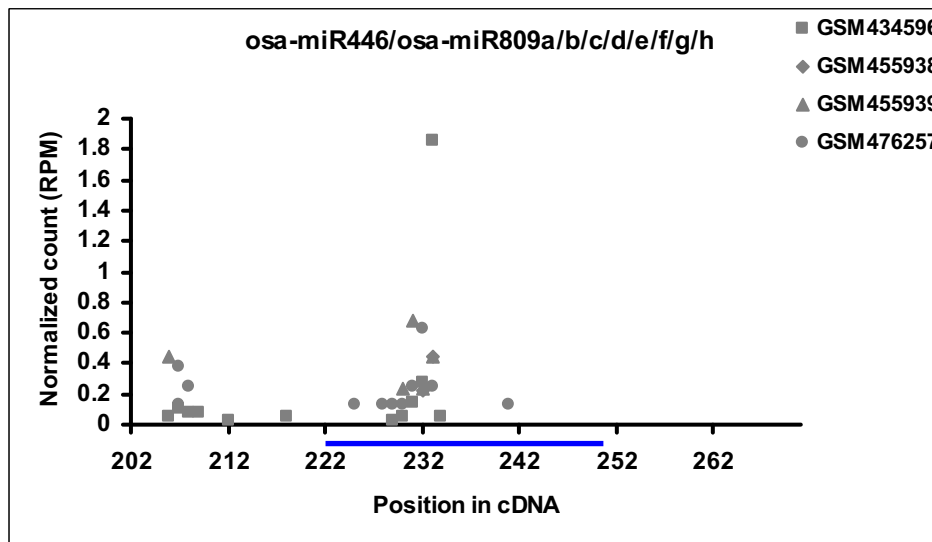

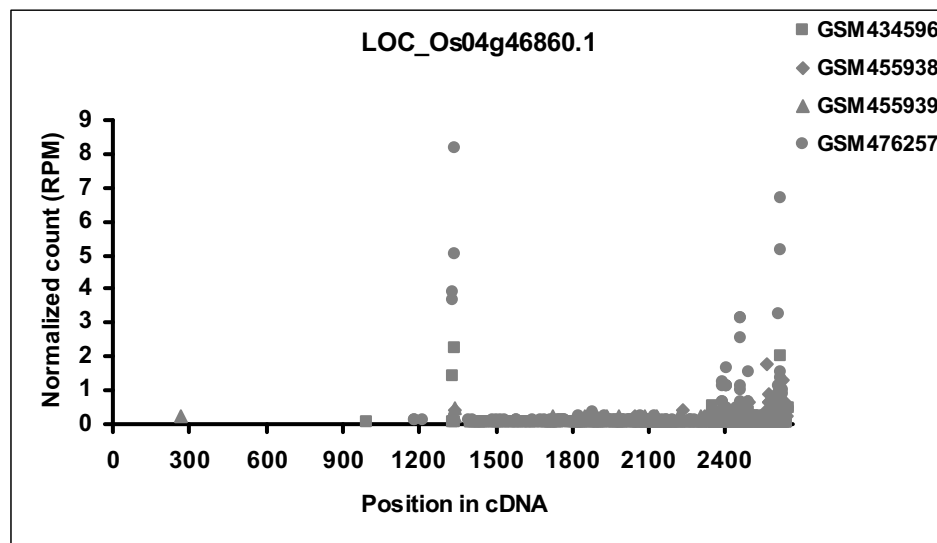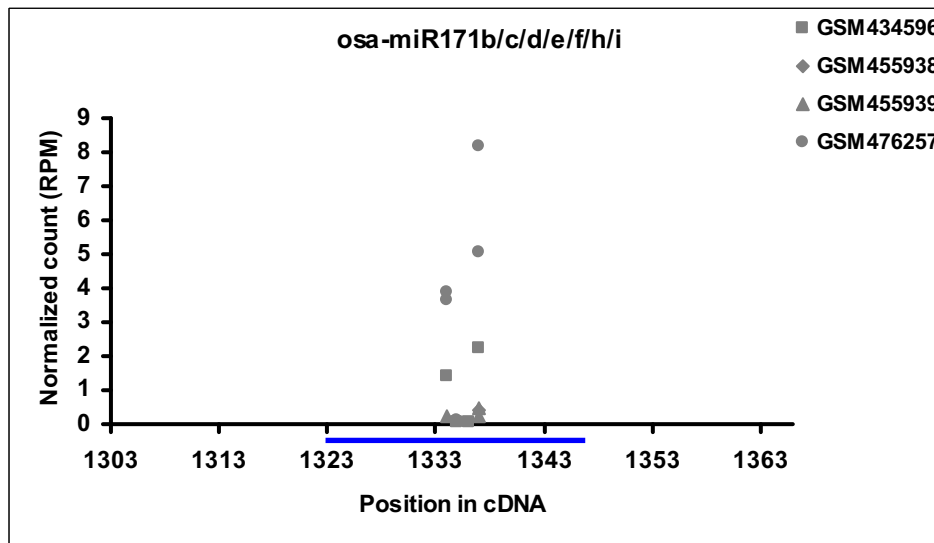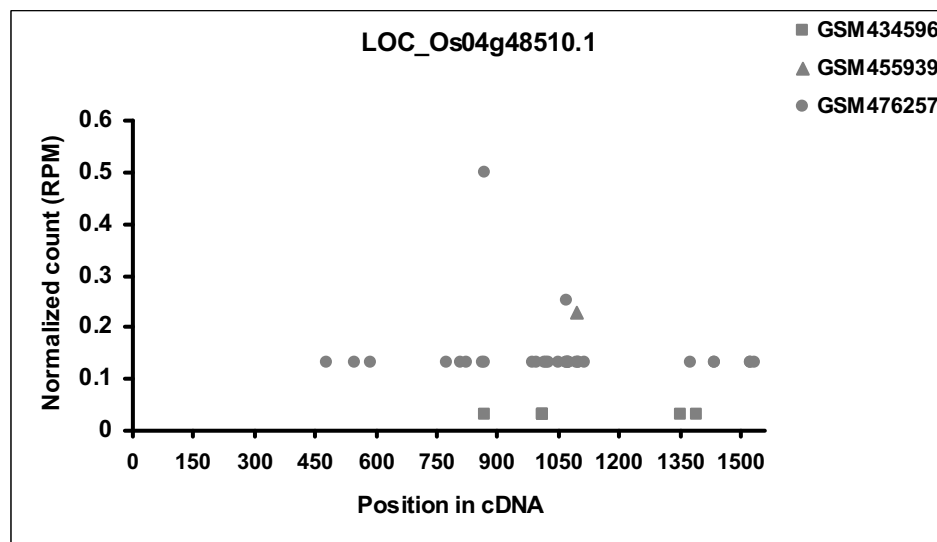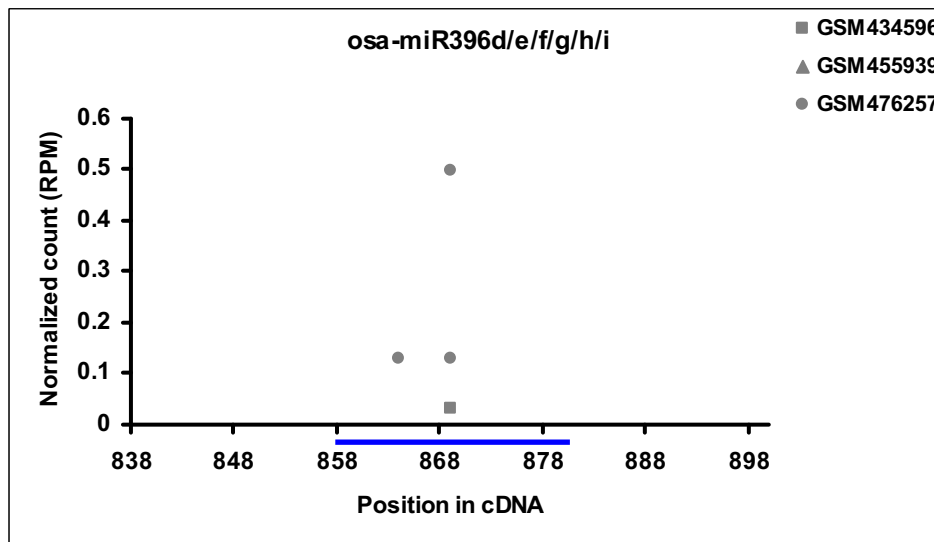

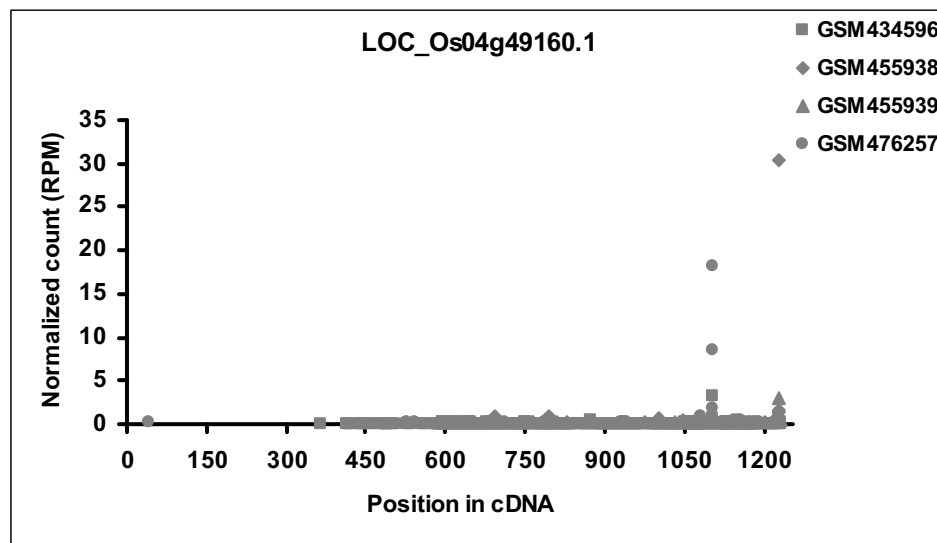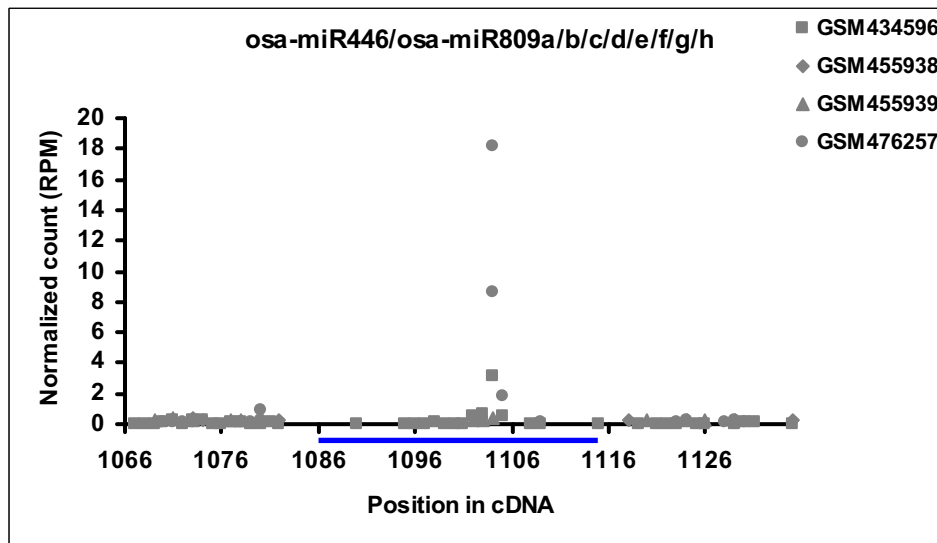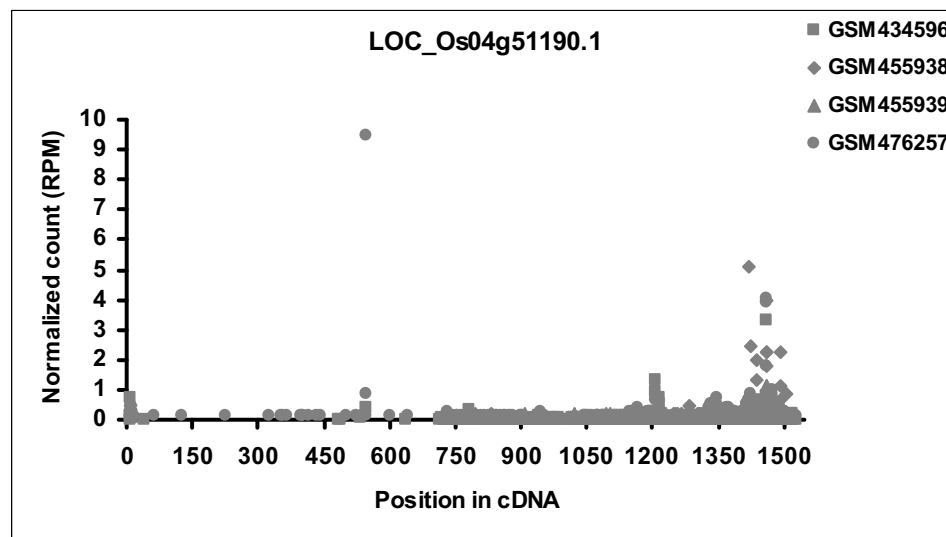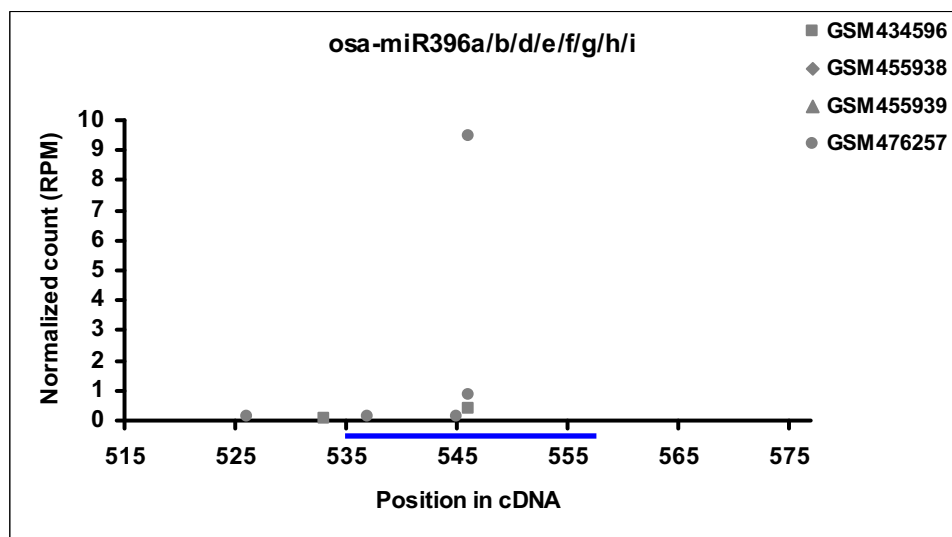

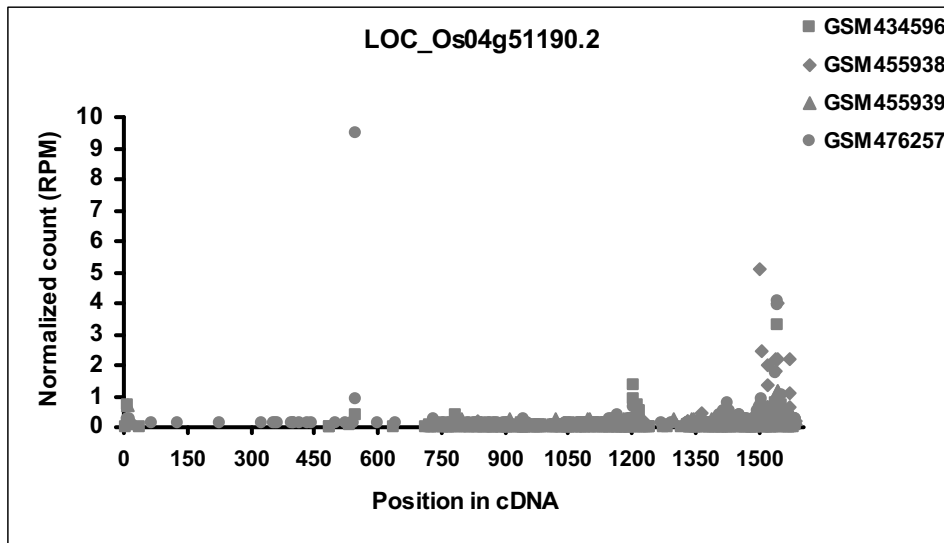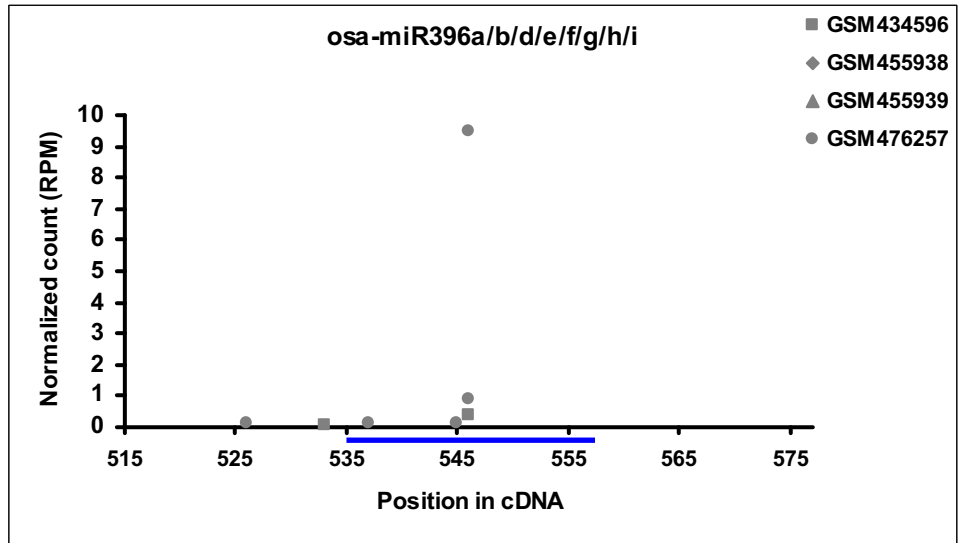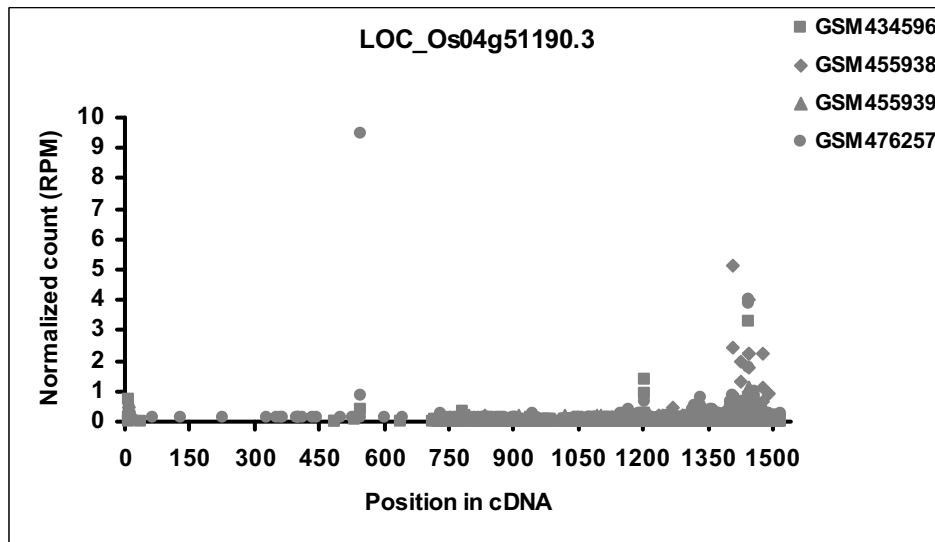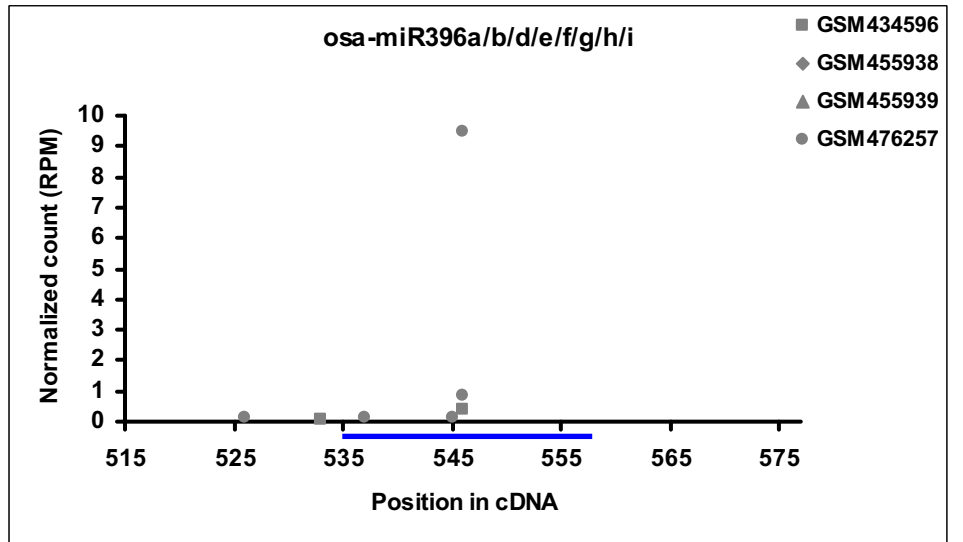

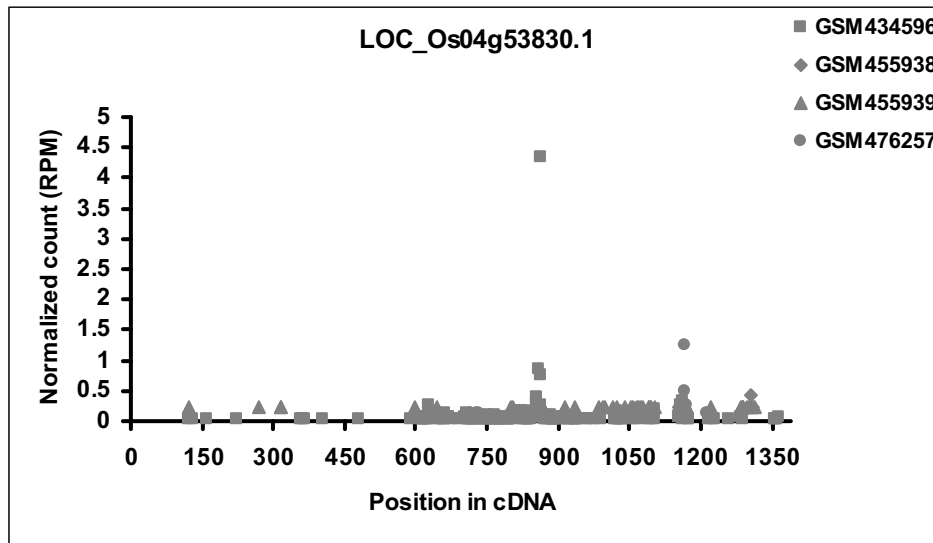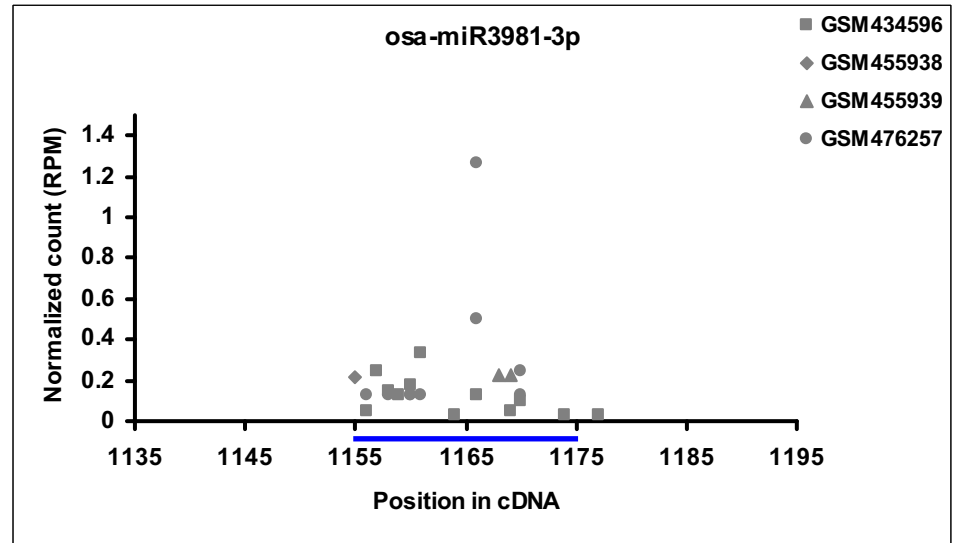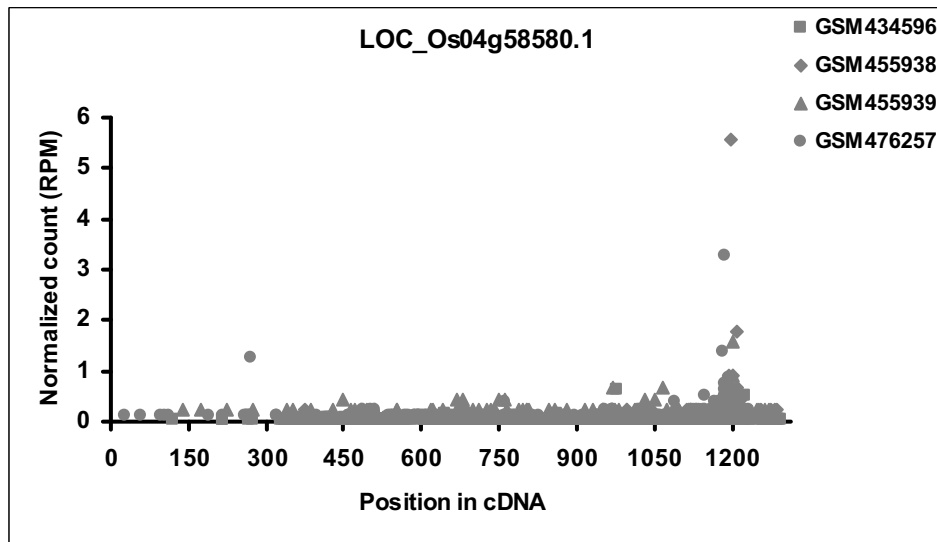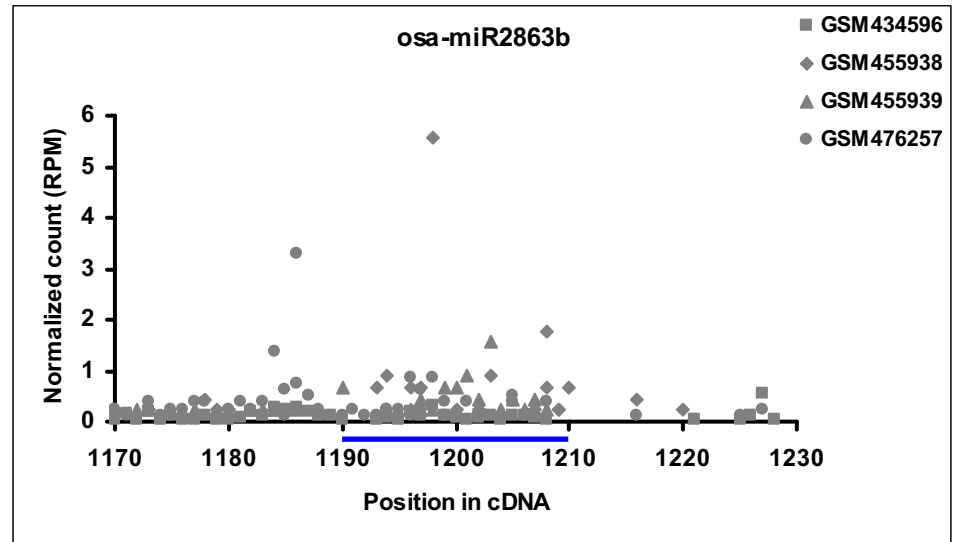

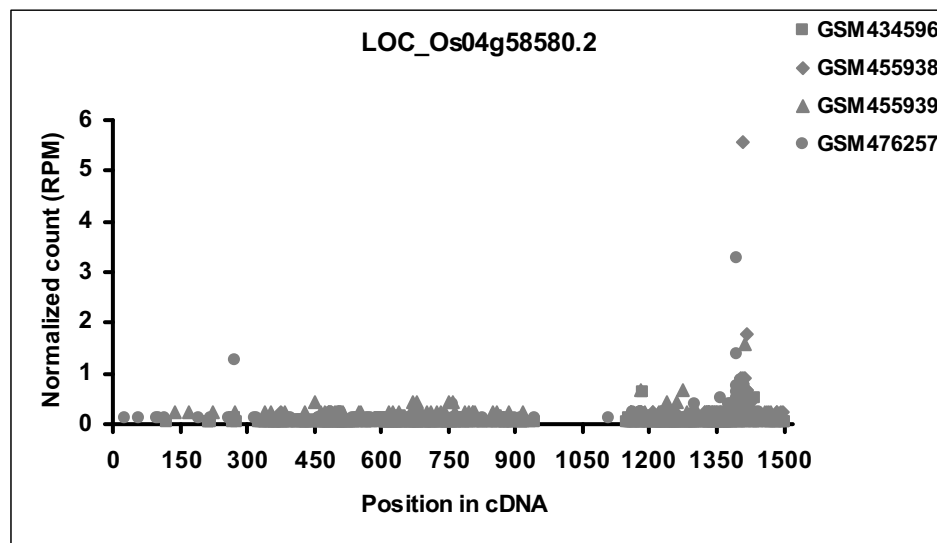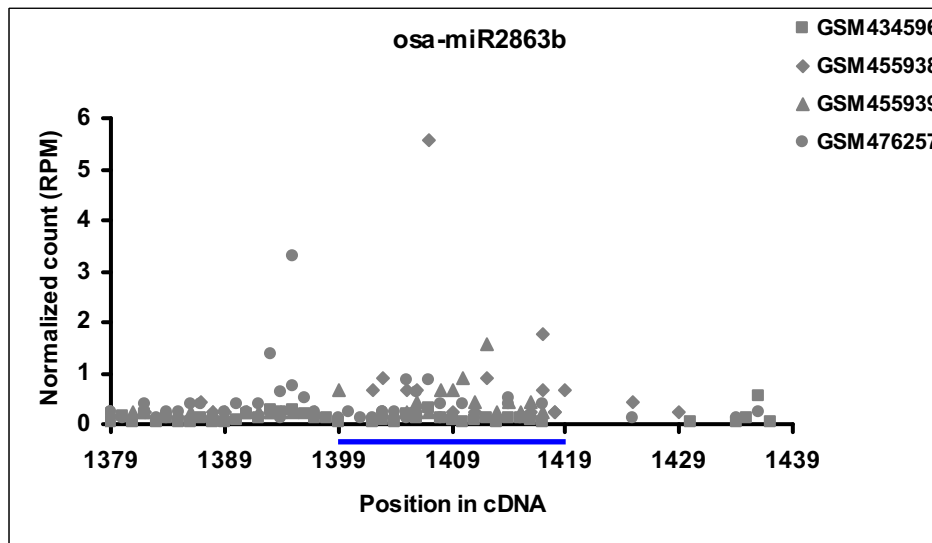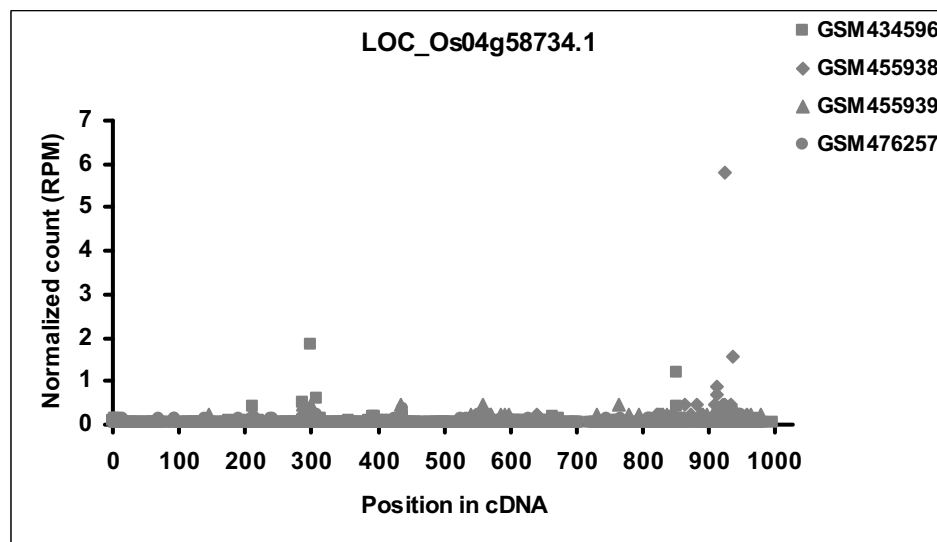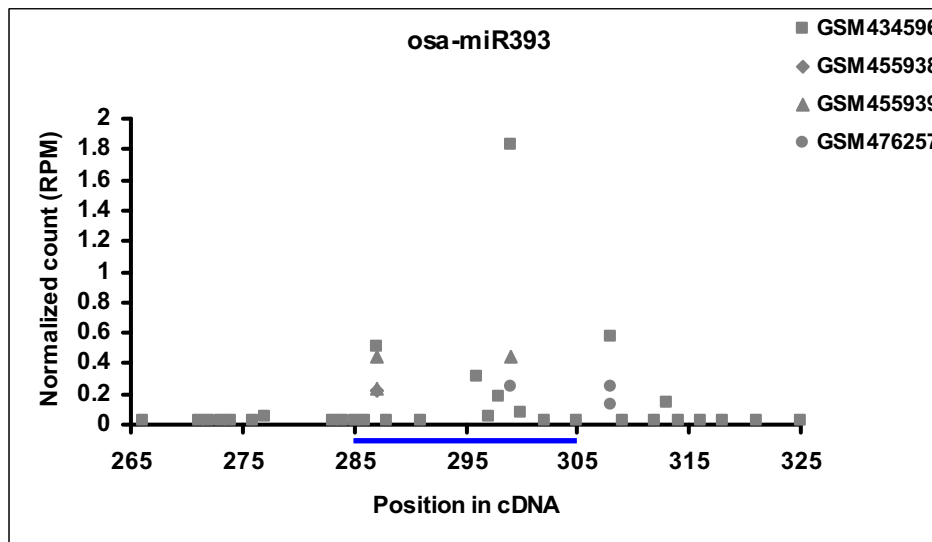

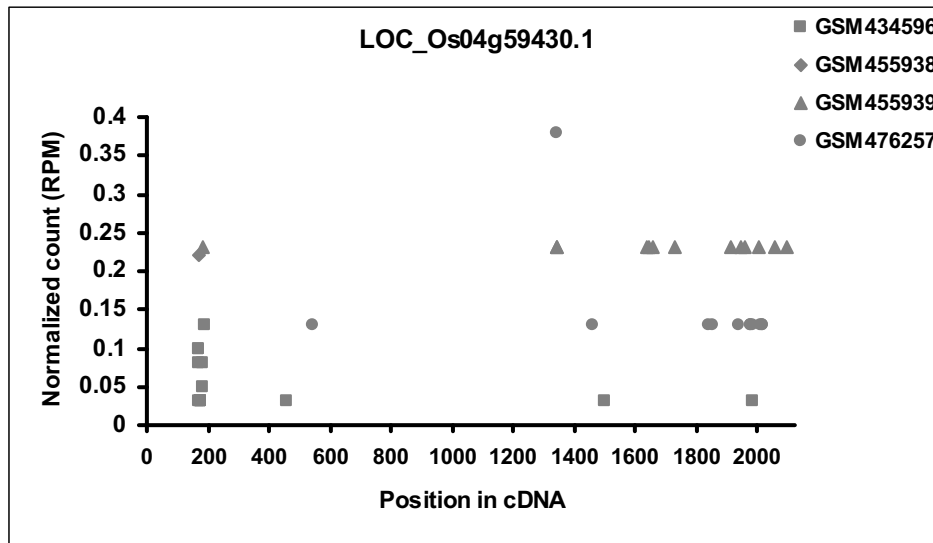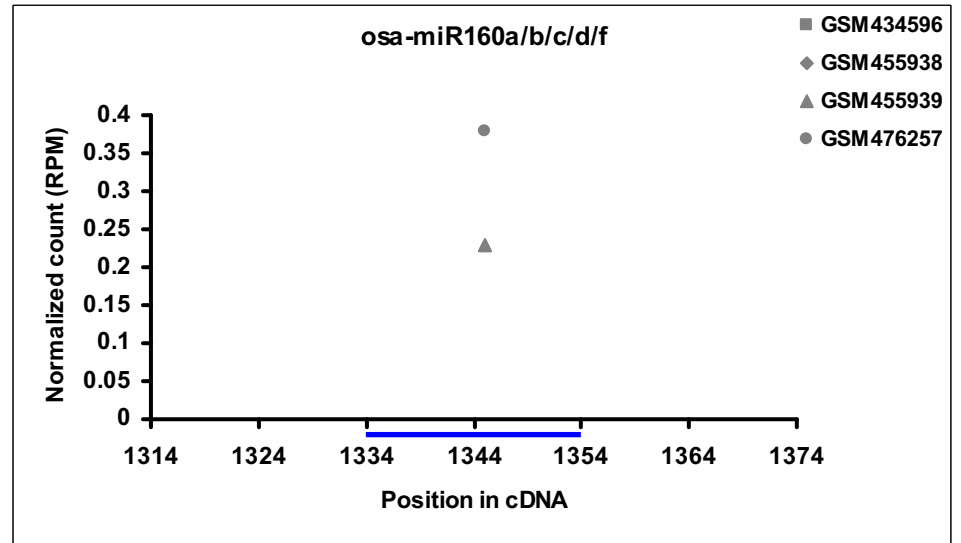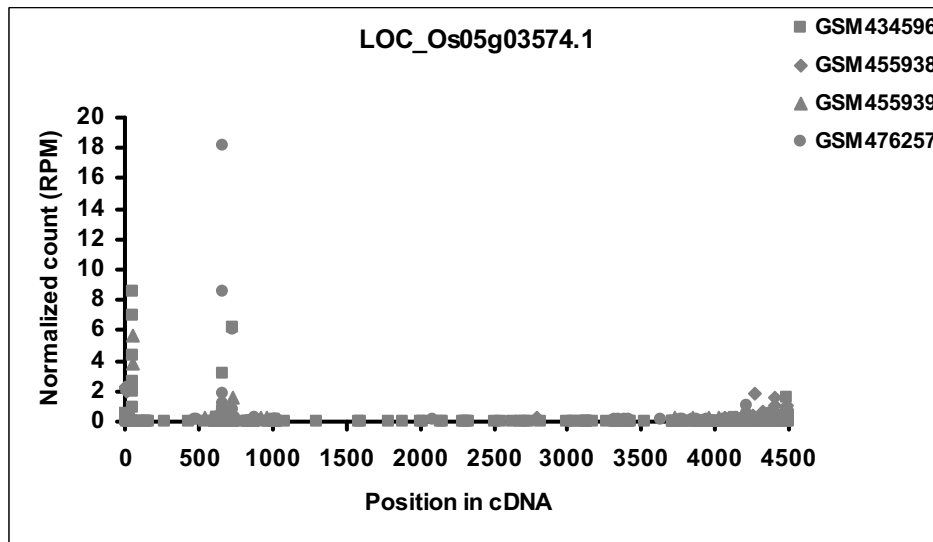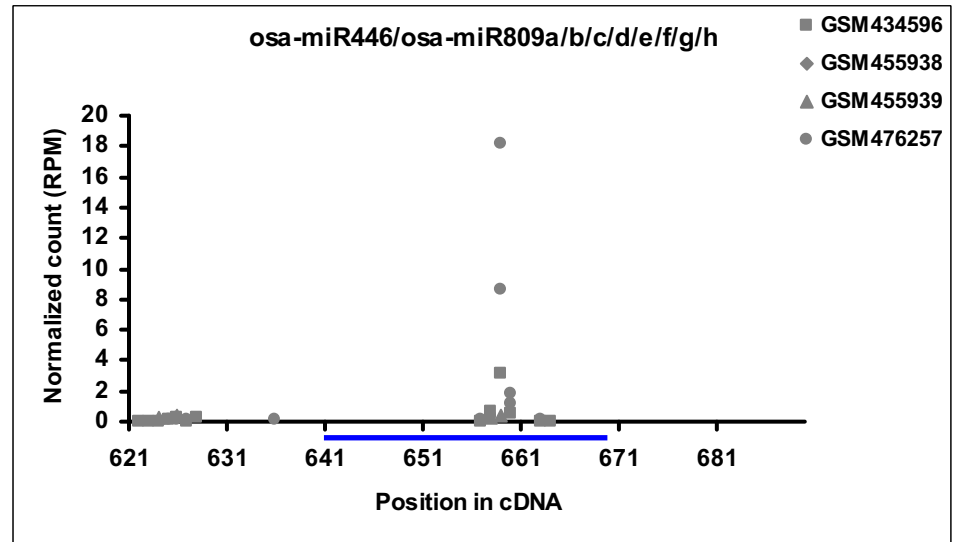

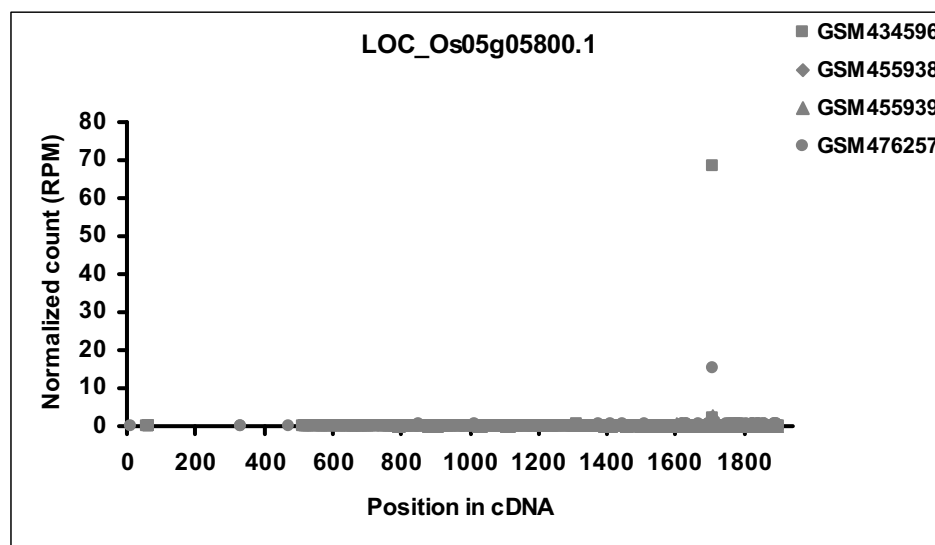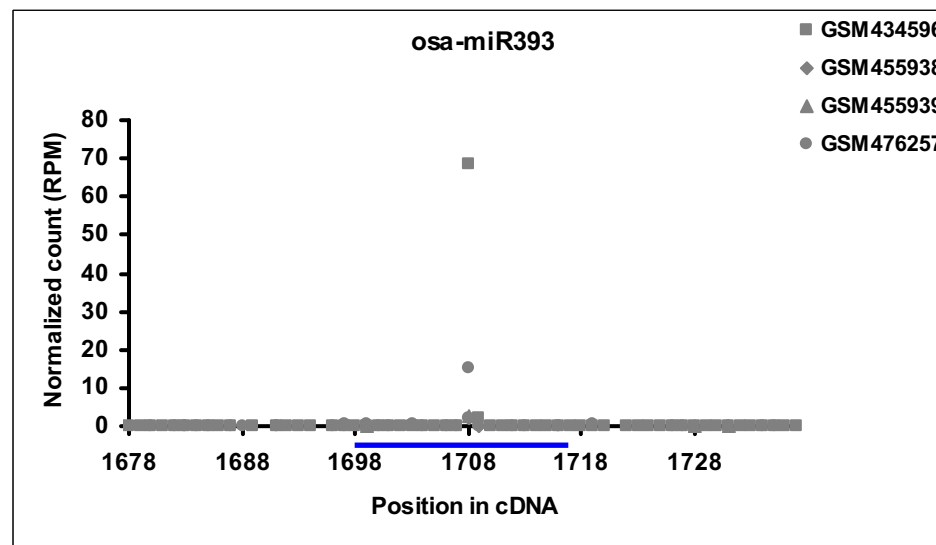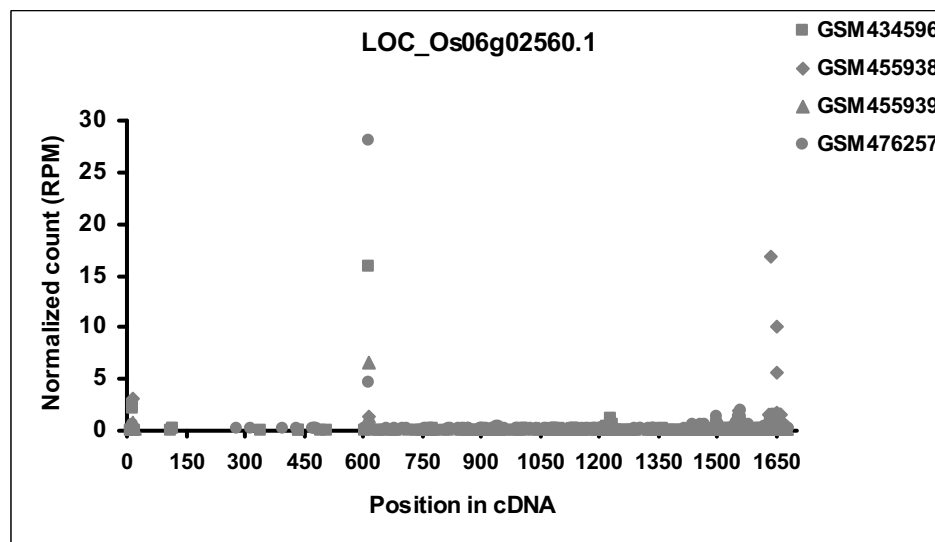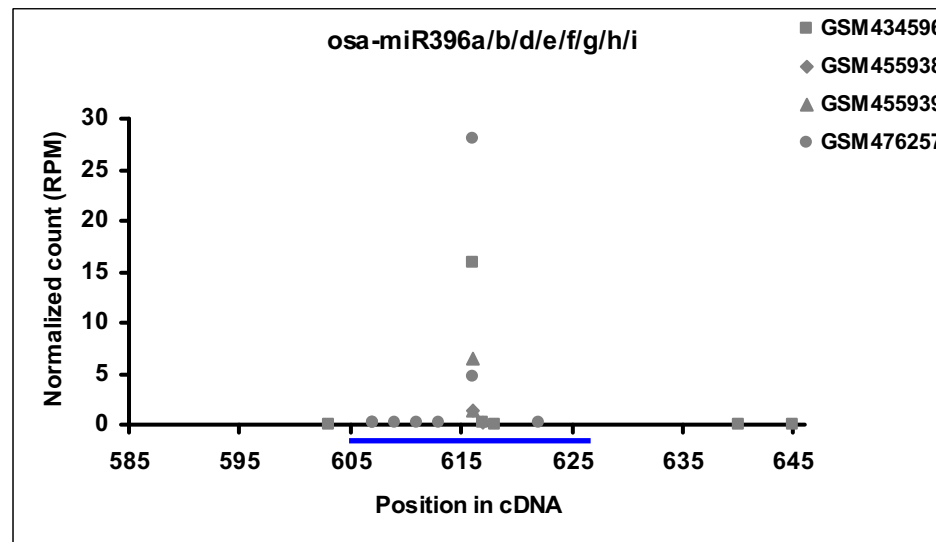

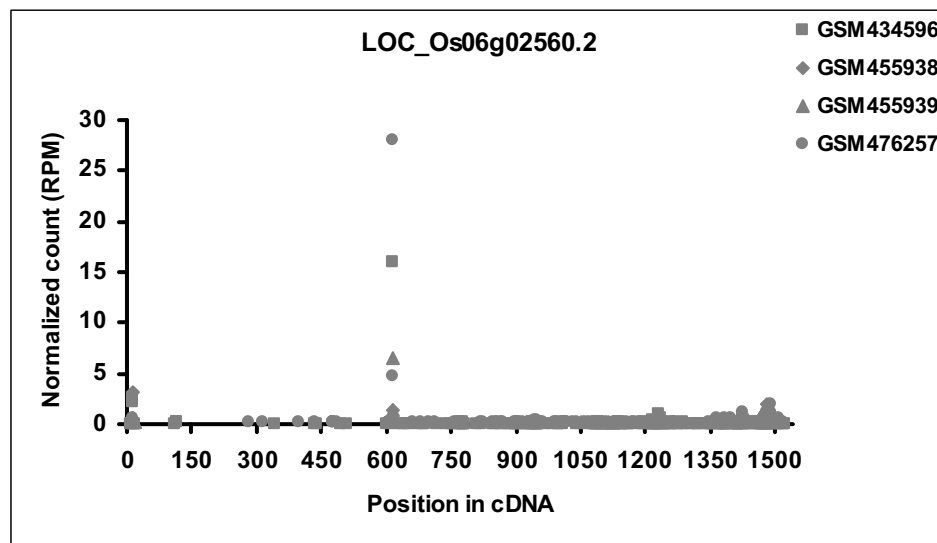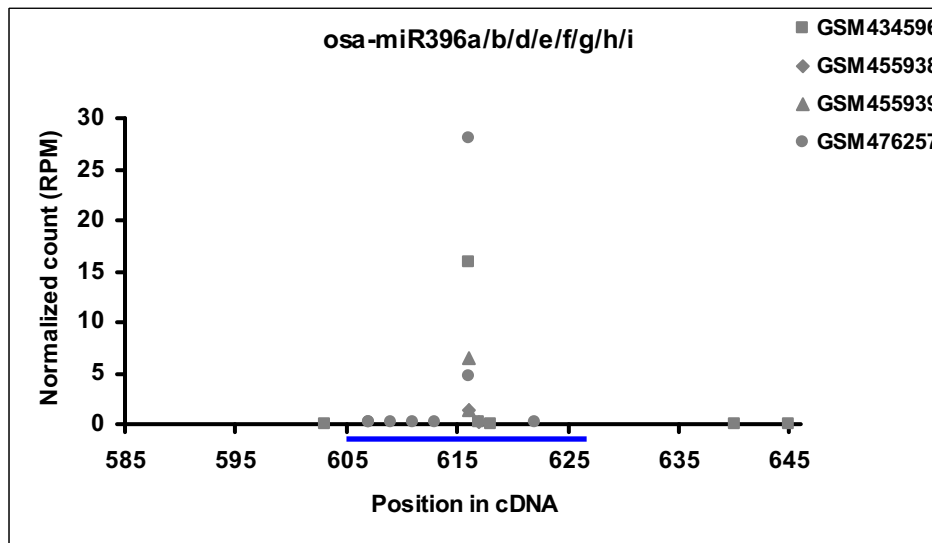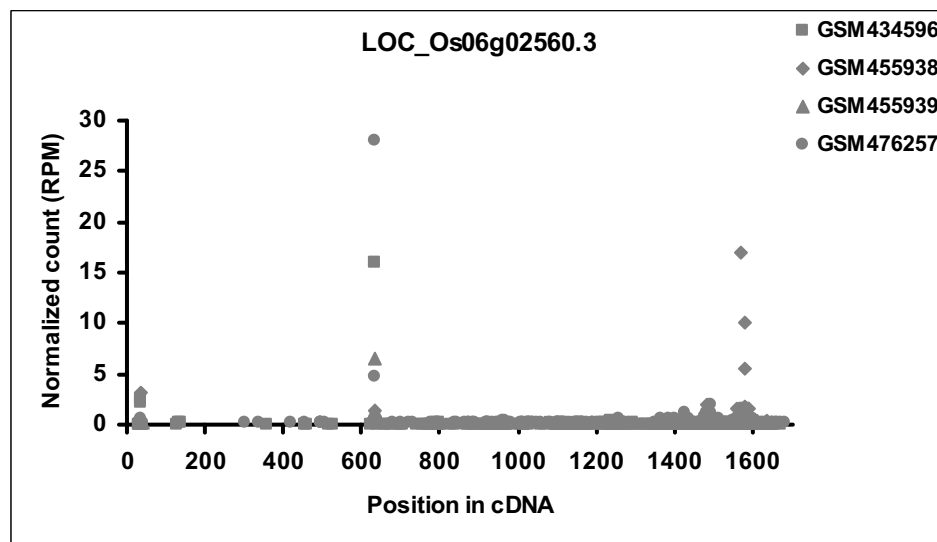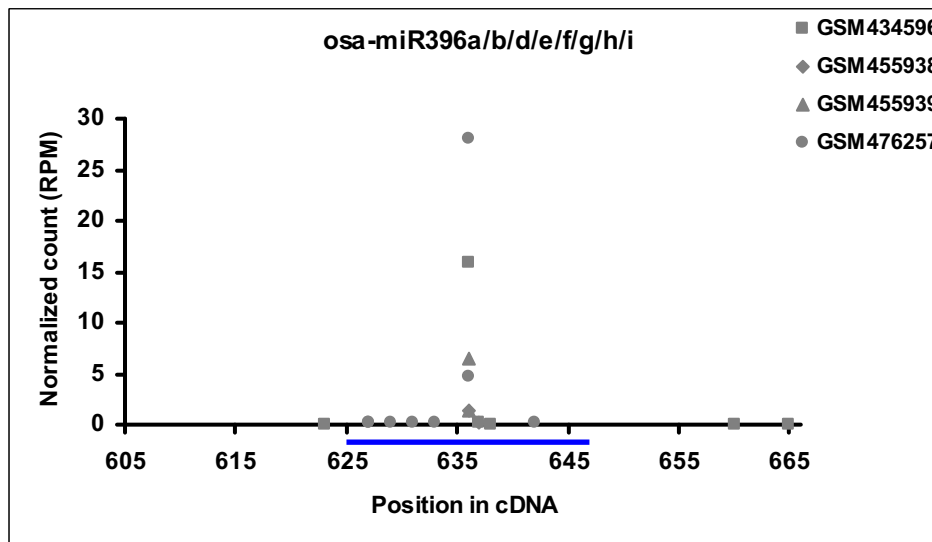

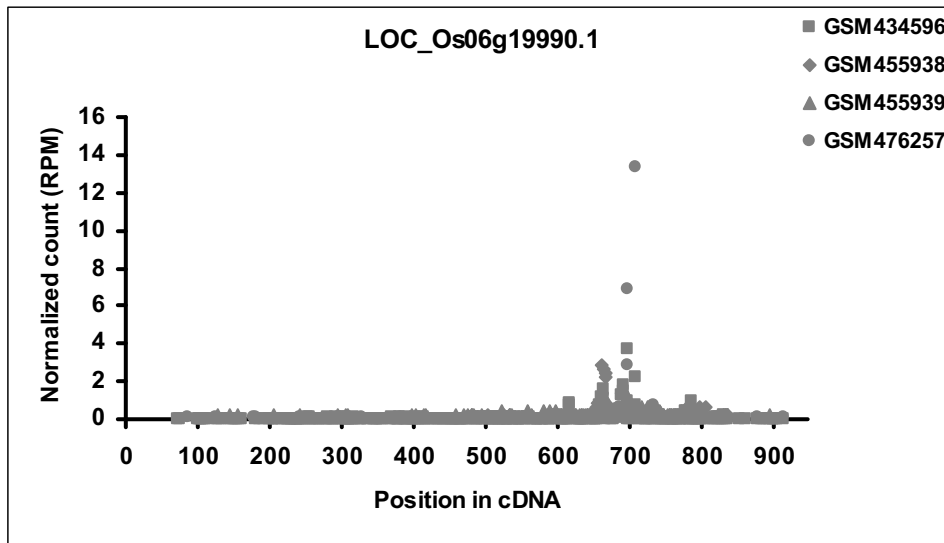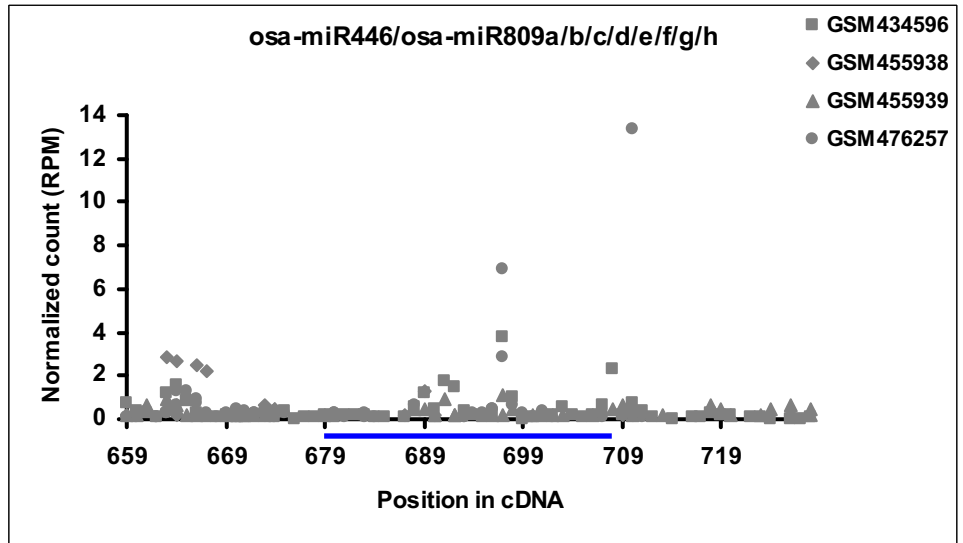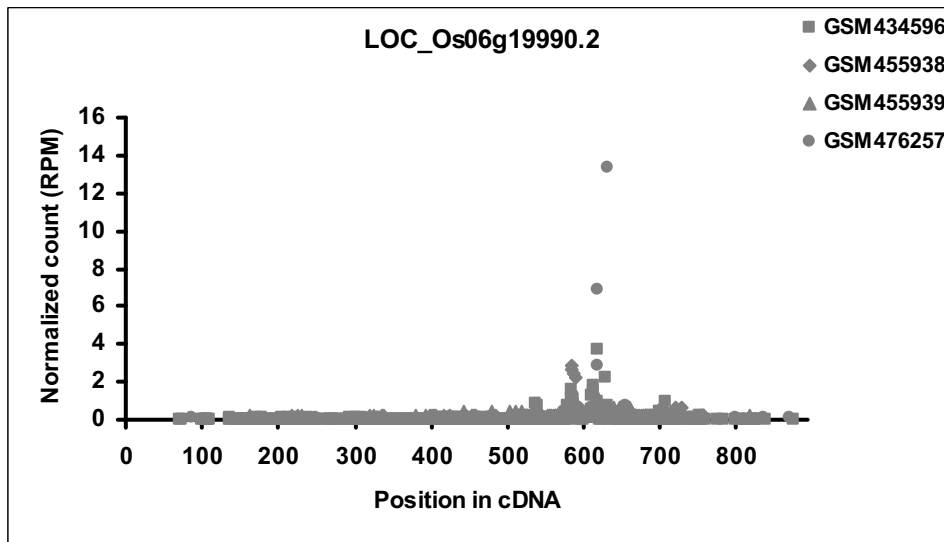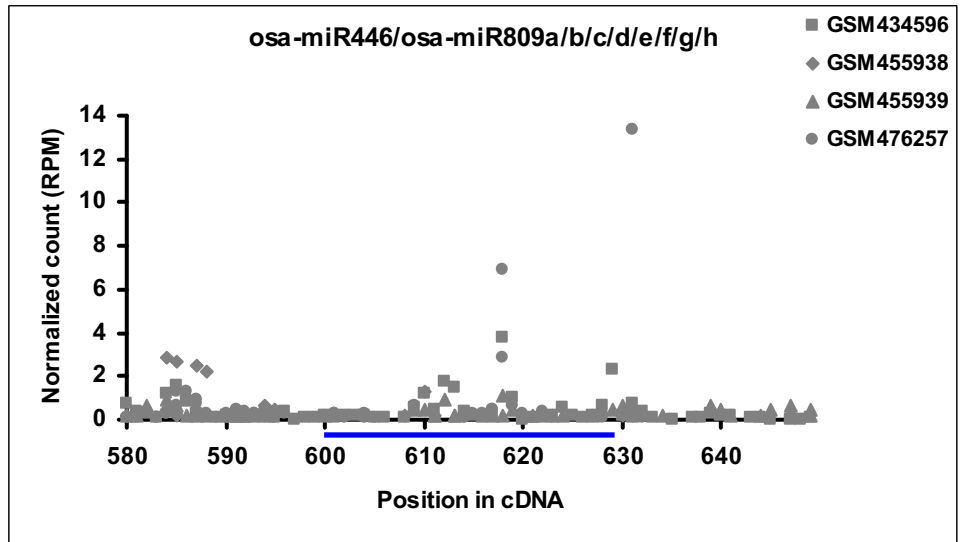

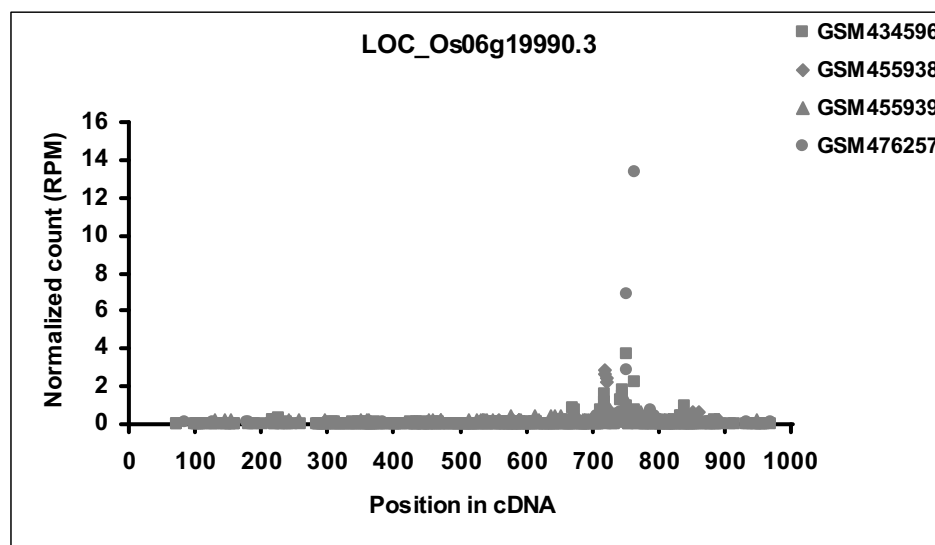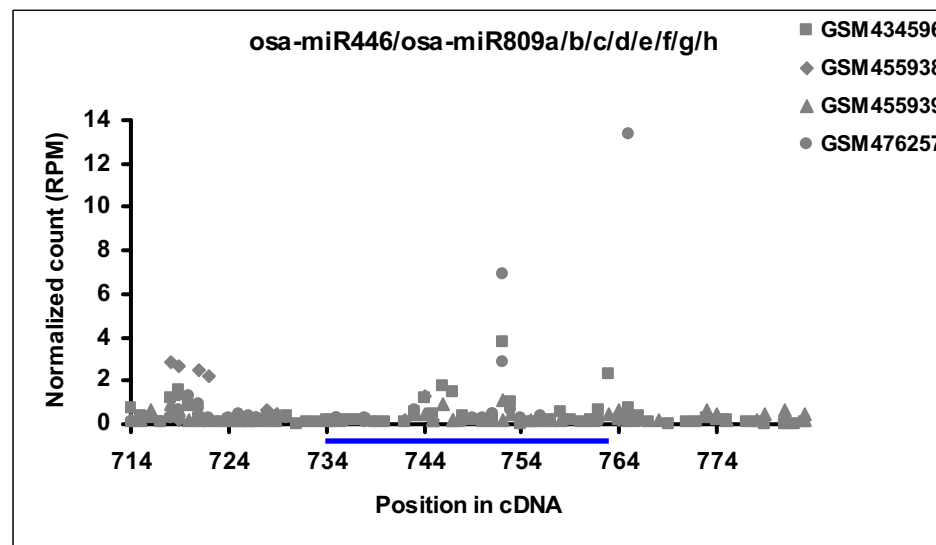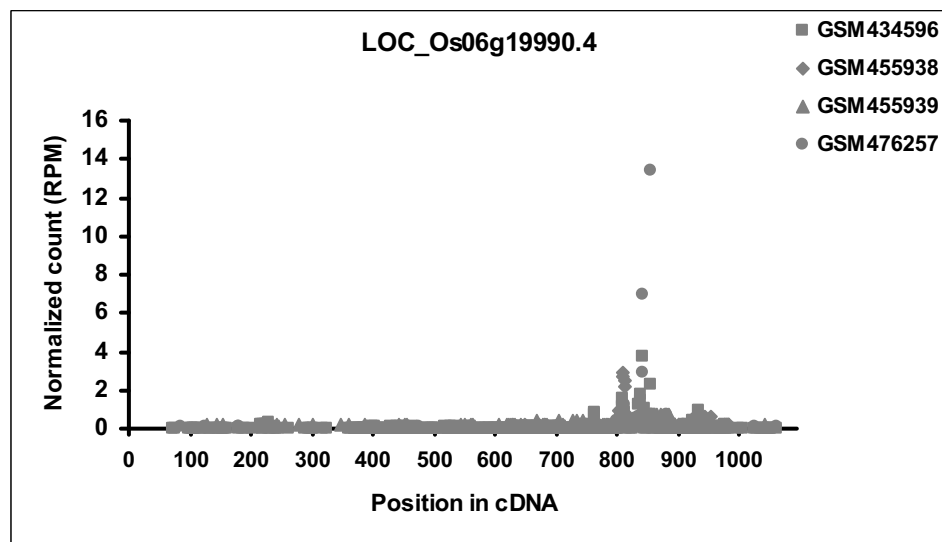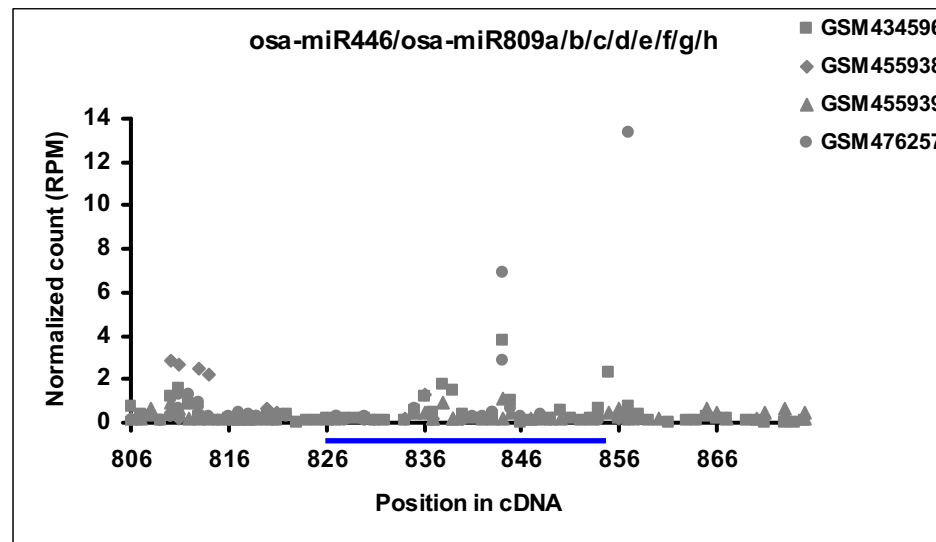

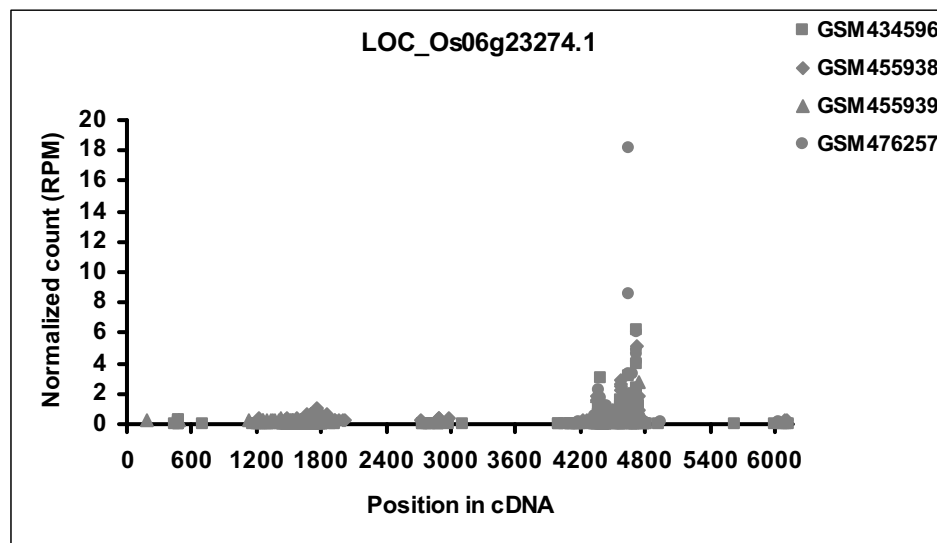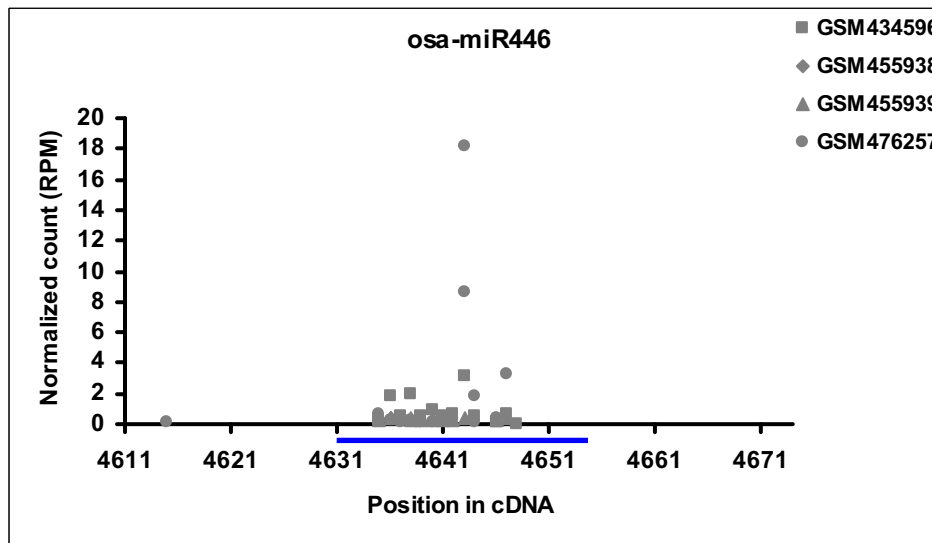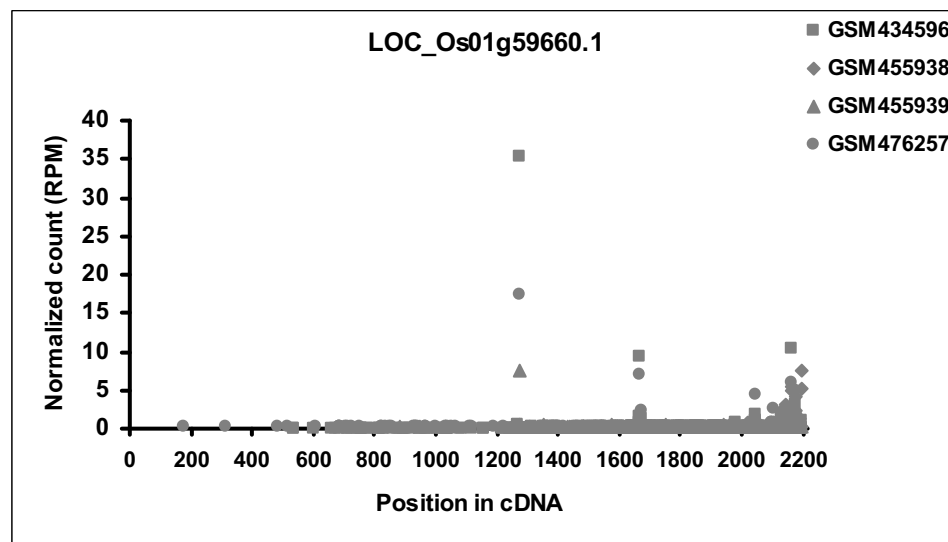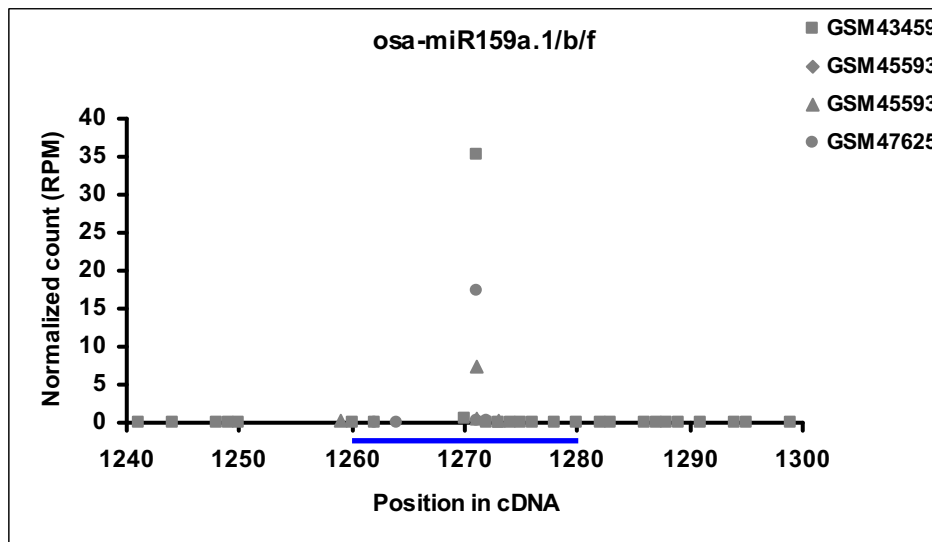

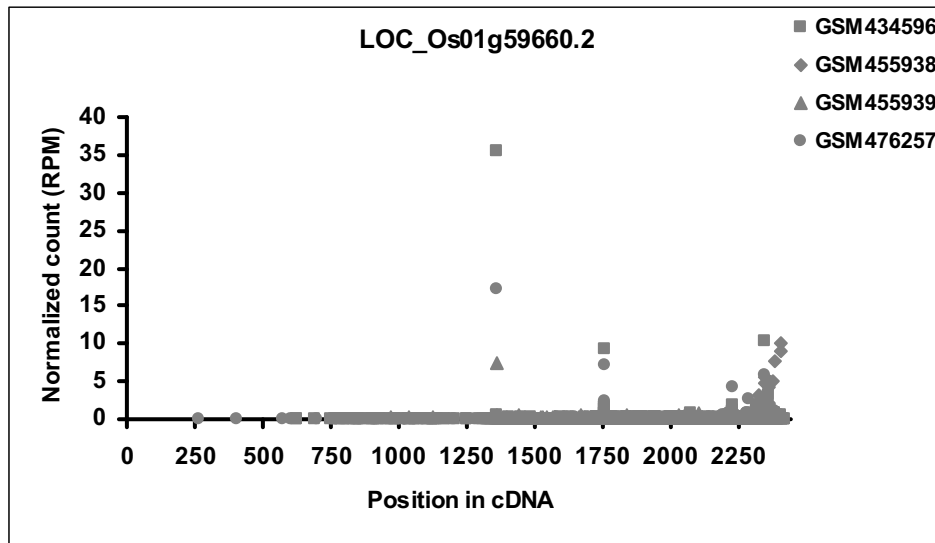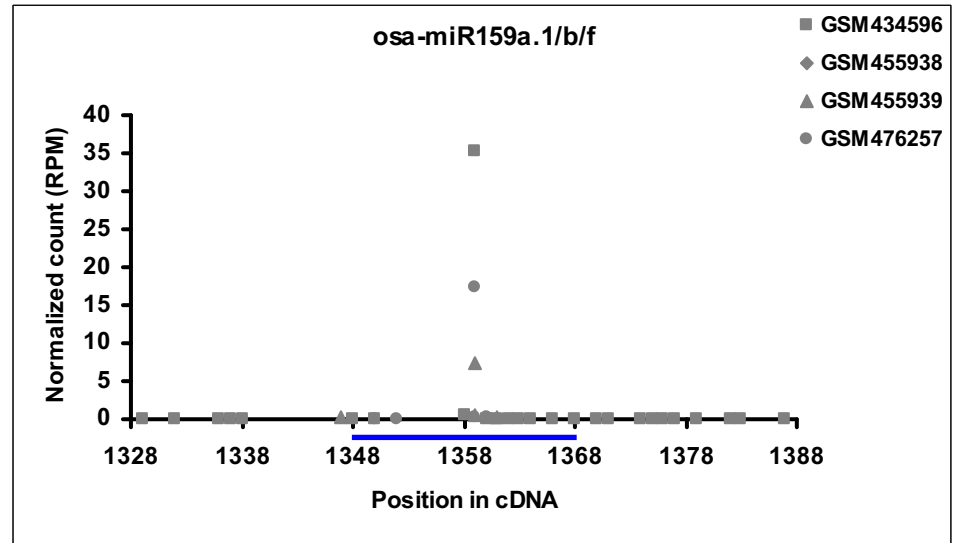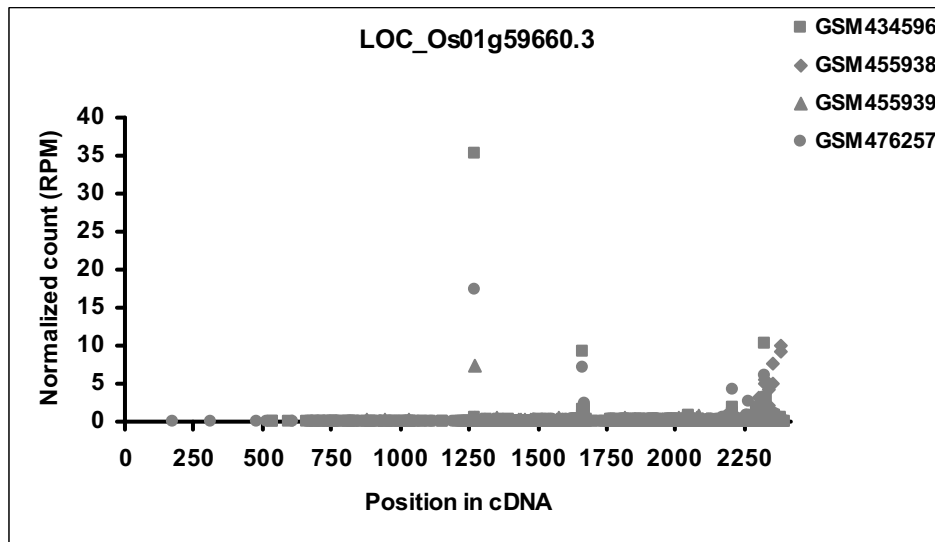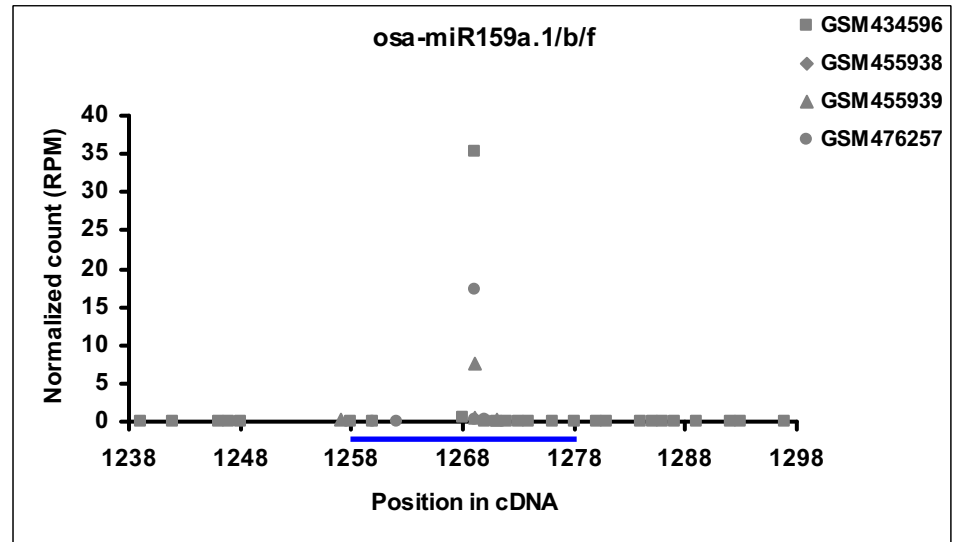

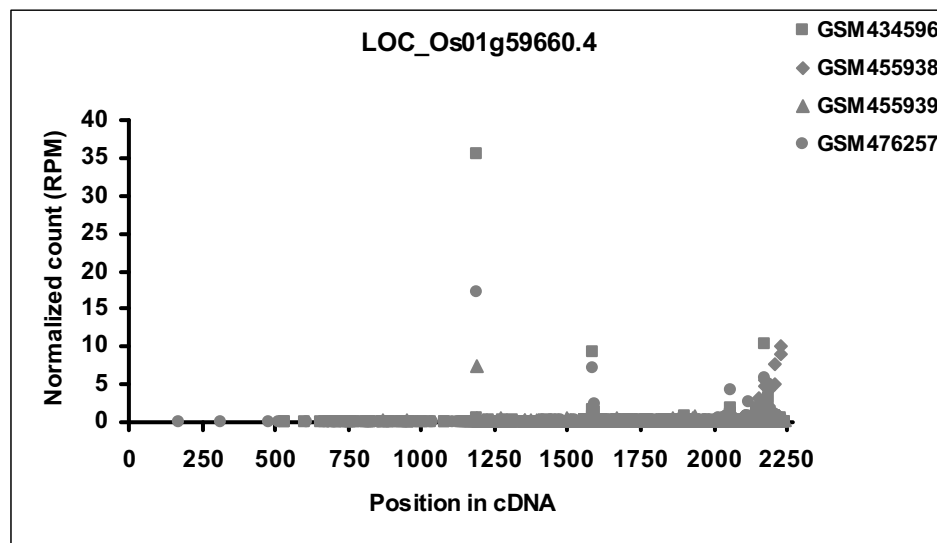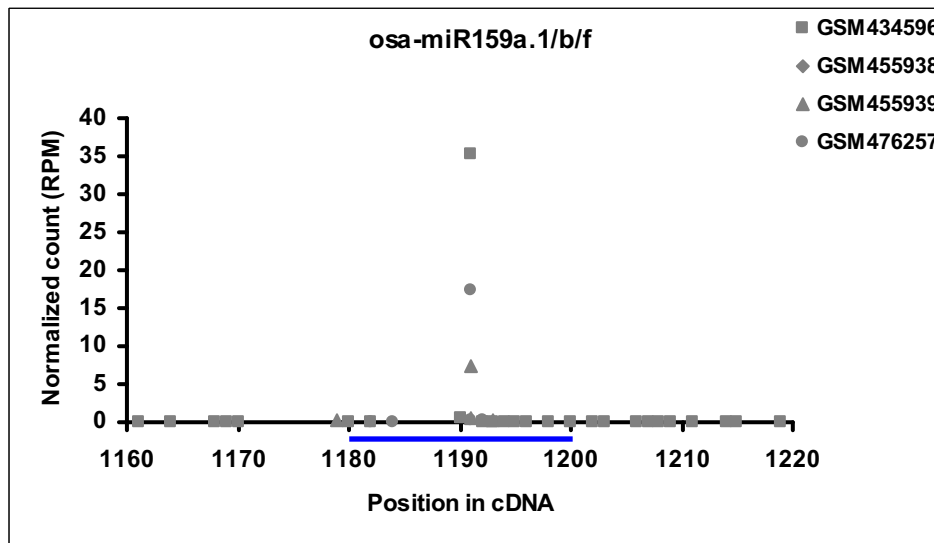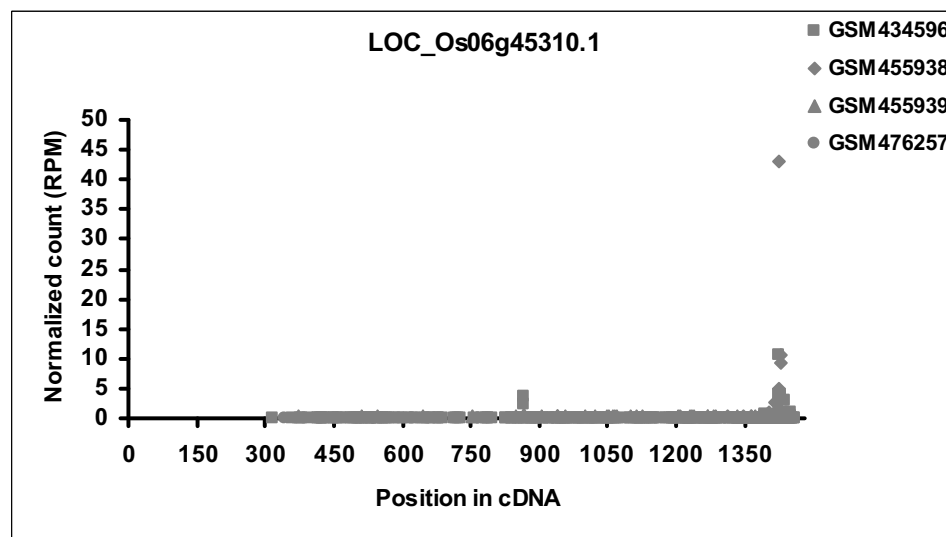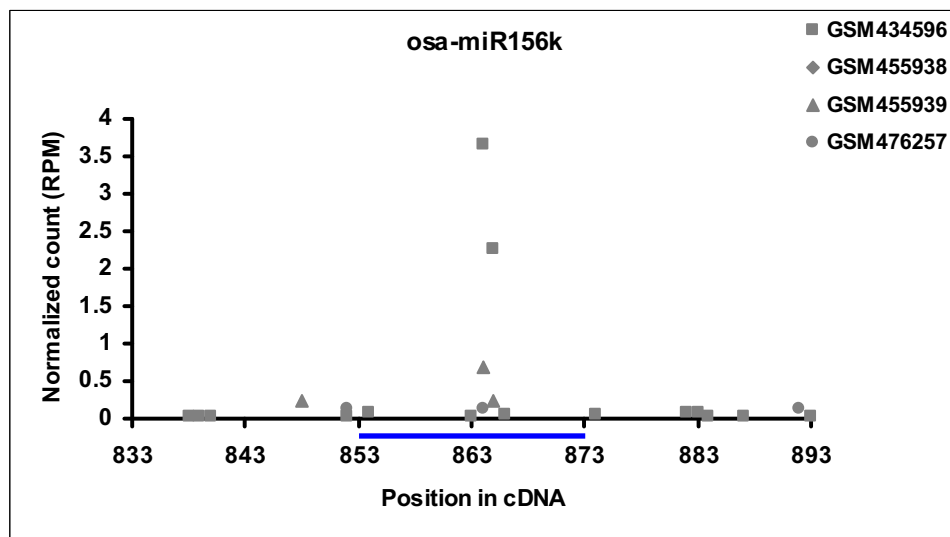

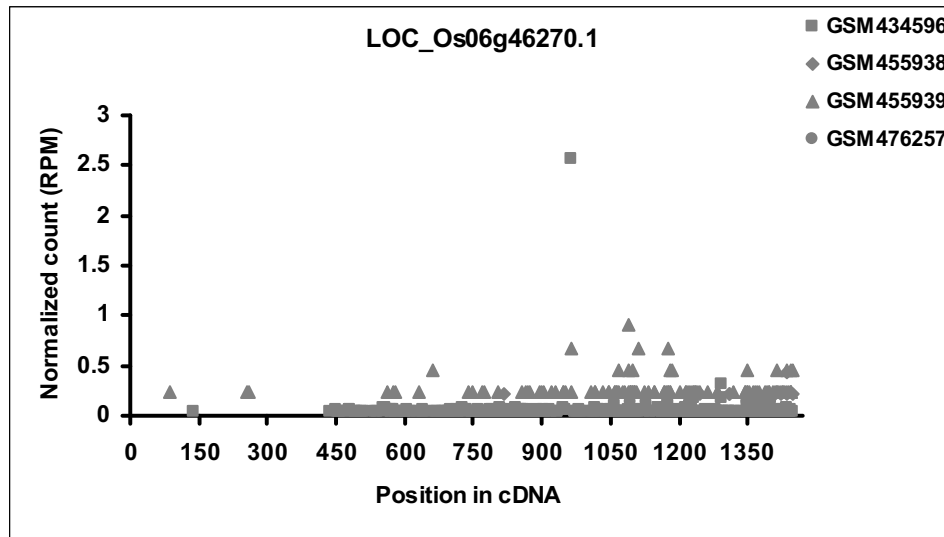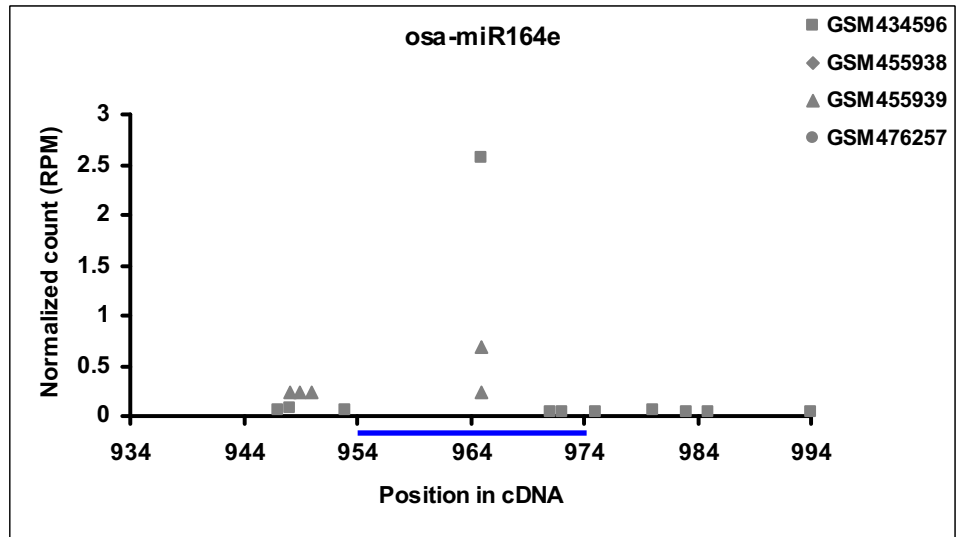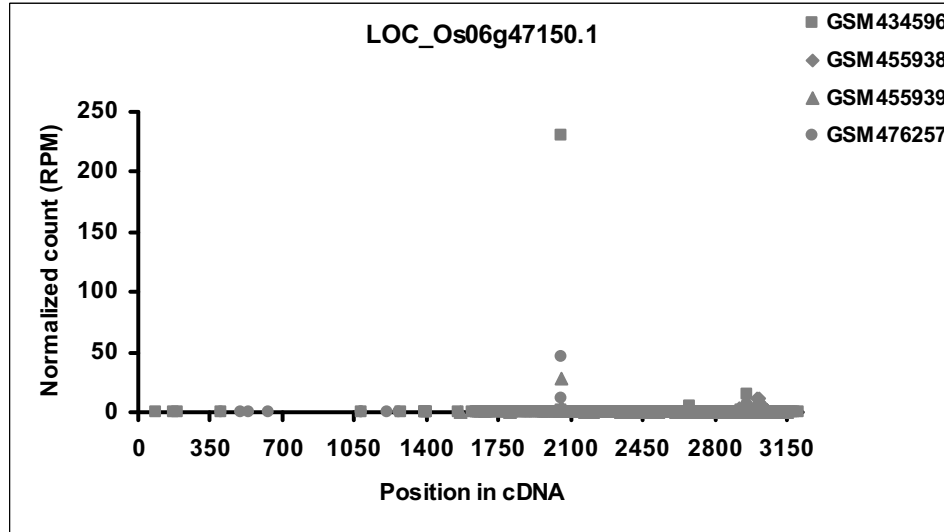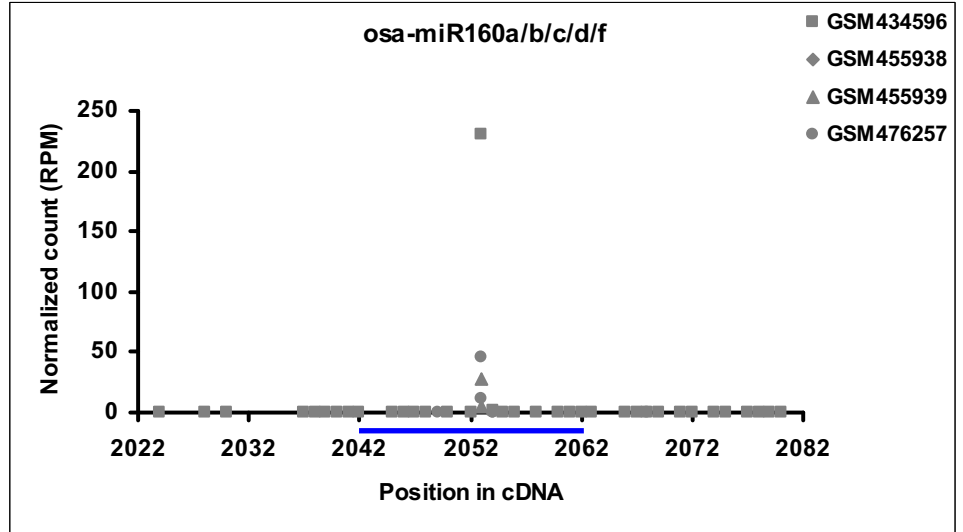

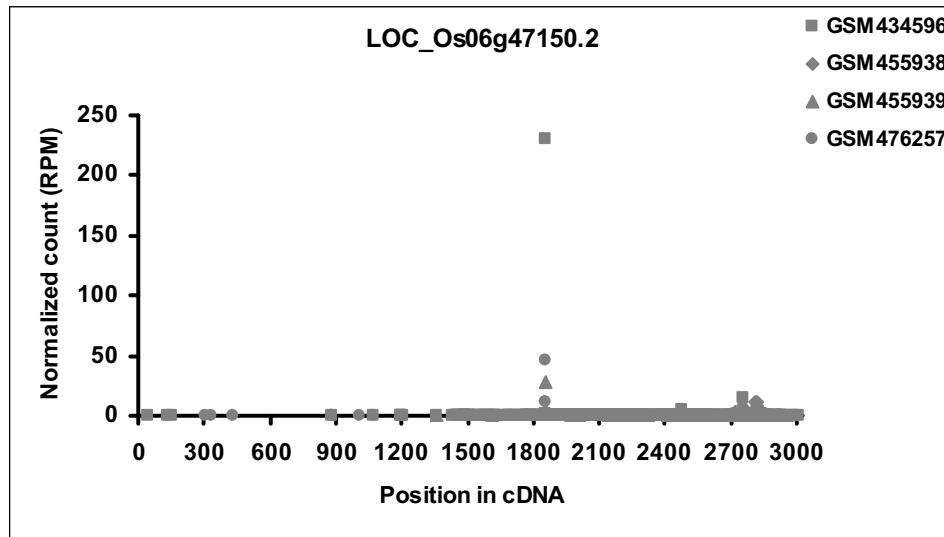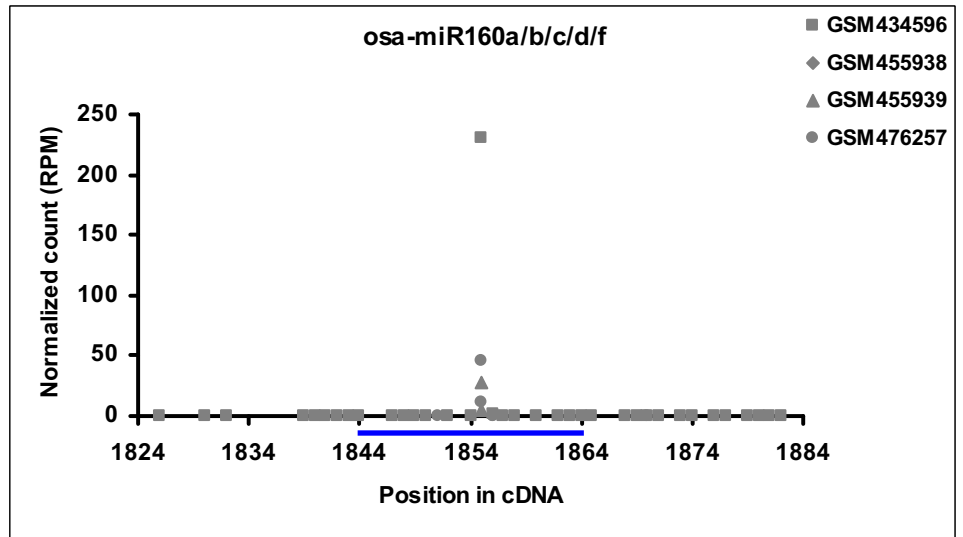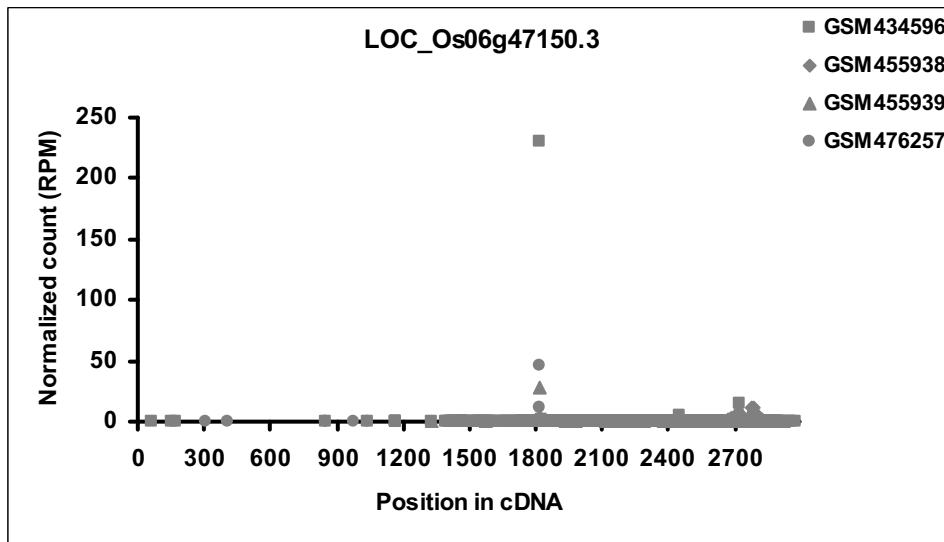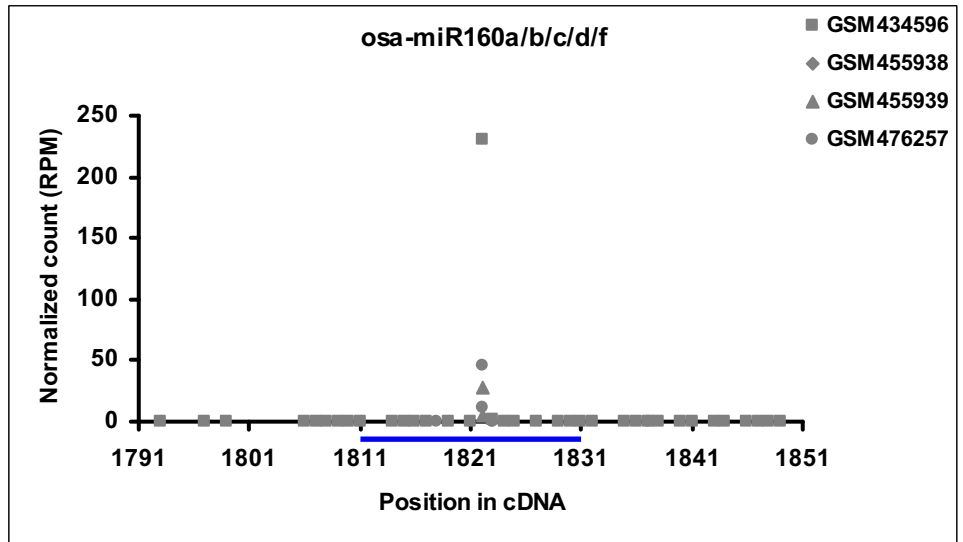

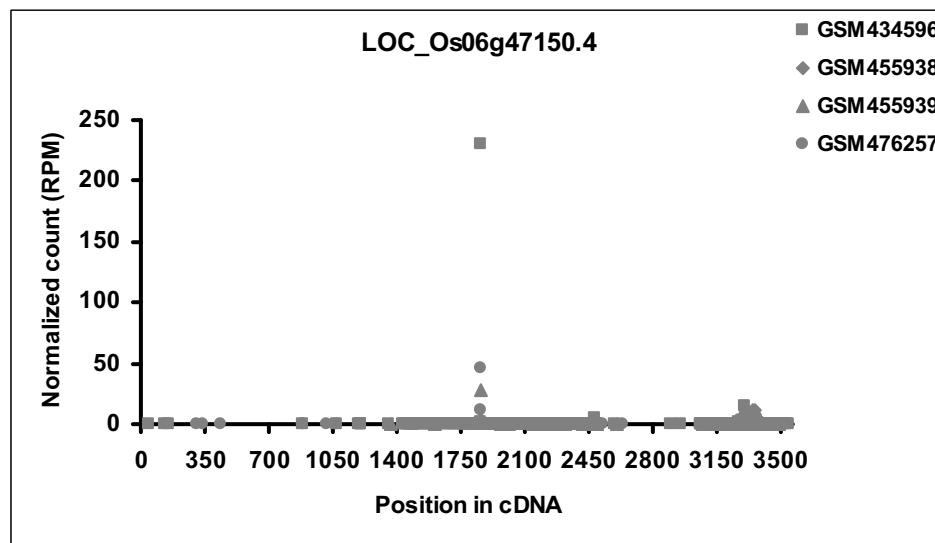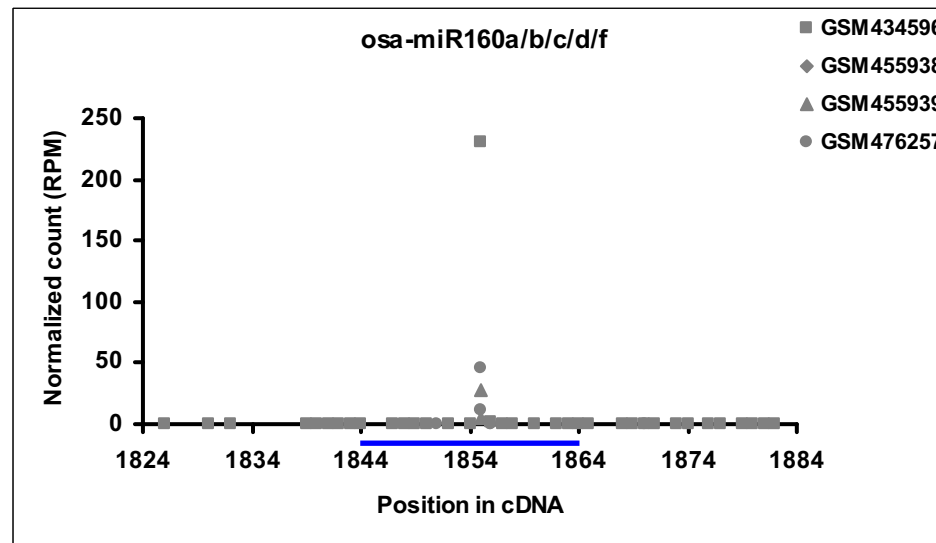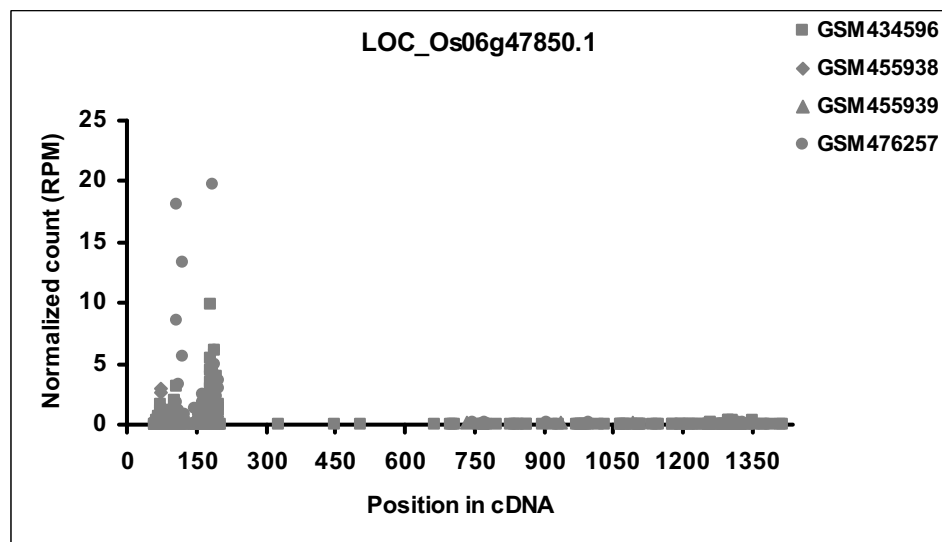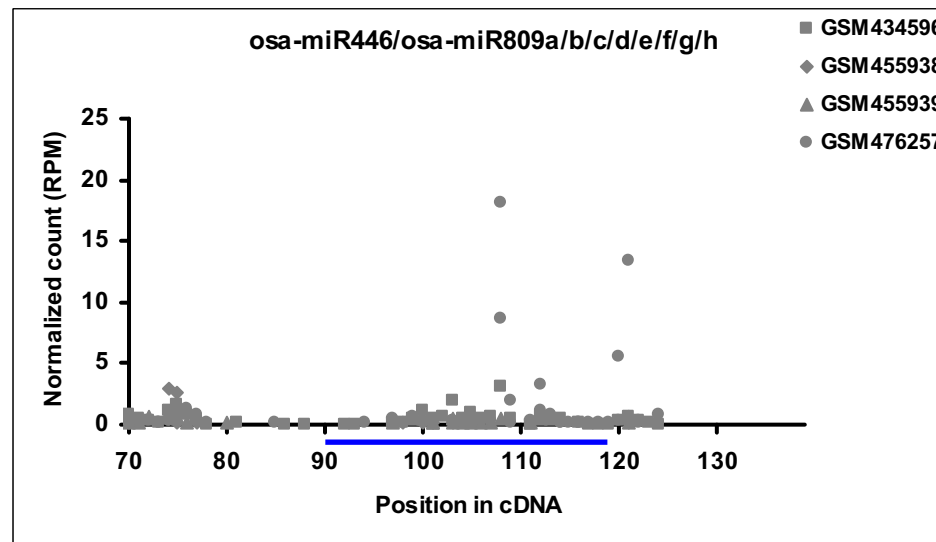

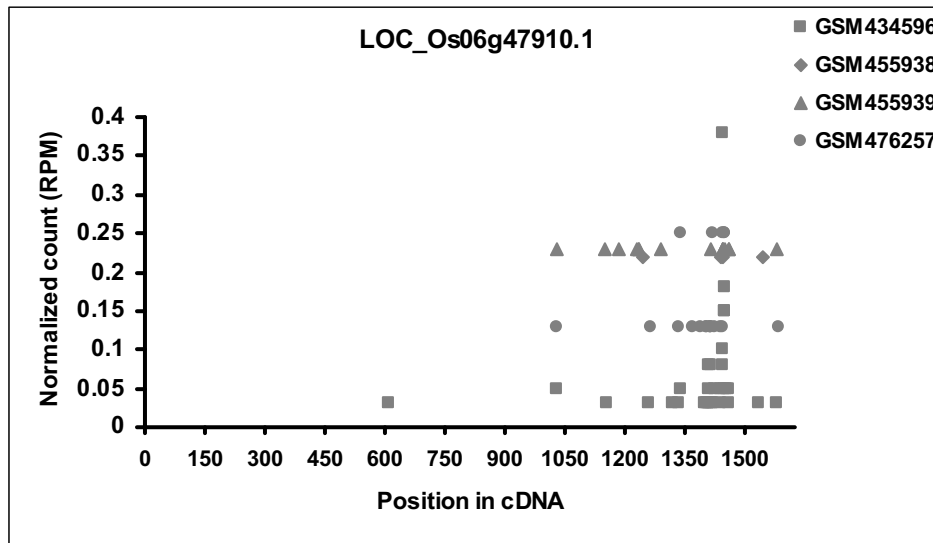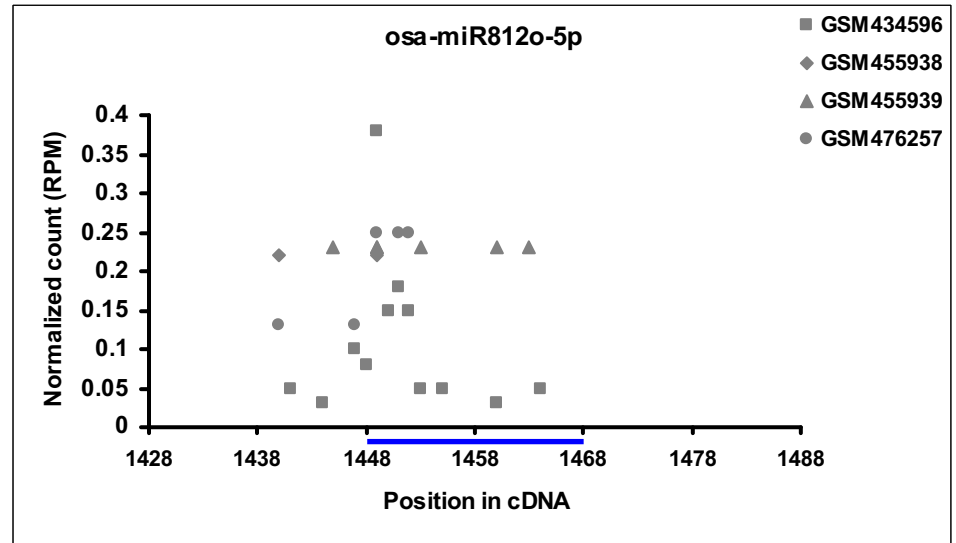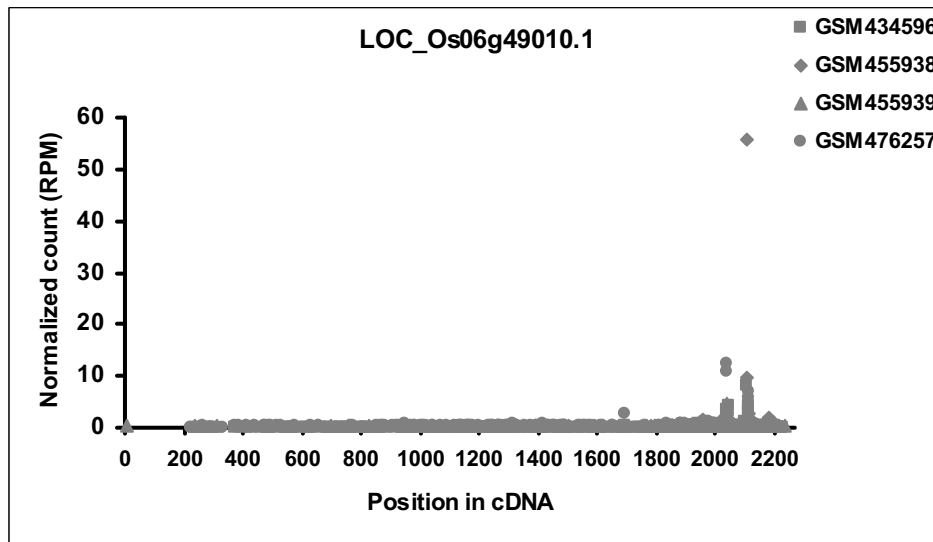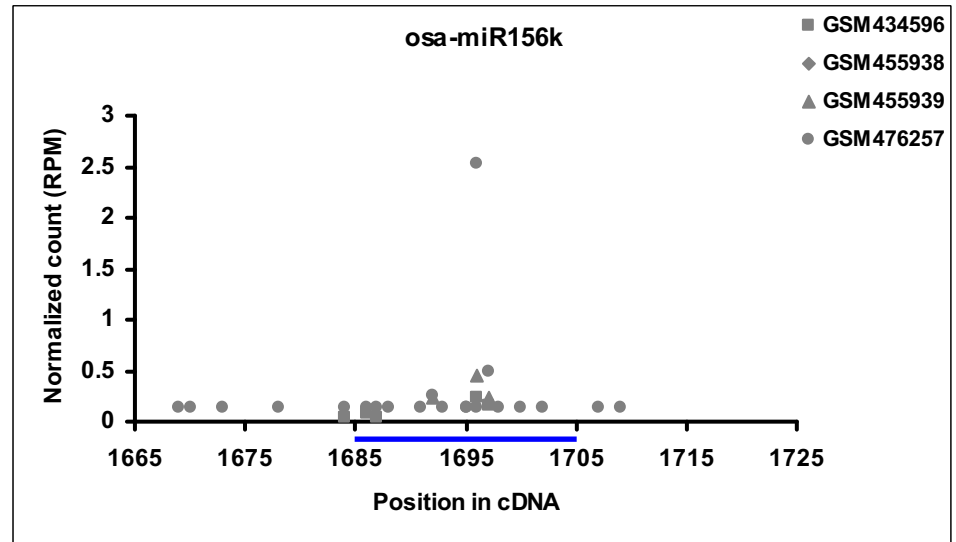

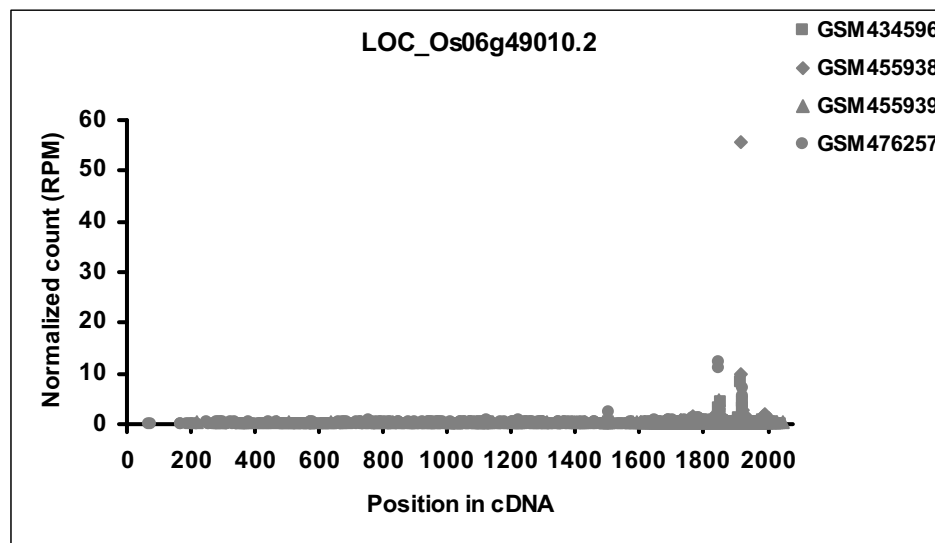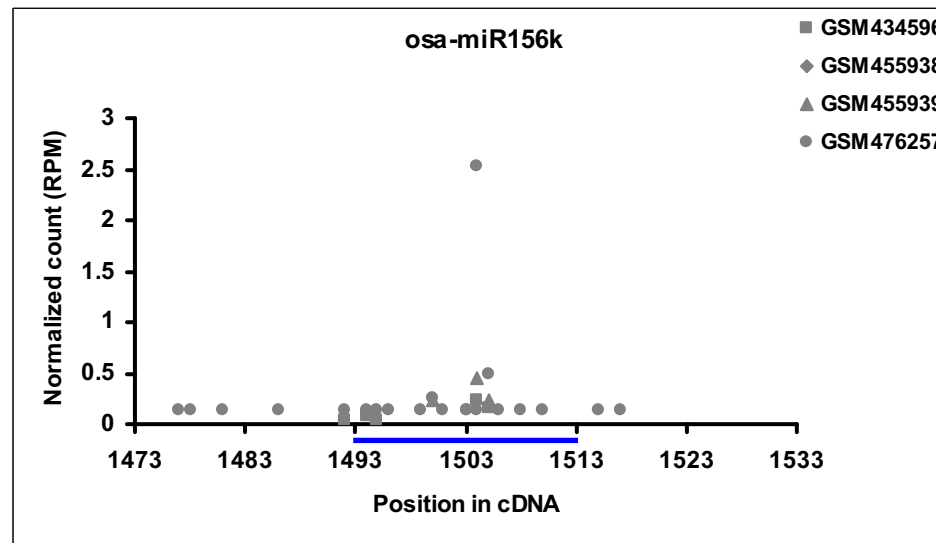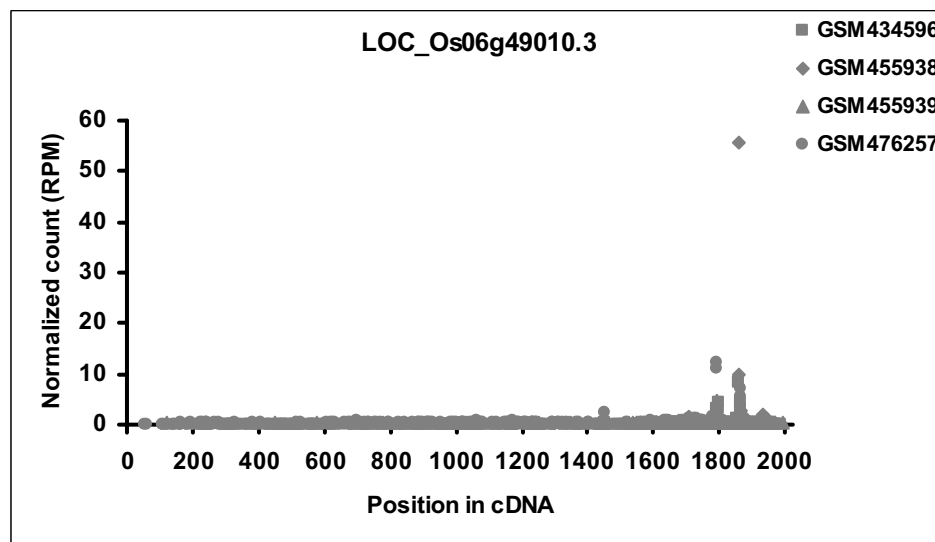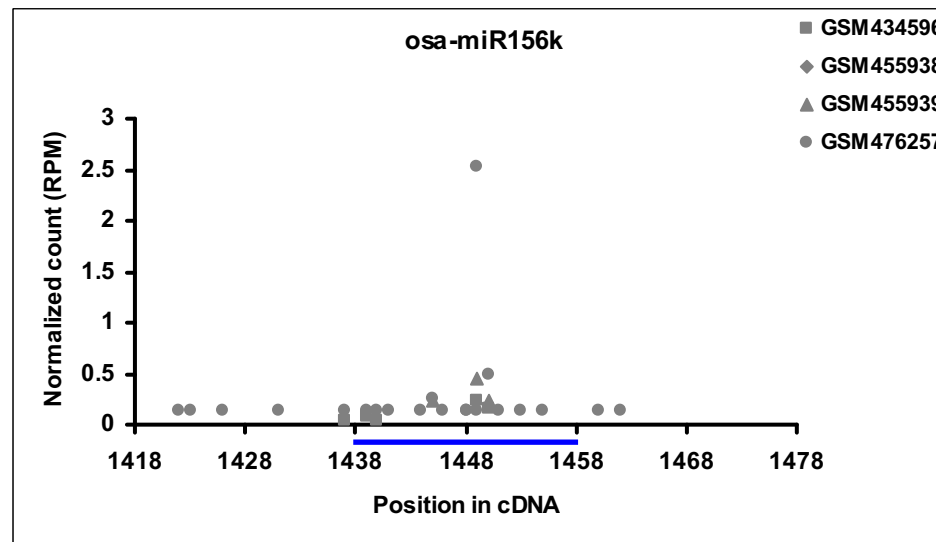

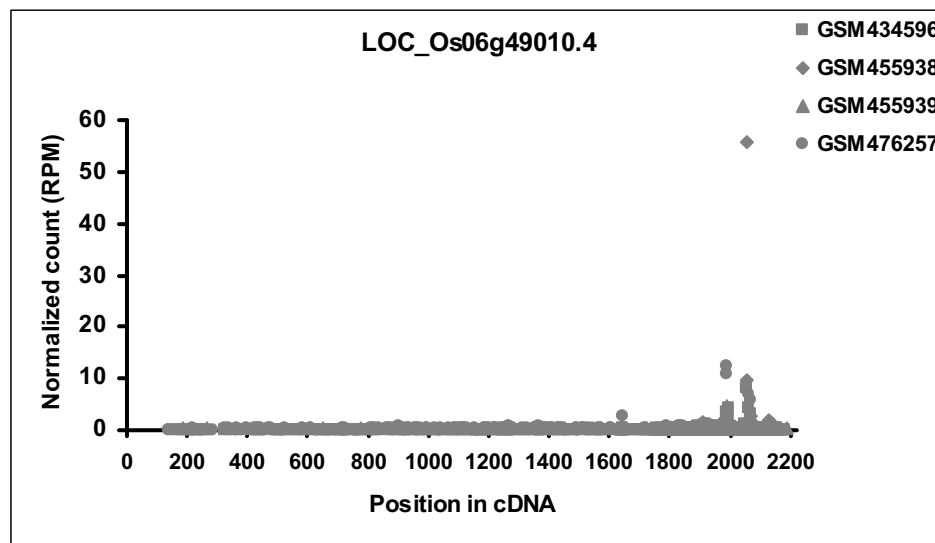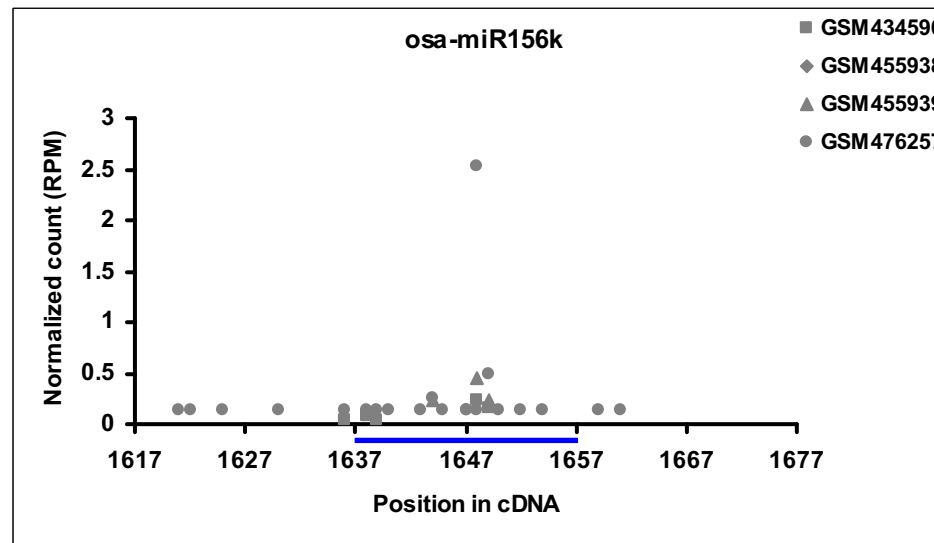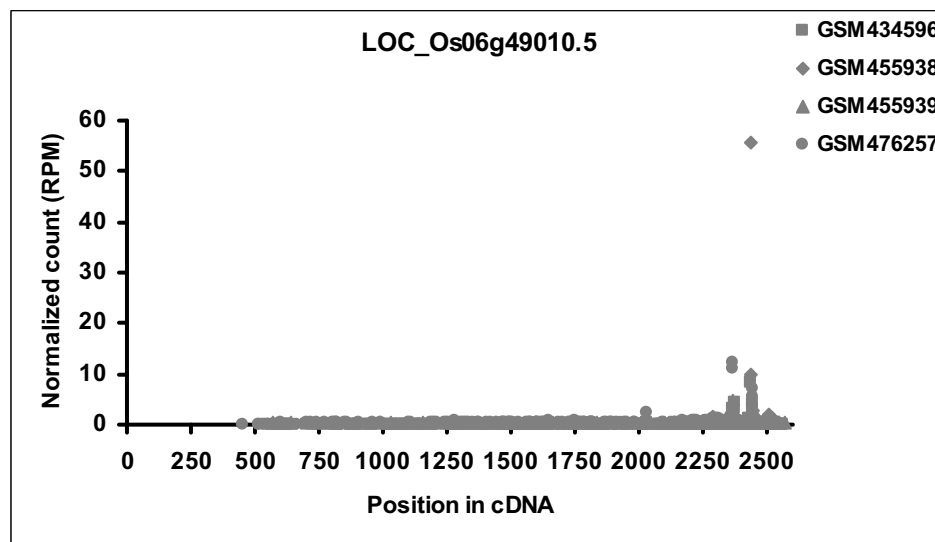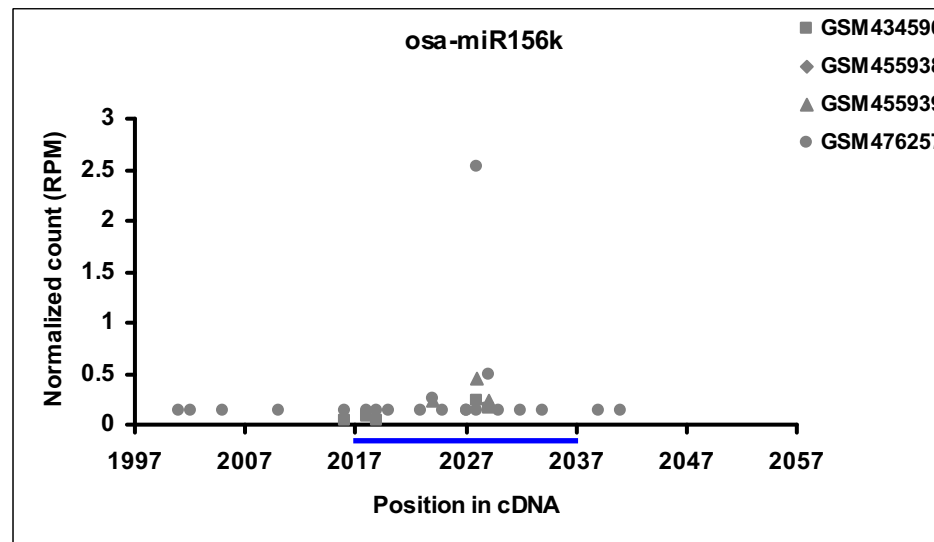

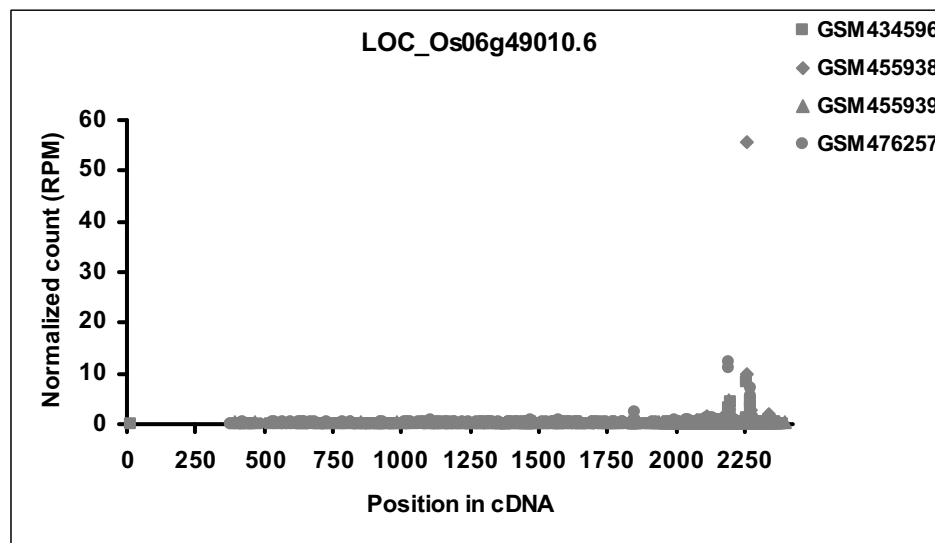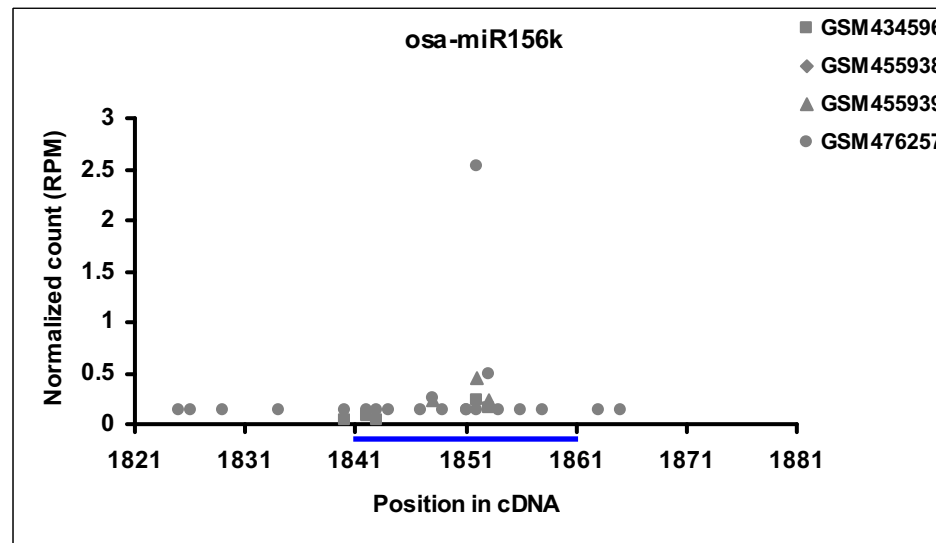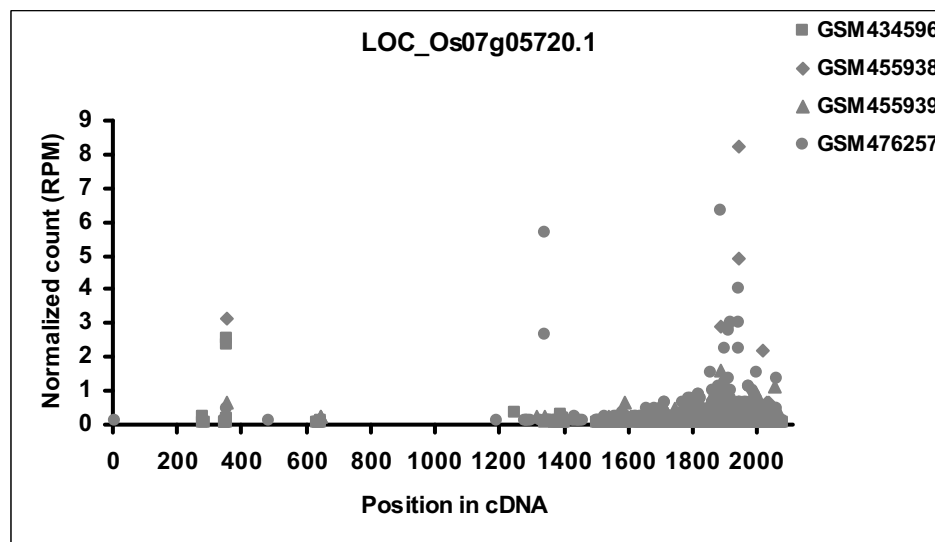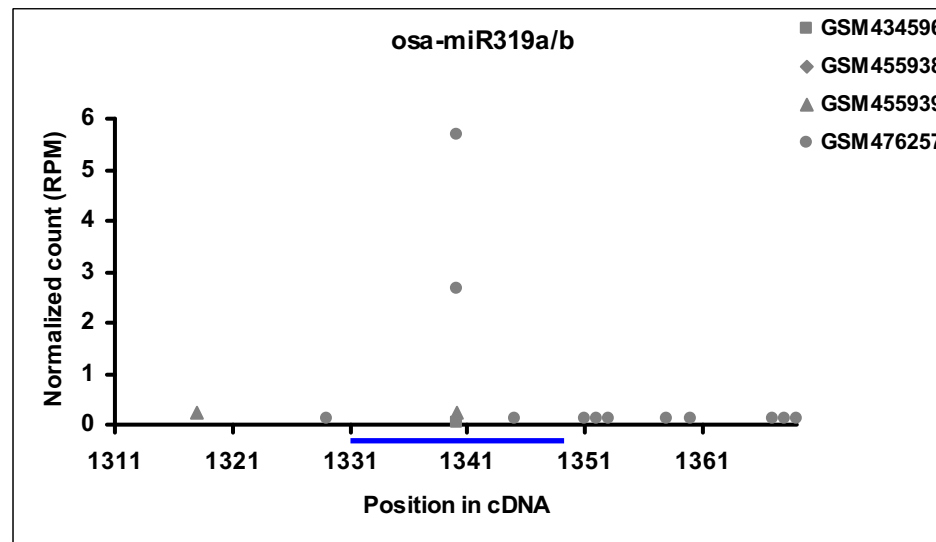

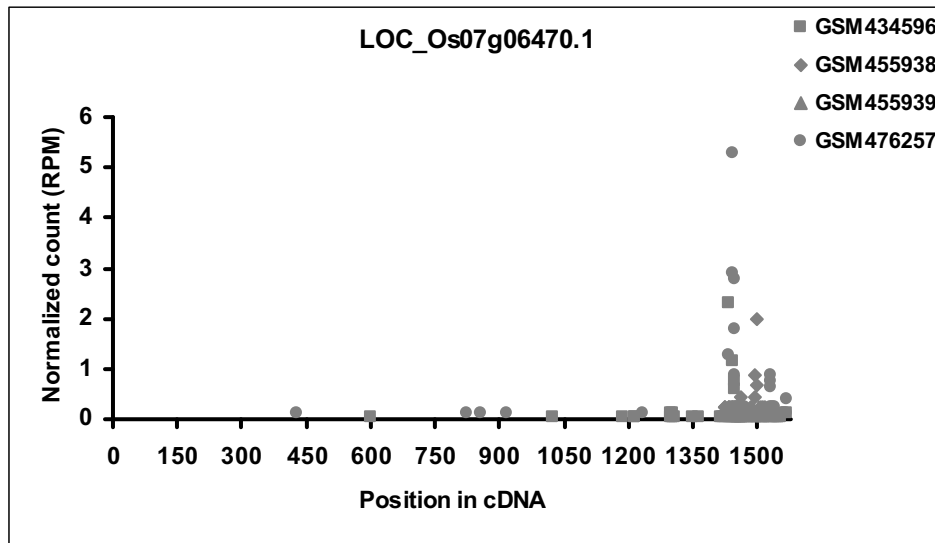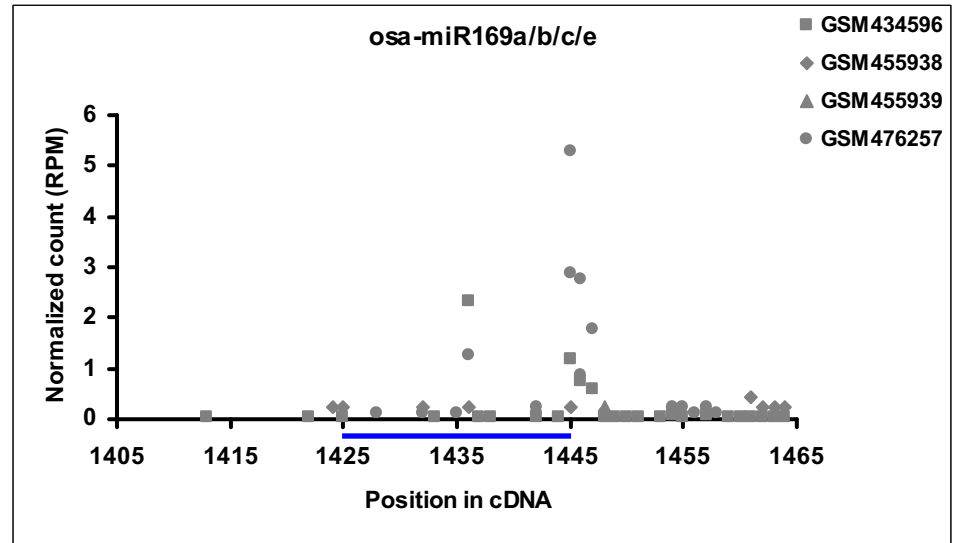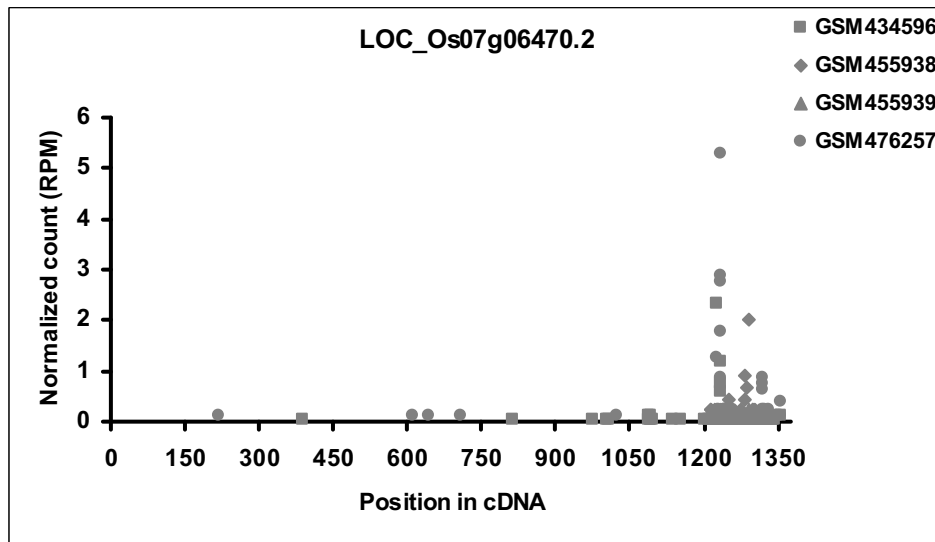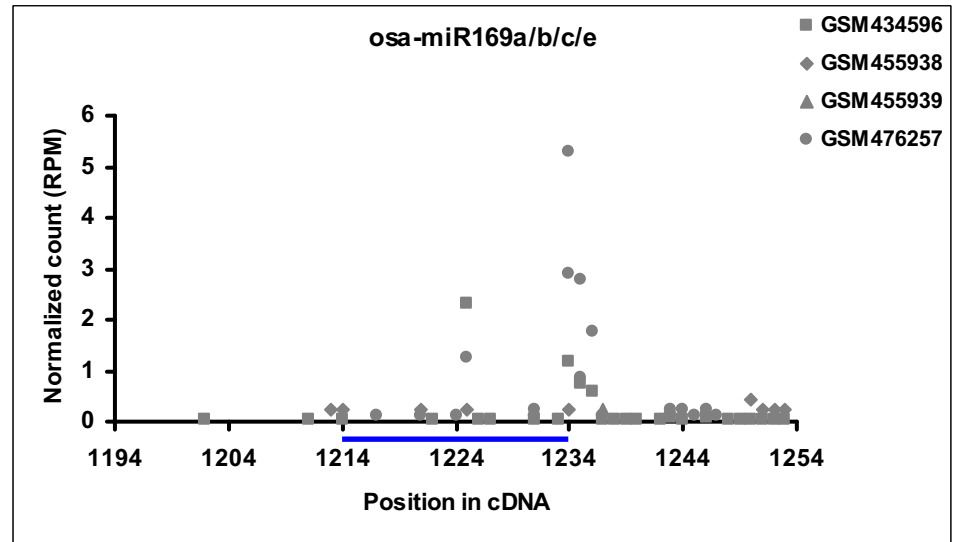

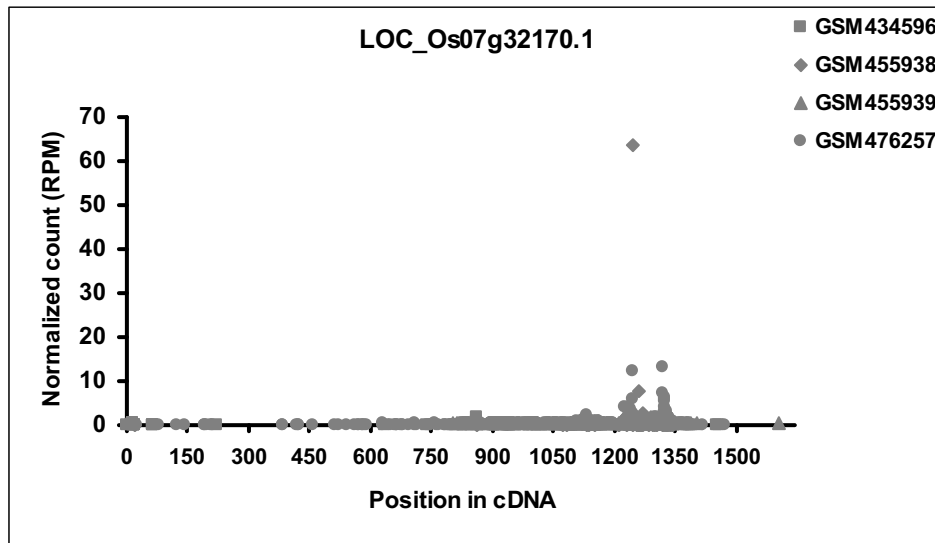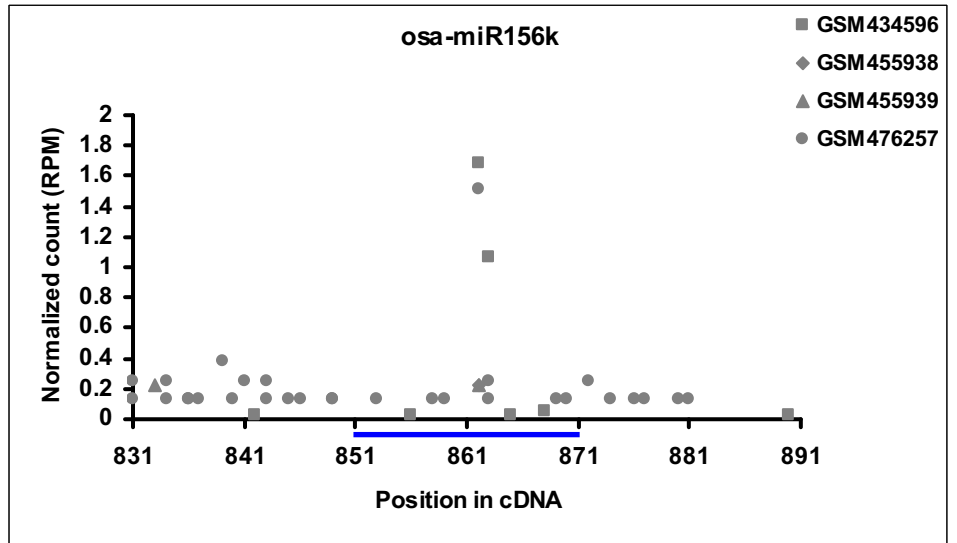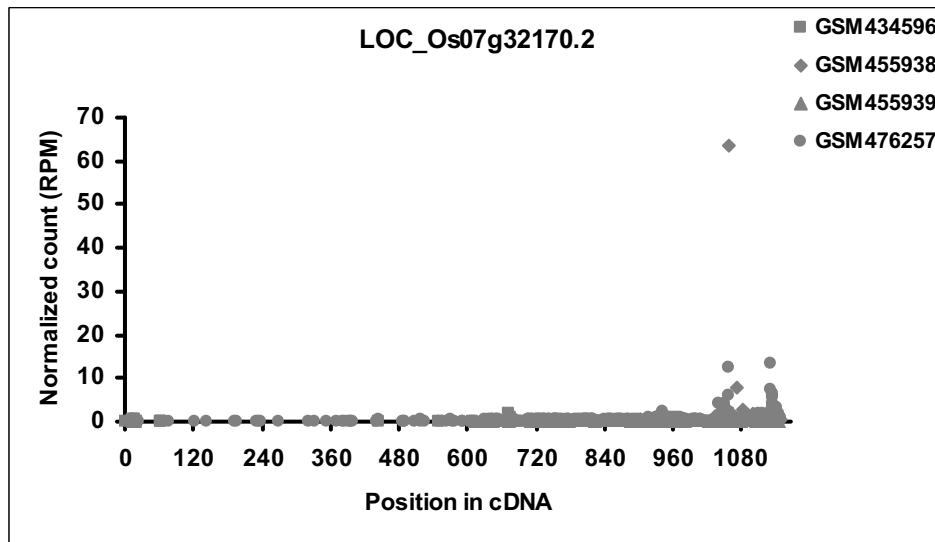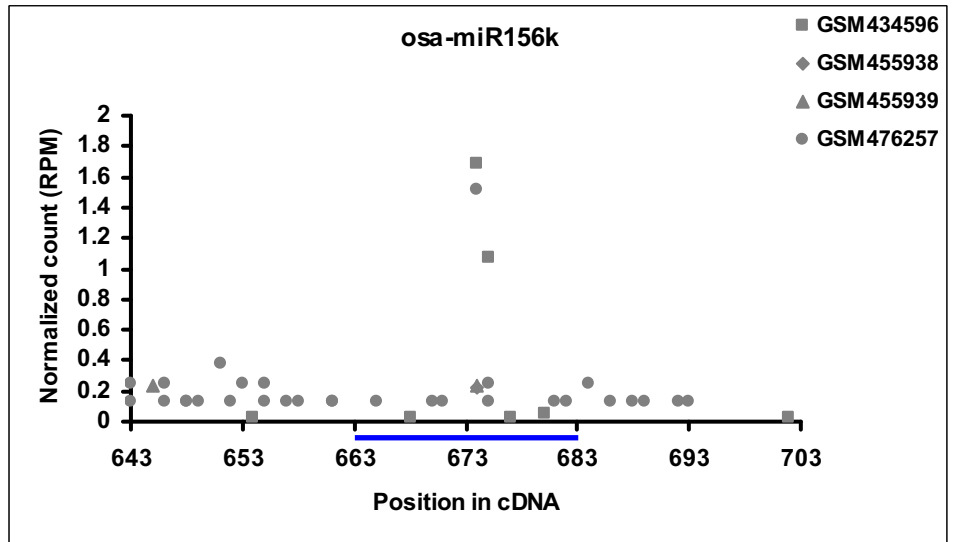

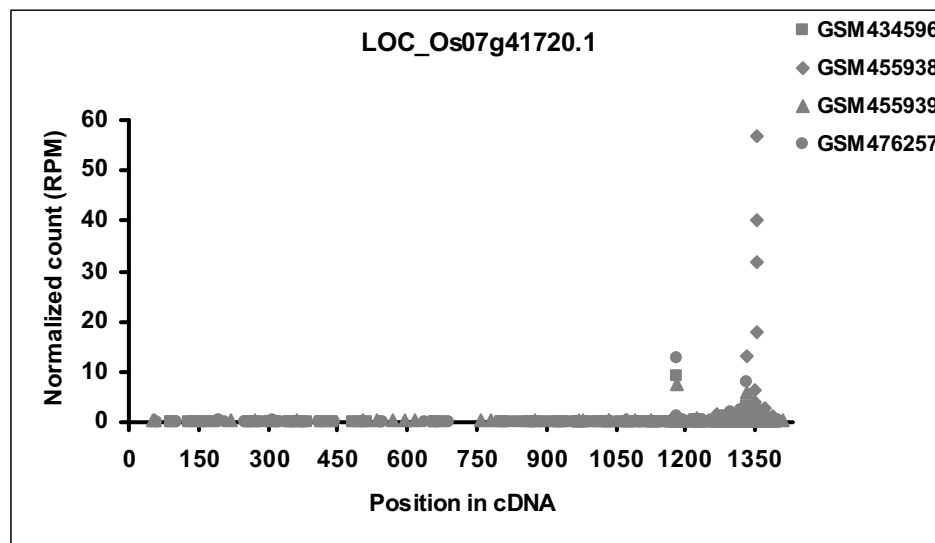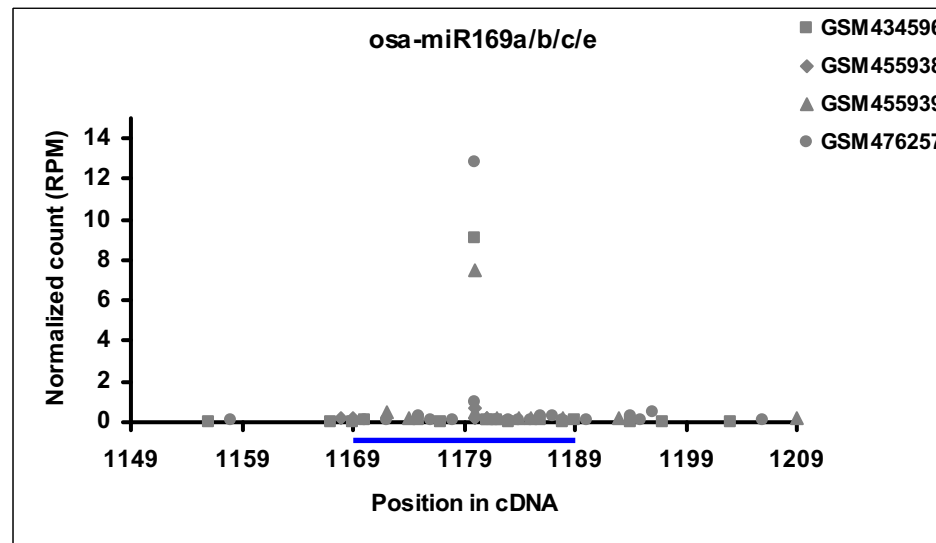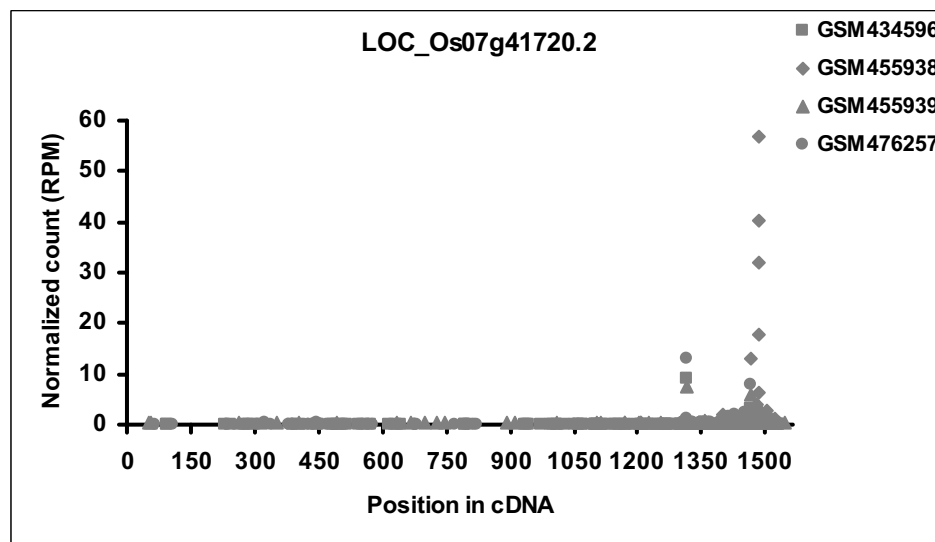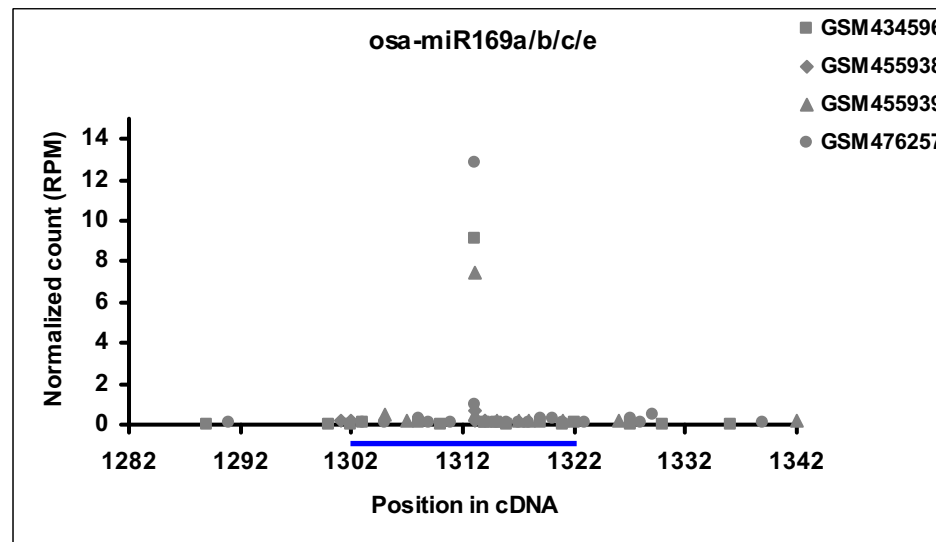

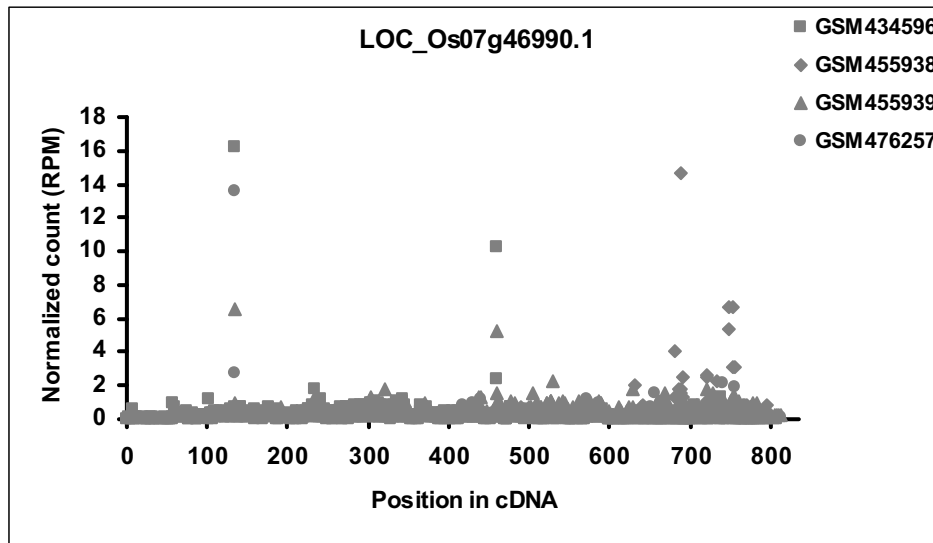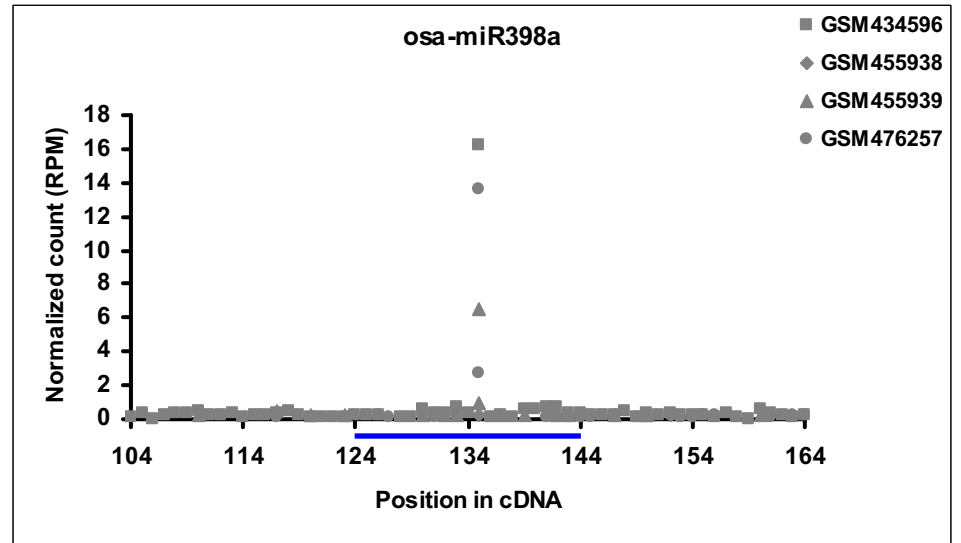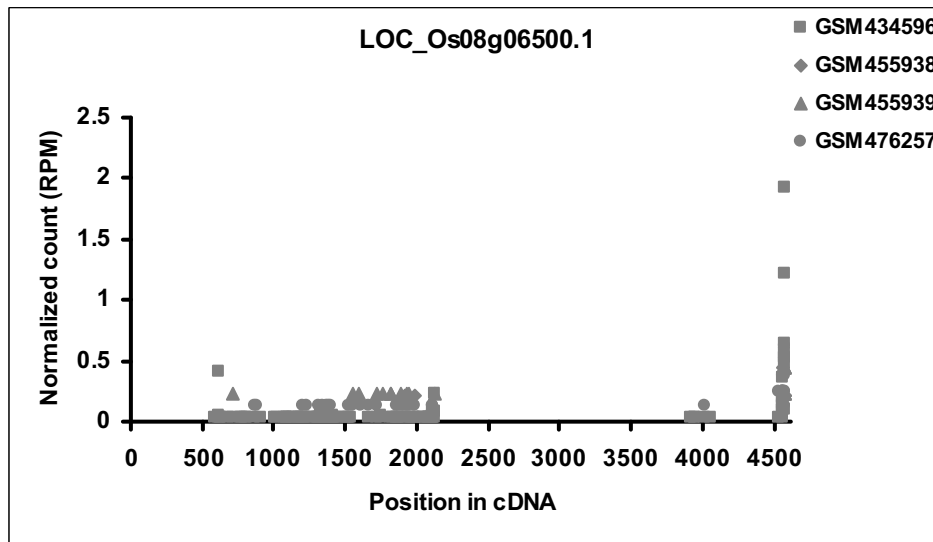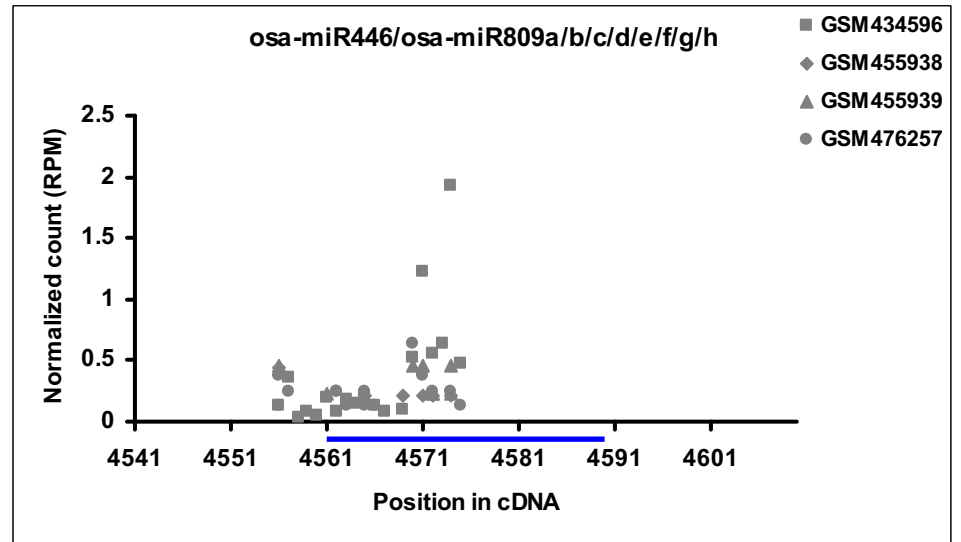

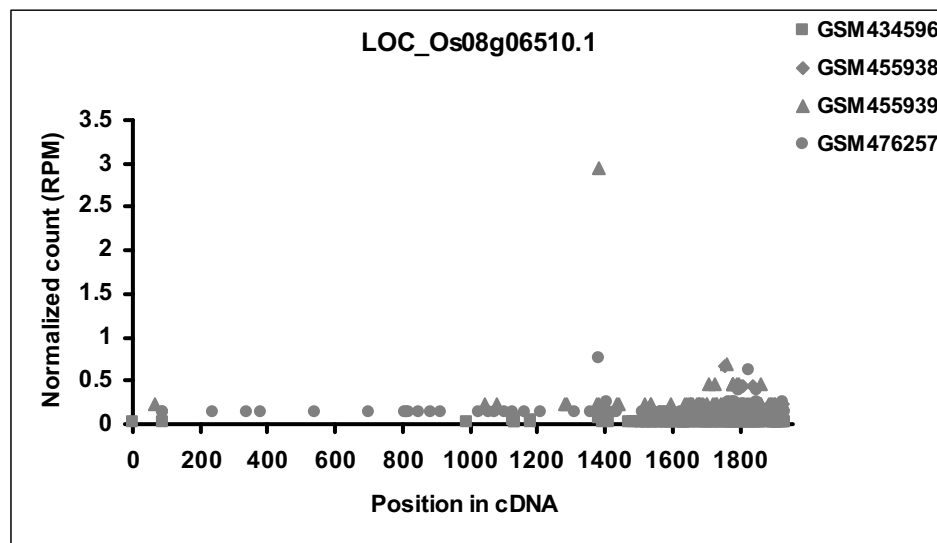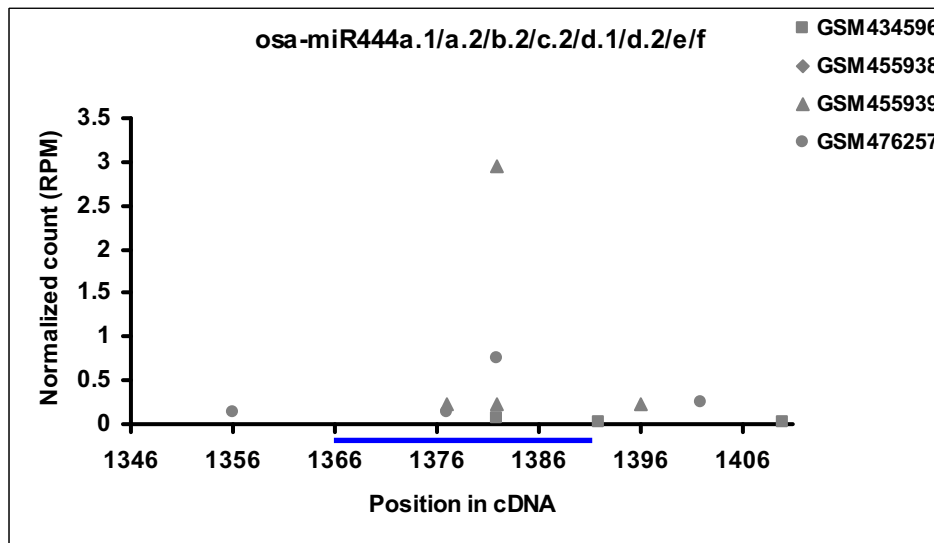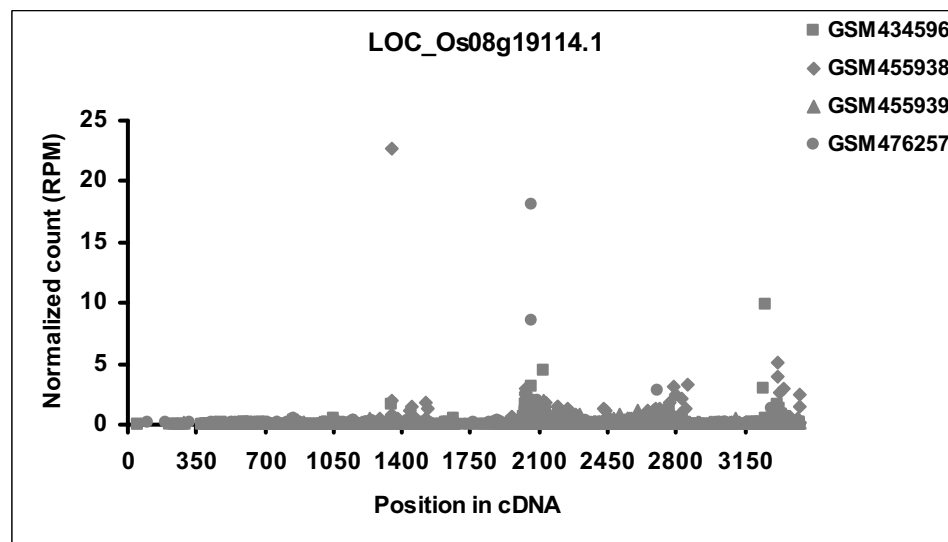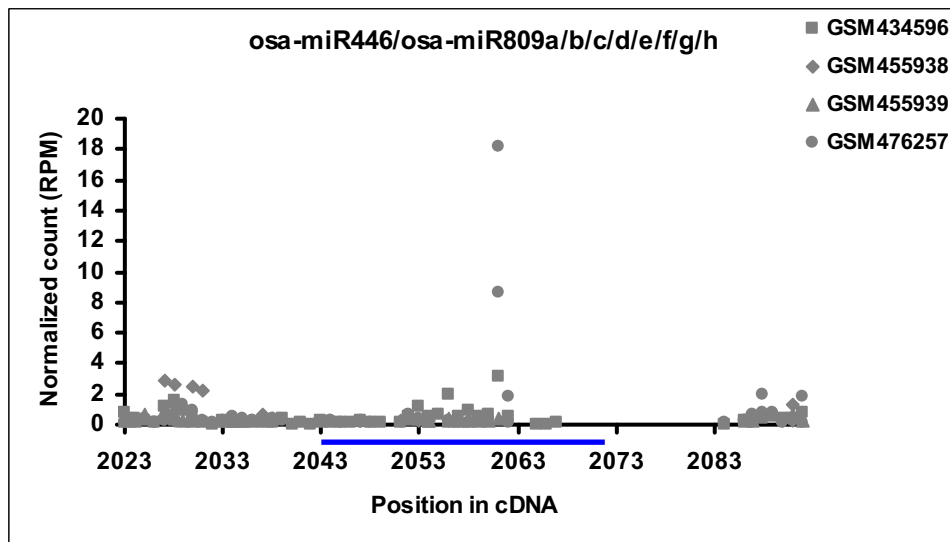

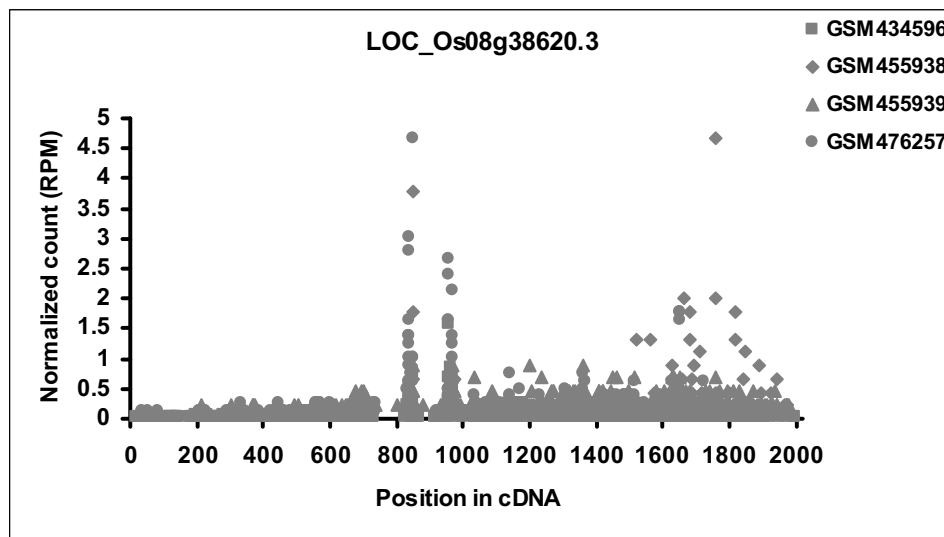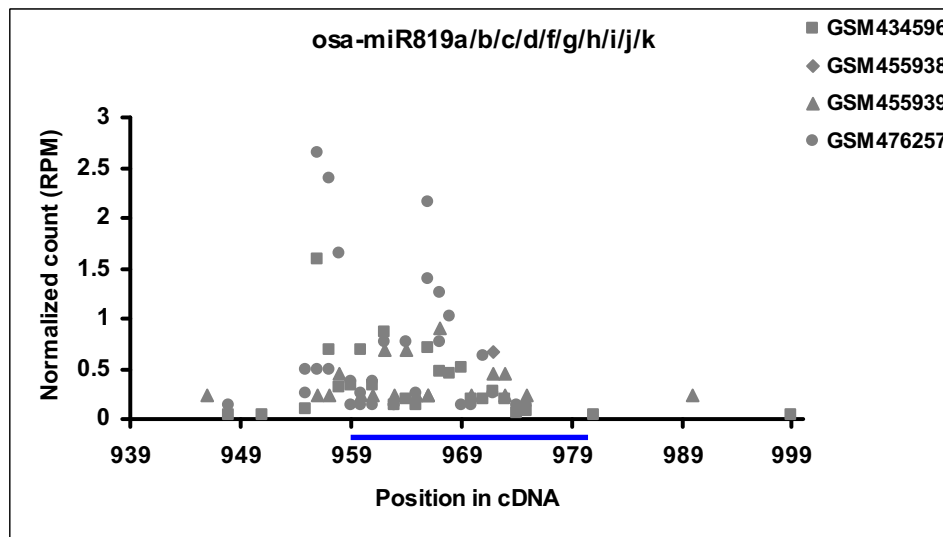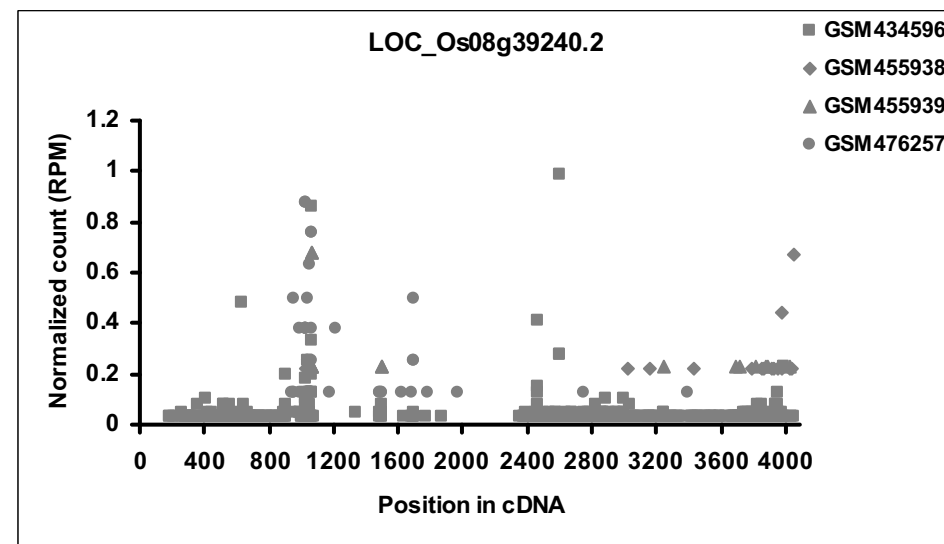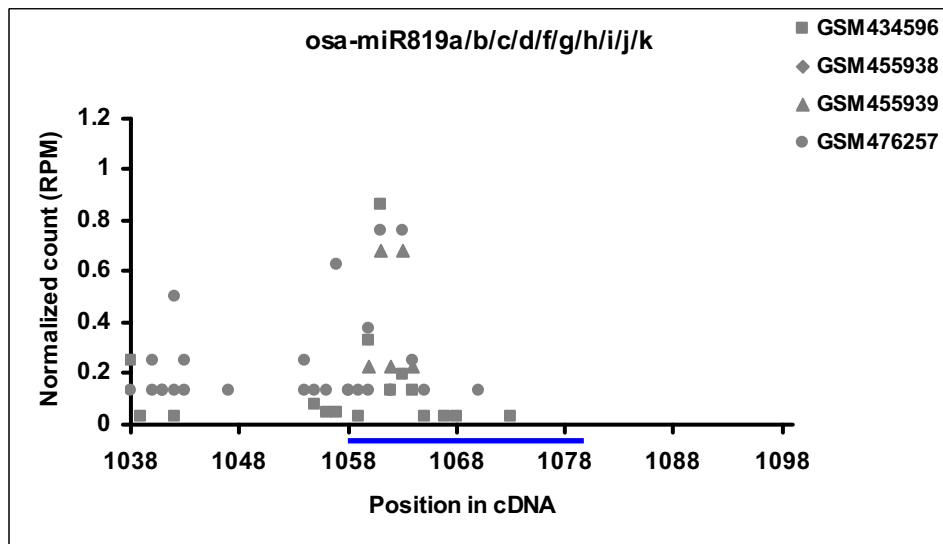

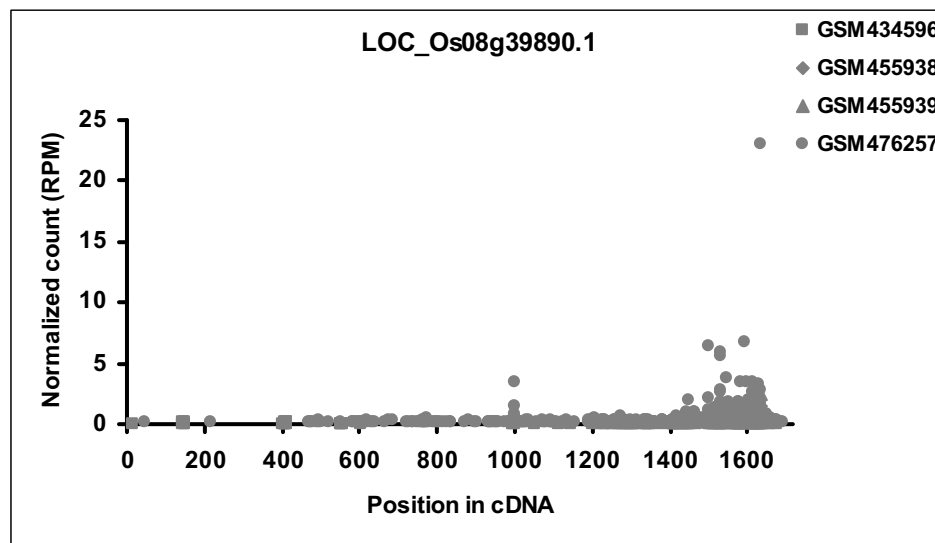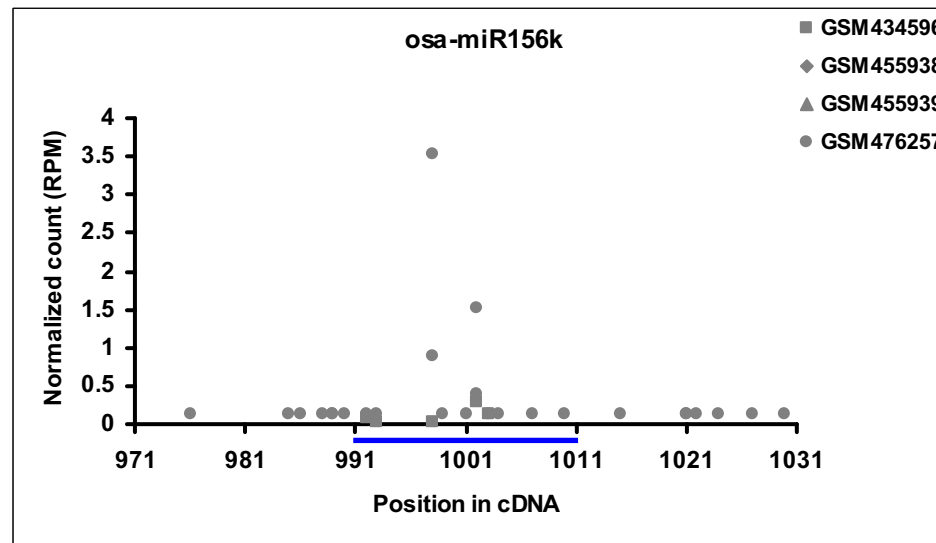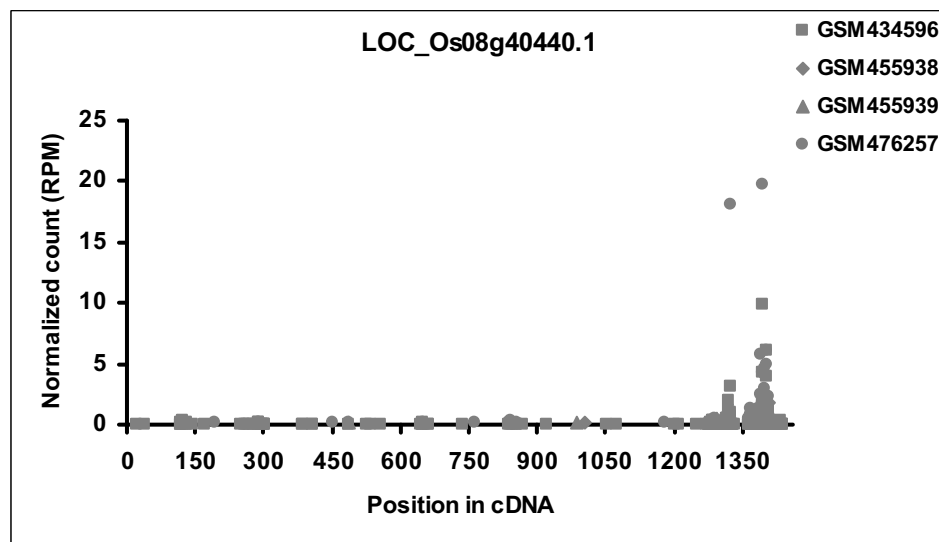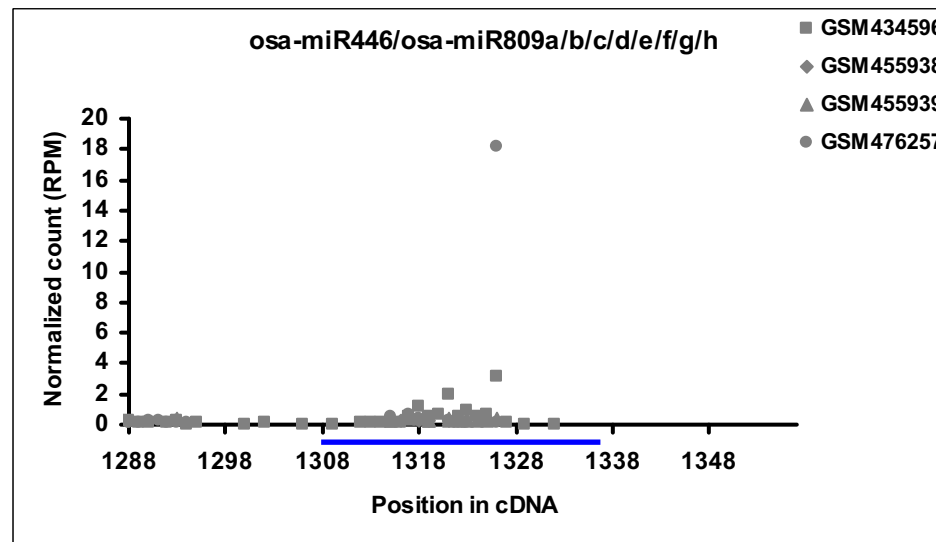

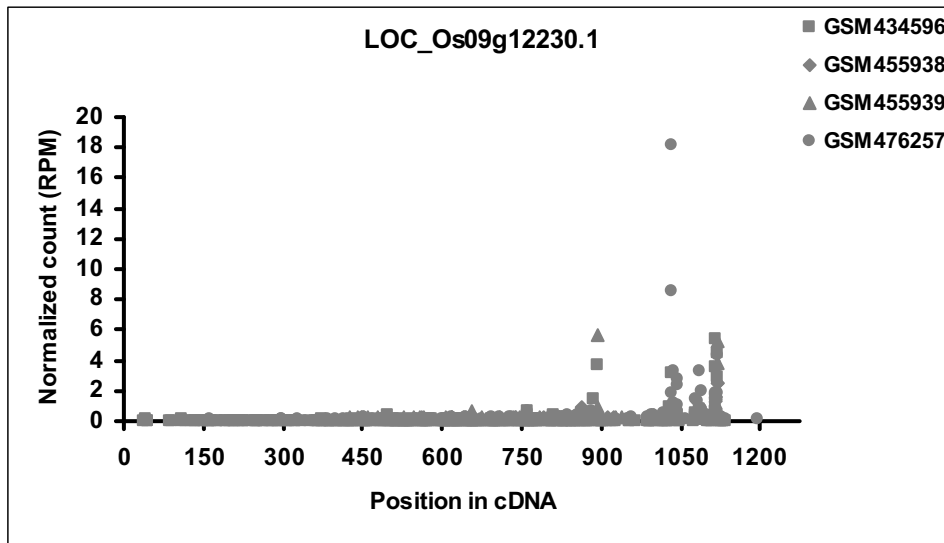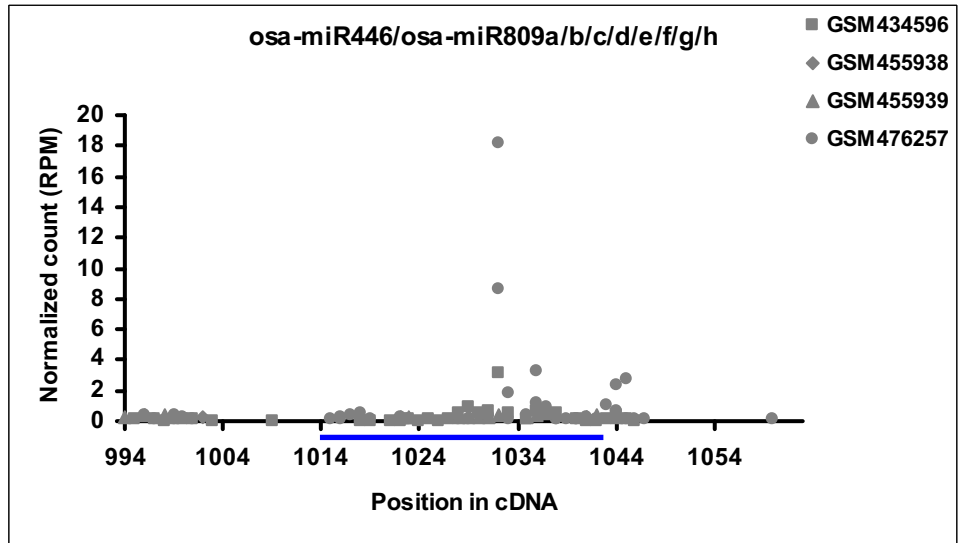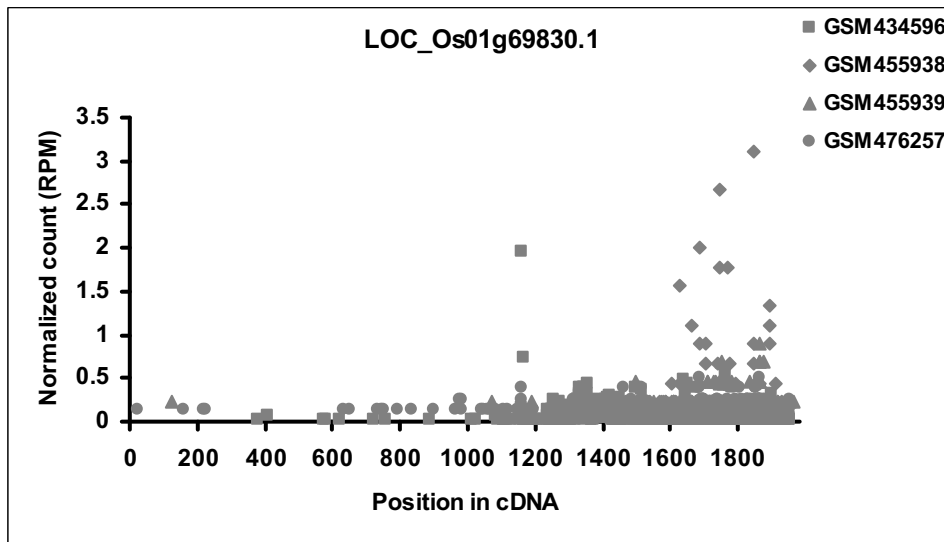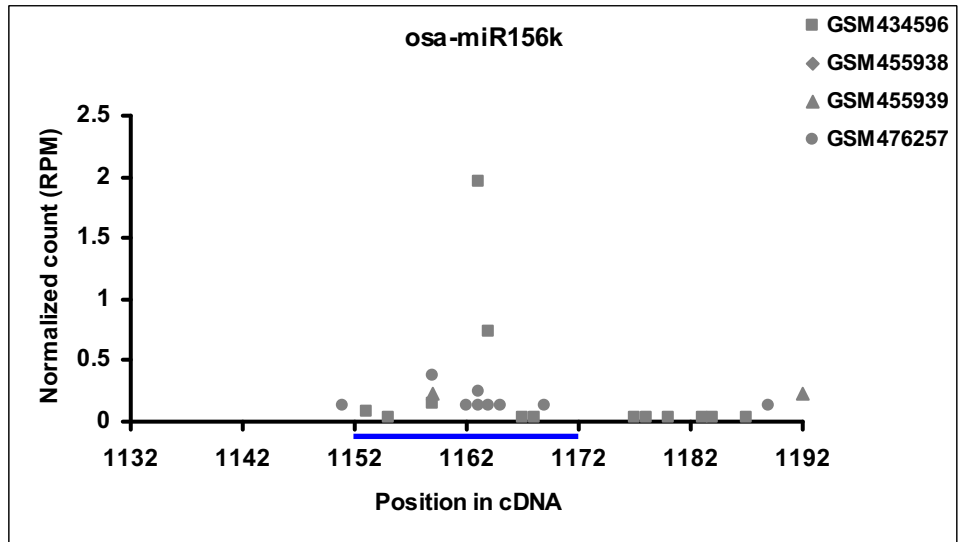

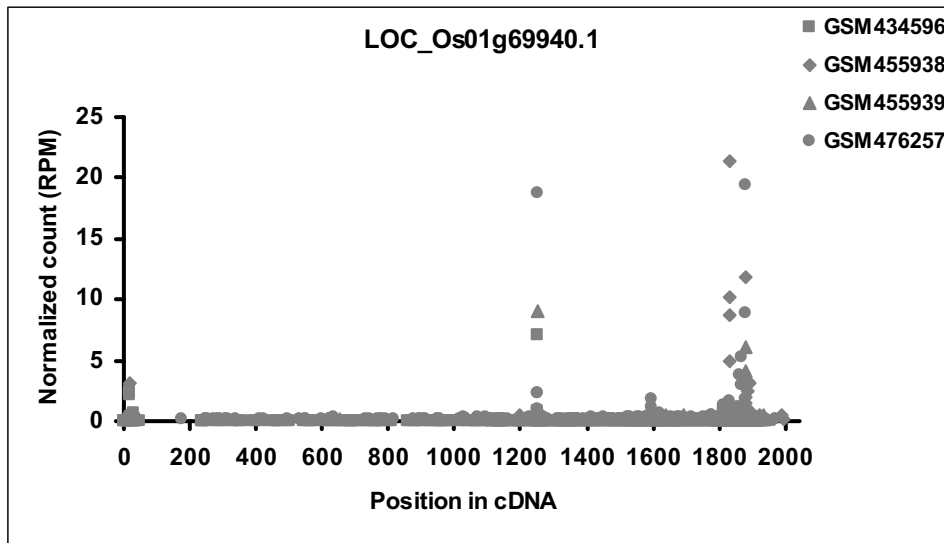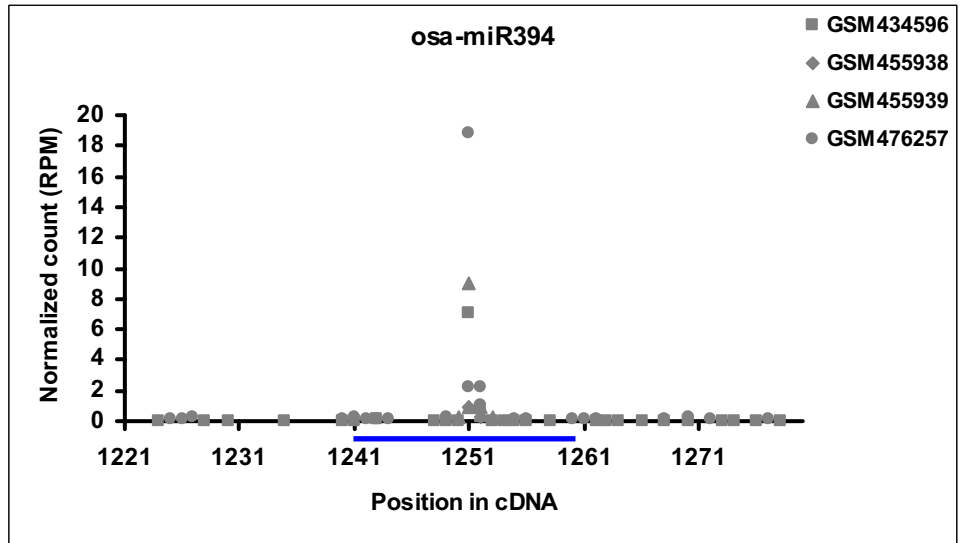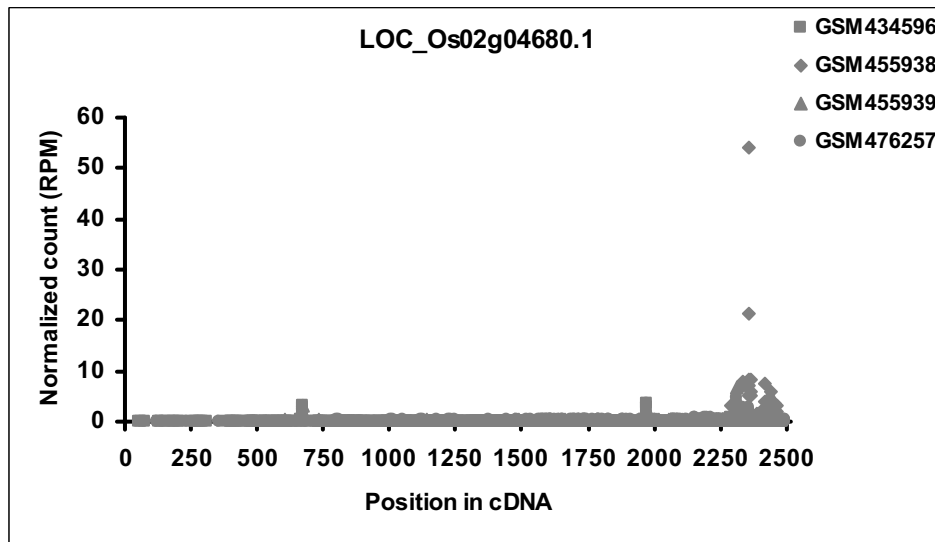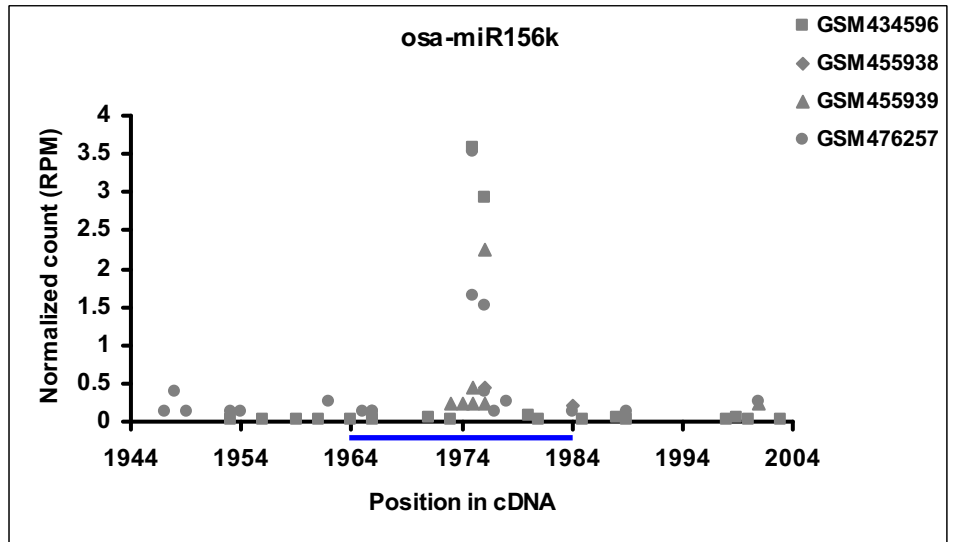

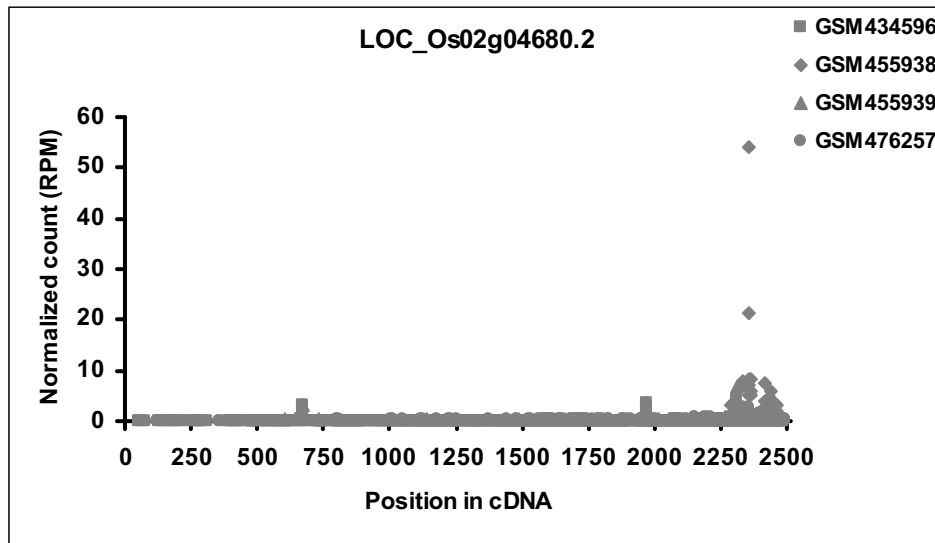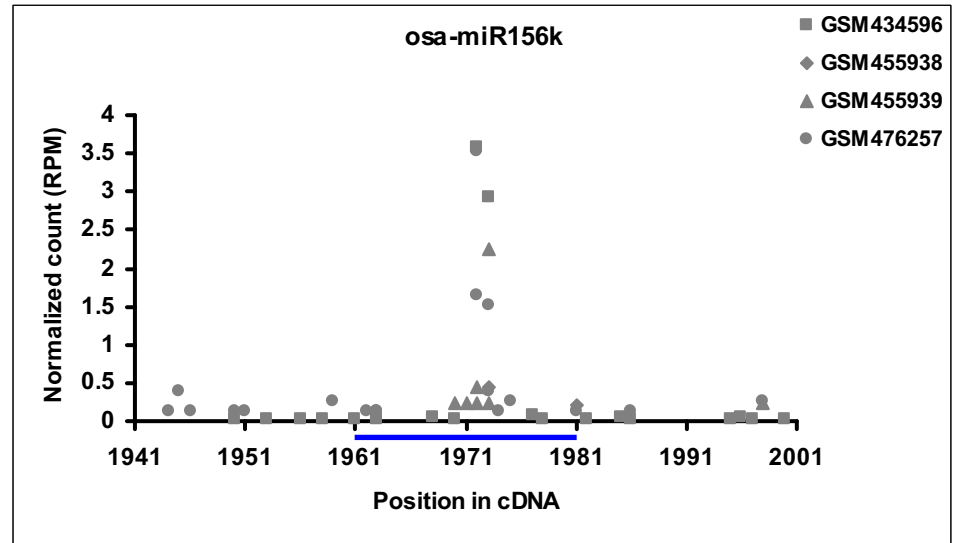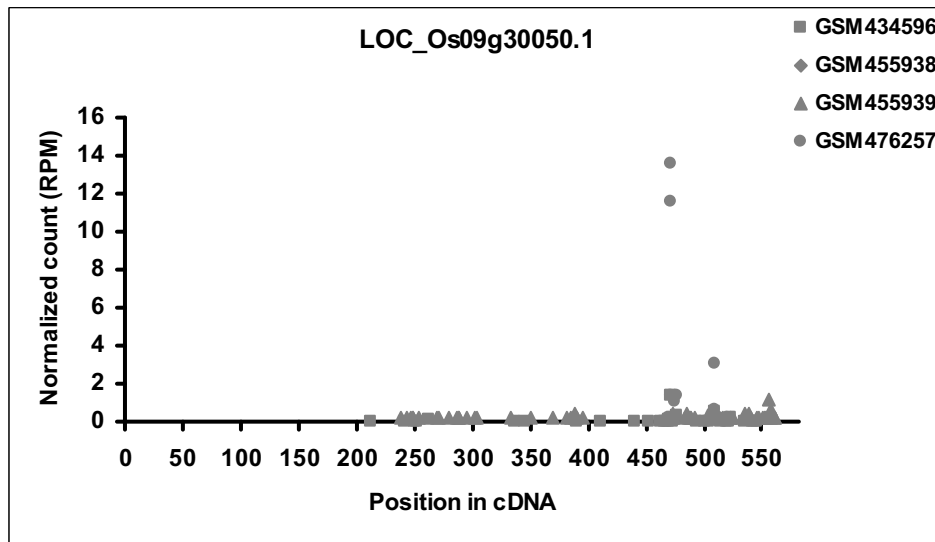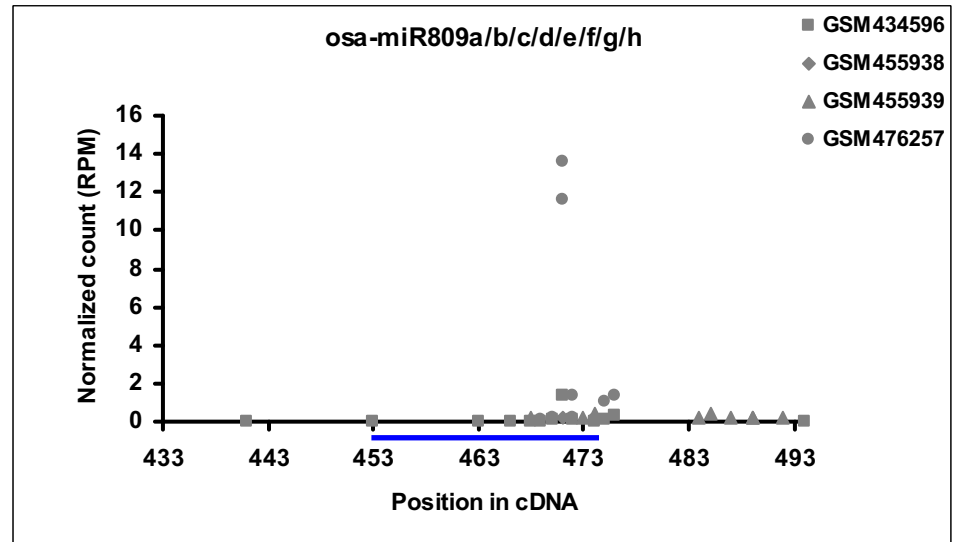

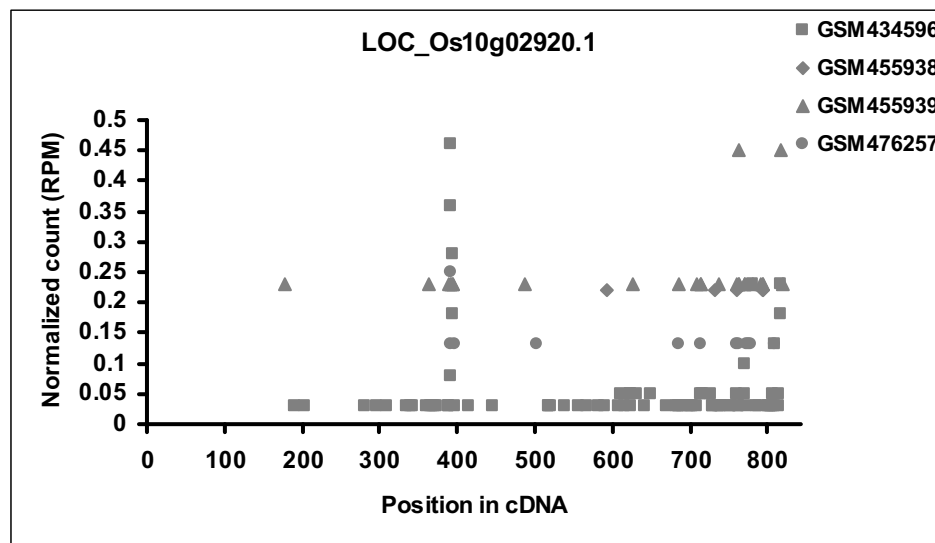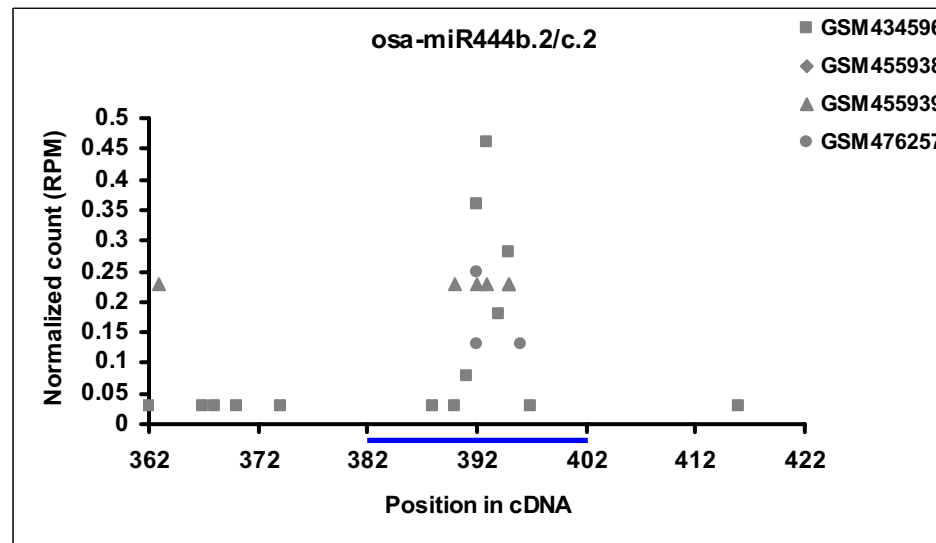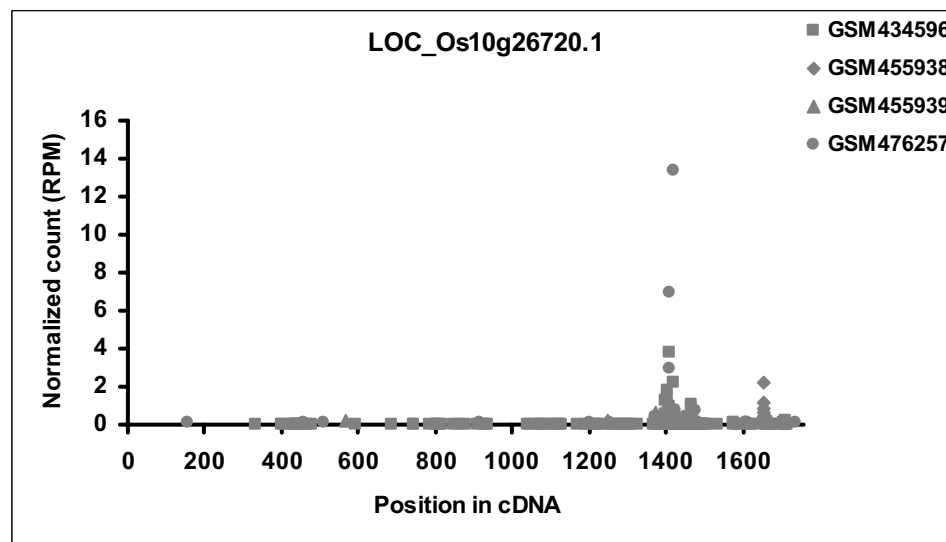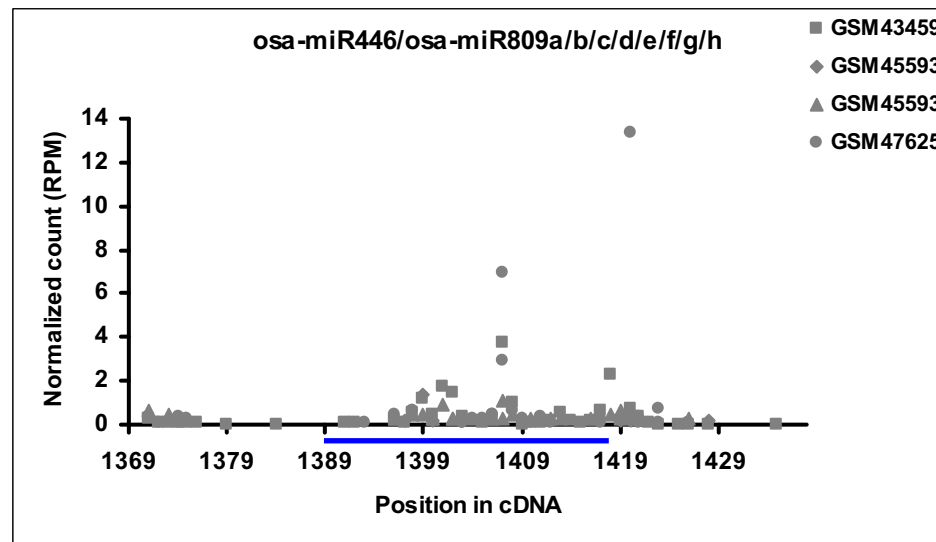

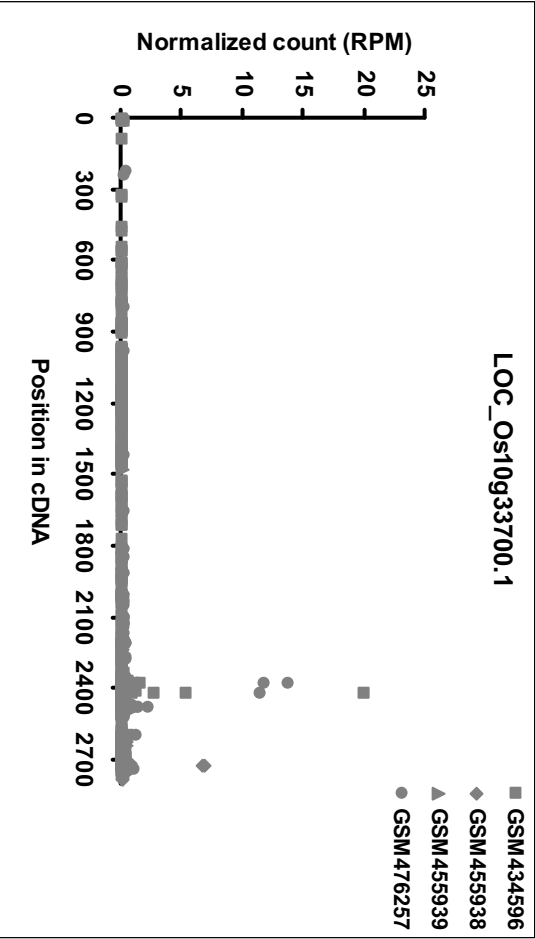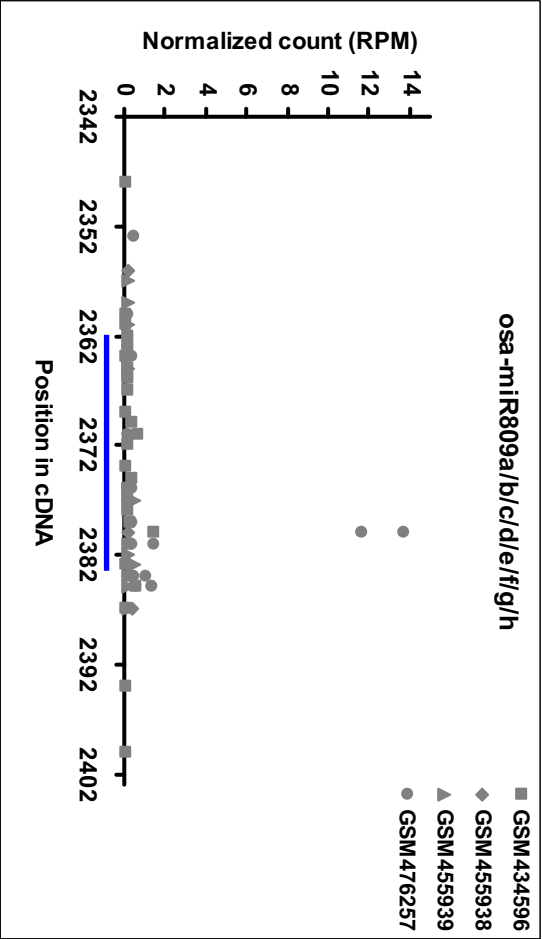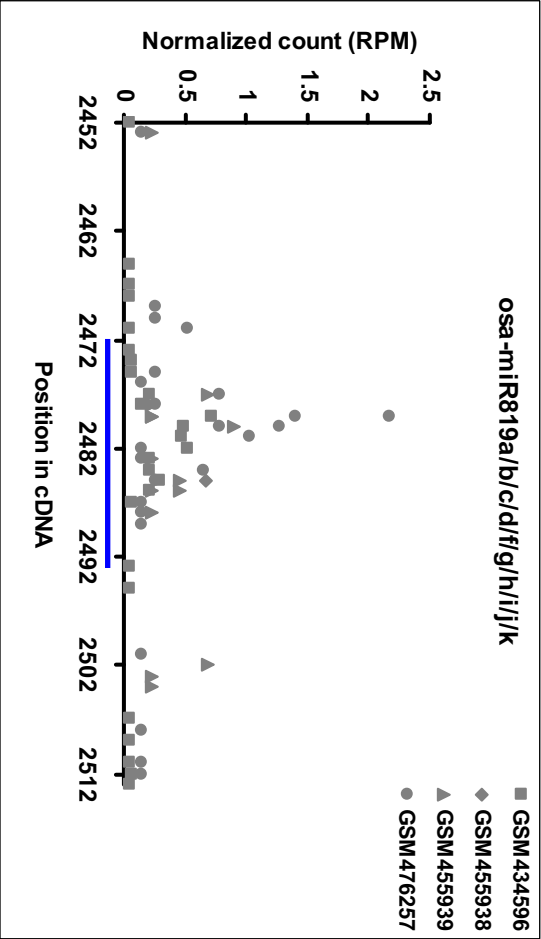

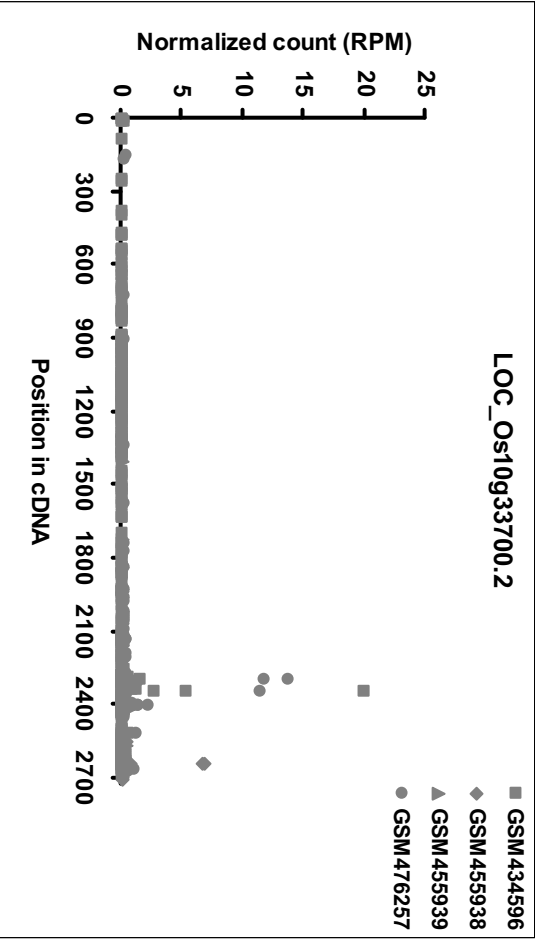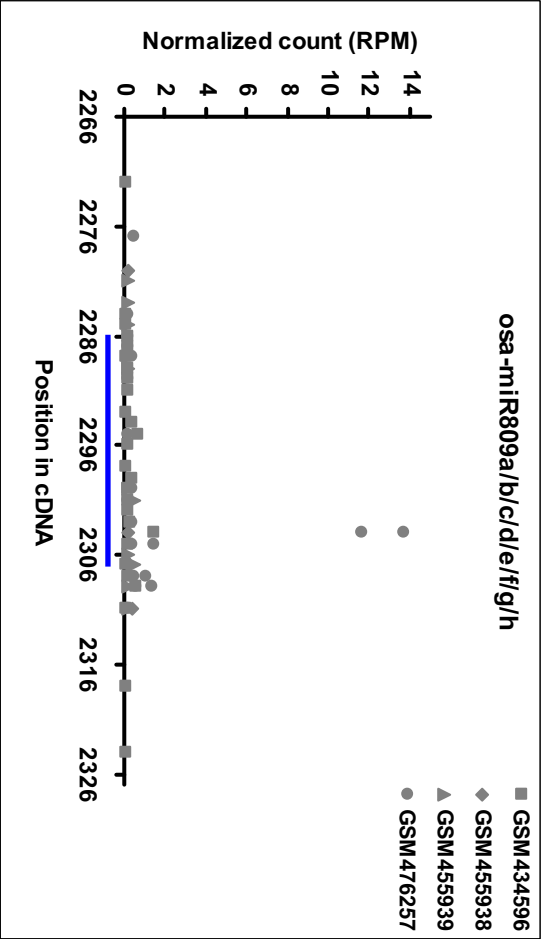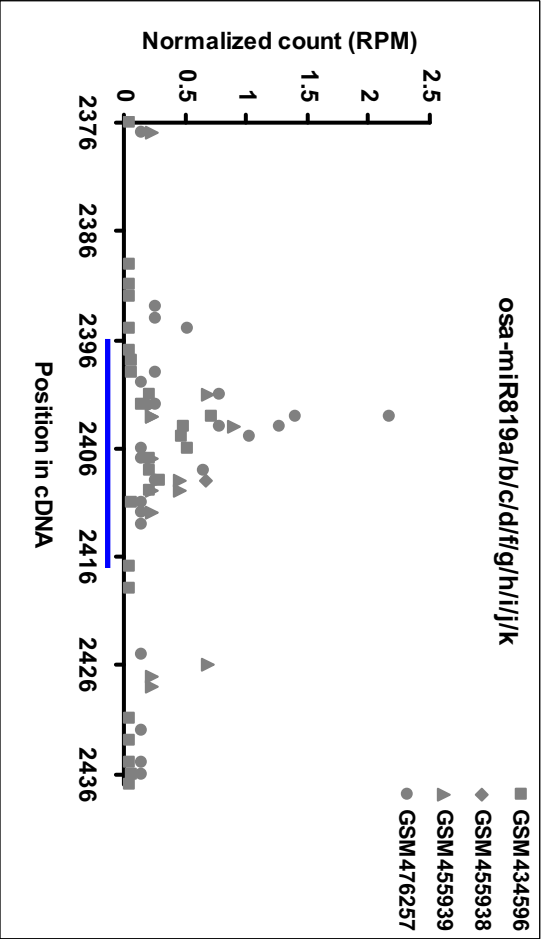

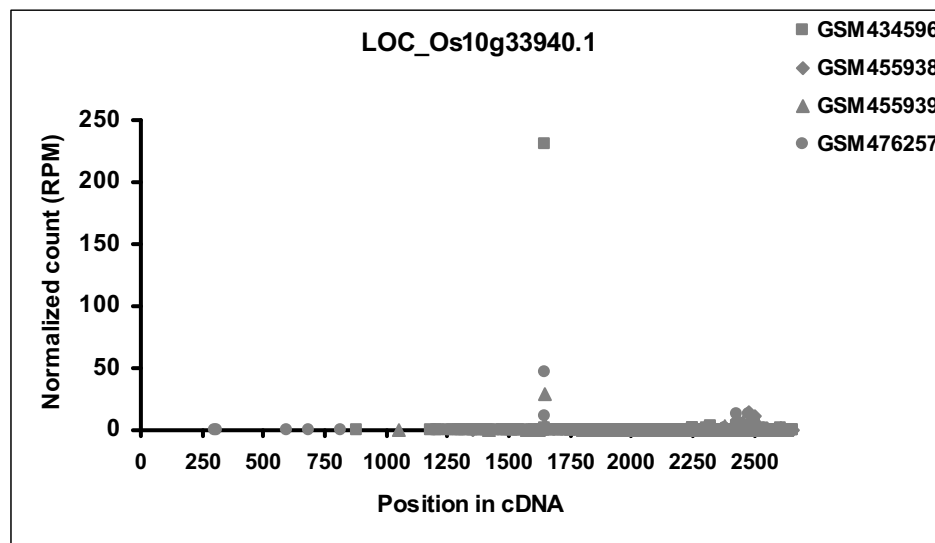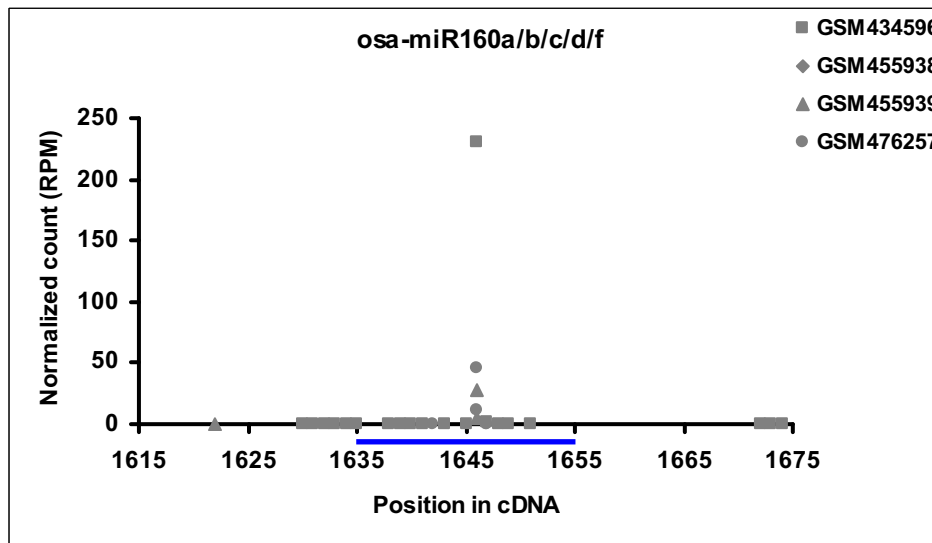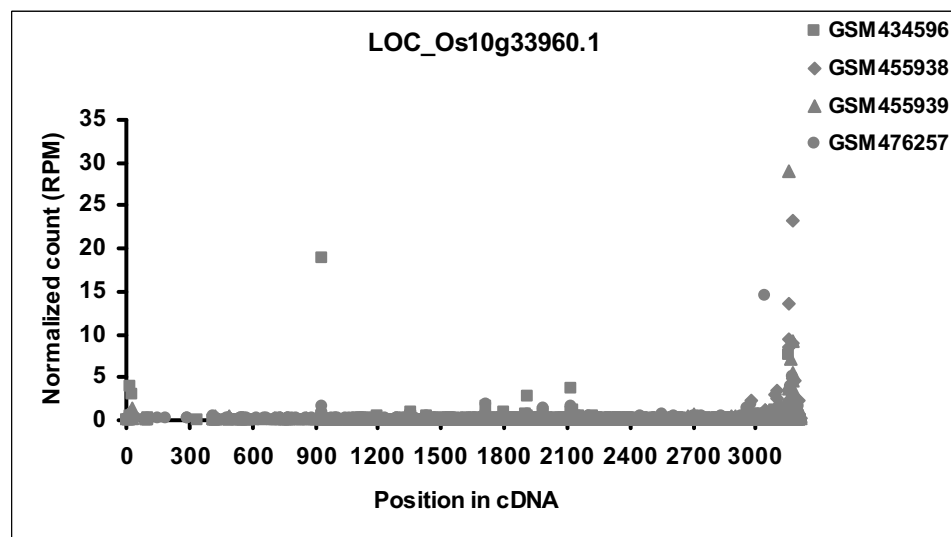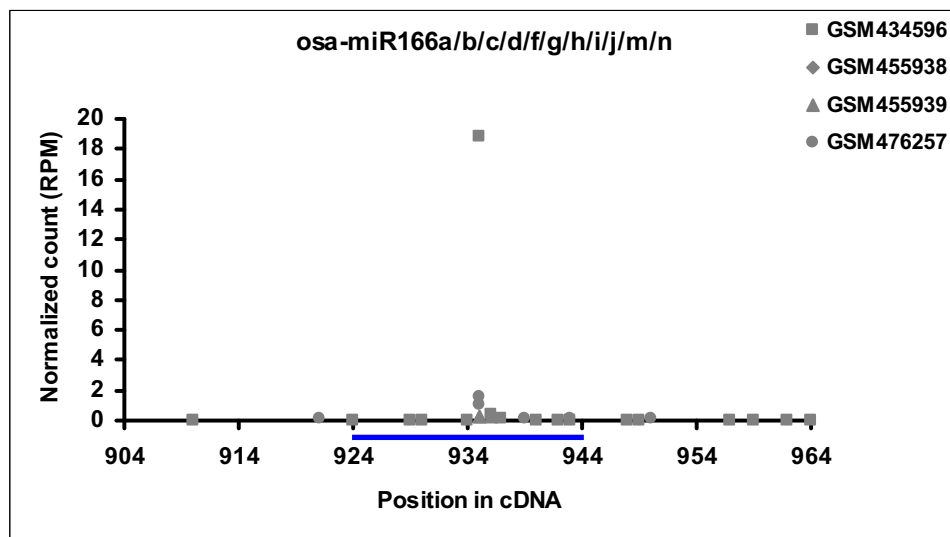

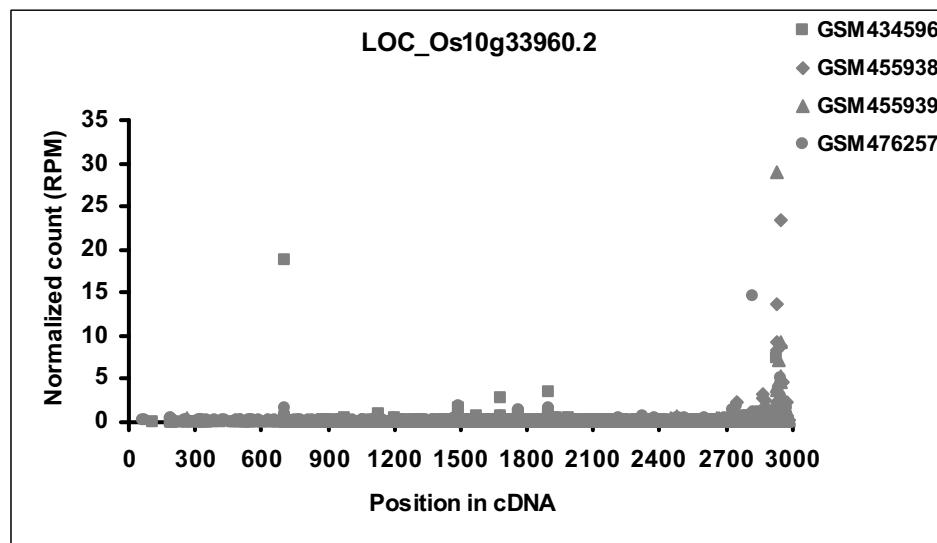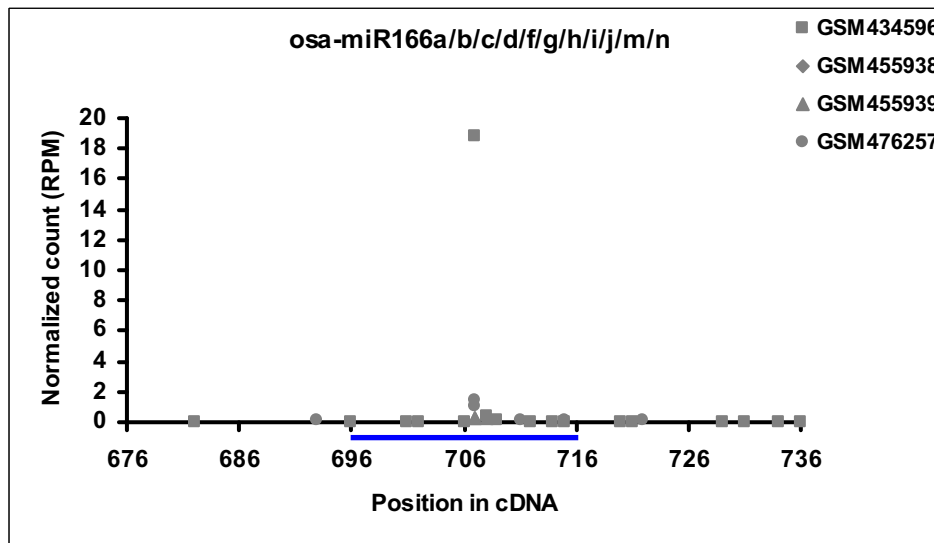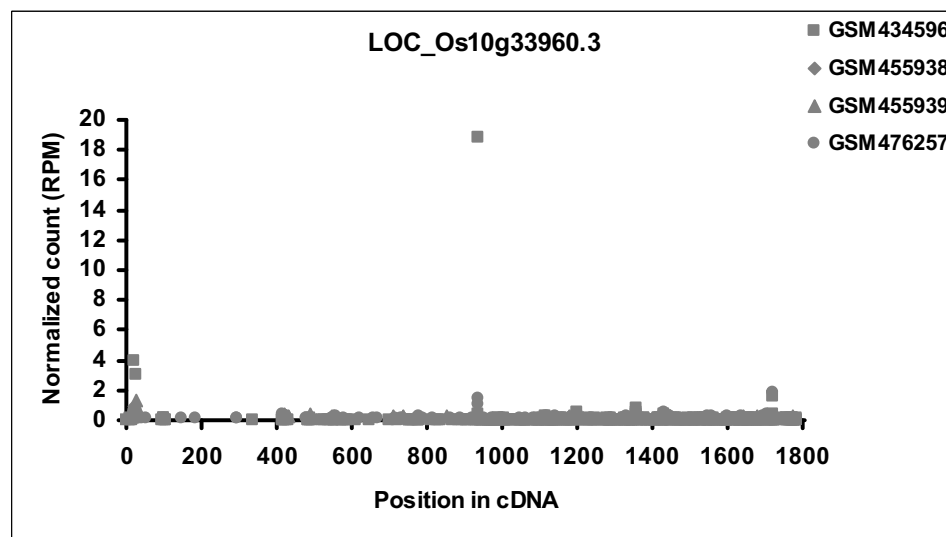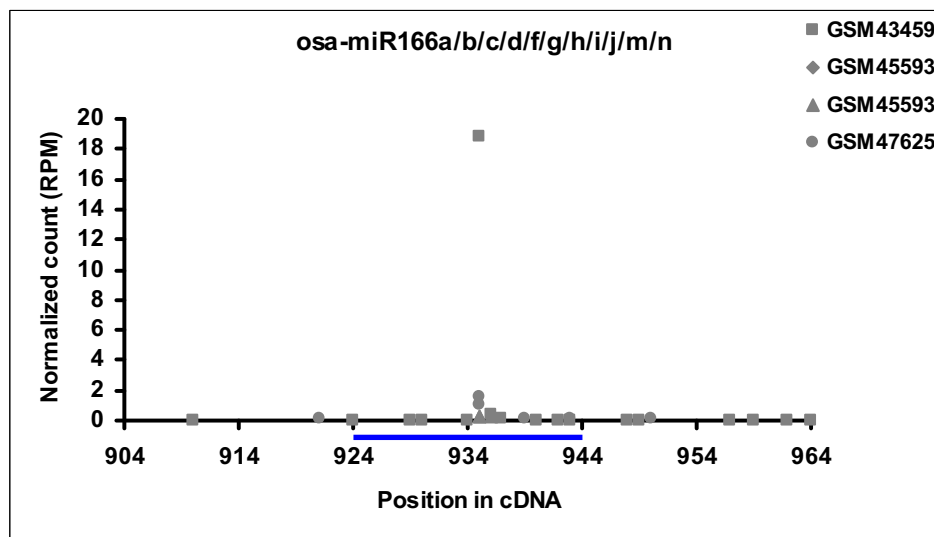

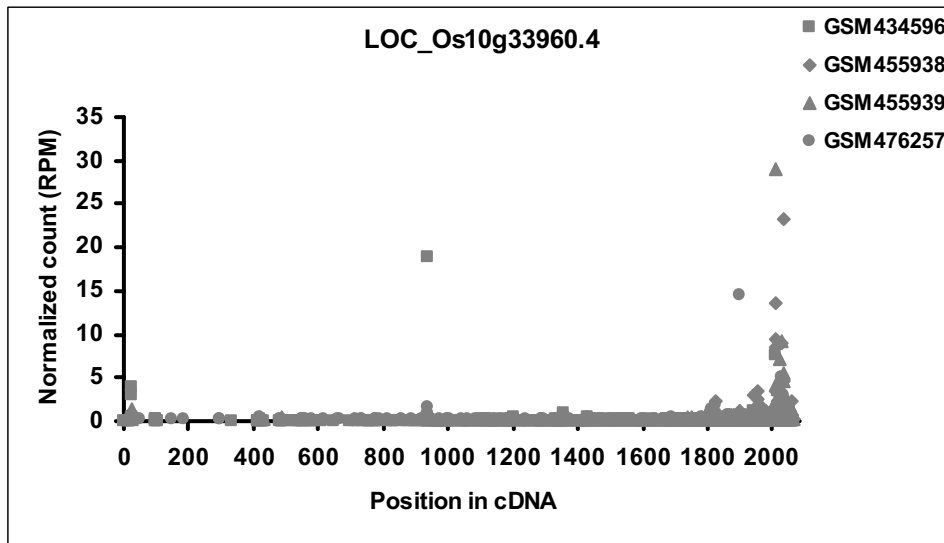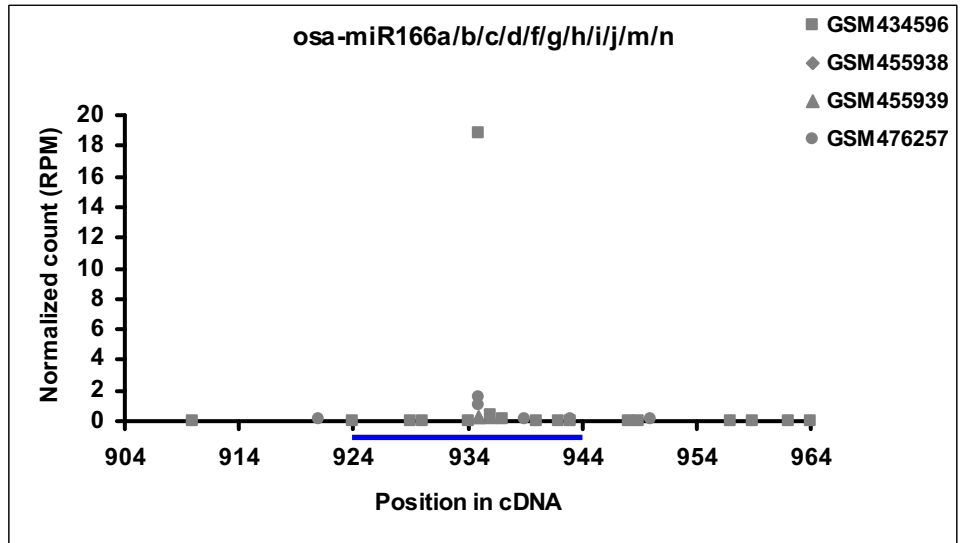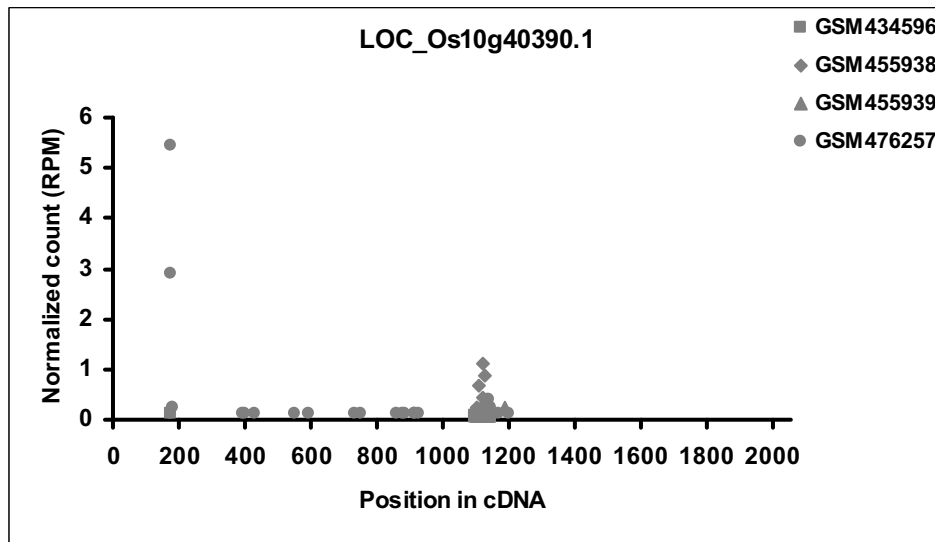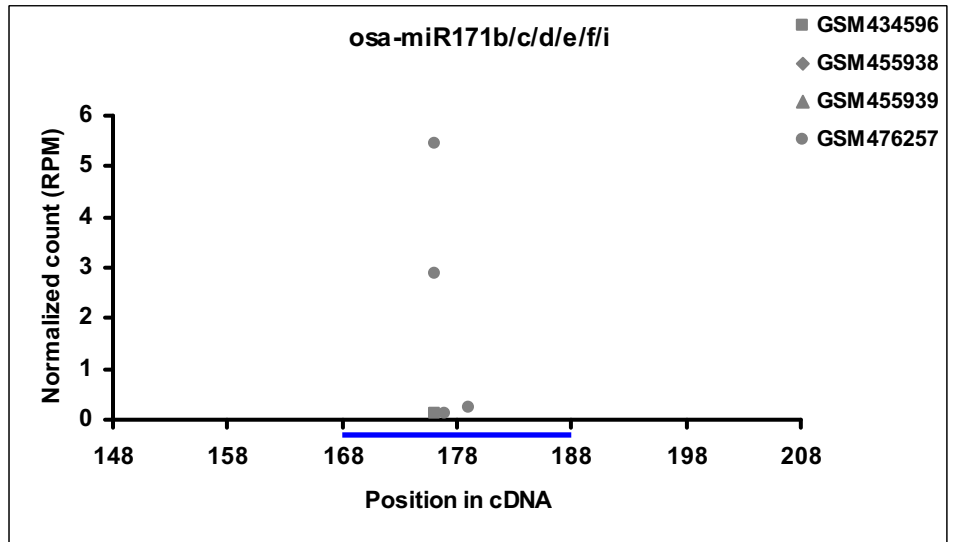

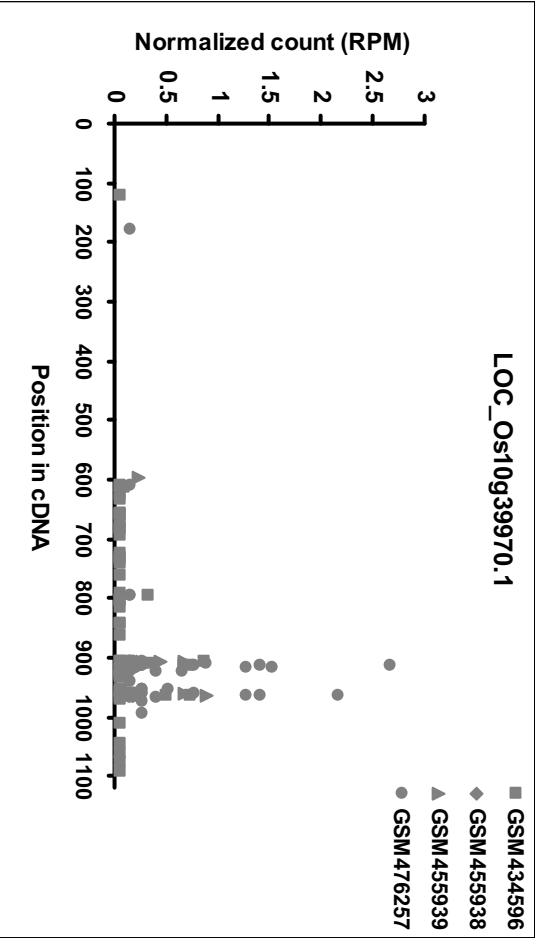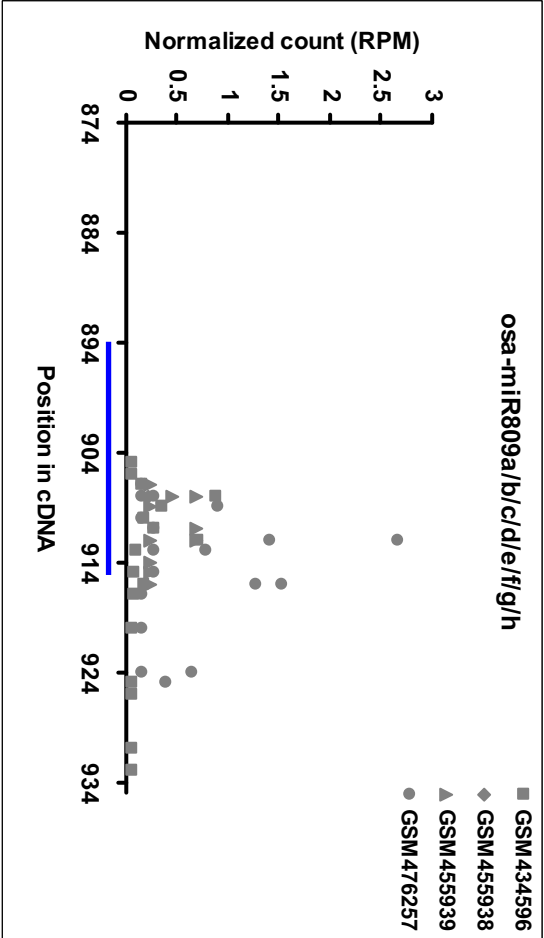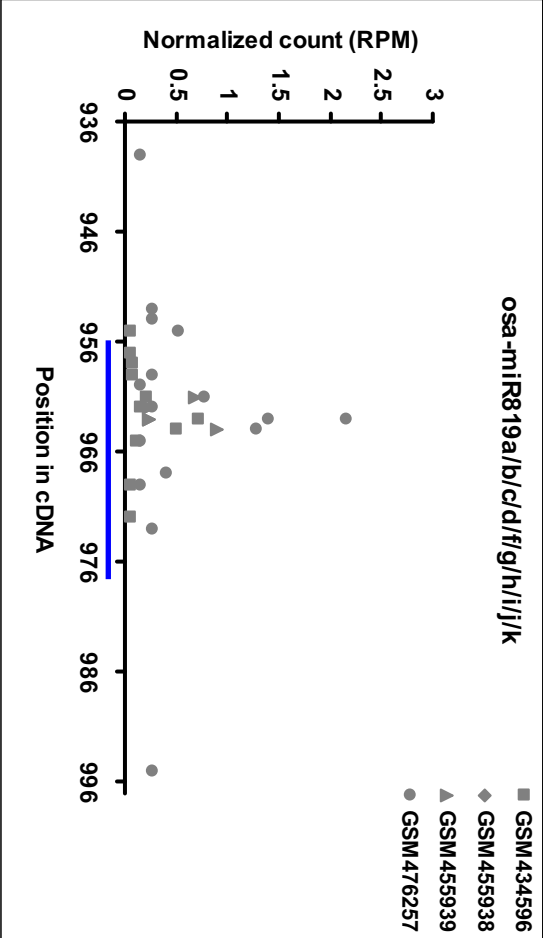

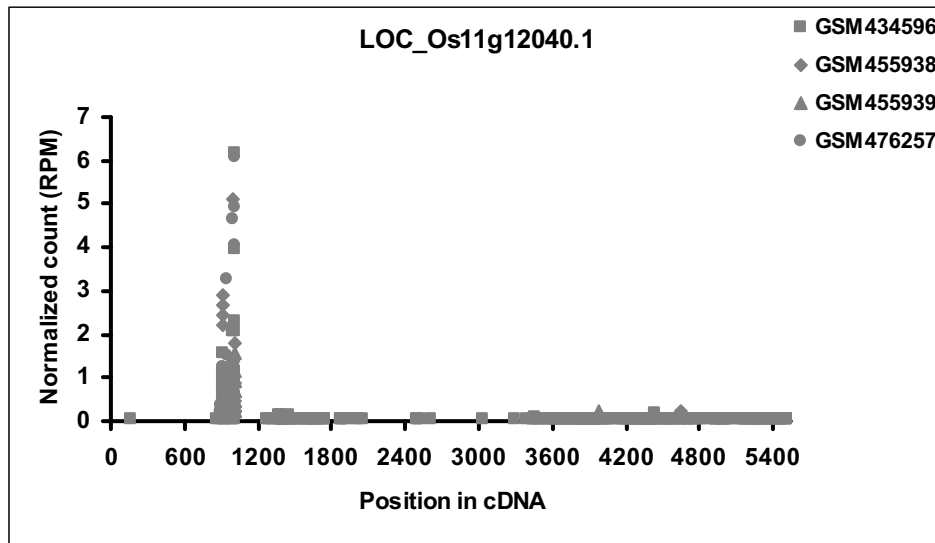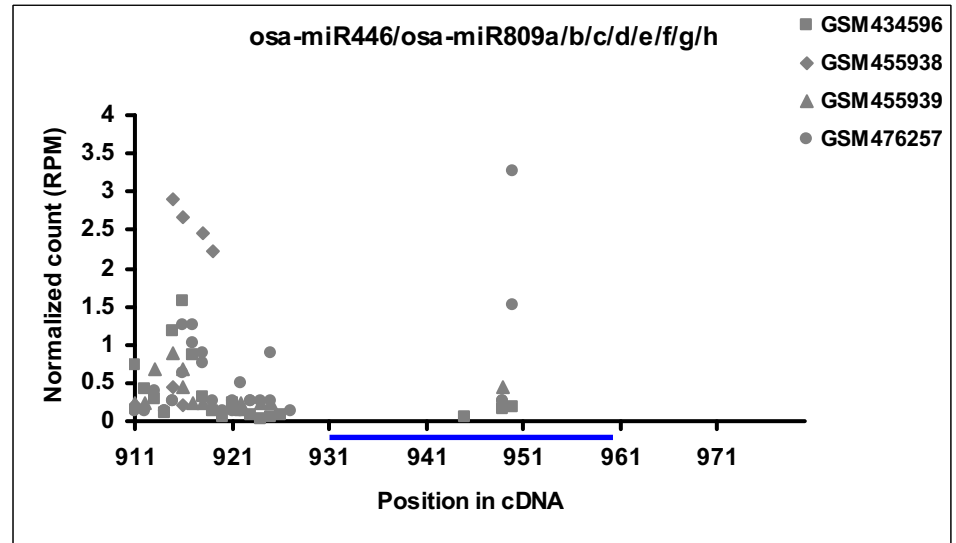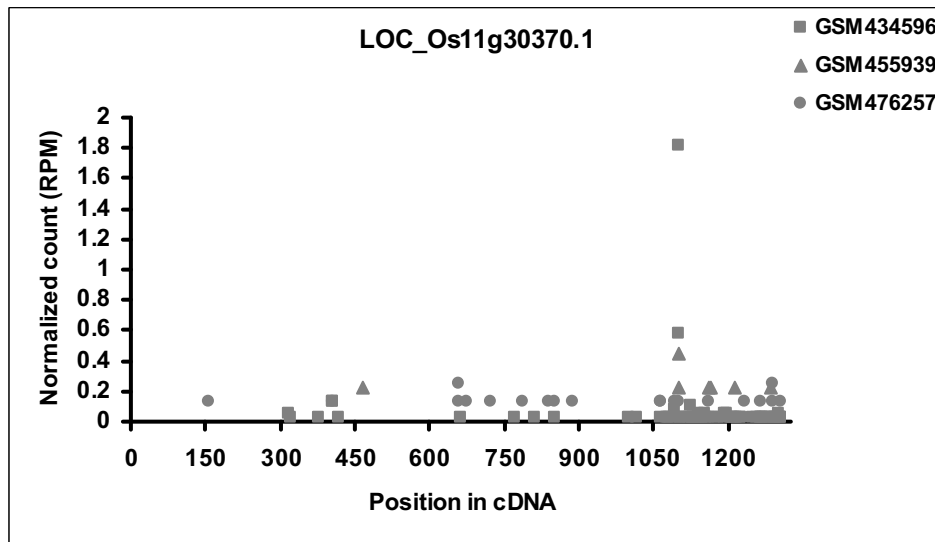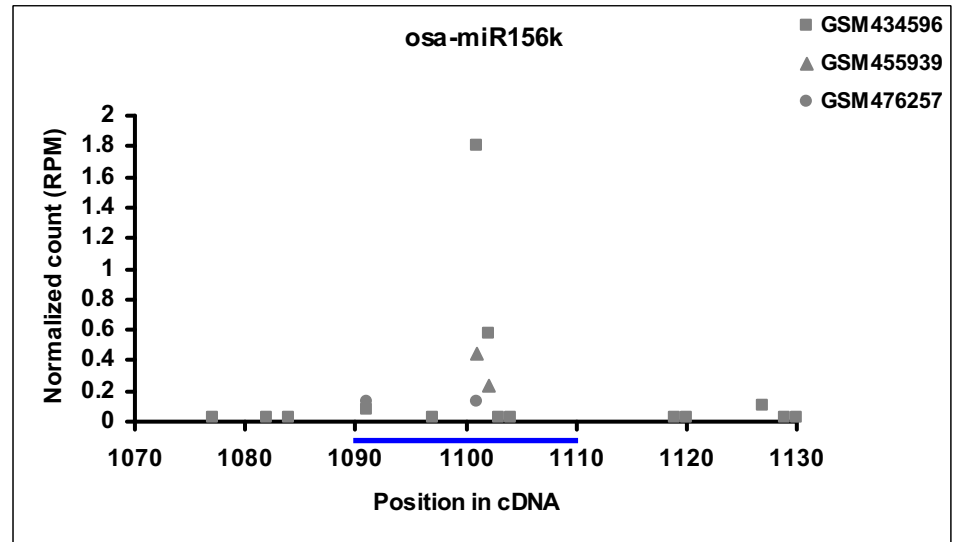

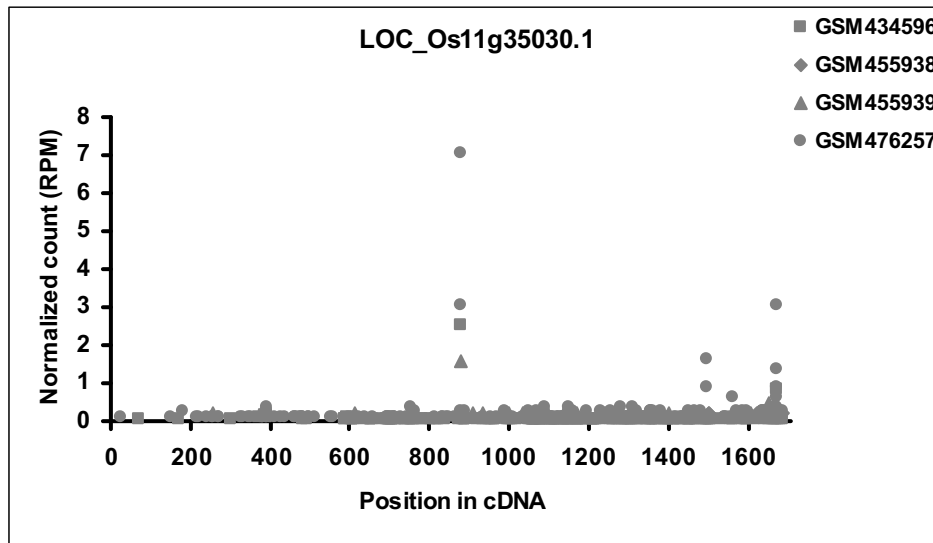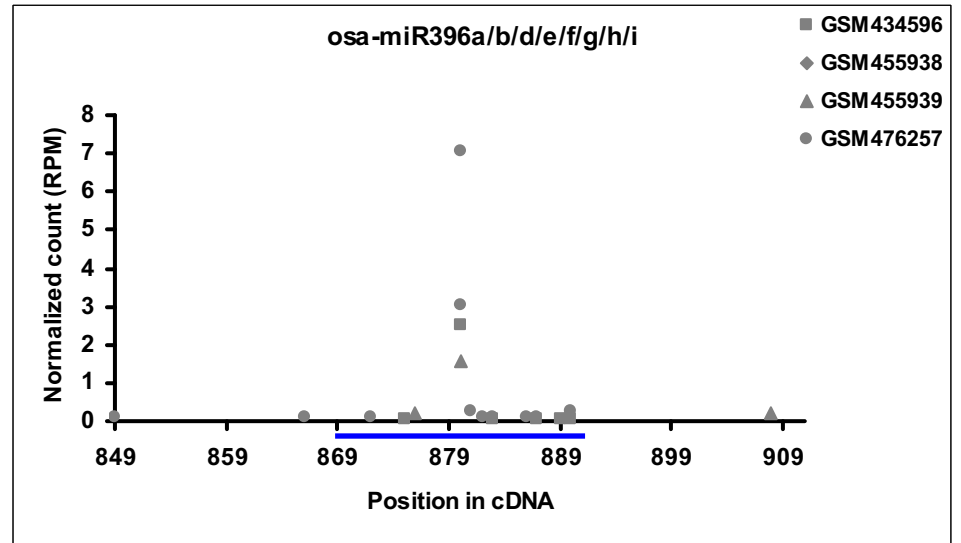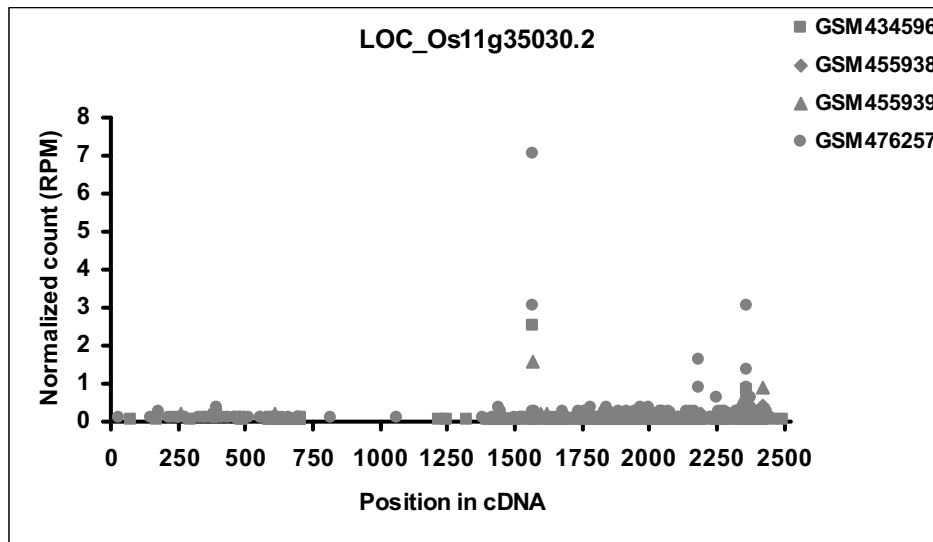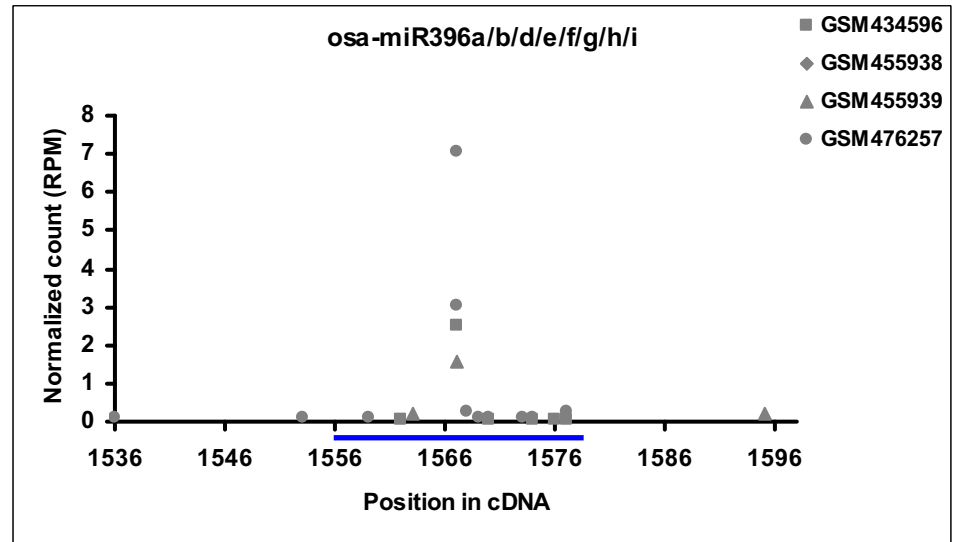

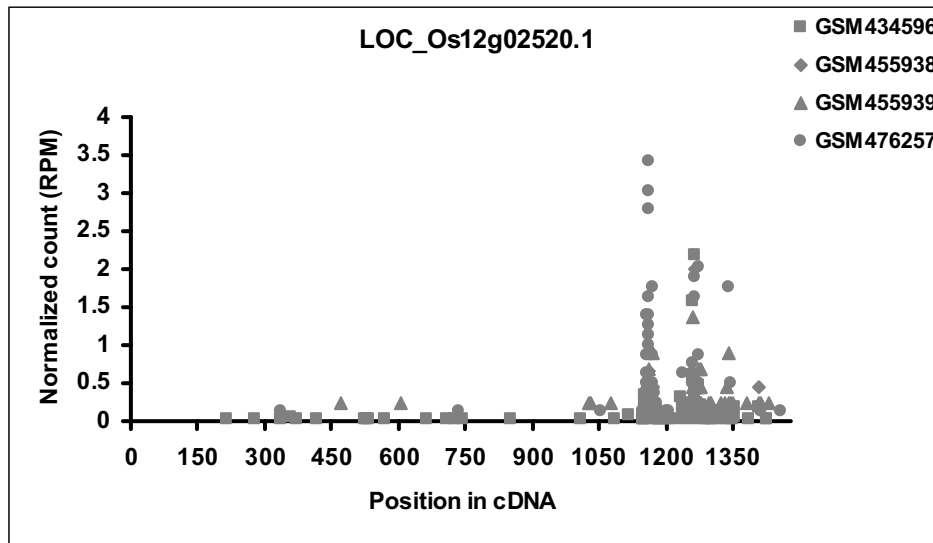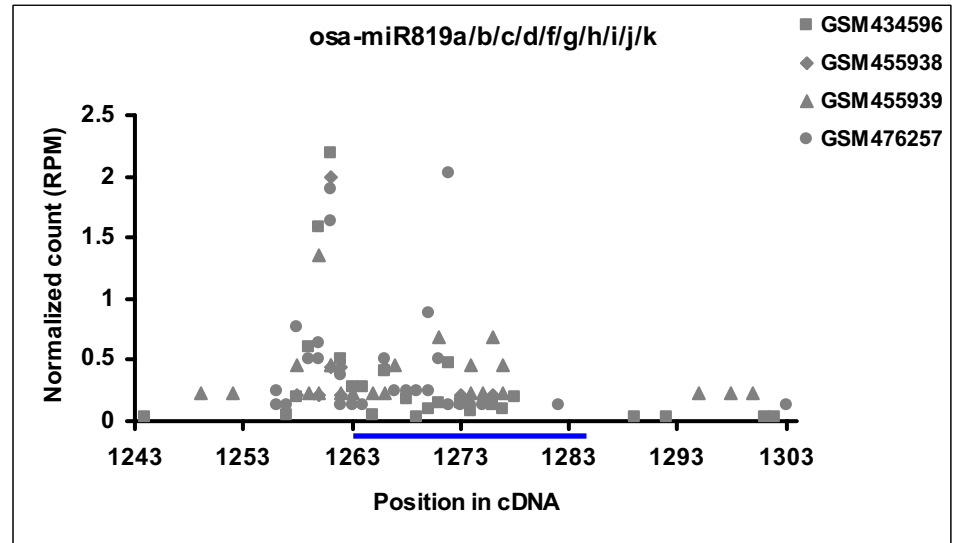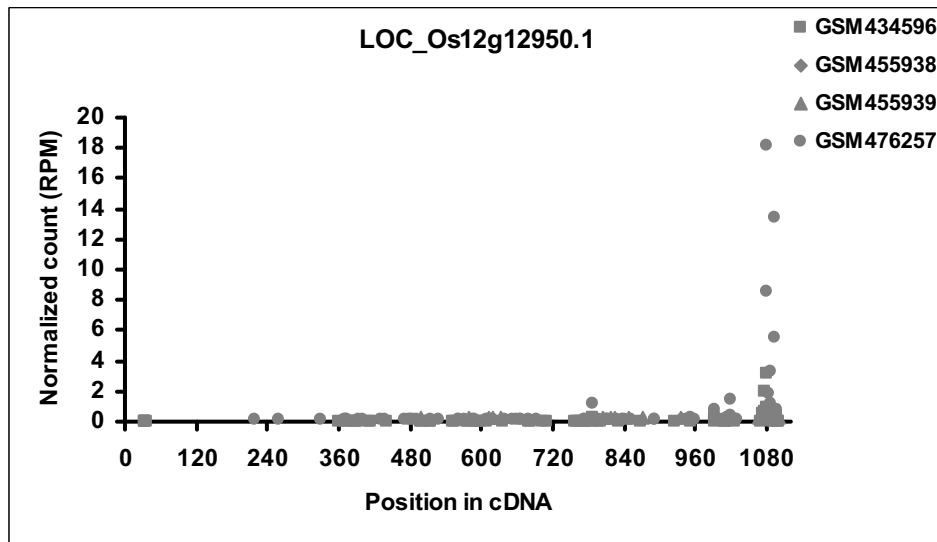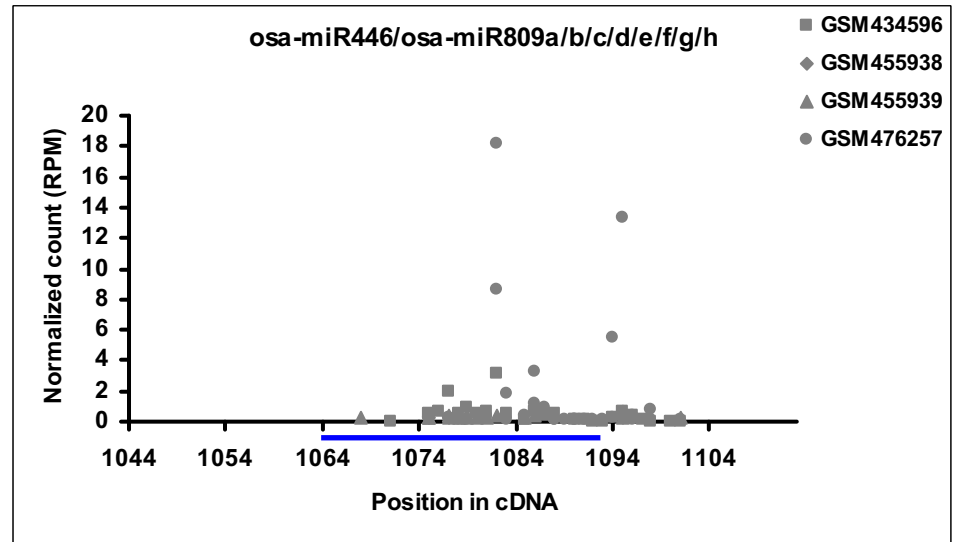

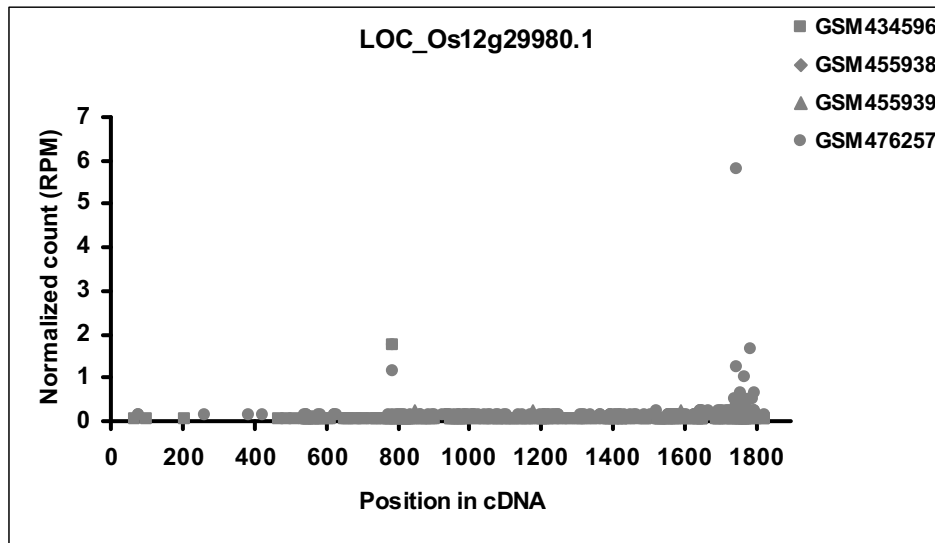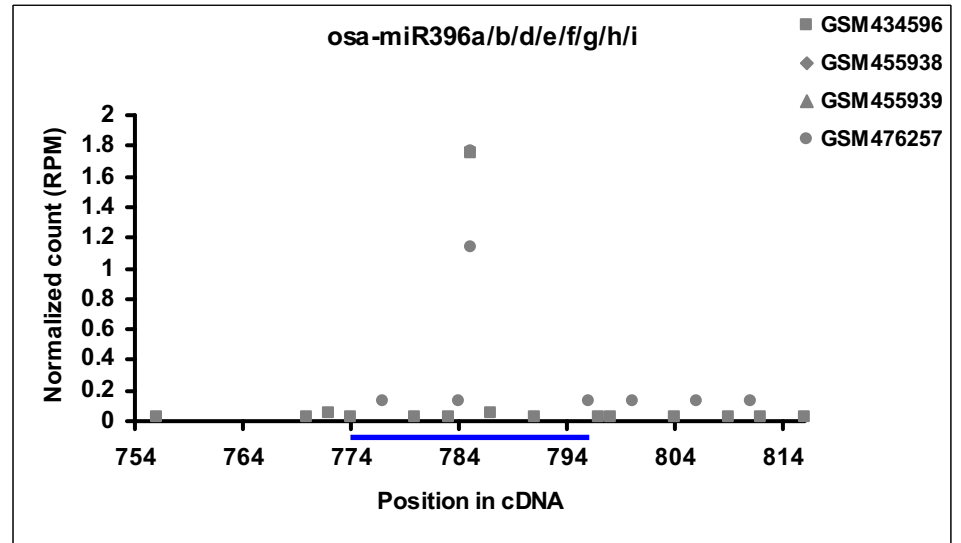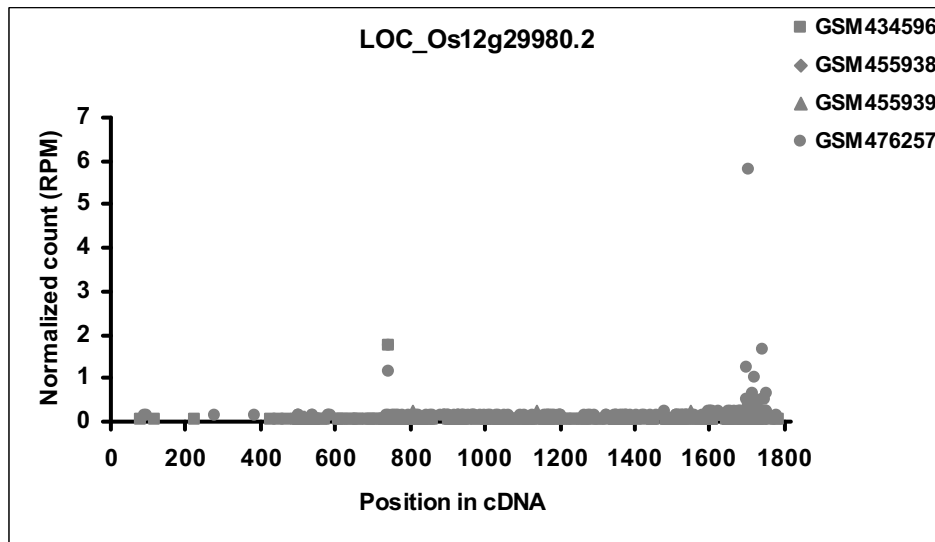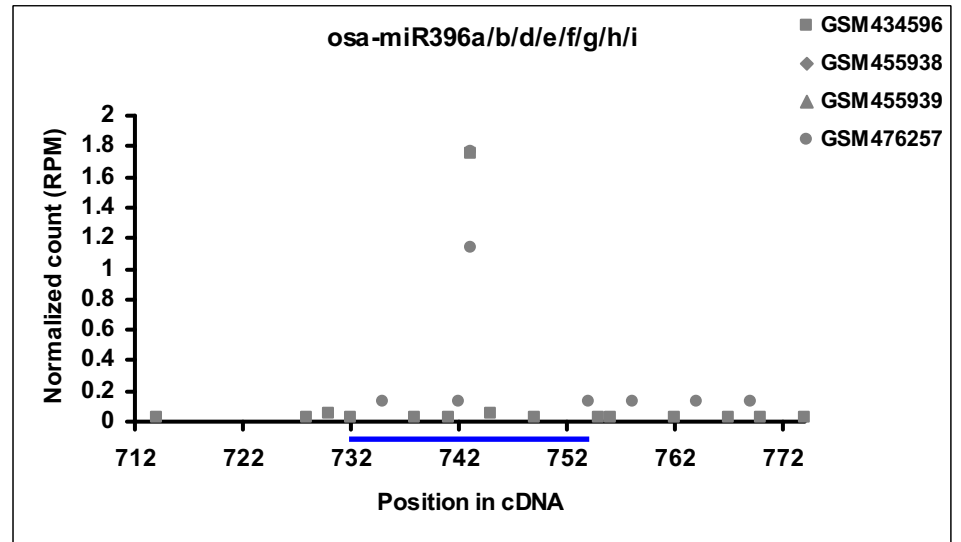

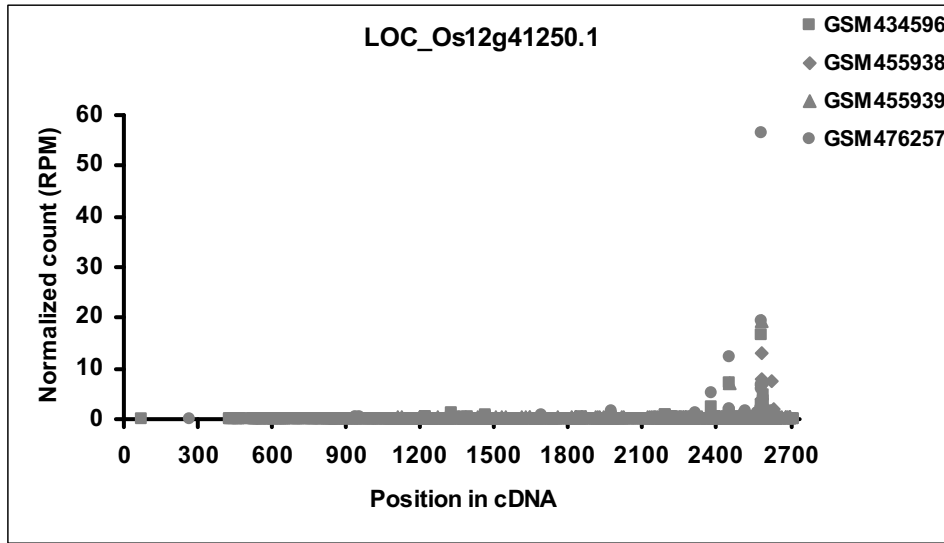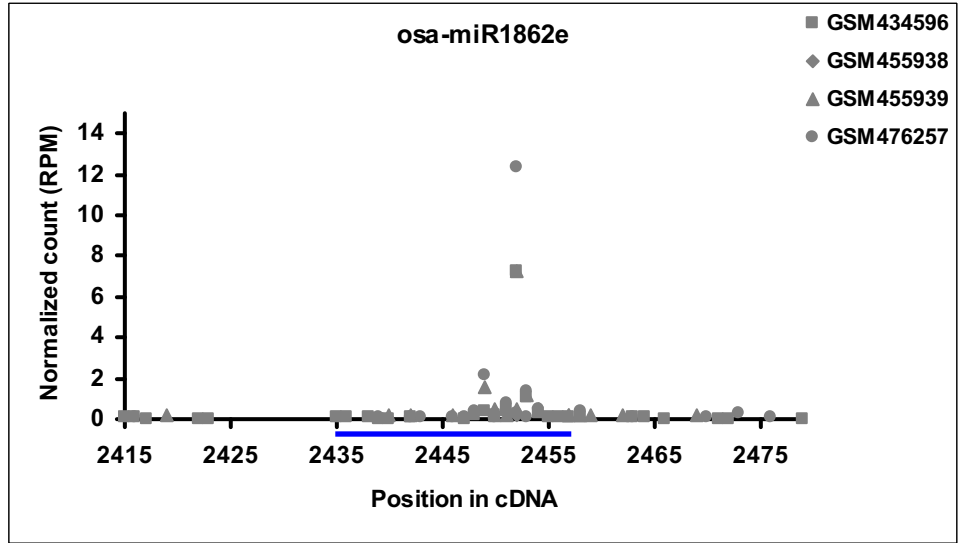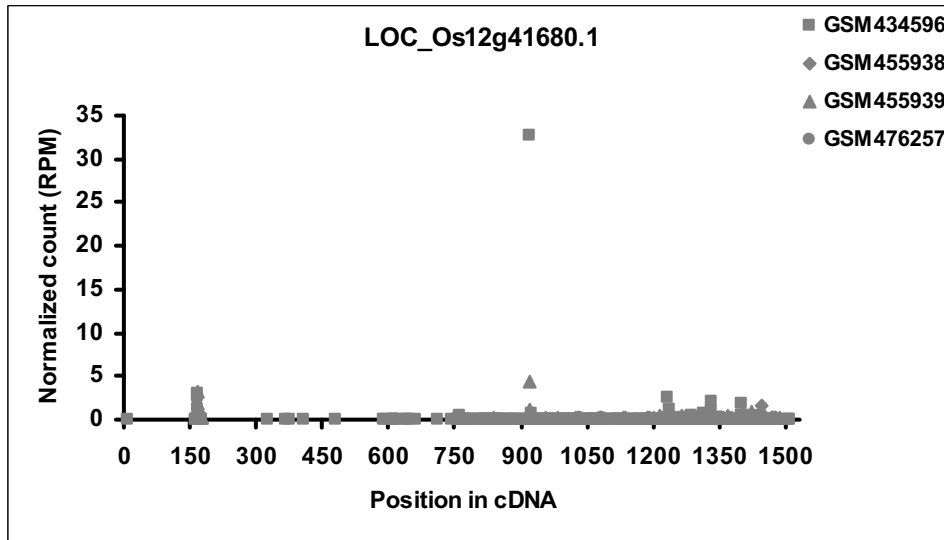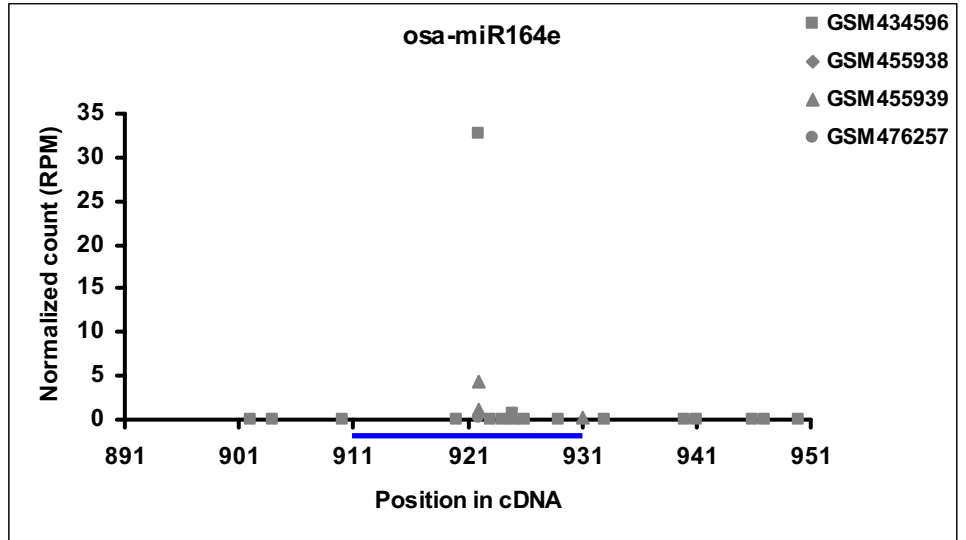

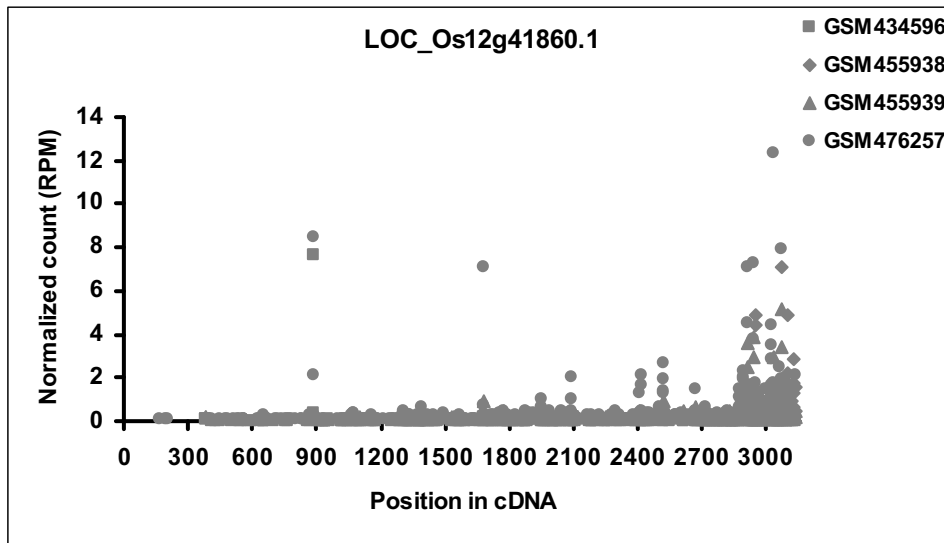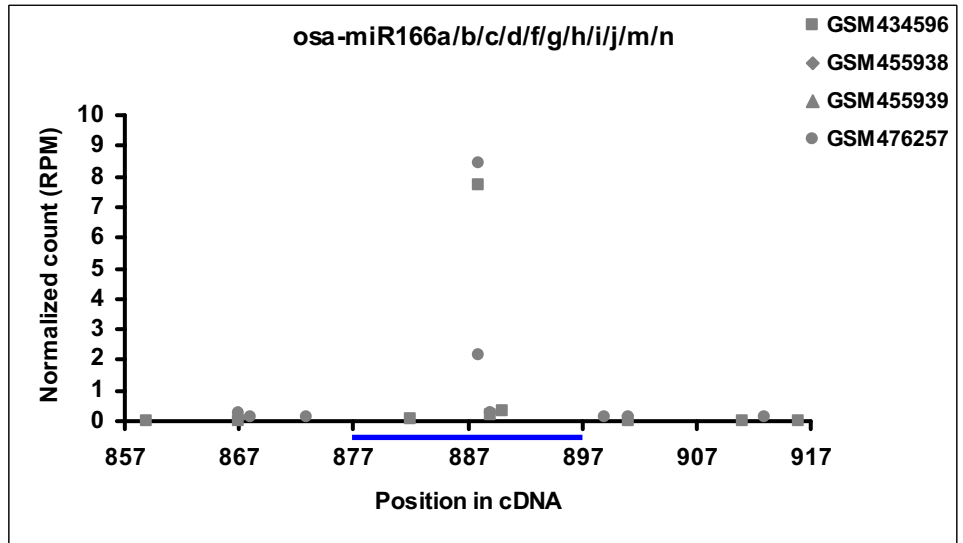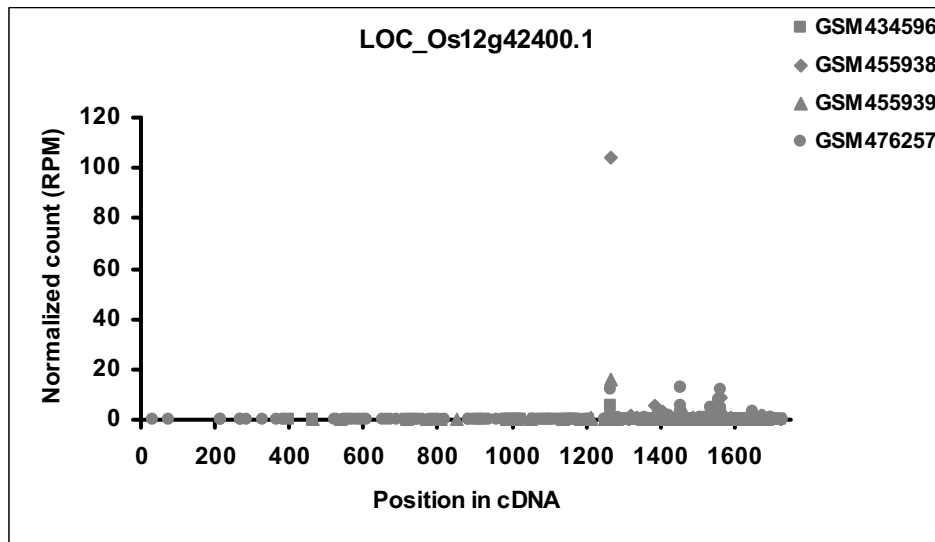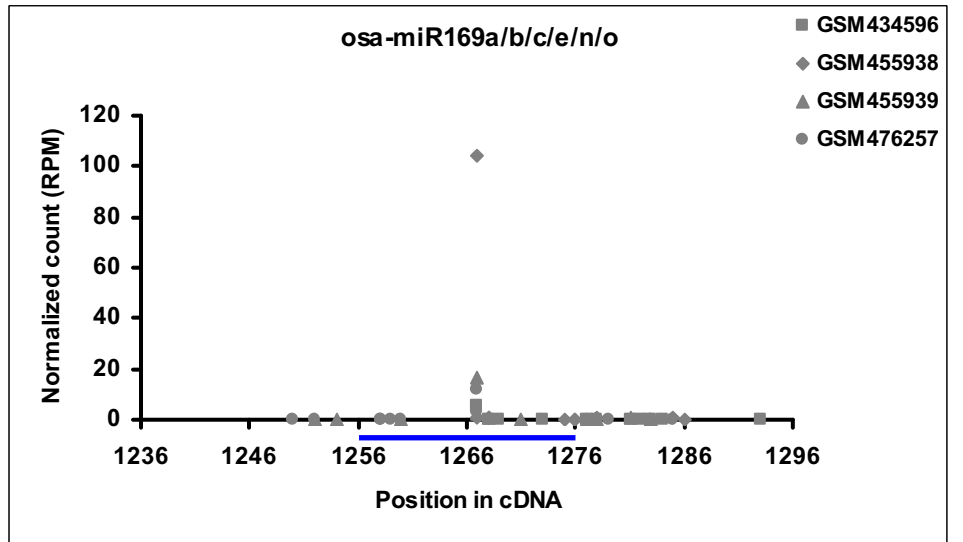

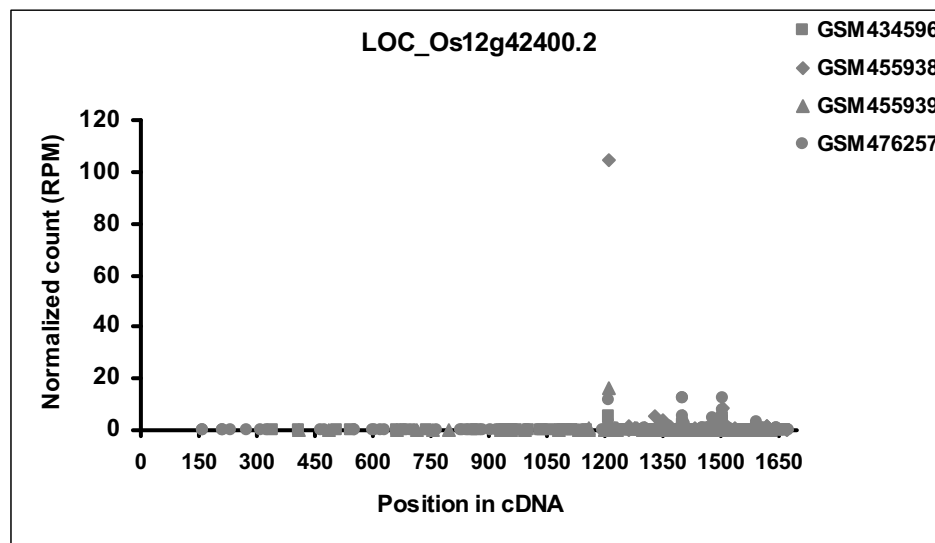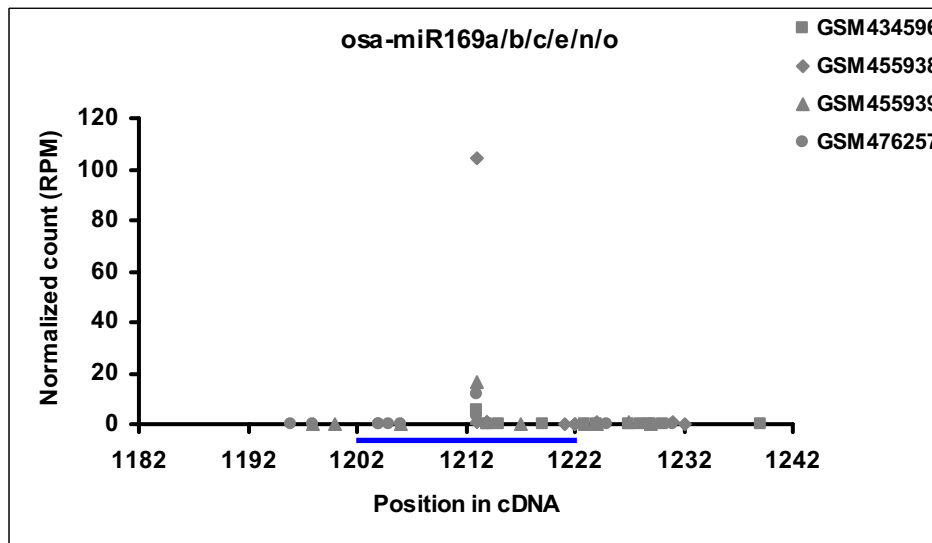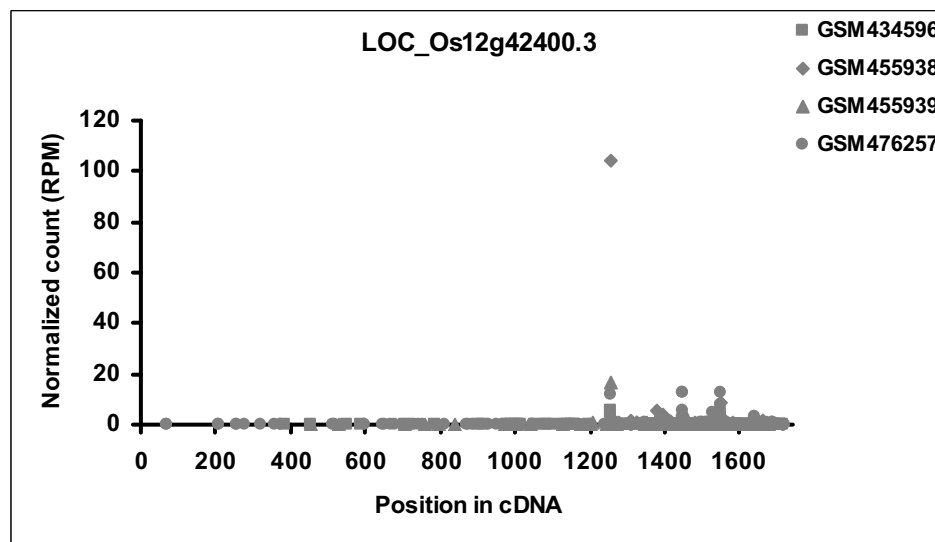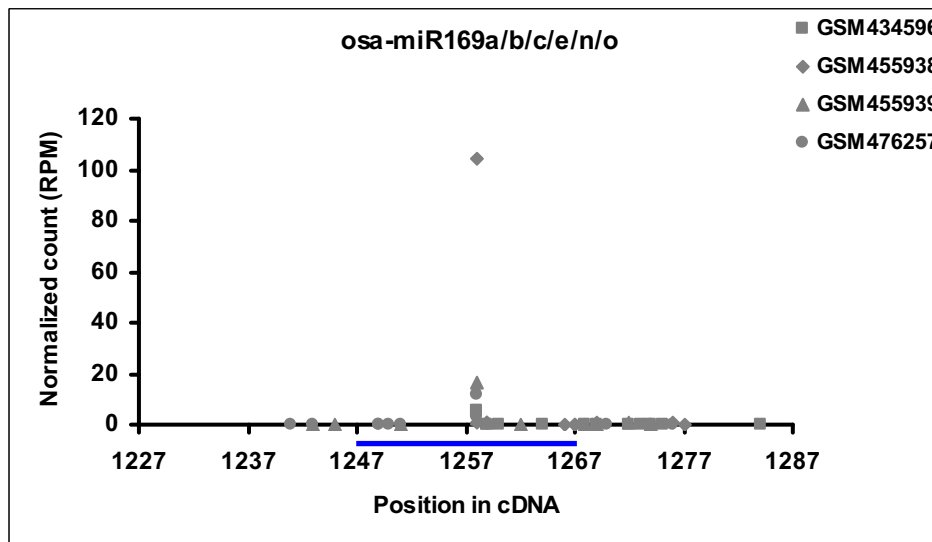

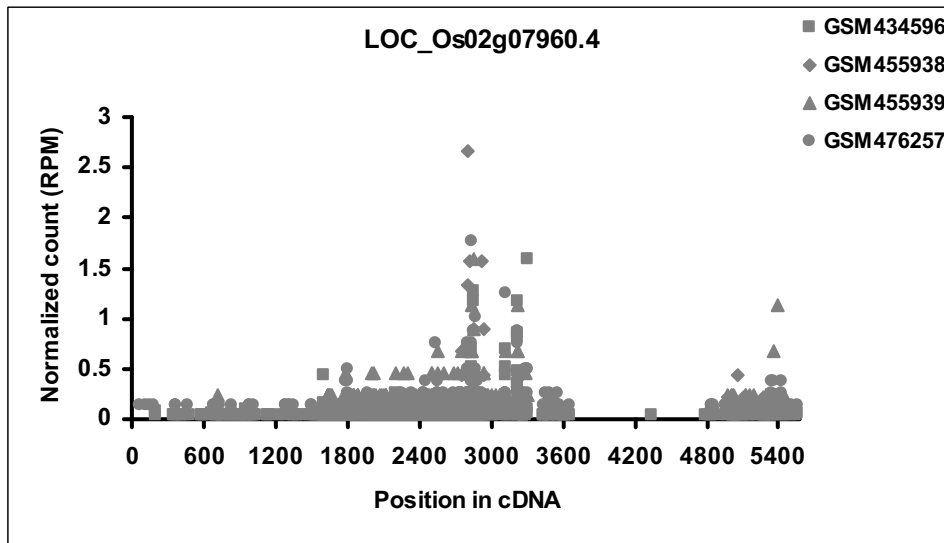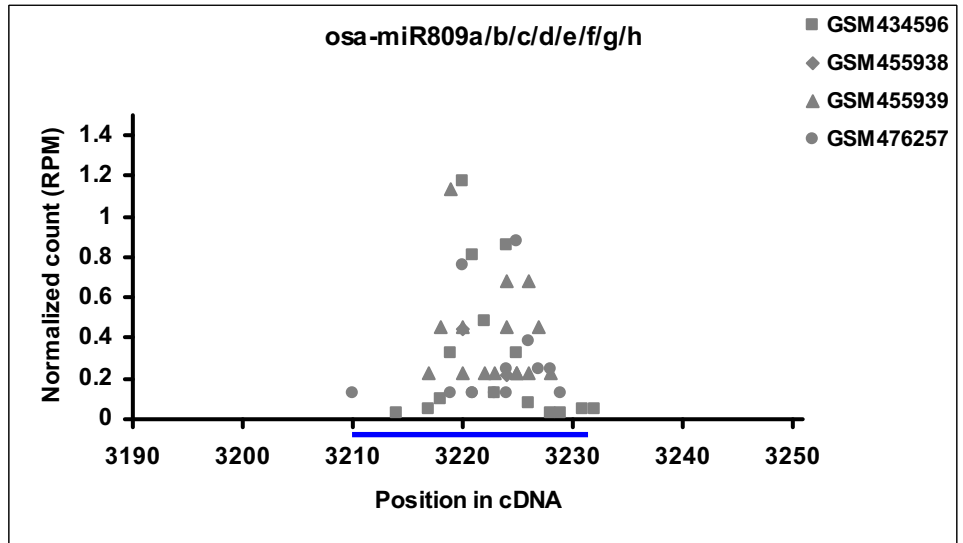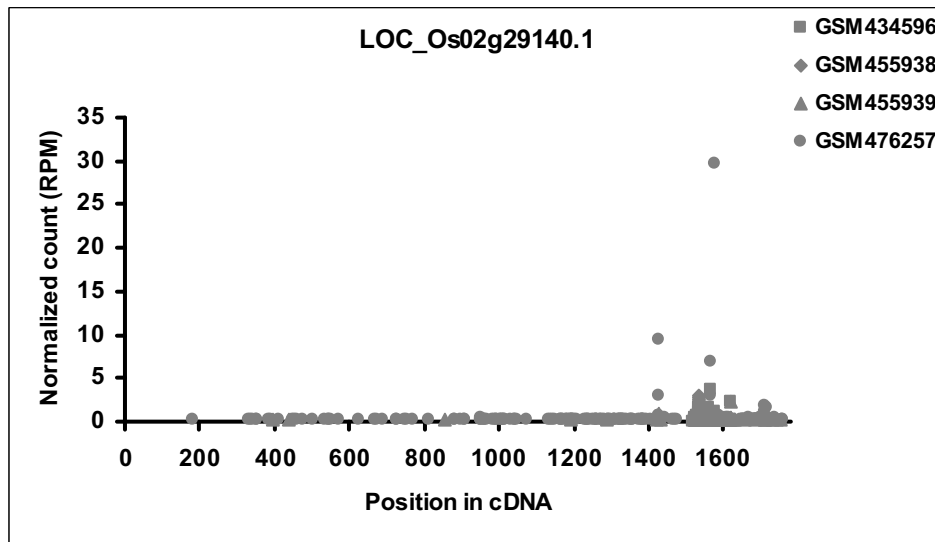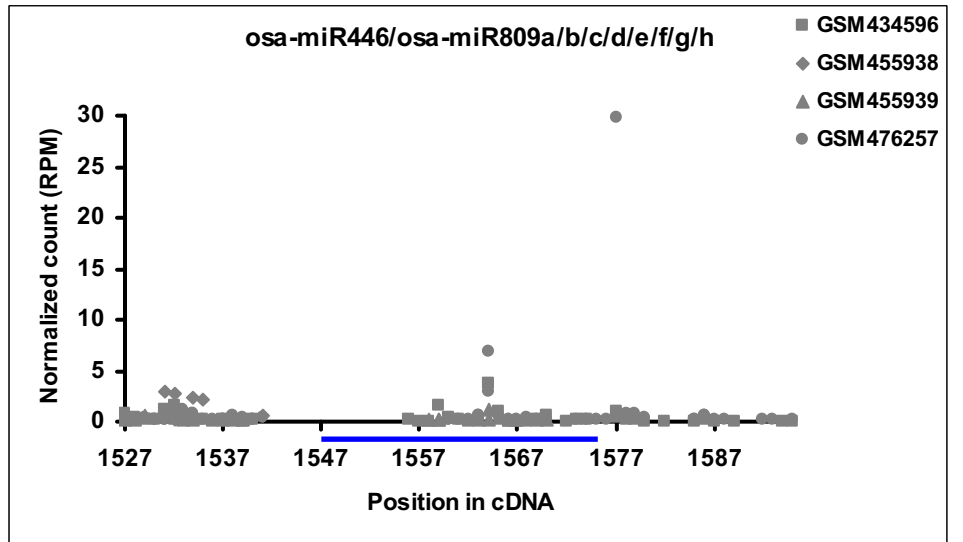

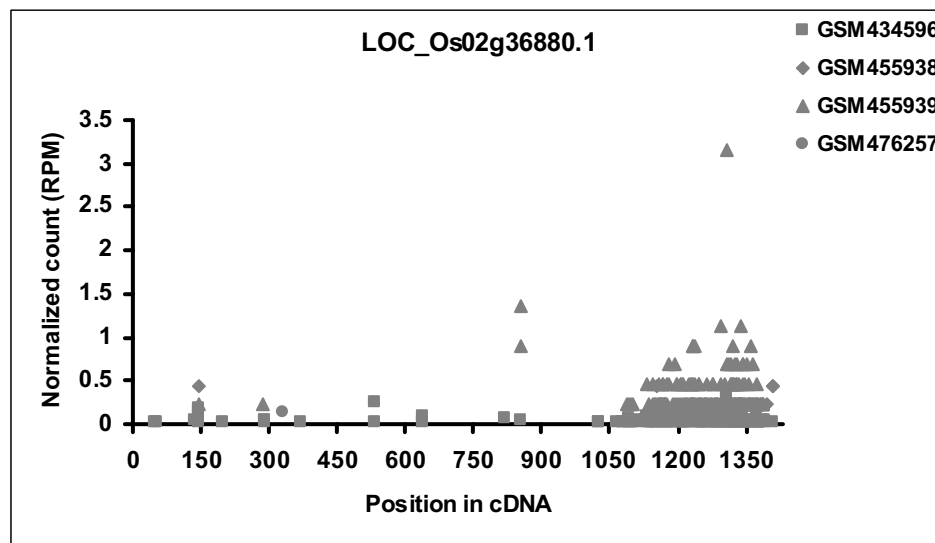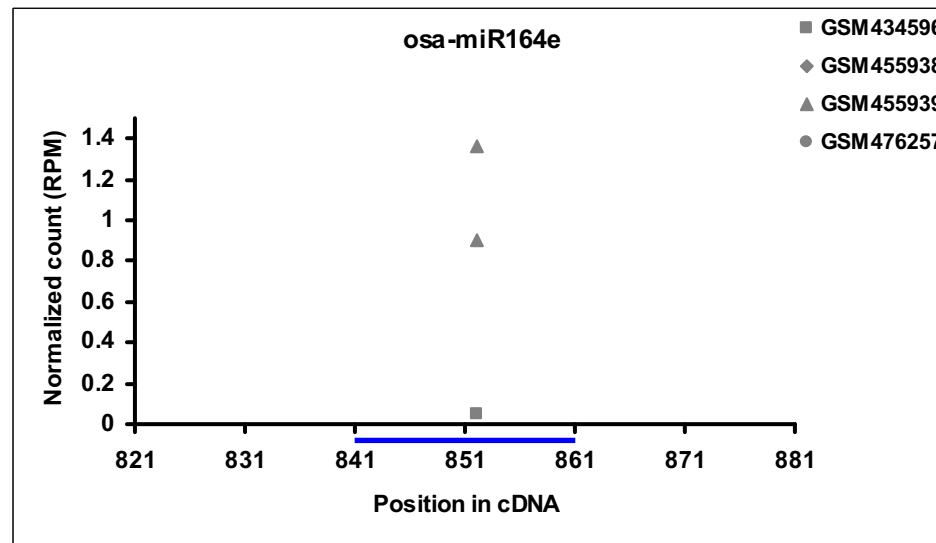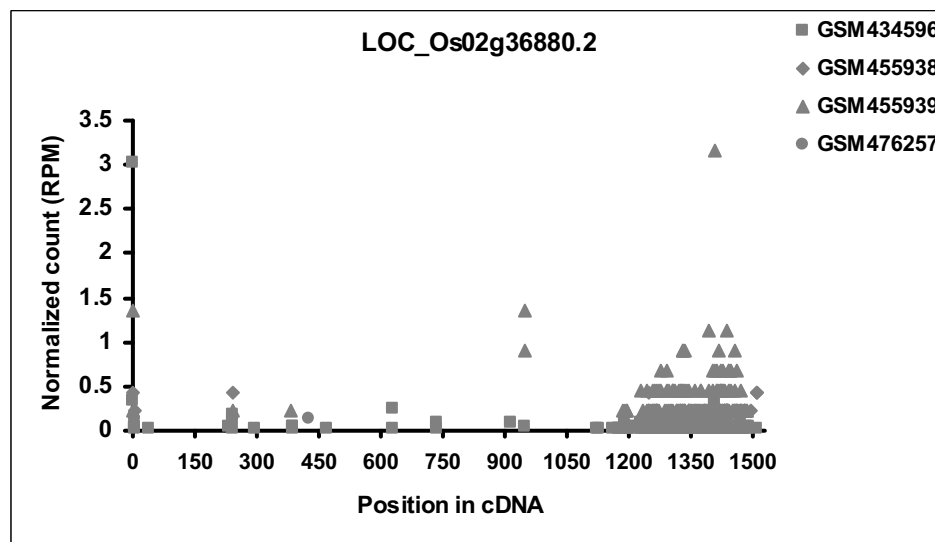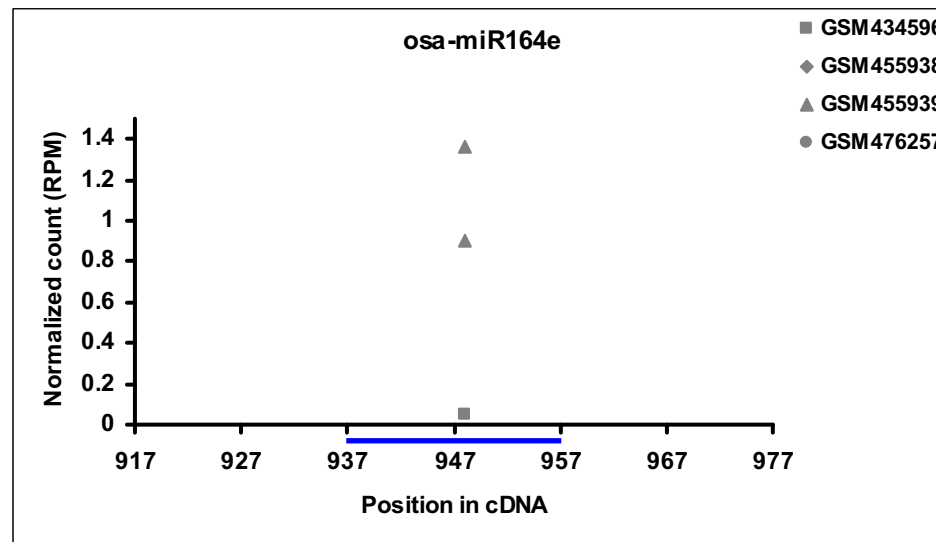

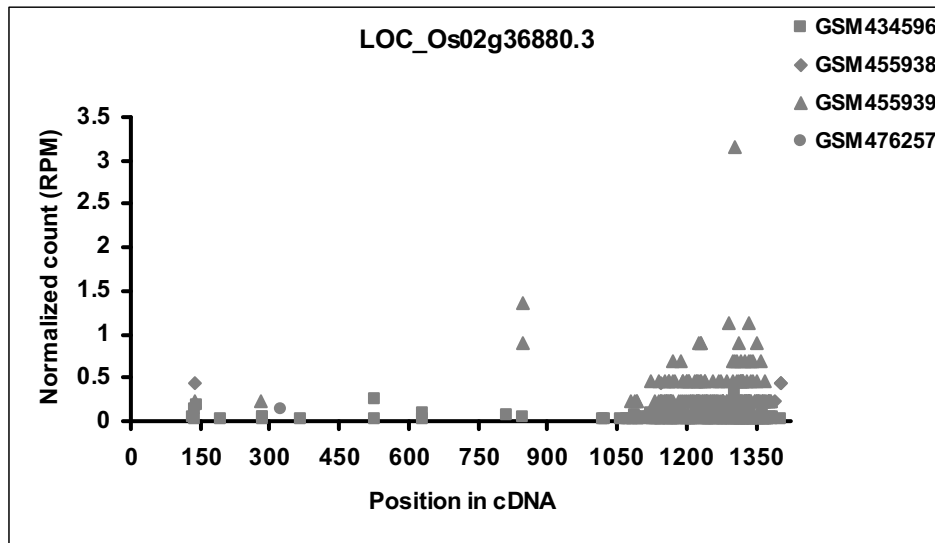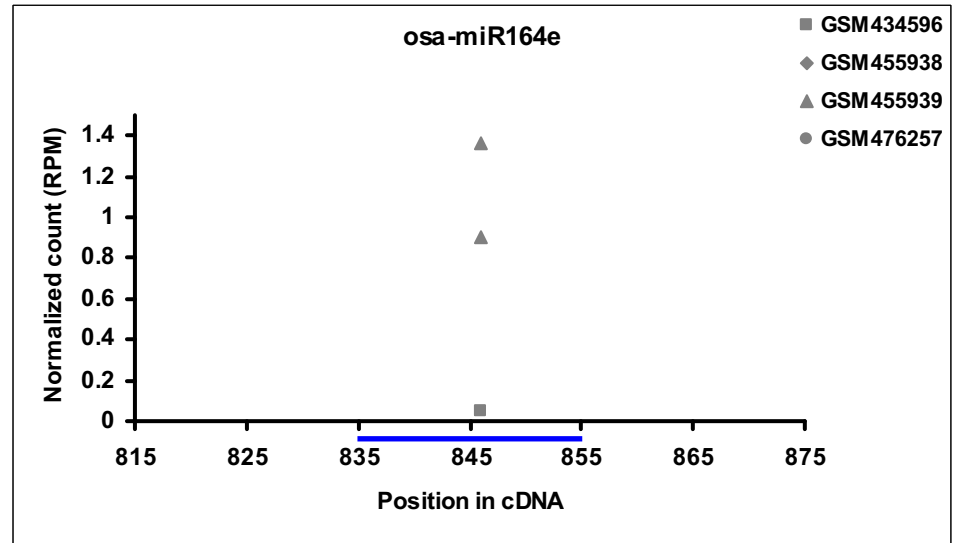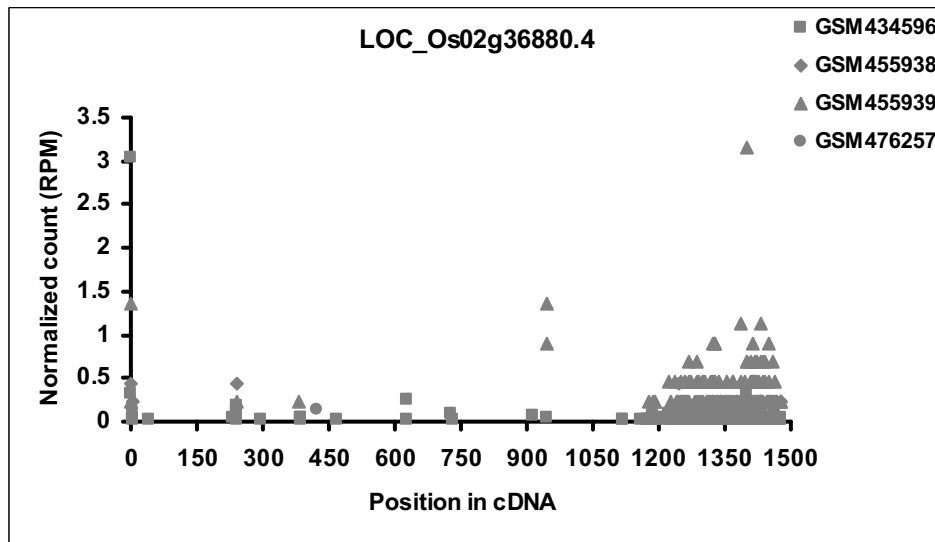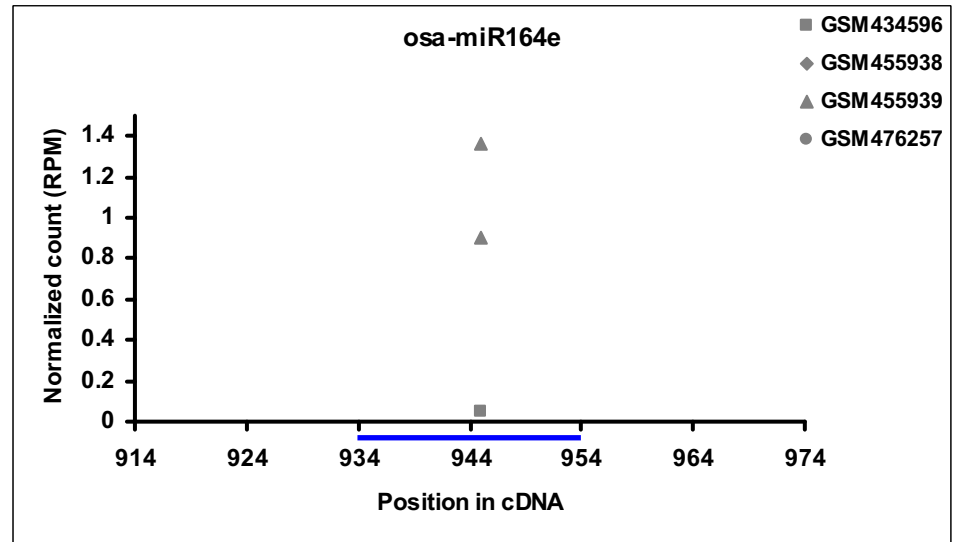

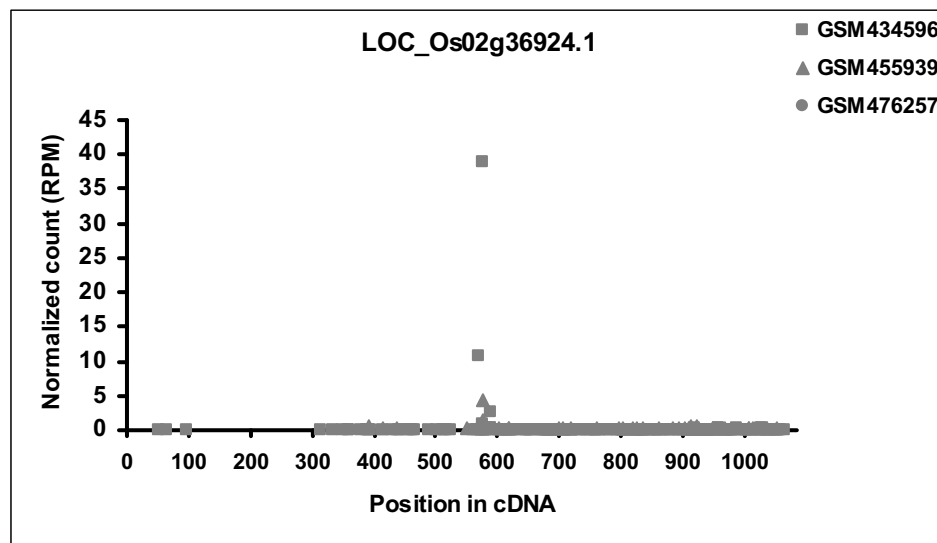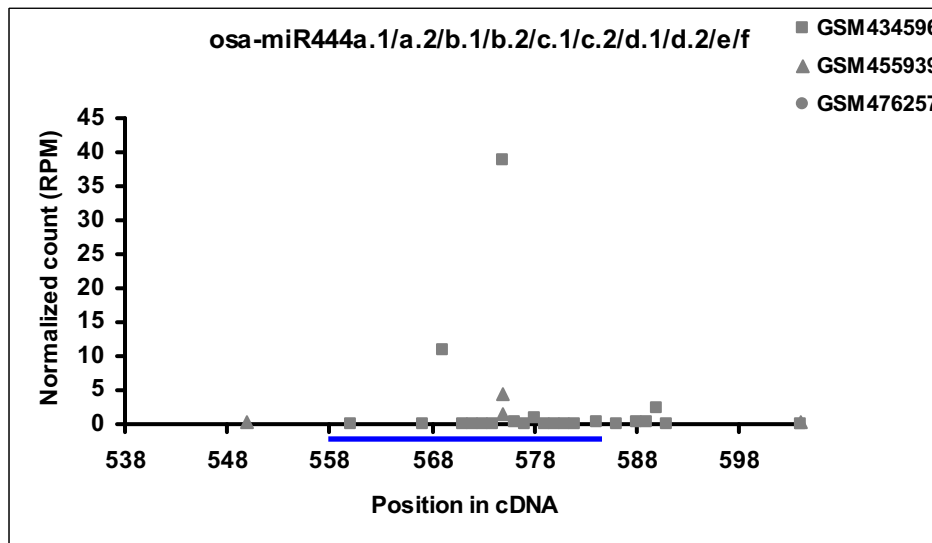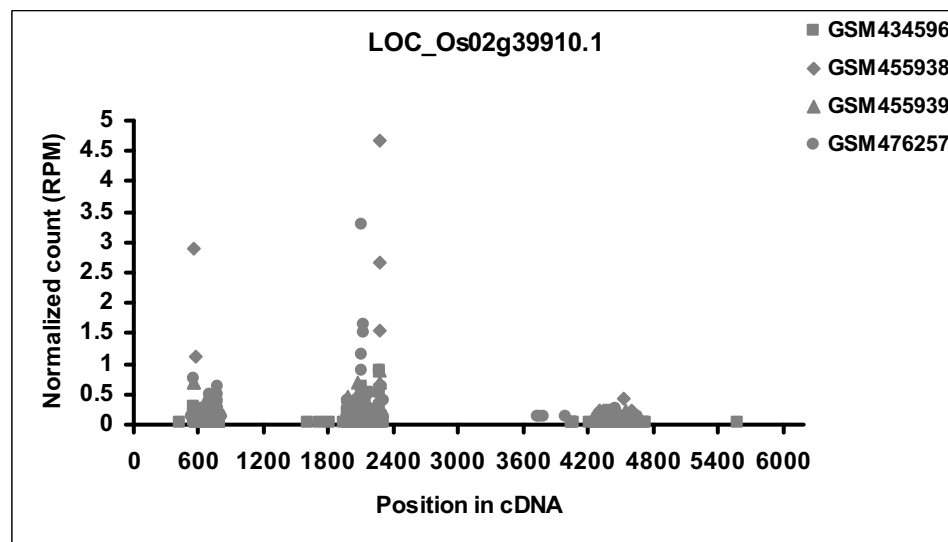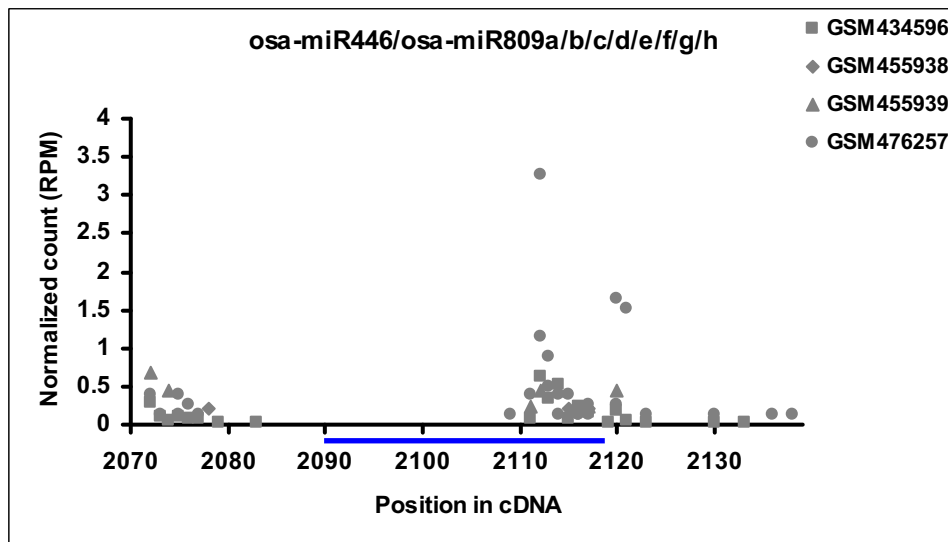

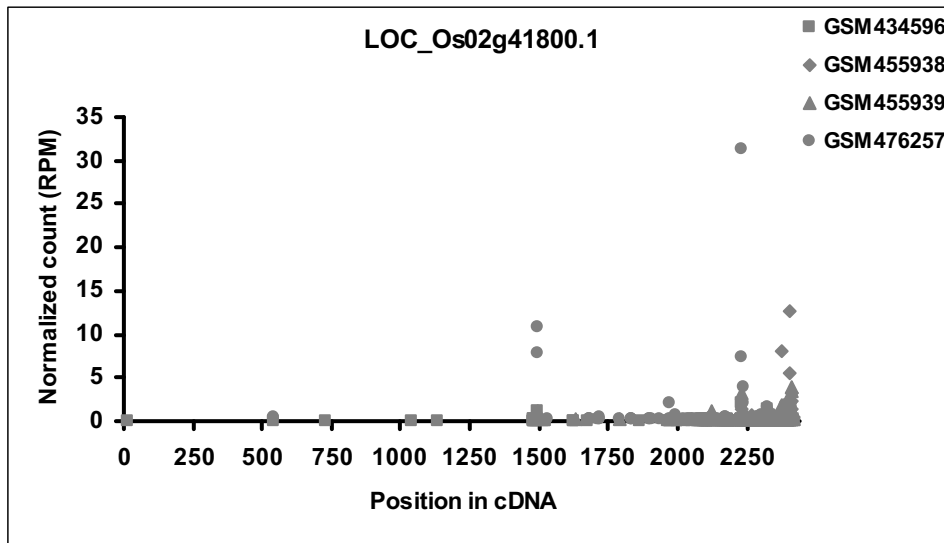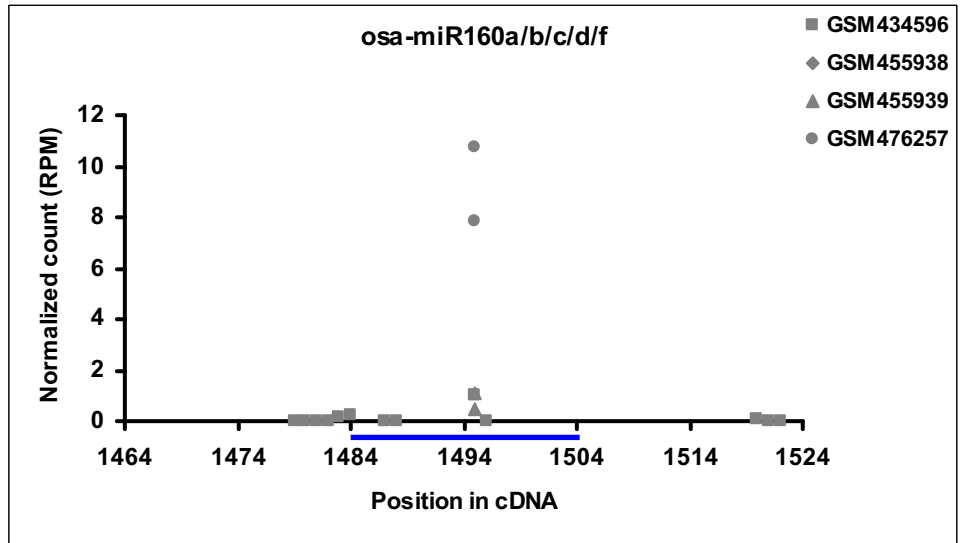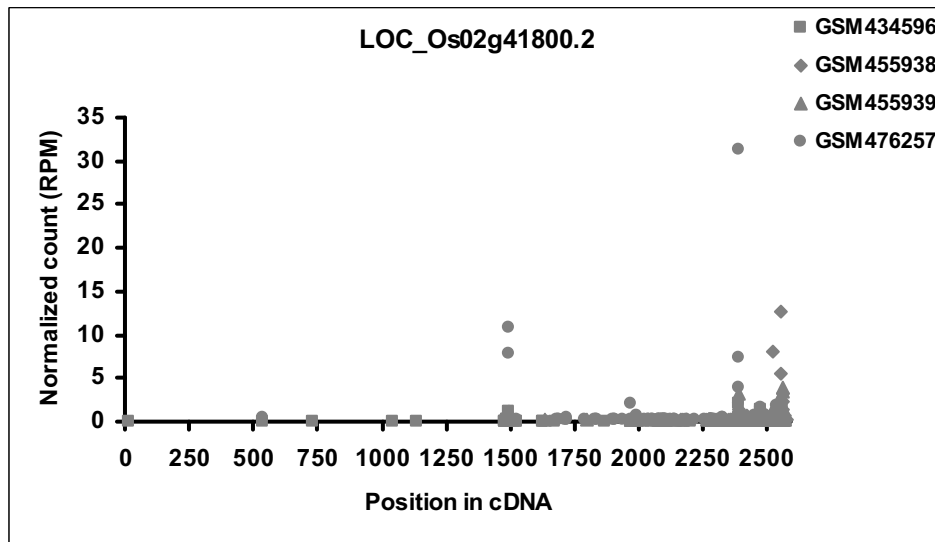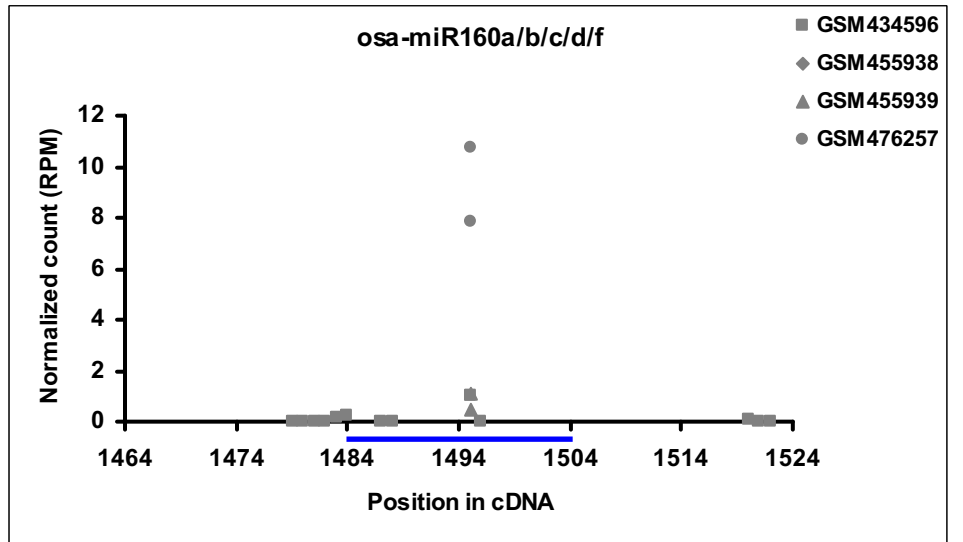

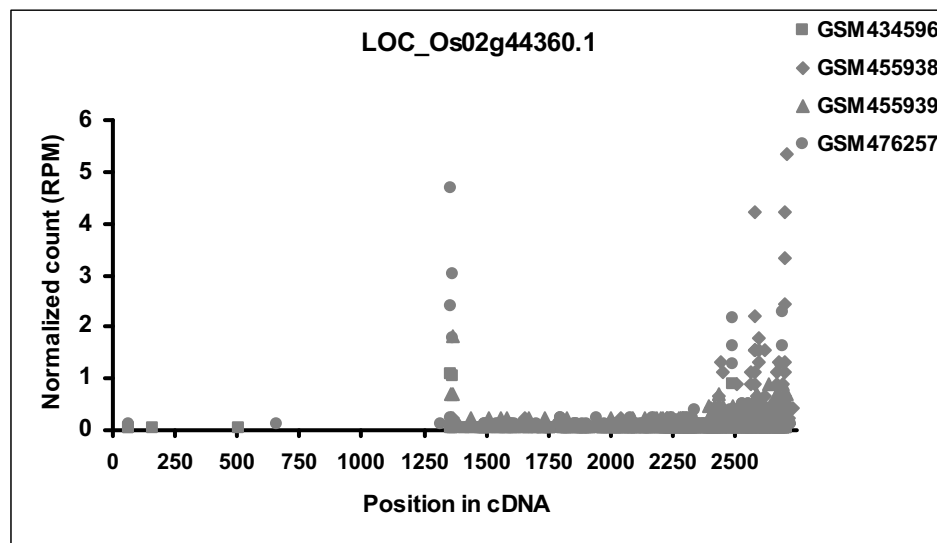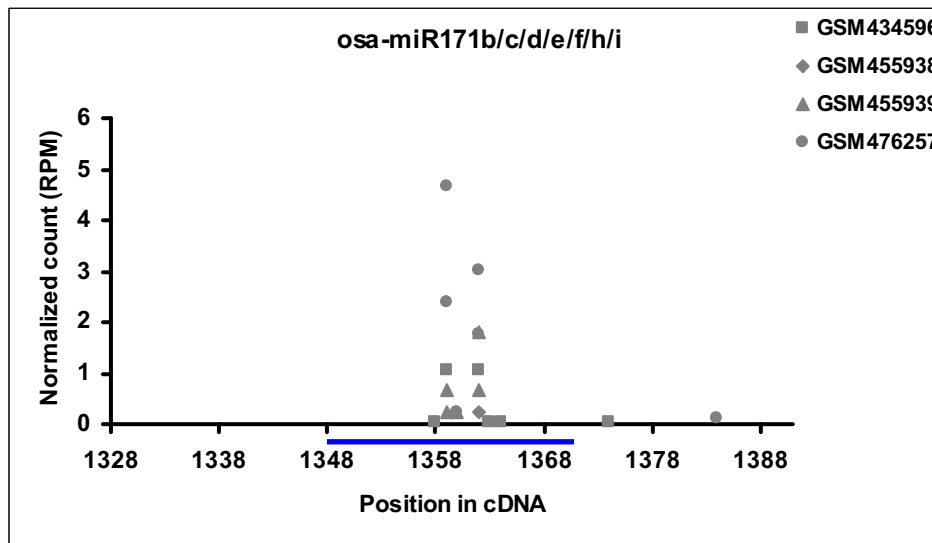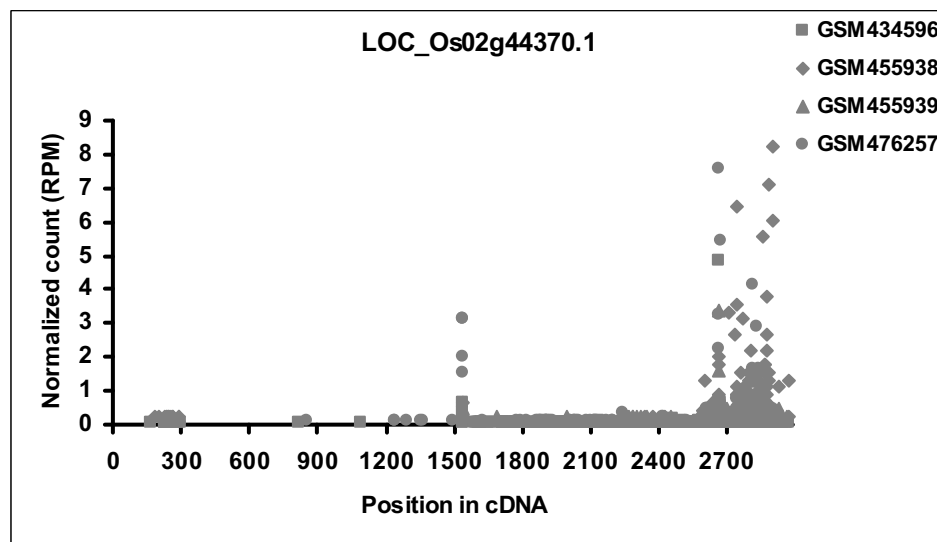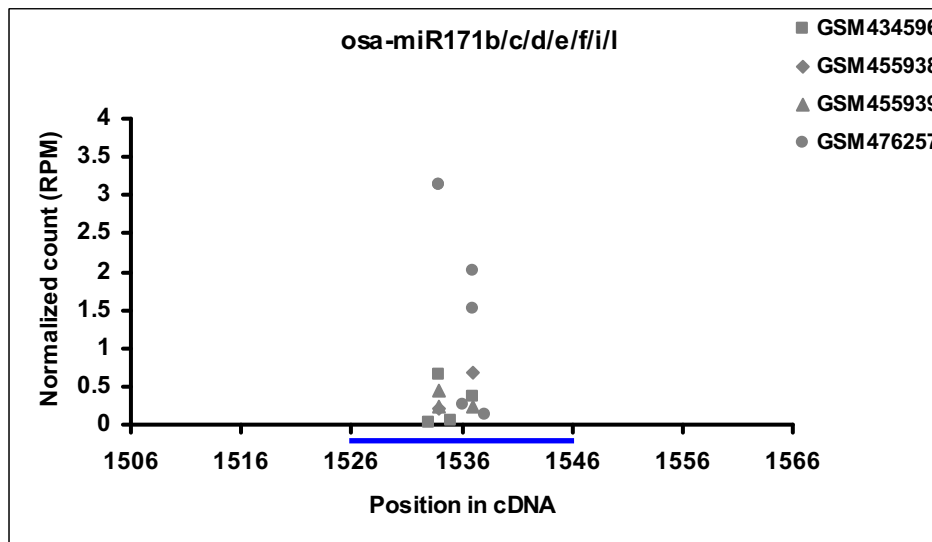

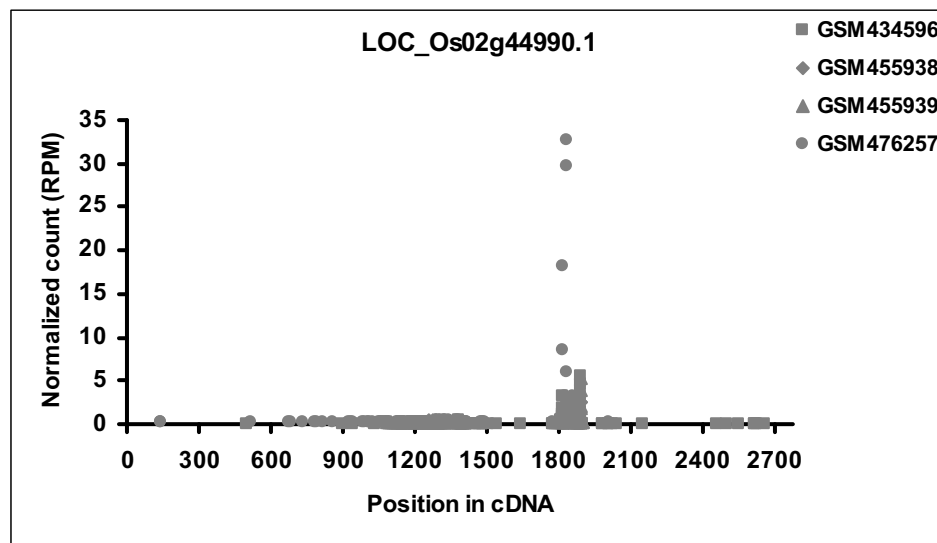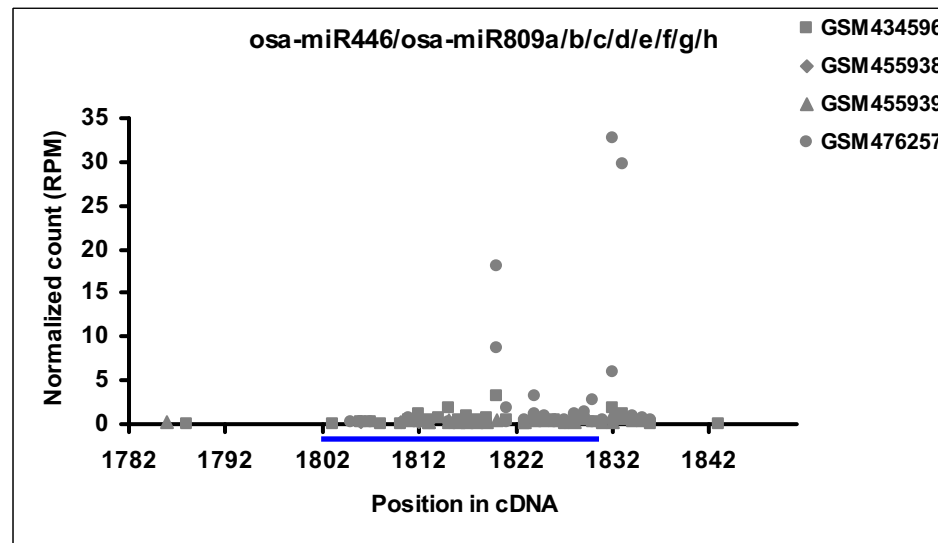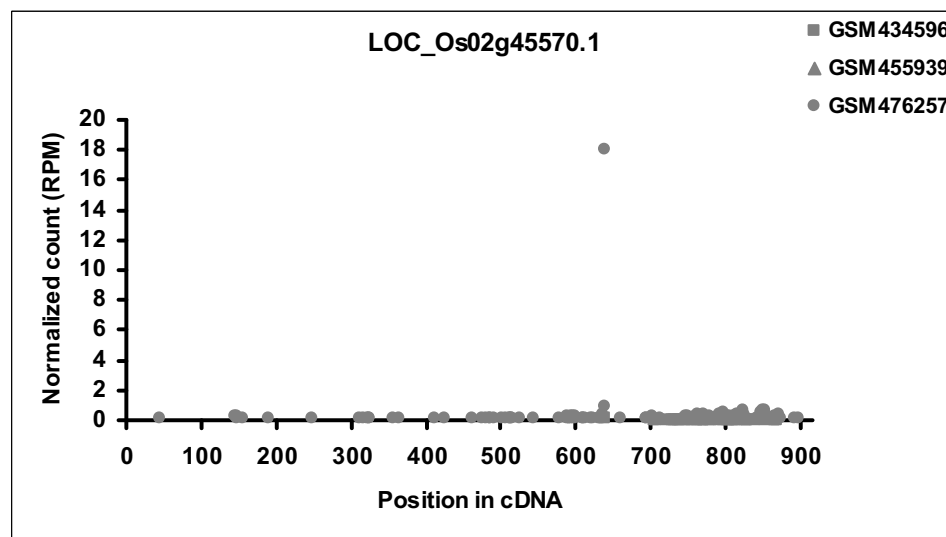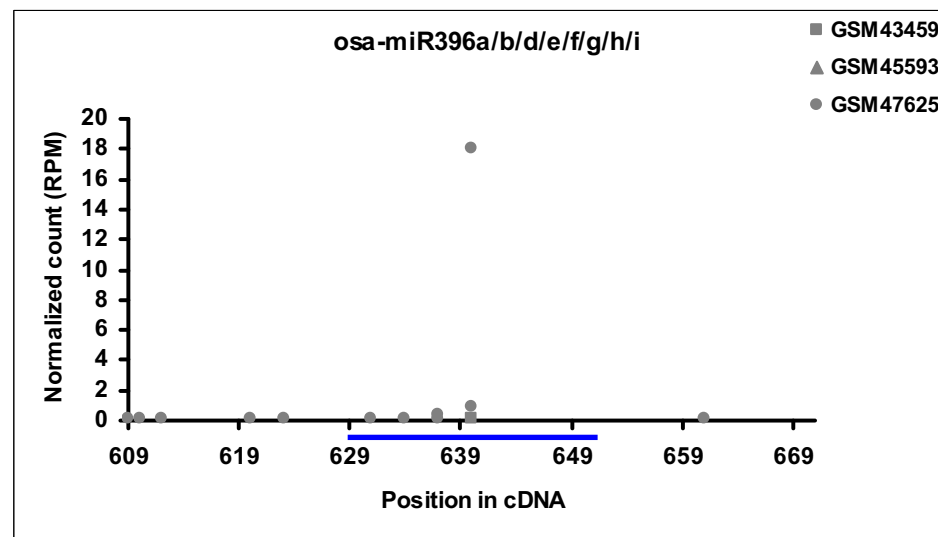

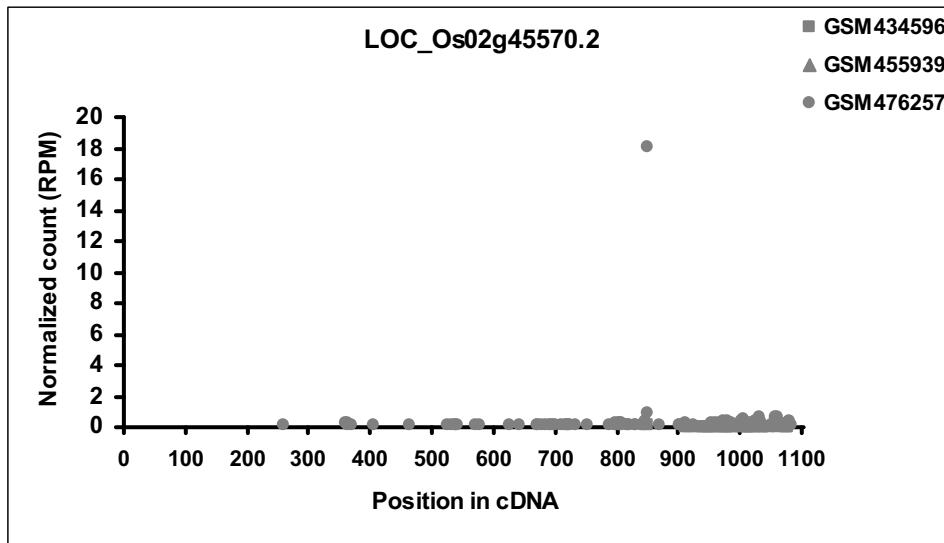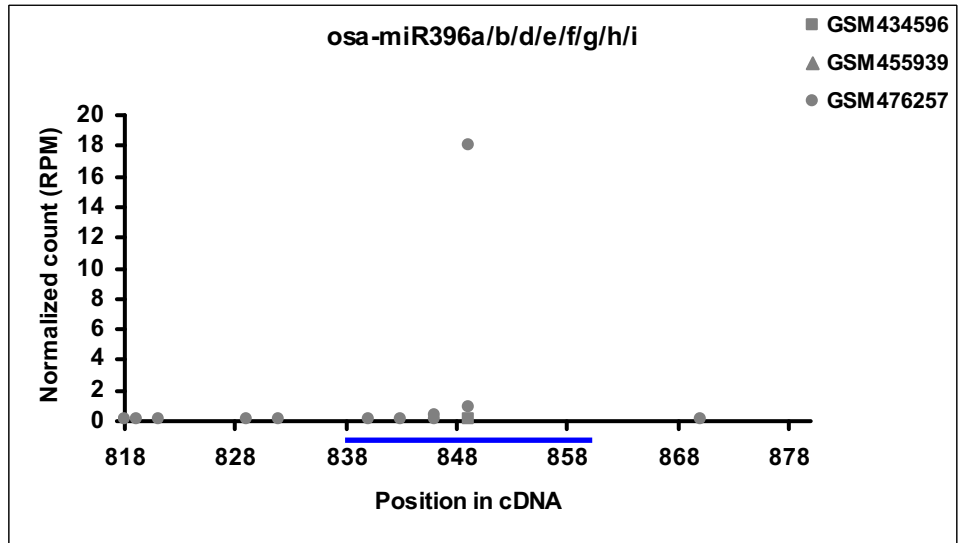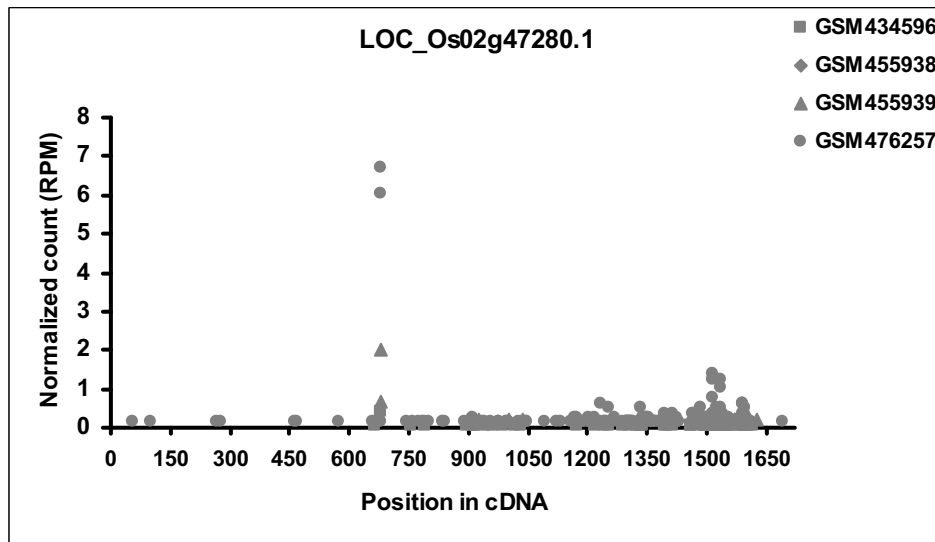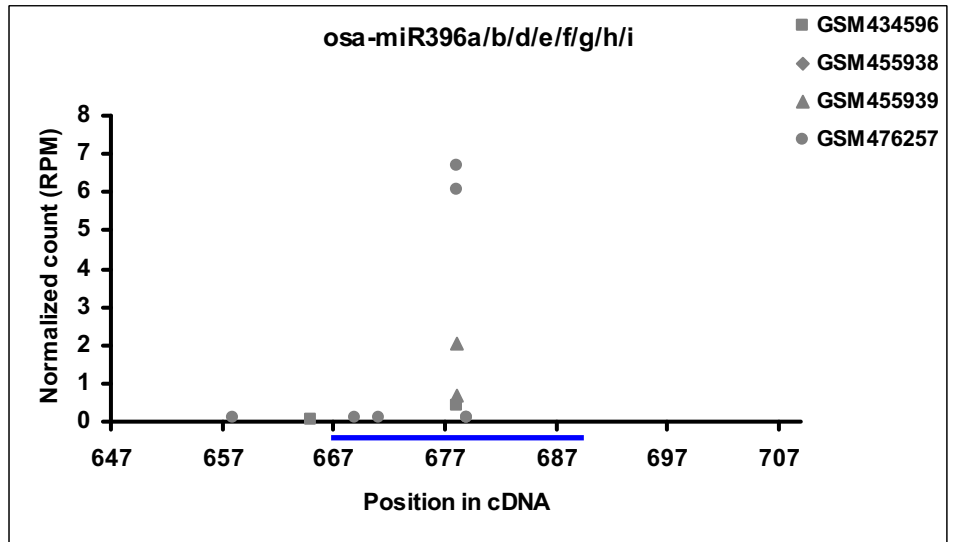

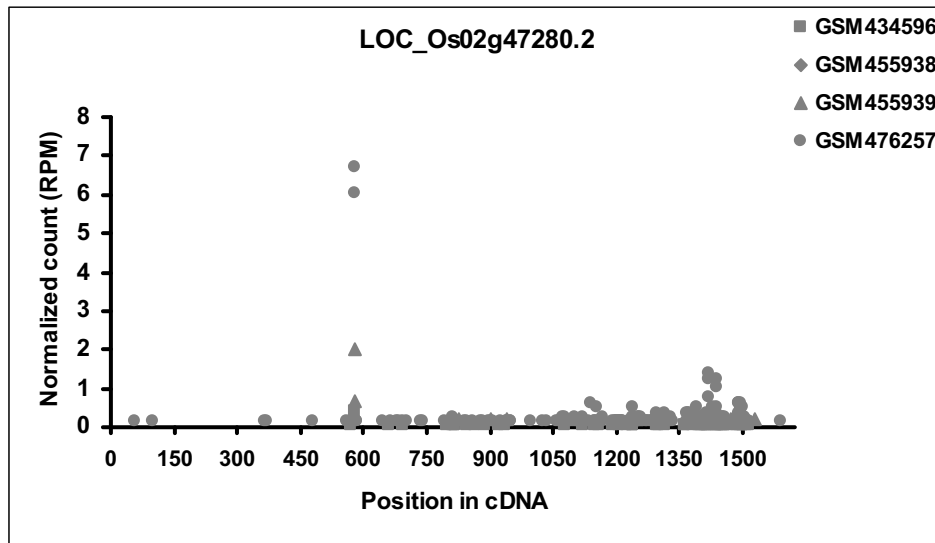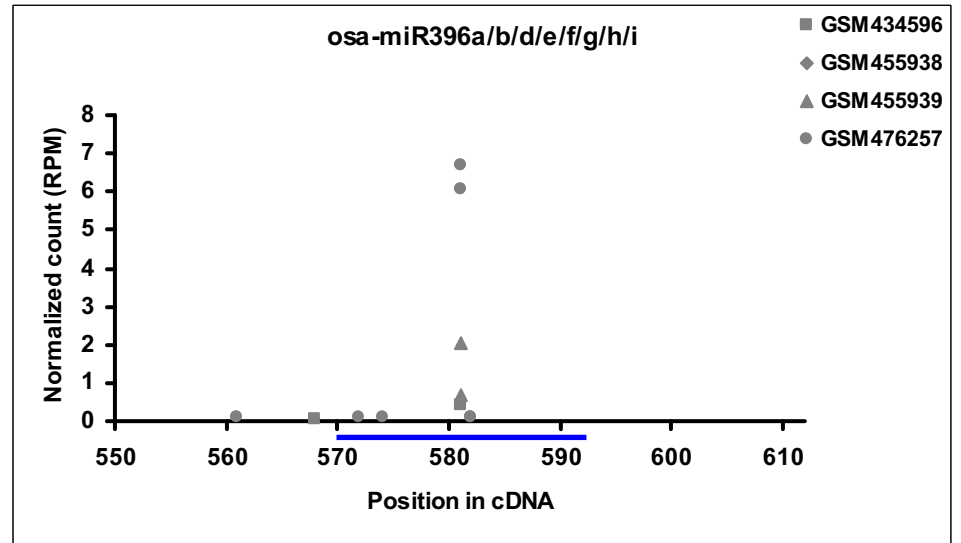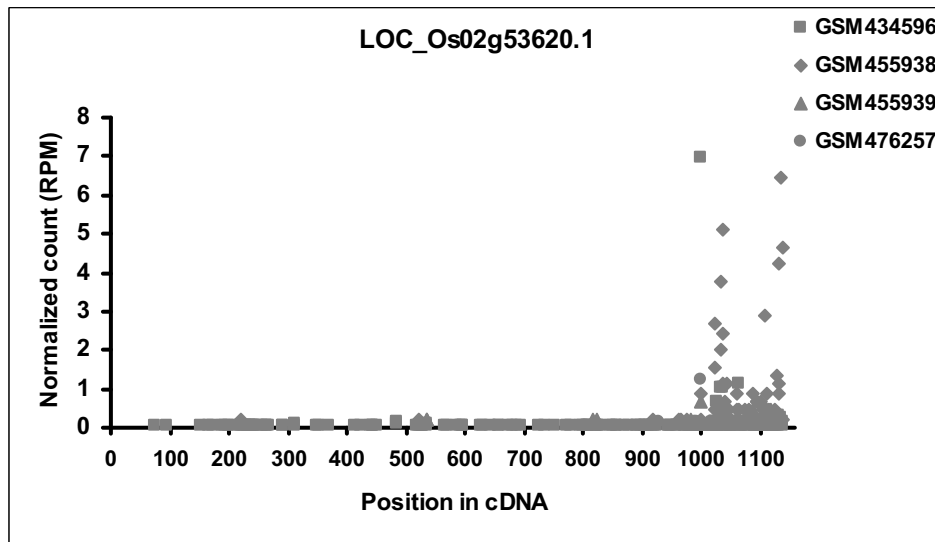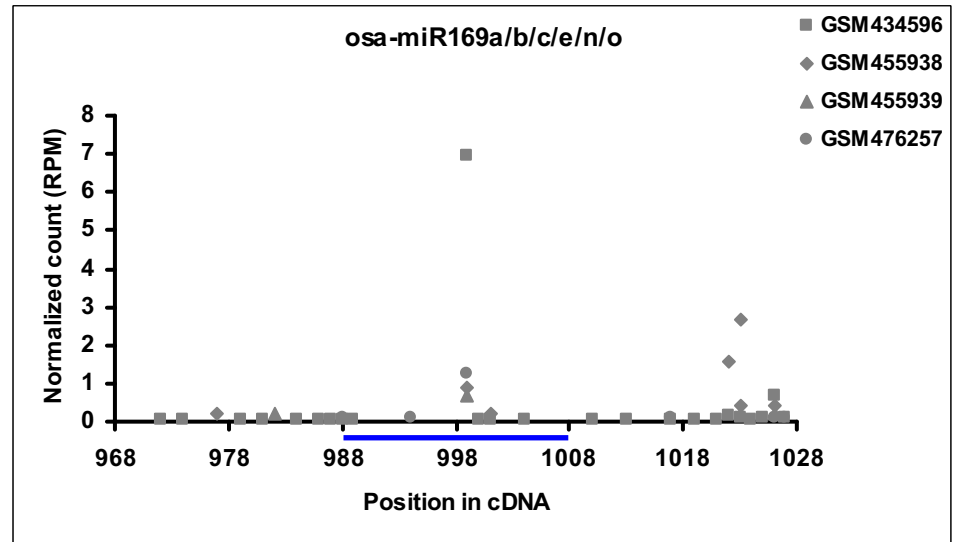

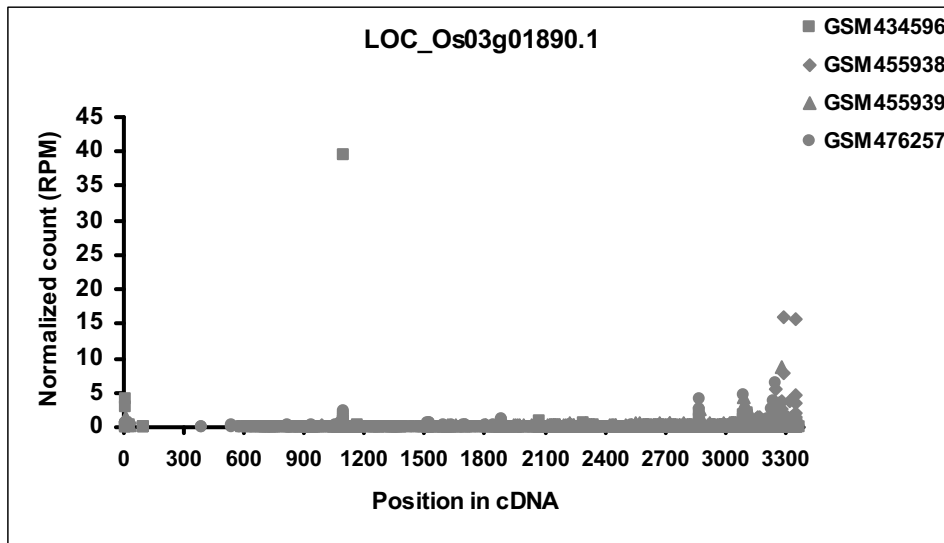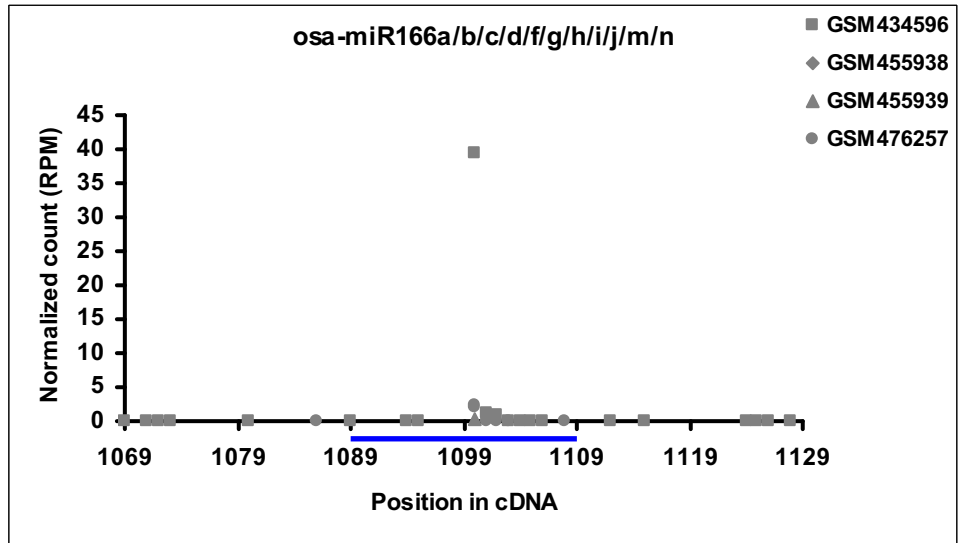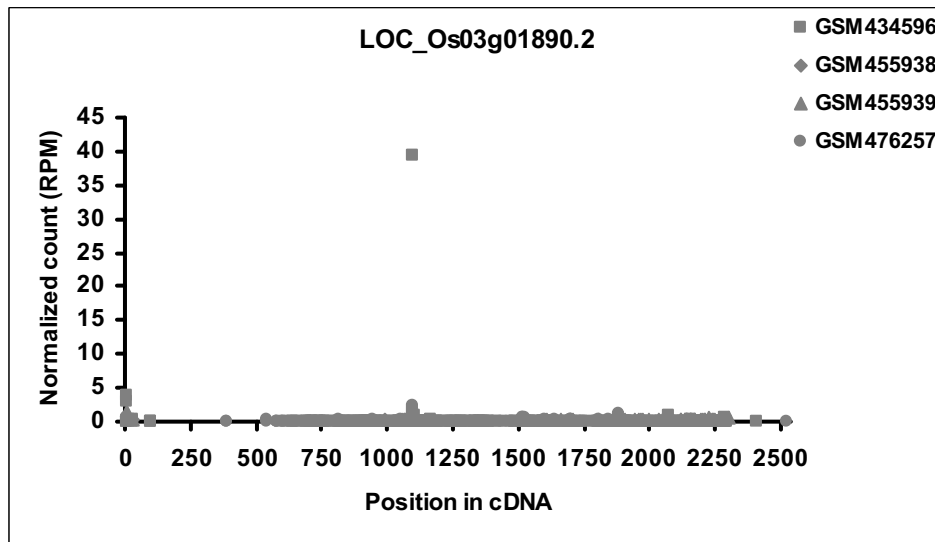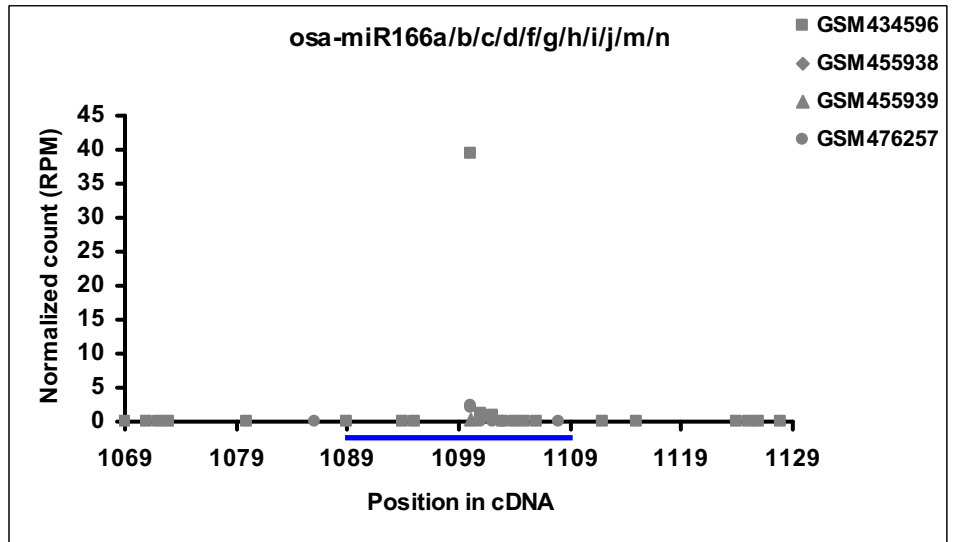

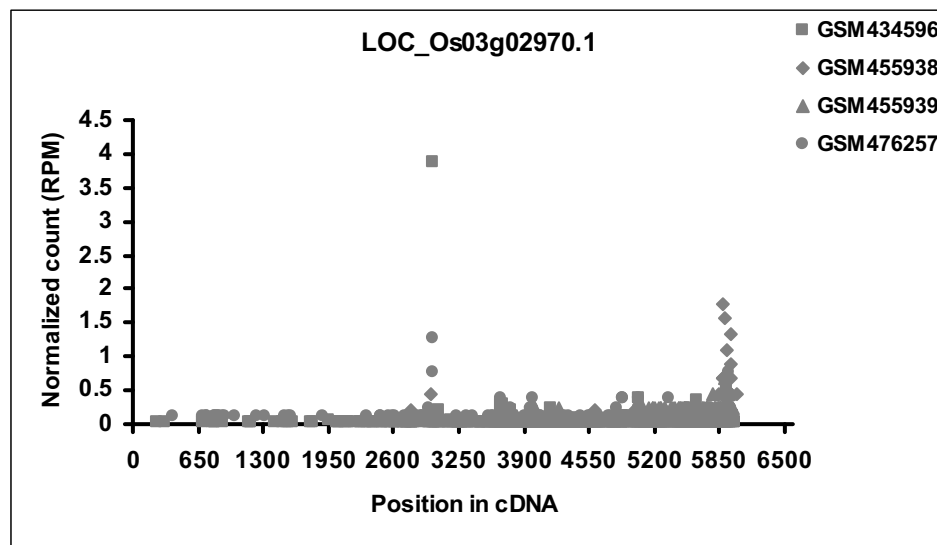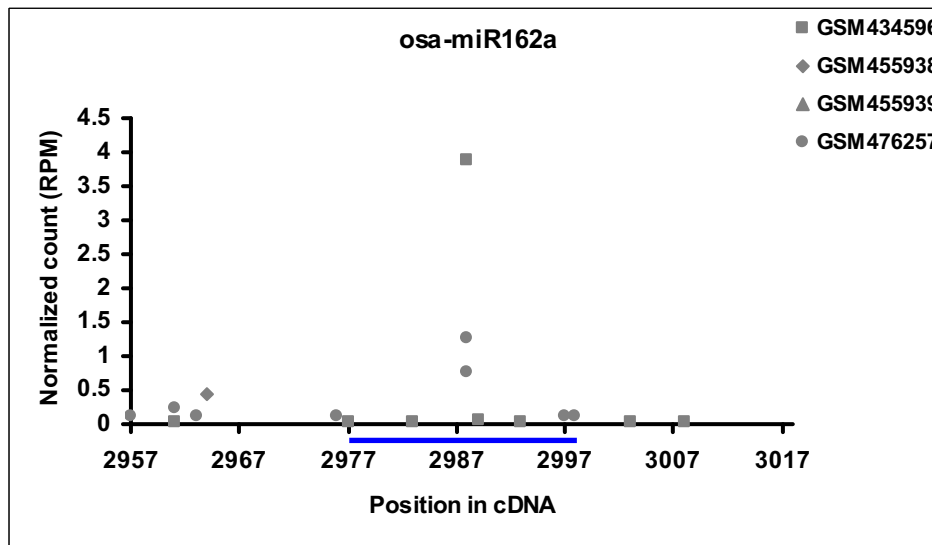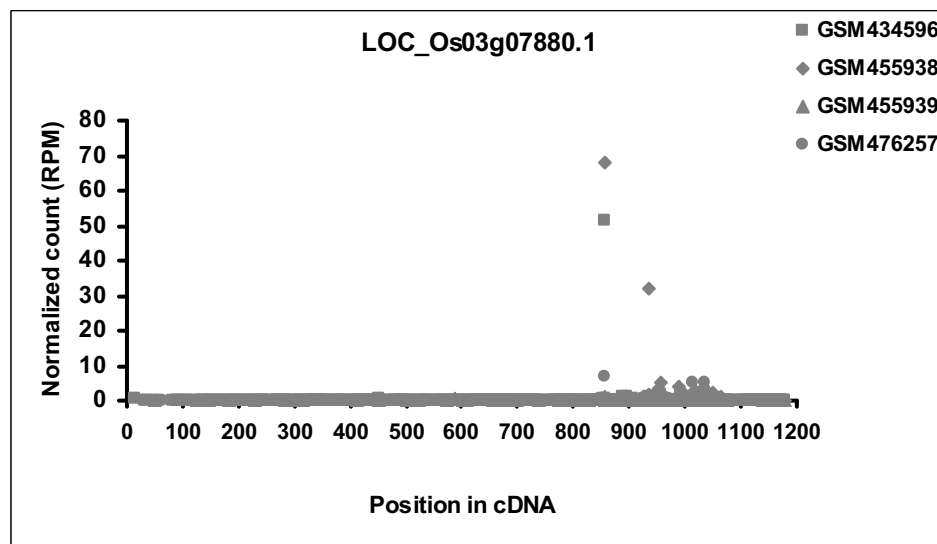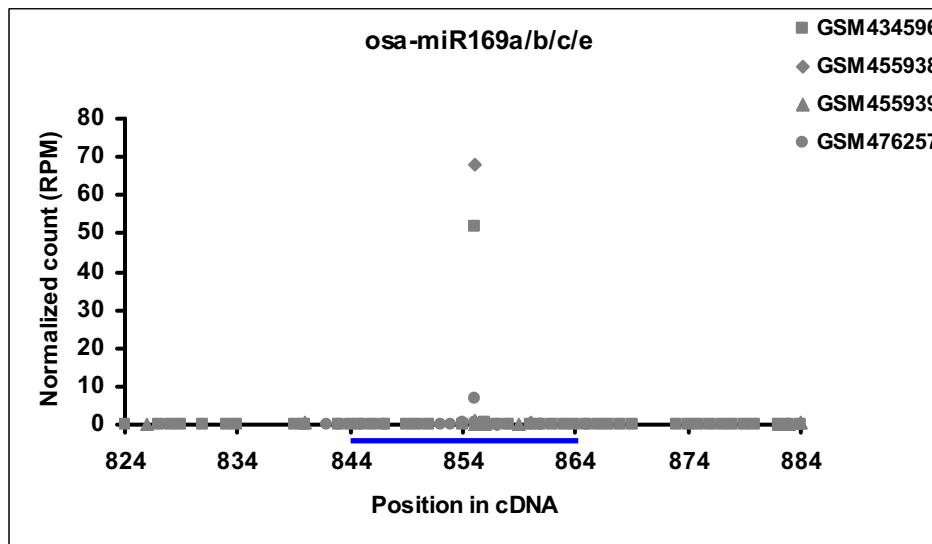

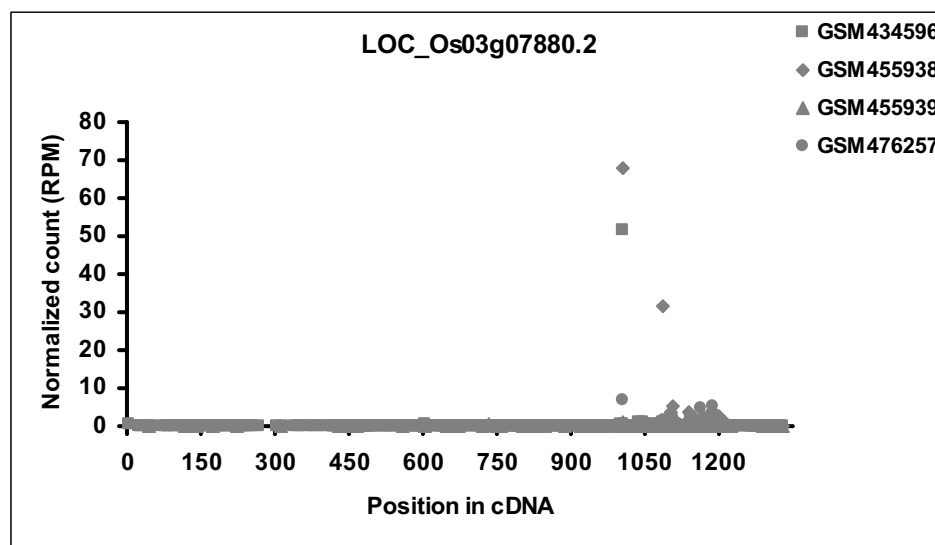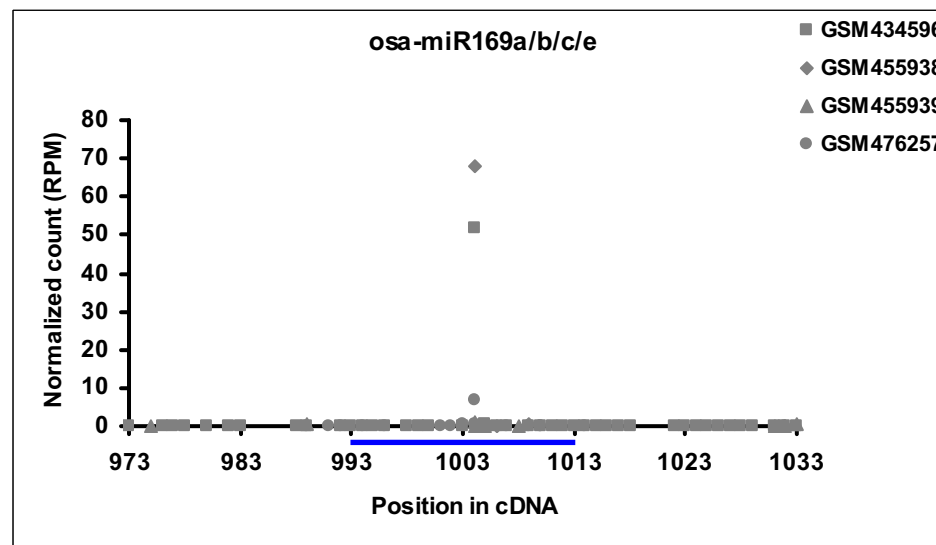

Supplement: Additional file 5 — Figure S2. Degradome sequencing data-based identification of the targets of sequestered microRNAs in rice. [file 1471-2164-13-197-S5.pdf]

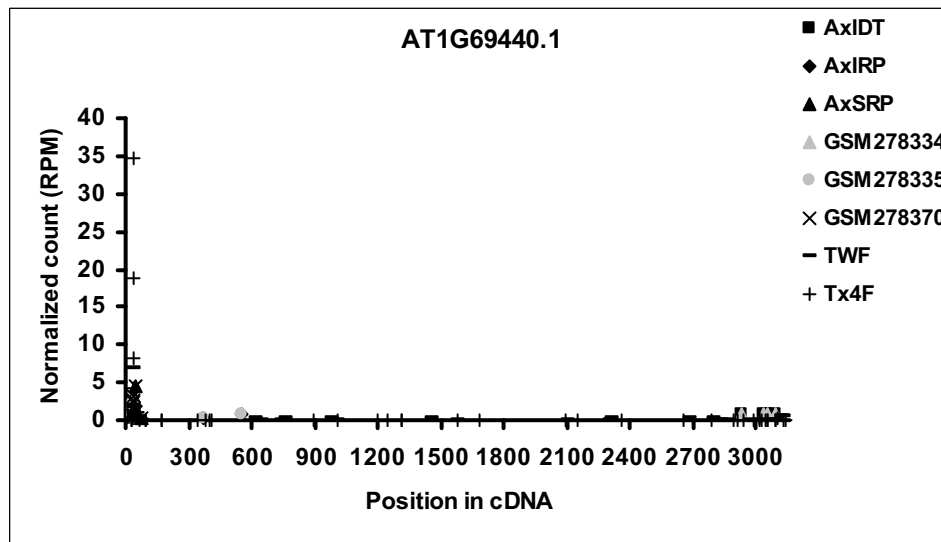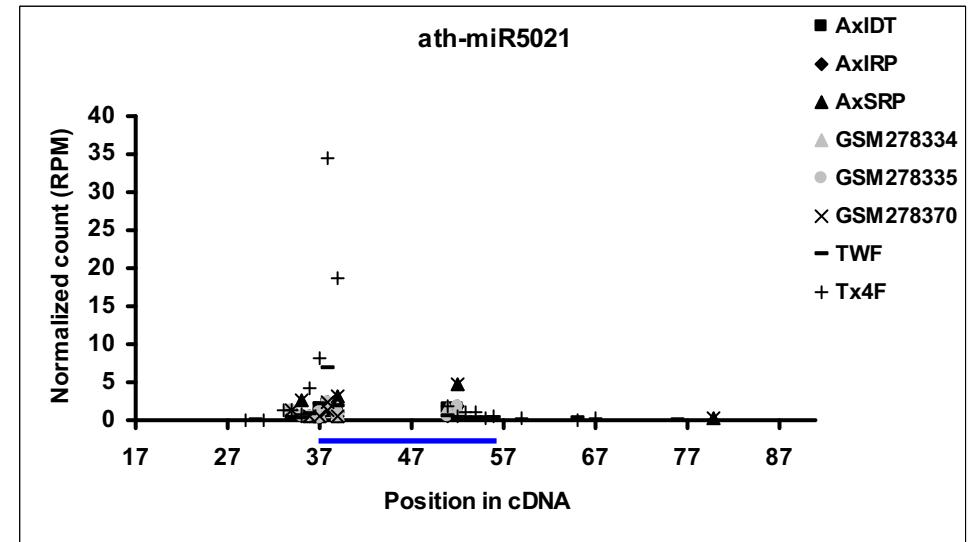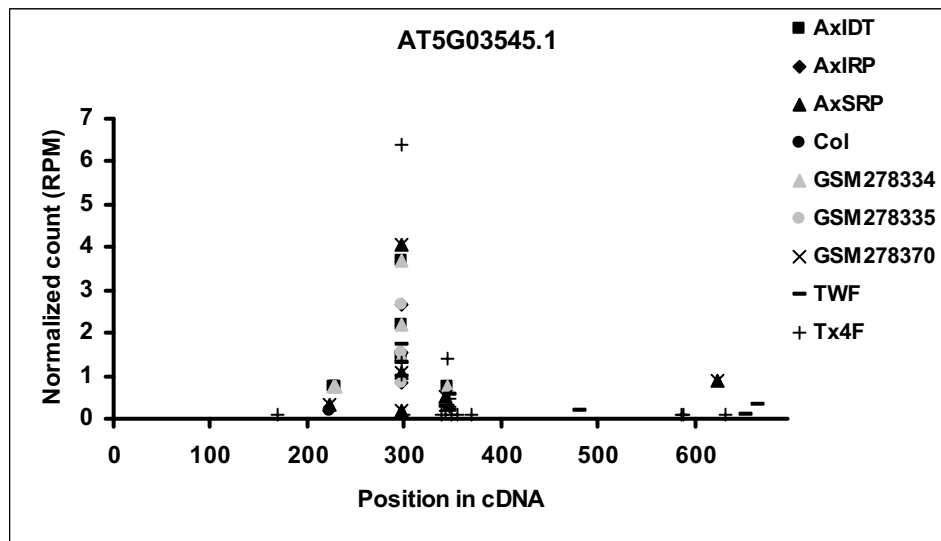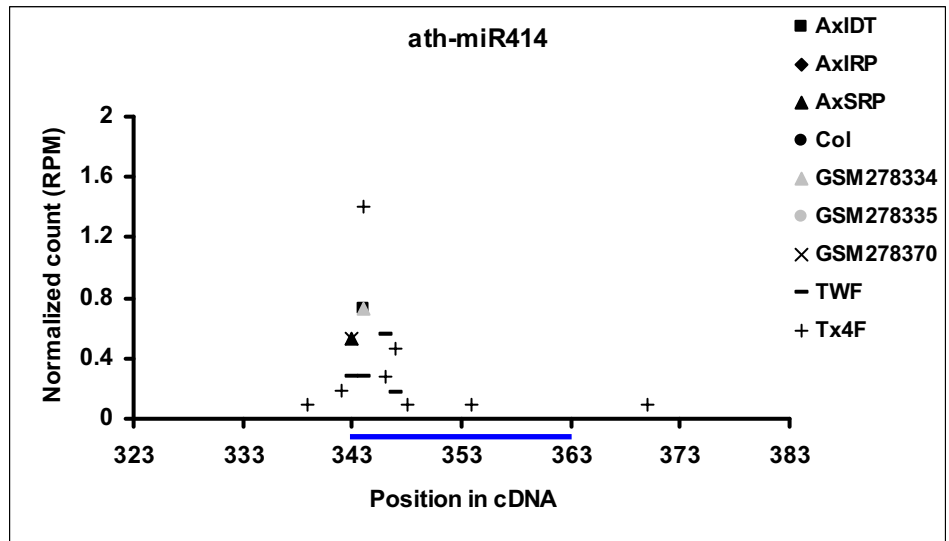

Supplement: Additional file 7 — Figure S4. Degradome sequencing data-based identification of the microRNAs regulating the target mimics in Arabidopsis. [file 1471-2164-13-197-S7.pdf]

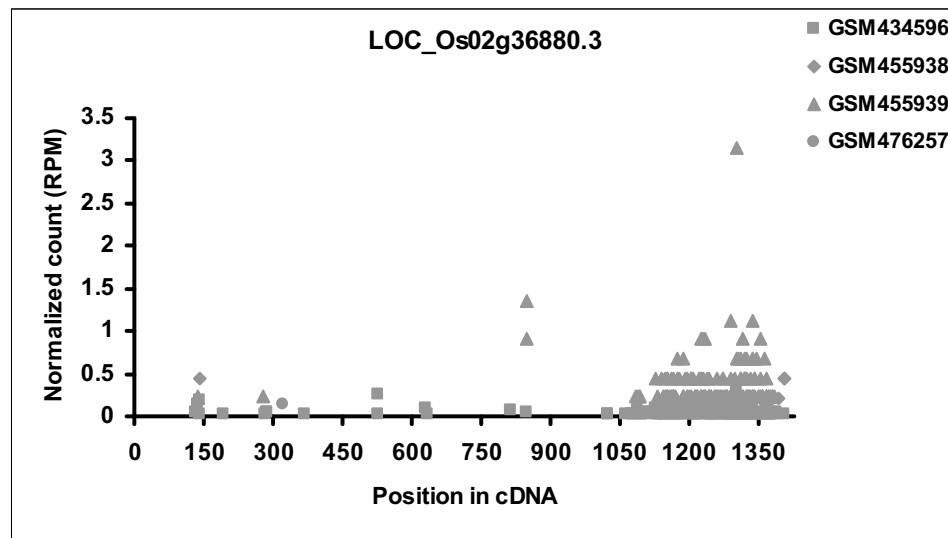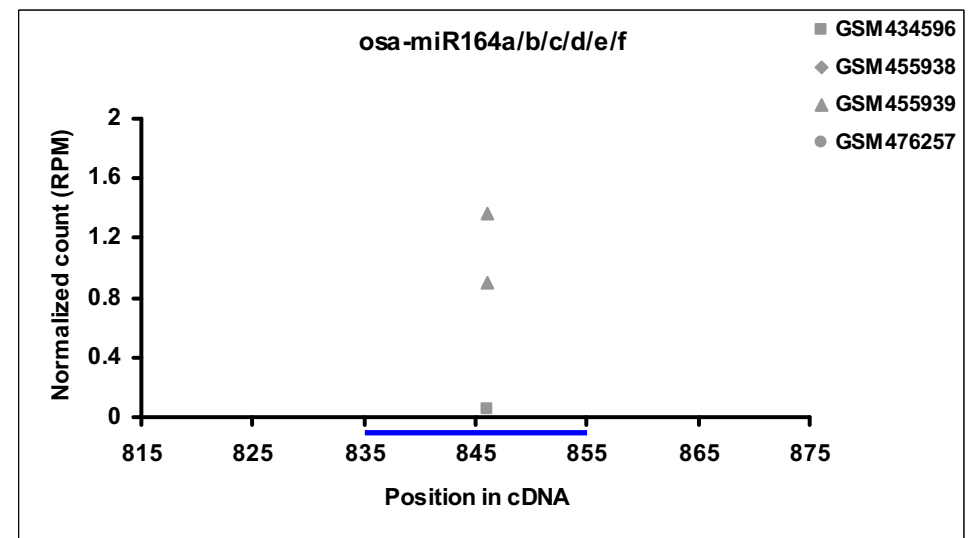

Supplement: Additional file 8 — Figure S5. Degradome sequencing data-based identification of the microRNAs regulating the target mimics in rice. [file 1471-2164-13-197-S8.pdf]

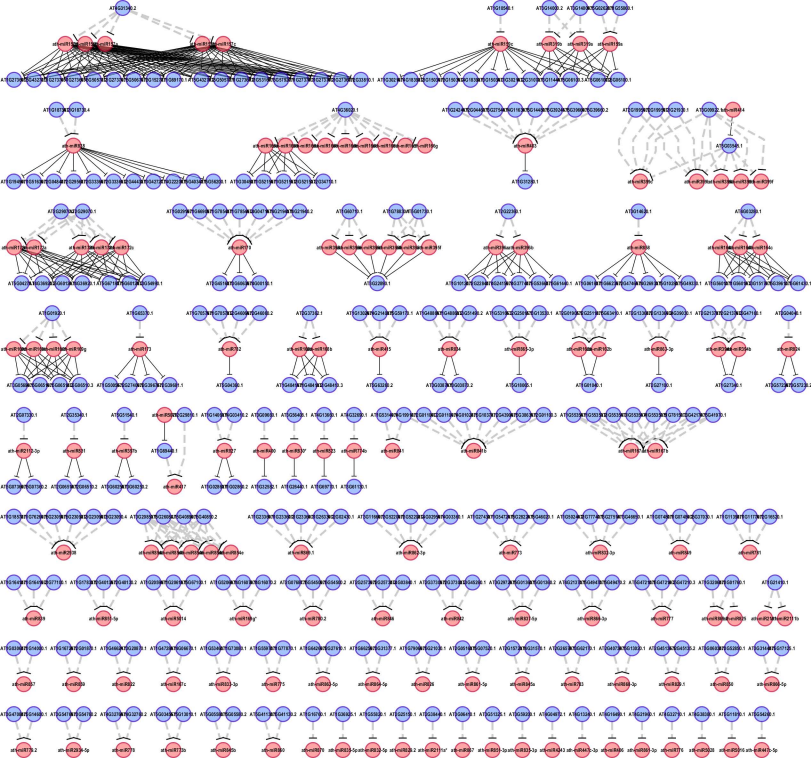

Supplement: Additional file 9 — Figure S6. The whole network constructed in Arabidopsis. [file 1471-2164-13-197-S9.pdf]

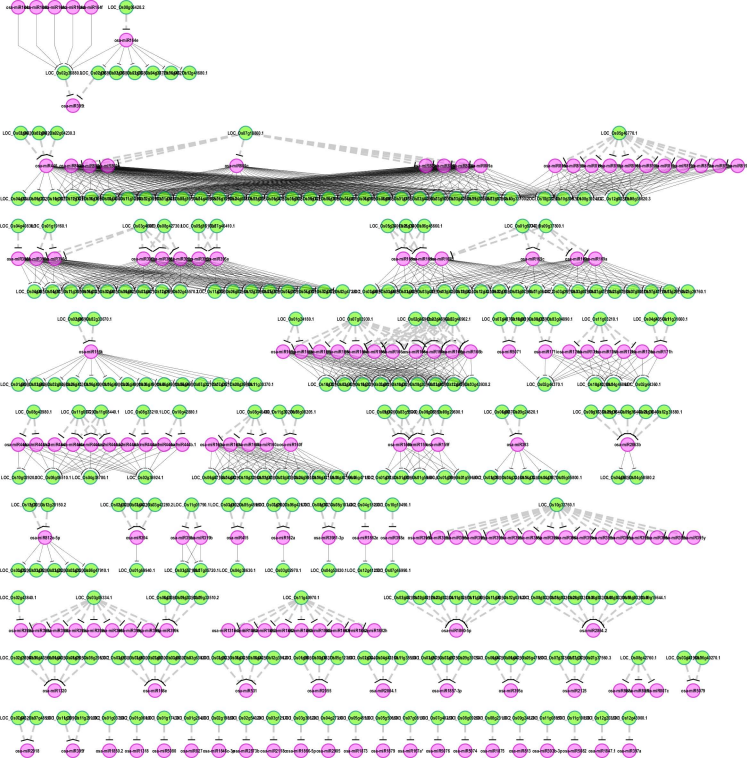

Supplement: Additional file 10 — Figure S7. The whole network constructed in rice. [file 1471-2164-13-197-S10.pdf]

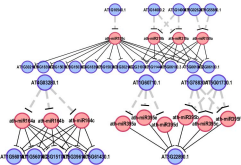

Supplement: Additional file 12 — Figure S8. Ath-miR159/319-, ath-miR164-, and ath-miR395-involved subnetworks. [file 1471-2164-13-197-S12.pdf]

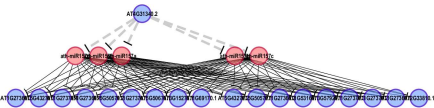

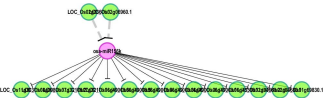

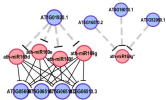

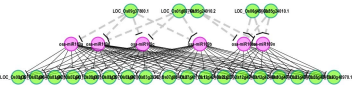

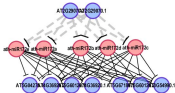

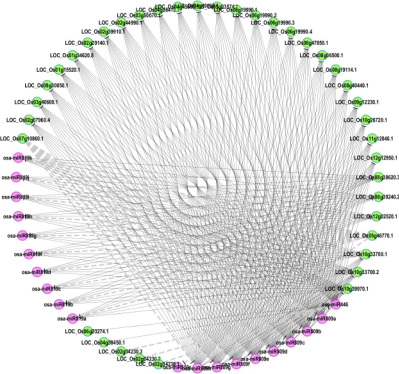

Supplement: Additional file 13 — Figure S9. Certain subnetworks in Arabidopsis and rice. [file 1471-2164-13-197-S13.pdf]
