# Supplementary material for: Target mimics: an embedded layer of microRNA-involved gene regulatory networks in plants
Source: BMC Genomics. 2012 May 21;13:197. doi: 10.1186/1471-2164-13-197 (PMC3441763; doi:10.1186/1471-2164-13-197)

(A) Arabidopsis_Molecular Function

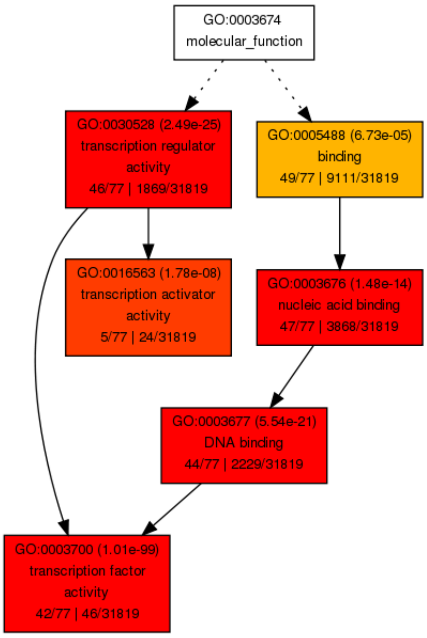

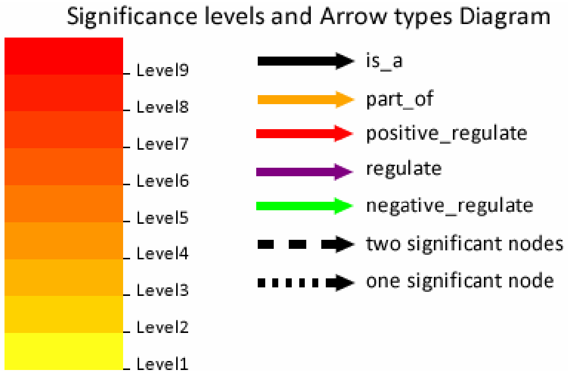

(B) Rice_Molecular Function

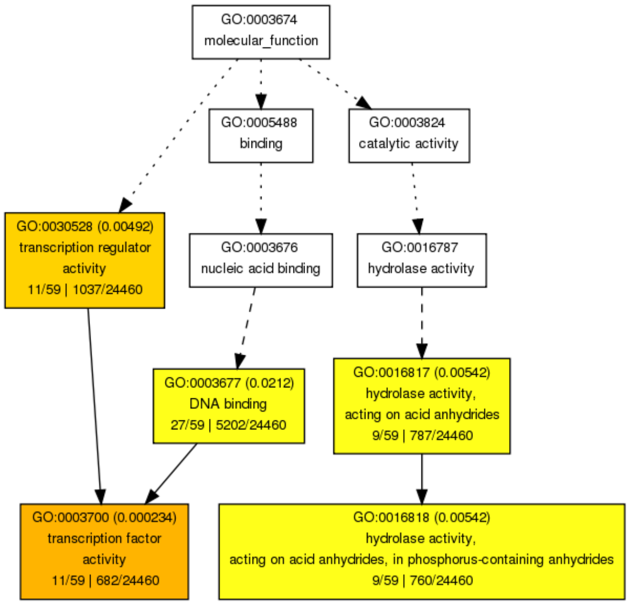

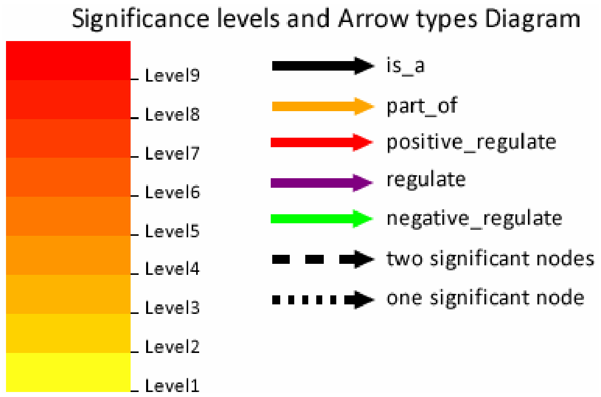

(C) Arabidopsis_Biological Process

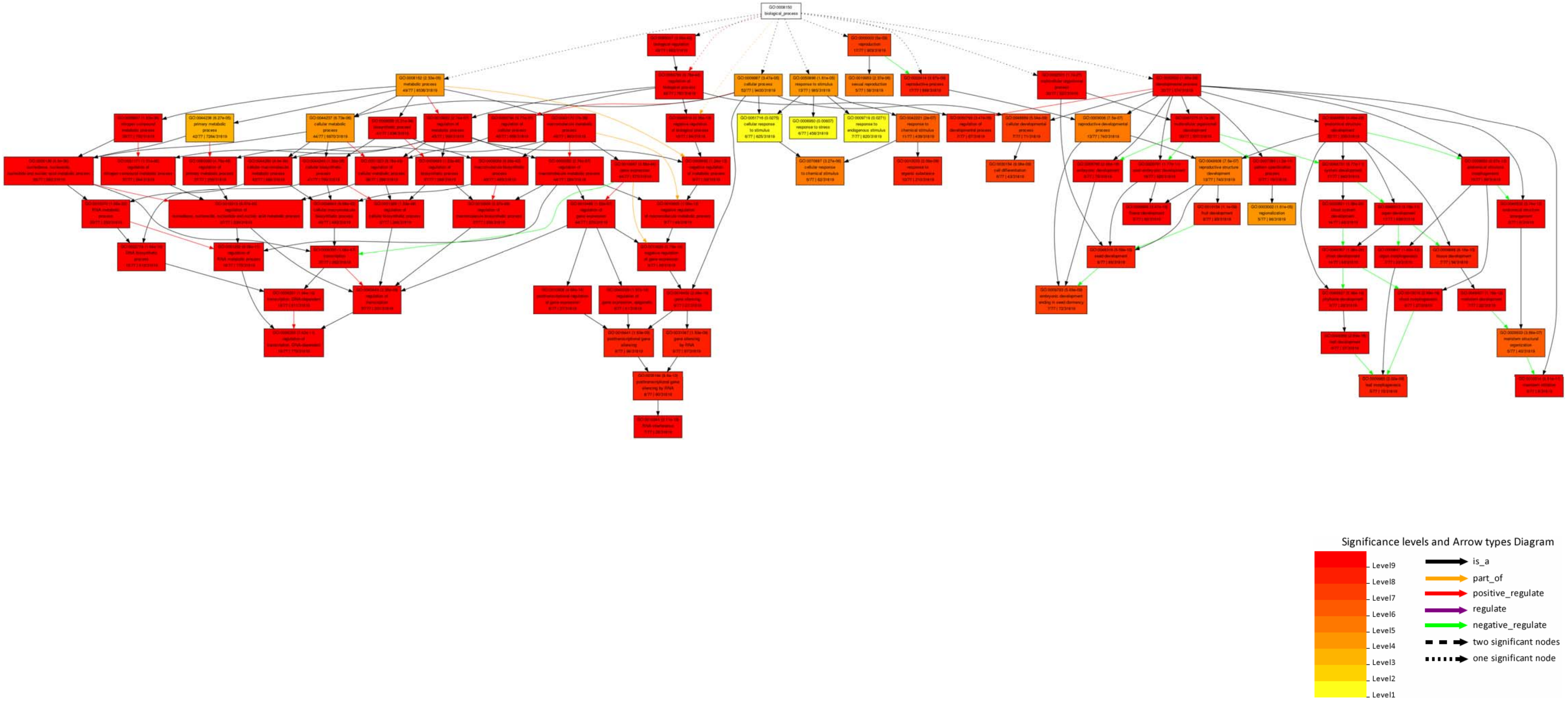

(D) Rice_ Biological Process

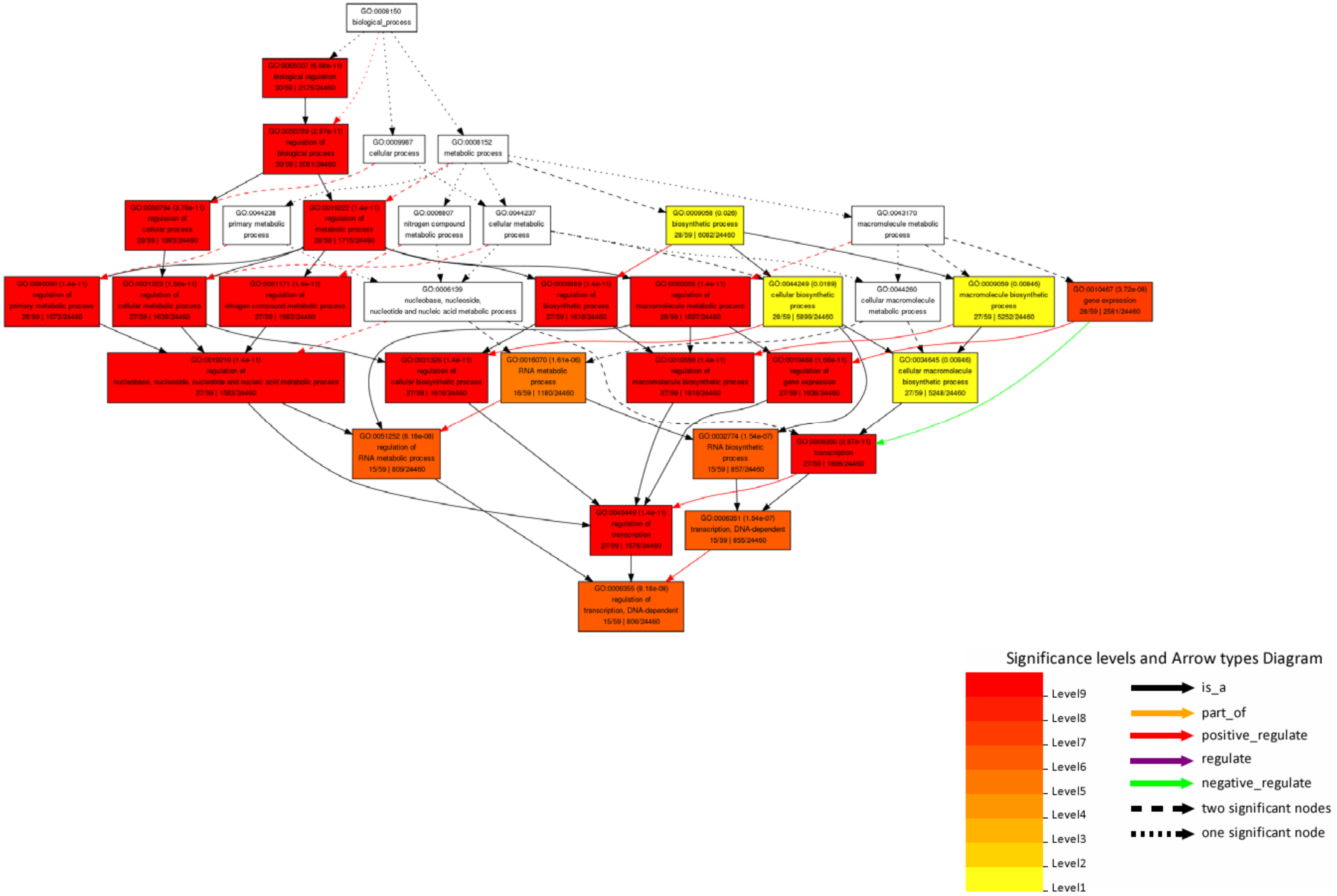

Supplement: Additional file 6 — Figure S3. GO (Gene Ontology) term enrichment analysis of the targets of sequestered microRNAs in Arabidopsis and rice. [file 1471-2164-13-197-S6.pdf]
